# Oral Presentation

**DOI:** 10.1111/ene.70189

**Published:** 2025-06-21

## Saturday, June 21 2025

## Motor Neurone Diseases

## OPR‐001

### Glymphatic dysfunction in clinical phenotypes of motor neuron disease

#### I. Bottale ^1^; E. Spinelli^1^; S. Basaia^1^; A. Ghirelli^1^; T. Russo^2^; E. Canu^1^; V. Castelnovo^1^; P. Schito^2^; Y. Falzone^2^; M. FIlippi^2^; F. Agosta^1^

##### ^1^Neuroimaging Research Unit, Division of Neuroscience, IRCCS San Raffaele Scientific Institute, Milan, Italy; ^2^Neurology Unit, IRCCS San Raffaele Scientific Institute, Milan, Italy

**Background and Aims:** Converging evidence supports a key pathogenic role of the glymphatic system in the accumulation of pathological aggregates in several proteinopathies, including amyotrophic lateral sclerosis (ALS) and other motor neuron diseases (MNDs). This study aimed to verify glymphatic function impairment calculating diffusion tensor imaging analysis along the perivascular space (DTI‐ALPS) and explore its clinical correlations in MND phenotypes.

**Methods:** Fifty‐seven MND patients (41 ALS, 7 with lower motor neuron, and 9 with upper motor neuron presentations) and 32 age‐ and sex‐matched healthy controls underwent 3 Tesla brain MRI, including DTI sequences. We obtained DTI‐ALPS index from each individual, evaluating its relationship with measures of motor and cognitive disability, site of symptom onset, cognitive status, and fractional anisotropy (FA) of white matter tracts. Comparisons between groups were evaluated using ANCOVA models, age‐ and sex‐adjusted. Partial correlations with clinical and cognitive measures were also tested.

**Results:** MND patients exhibited significantly reduced DTI‐ALPS index values compared to healthy controls (*p* < 0.001). Patients with bulbar onset had lower DTI‐ALPS values than those with spinal onset (*p* = 0.046). Similar DTI‐ALPS values were found across all MND phenotypes, with no effect of cognitive diagnosis or C9orf72 expansion status. Significant correlations were identified between DTI‐ALPS and disease duration (*r* = −0.30, *p* = 0.03), as well as FA values in the anterior corona radiata (*r* = 0.31, *p* = 0.02) and body of the corpus callosum (*r* = 0.37, *p* = 0.049).

**Conclusion:** This study confirms glymphatic dysfunction across MND phenotypes, particularly in bulbar‐onset cases, supporting a pathogenic involvement of this system for the accumulation of TDP‐43 proteinopathy in MND.

**Disclosure:** Supported by European Research Council (StG‐2016_714388_NeuroTRACK); Next Generation EU, in the context of the National Recovery and Resilience Plan, Investment PE8 ‐ Project Age It: “Ageing Well in an Ageing Society”. F. Agosta received speaker honoraria from Biogen Idec, Roche, Eli Lilly, GE Healthcare; receives research supports from IMH, IMUR, AriSLA, ERC, EU JPND Research, Foundation Research on AD (France). M. Filippi is Editor‐in‐Chief of the Journal of Neurology, Associate Editor of Human Brain Mapping, Neurological Sciences, and Radiology; received compensation for consulting services from Alexion, Almirall, Biogen, Merck, Novartis, Roche, Sanofi; speaking activities from Bayer, Biogen, Celgene, Chiesi Italia SpA, Eli Lilly, Genzyme, Janssen, Merck‐Serono, Neopharmed Gentili, Novartis, Novo Nordisk, Roche, Sanofi, Takeda, and TEVA; participation in Advisory Boards for Alexion, Biogen, Bristol‐Myers Squibb, Merck, Novartis, Roche, Sanofi, Sanofi‐Aventis, Sanofi‐Genzyme, Takeda; scientific direction of educational events for Biogen, Merck, Roche, Celgene, Bristol‐Myers Squibb, Lilly, Novartis, Sanofi‐Genzyme; he receives research support from Biogen Idec, Merck‐Serono, Novartis, Roche, the Italian Ministry of Health, the Italian Ministry of University and Research, and Fondazione Italiana Sclerosi Multipla.

## OPR‐002

### Tofersen in the treatment of SOD1 ALS—experience from the Polish EAP

#### M. Kuzma‐kozakiewicz ^1^; M. Dorobek^2^; A. Gruszczak^2^; J. Zielinska^2^; H. Bartosik‐Psujek^3^; T. Garbacz^3^; K. Zur0Wyrozumska^4^; D. Kozaiorowski^5^; A. Dutkiewicz^5^; E. Krzyzanowska^5^; B. Poczatek^6^; A. Lasek‐Bal^7^; A. Migacz^7^; M. Koszewicz^8^; S. Budrewicz^8^; K. Szabat^9^; K. Tomicka^9^

##### ^1^Department of Neurology, Medical University of Warsaw, Warsaw, Poland; ^2^Department of Neurology, Central Clinical Hospital of the Ministry of Interior in Warsaw, Warsaw, Poland; ^3^Department of Neurology, Faculty of Medicine, University of Rzeszow, Rzeszow, Poland; ^4^Department of Neurology, The Józef Dietl Specialist Hospital; Department of Medical Education, Jagiellonian University Medical College, Kraków, Poland; ^5^Department of Neurology, Faculty of Health Sciences, Medical University of Warsaw, 03‐242 Warsaw, Poland; ^6^Department of Neurology, Faculty of Health Sciences, Medical University of Warsaw, 03‐242 Warsaw, Poland; ^7^School of Health Sciences in Katowice, Medical University of, Katowice, Poland; ^8^Department of Neurology, Wroclaw Medical University, Wroclaw, Poland; ^9^7th Naval Hospital in Gdańsk, Gdańsk, Poland

**Background and Aims:** Tofersen is approved for the treatment of amyotrophic lateral sclerosis (ALS) caused by SOD1 mutations. We prospectively analyzed its effectiveness in participants of the expanded access programs (EAP) in Poland.

**Methods:** Twenty SOD1‐ALS patients (11 FALS and 9 SALS, 65% males) qualified to EAP between 10.2023 and 08.2024. The analysis included the demographic and genetic data, ALS functional rating scale‐revised (ALSFRS‐R), delta ALSFRS‐R, forced vital capacity (FVC), and NfL CSF concentration.

**Results:** Median age of disease onset was 51 years, age at tofersen administration ‐ 53 years and the disease duration ‐ 28 months. The mean duration of tofersen treatment was 11 months (5–15 months). Mean delta ALSFRS‐R delta ranged from 0,19 prior to treatment to 0,14 at the last assessment. One patient withdrew after the first tofersen administration due to clinical state. Twelve patients (63%) showed an increase of ALSFRS‐R by 1–2 points in the treatment period, 4 patients (21%) stabilized, while 3 patients (16%) showed further progression. The NfL concentration decreased in 6/7 of analyzed patients, while delta ALSFRS‐R decreased in 80% of patients with stable/increased functional state and only 33% with decreasing ALSFRS‐R. The treatment was well tolerated in 95% of patients. One patient experienced SAE after the 9th infusion – a transverse myelitis with fever, myalgia and gait disturbance (ALSFS‐R drop from 40 to 39/48), which completely resolved within 8 weeks after iv methylprednisolone treatment and physical therapy.

**Conclusion:** the data supports the clinical andë molecular response to tofersen in SOD1‐ALS.

**Disclosure:** Authors participated in the advisory boards for Biogen (MKK, MK) and Ferrer (MKK); MKK obtained financial compensations from Biogen and Ferrer for lectures on ALS.

## OPR‐003

### Predictors in late‐stage amyotrophic lateral sclerosis

#### M. Fortuna Baptista ^1^; M. Gromicho^2^; I. Alves^2^; M. Oliveira Santos^1^; M. de Carvalho^1^

##### ^1^Department of Neurosciences and Mental Health, Unidade Local de Saúde de Santa Maria, Lisbon, Portugal; ^2^Instituto Gulbenkian de Medicina Molecular, Lisbon, Portugal

**Background and Aims:** Prognostic factors in amyotrophic lateral sclerosis (ALS) are defined by clinical features, progression rate and physiological changes at first observation or over follow‐up. The prognostic factors associated with late‐stage disease are uncertain. We sought to identify factors predicting survival in advanced ALS.

**Methods:** We collected data from patients followed at our clinic and analysed a subgroup with late‐stage ALS defined as ALSFRS‐R≤24. We characterized this population by examining demographic and clinical variables, including phenotype, sex, age, disease duration at diagnosis, non‐invasive ventilation (NIV), percutaneous endoscopic gastrostomy (PEG), early and late disease progression rates measured by ALSFRS‐R score and survival. Multivariate analysis with Cox regression was performed to ascertain predictive factors for survival in late‐stage.

**Results:** We included 704 late‐stage ALS patients (Group A) with 260 having at least 6 more months of follow‐up (Group B). Factors associated with short survival in late‐stage were onset‐age (age > 54 years, HR 2.15, 1.49–3.09), bulbar‐onset (HR 1.67, 1.05–1.87), diagnostic delay (≥ 12 months, HR 0.61, 0.40–0.92) and progression rate until late‐stage (ΔFS until late‐stage> 0.55, HR 3.56, 2.25–5.64). PEG and NIV in the late‐stage were not independent predictors of survival.

**Conclusion:** Independent predictors of late‐stage ALS survival include onset‐age, onset‐region, diagnostic delay, and functional decline (ΔFS) until late‐stages (but not at diagnosis). For this group, monitoring functional decline during follow‐up is valuable for prognosis.

**Disclosure:** Nothing to disclose.

## OPR‐004

### Abstract withdrawn

## OPR‐005

### Identifying mild cognitive and behavioural dysfunction in amyotrophic lateral sclerosis provides key prognostic insights

#### V. Iuzzolino ^1^; M. Spisto^2^; G. Senerchia^1^; L. Aruta^1^; R. Dubbioso^1^

##### ^1^Department of Neurosciences, Reproductive Sciences and Odontostomatology, University Federico II of Naples, Naples, Italy; ^2^Department of Psychology, University of Campania Luigi Vanvitelli, Naples, Italy

**Background and Aims:** Amyotrophic lateral sclerosis (ALS) is a multisystem neurodegenerative disease encompassing cognitive and behavioral impairments. The Revised Diagnostic Criteria for ALS‐frontotemporal spectrum disorder (ALS‐FTDS), while widely adopted, may overlook subtle impairments such as memory and visuospatial deficits, limiting their prognostic value. This study aimed to apply the Mild Neurocognitive‐Behavioural Dysfunction (MiND) approach, adapted from other neurodegenerative diseases to ALS patients and assessed its prognostic utility for survival and disease progression.

**Methods:** A prospective cohort of 201 ALS patients was evaluated between January 2018 and July 2024. Participants underwent comprehensive cognitive and behavioral assessments. The MiND approach identified patients with mild cognitive impairment (MCI), mild behavioral impairment (MBI), or combined mild cognitive‐behavioral impairment (MCBI). Prognostic value was analyzed using Kaplan‐Meier survival curves, Cox proportional hazards models, and logistic regression for disease progression, adjusting for clinical covariates.

**Results:** MiND was identified in 67% of patients previously classified as cognitively normal by the Revised Diagnostic Criteria for ALS‐FTDS. At a median follow‐up of 15 months, these patients had shorter tracheostomy‐free survival compared to those with normal cognition (all *p* < 0.005). Mild cognitive impairment (HR 5.7; CI 1.26–25.82; *p*  =  0.024) and frontotemporal dementia (HR 4.7; CI 1.01–21.4; *p*  =  0.04) independently predicted poor outcomes. Logistic regression showed mild cognitive‐behavioral impairment and frontotemporal dementia were associated with rapid disease progression (both *p* < 0.019).

**Figure:**
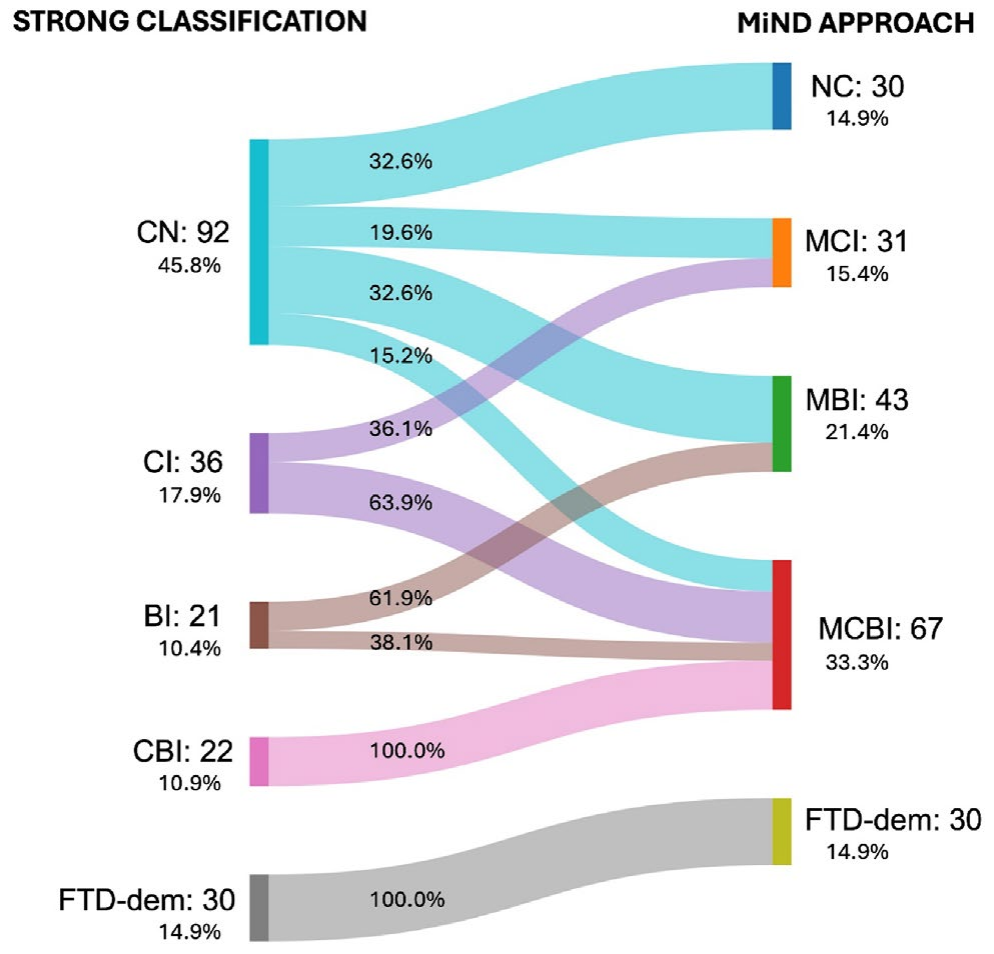
**FIGURE 1** Sankey Diagram Comparing Patient Transitions Between Strong classification and MiND approach for Cognitive and Behavioral Impairments in ALS.

**Figure:**
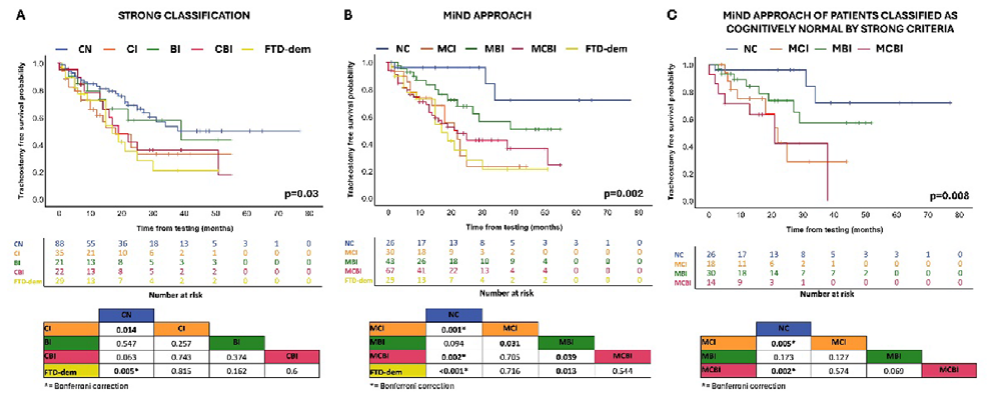
**FIGURE 2** Survival analysis according to MiND approach, Strong criteria, and in the subgroup of patients classified as cognitively normal according to Strong criteria.

**Figure:**
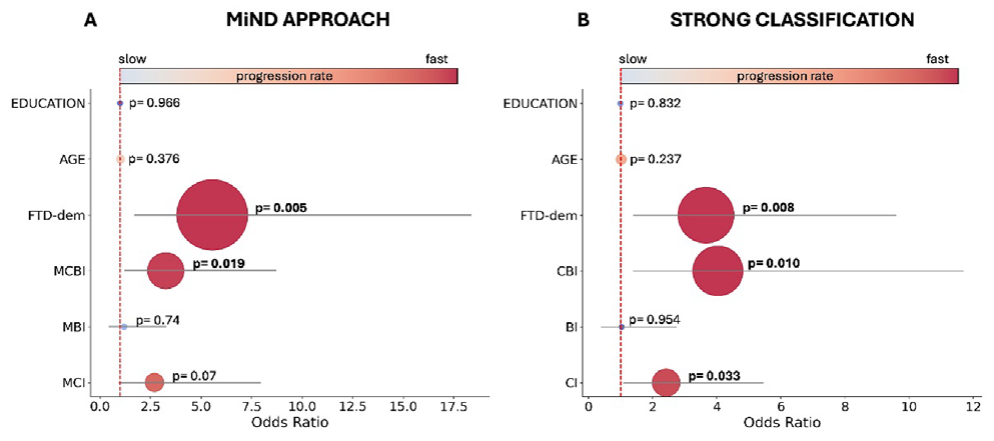
**FIGURE 3** Binary logistic regression for disease progression rate

**Conclusion:** The MiND approach enhances detection of mild cognitive and behavioral impairments in ALS, providing prognostic insights and improving stratification over ALS‐FTDS criteria. This supports personalized care and clinical trial design for early disease stages.

**Disclosure:** Nothing to disclose.

## OPR‐006

### The brain functional neural organization of apathy and depression in ALS: A connectome‐based study

#### V. Castelnovo ^1^; E. Canu^1^; S. Basaia^2^; E. Spinelli^3^; F. Freri^1^; P. Schito^4^; T. Russo^4^; Y. Falzone^4^; F. Verde^5^; S. Torre^6^; B. Poletti^7^; L. Tremolizzo^8^; I. Appollonio^8^; N. Ticozzi^5^; V. Silani^5^; M. Filippi^9^; F. Agosta^3^

##### ^1^Neuroimaging Research Unit, Division of Neuroscience, and Neurology Unit, IRCCS San Raffaele Scientific Institute, Milan, Italy; ^2^Neuroimaging Research Unit, Division of Neuroscience, IRCCS San Raffaele Scientific Institute, Milan, Italy; ^3^Neuroimaging Research Unit, Division of Neuroscience, and Neurology Unit, IRCCS San Raffaele Scientific Institute, and Vita‐Salute San Raffaele University, Milan, Italy; ^4^Neurology Unit, IRCCS San Raffaele Scientific Institute, Milan, Italy; ^5^Department of Neurology and Laboratory of Neuroscience, IRCCS Istituto Auxologico Italiano, and Department of Pathophysiology and Transplantation, “Dino Ferrari” Center, Università degli Studi di Milano, Milan, Italy; ^6^Department of Neurology and Laboratory of Neuroscience, IRCCS Istituto Auxologico Italiano, Milan, Italy; ^7^Department of Neurology and Laboratory of Neuroscience, IRCCS Istituto Auxologico Italiano, and Department of Oncology and Hemato‐Oncology, Università degli Studi di Milano, Milano, Italy; ^8^Neurology Unit, “San Gerardo” Hospital and University of Milano‐Bicocca, Monza, Italy; ^9^Neurology Unit, Neurorehabilitation Unit, Neurophysiology Service, and Neuroimaging Research Unit, Division of Neuroscience, IRCCS San Raffaele Scientific Institute, and Vita‐Salute San Raffaele University, Milan, Italy

**Background and Aims:** Apathy and depression are the most prevalent neuropsychiatric symptoms in Amyotrophic Lateral Sclerosis (ALS). Although insufficiently investigated, their distinction holds important clinical relevance for accurate diagnosis of ALS with behavioural impairment, and for patients’ prognosis and management. In the present study, we aimed to assess both apathy and depressive symptoms in patients with ALS and whether they have similar or different functional neural correlates.

**Methods:** Using graph analysis and connectomics, global and lobar nodal properties and regional functional brain connectivity were assessed in ALS patients without apathy/depression (ALSn, *n* = 42), with apathy without depression (ALSa, *n* = 14), with depressive symptoms without apathy (ALSd, *n* = 20), with apathy and depressive symptoms (ALSad, *n* = 6) and 46 healthy controls. Correlations between brain functional properties, apathy and depressive symptoms were performed in all patients.

**Results:** Depressive symptoms were related with reduced path length within bilateral basal ganglia (BG) network, apathy was related with increased path length, decreased nodal strength and local efficiency within left BG network. ALSa patients showed altered functional nodal properties within BG network compared to ALSn and ALSd. Compared to healthy controls and all non‐apathetic patients (ALSn and ALSd), all apathetic patients (ALSa and ALSad) exhibited altered functional nodal properties within parietal, occipital and frontal networks. Non‐apathetic patients, compared to apathetic patients, showed relatively preserved functional nodal properties in the BG network.

**Conclusion:** Our findings indicate differences in brain functional neural organization associated with apathy and depression, underscoring the importance of distinguishing these symptoms in ALS and highlighting the need for targeted interventions.

**Disclosure:** Funding: ERC (StG‐2016_714388_NeuroTRACK); Next Generation EU, in the context of the National Recovery and Resilience Plan, Investment PE8 ‐ Project Age‐It: “Ageing Well in an Ageing Society”. Disclosures. EC and SB received grants from Italian Ministry of Health (IMH); FV is Associate Editor (AE) JAD; BP received compensation from Liquidweb srl; AE Frontiers in Neuroscience; VS received compensation from AveXis, Cytokinetics, Italfarmaco, Liquidweb S.r.l., Novartis Pharma AG, Amylyx Pharmaceuticals, Biogen and Zambon Biotech SA; received supports from IMH, AriSLA, E‐Rare JTC; Editorial Board of ALS/FTD, European Neurology, AJND, Frontiers in Neurology, Exploration of Neuroprotective Therapy; MF received compensation from Alexion, Almirall, Bayer, Biogen, Celgene, Chiesi Italia SpA, Eli Lilly, Genzyme, Janssen, Merck‐Serono, Neopharmed Gentili, Novartis, Novo Nordisk, Roche, Sanofi Takeda, TEVA; Advisory Boards for Alexion, Biogen, Bristol‐Myers Squibb, Merck, Novartis, Roche, Sanofi, Sanofi‐Aventis, Sanofi‐Genzyme, Takeda; scientific direction of events for Biogen, Merck, Roche, Celgene, Bristol‐Myers Squibb, Lilly, Novartis, Sanofi‐Genzyme; receives research support from Biogen Idec, Merck‐Serono, Novartis, Roche, IMH, Italian Ministry of University and Research (IMUR), FISM; FA received speaker honoraria from Biogen Idec, Roche, Eli Lilly, GE Healthcare; receives research supports from IMH, IMUR, AriSLA, ERC, EU JPND Research, Foundation Research on AD (France).

## Headache 1

## OPR‐007

### Probability of response to subcutaneous anti‐CGRP monoclonal antibodies in migraine: How long should we wait?

#### A. Jaimes; J. Rodriguez‐Vico; A. Gómez; A. Nystrom Hernández; O. Pajares; J. Porta‐Etessam

##### Headache Unit, Neurology Department, Fundación Jiménez Díaz University Hospital

**Background and Aims:** Guidelines recommend evaluating the response to anti‐CGRP monoclonal antibodies (A‐CGRP mAbs) at 3–6 months, but studies analyzing month‐by‐month response probability are lacking.

**Methods:** Cumulative and instantaneous response probabilities were analyzed in patients treated with subcutaneous A‐CGRP mAbs, defining response as a ≥50% reduction in headache frequency. Kaplan‐Meier analysis was used to estimate survival function and response probabilities. Cox regression evaluated clinical covariates.

**Results:** Among 462 patients (76.6% with chronic migraine; 86.8% female; median age 48 years, IQR 41–56), cumulative response probability increased from 36.1% (95% CI: 31.6–40.4) in the first month to 72% (95% CI: 66.7–76.6) at 12 months. Monthly instantaneous response probability decreased from 36.1% (95% CI: 31.6–40.4) in the first month to 14.9% (95% CI: 14.5–15.4) in the third month, remaining below 10% from the fourth month onward. Multivariable analysis showed higher response probability with hemicranial pain (HR 1.31, 95% CI: 1.01–1.70) and photophobia (HR 1.64, 95% CI: 1.07–2.52), but lower in males (HR 0.68, 95% CI: 0.46–0.99), with more prior preventives (HR 0.95, 95% CI: 0.91–0.99), and higher baseline headache frequency (HR 0.97, 95% CI: 0.95–0.99).

**Figure:**
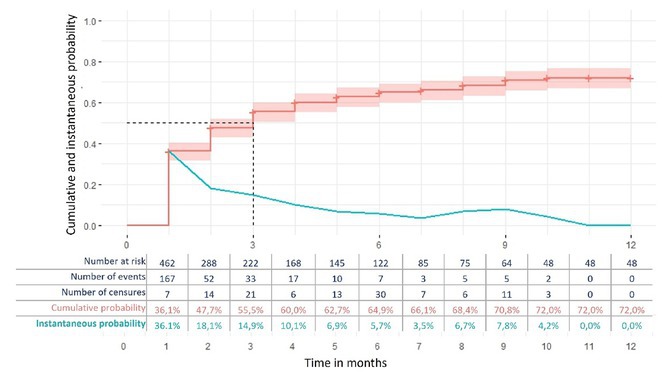
**FIGURE 1** Cumulative and instantaneous probability

**Figure:**
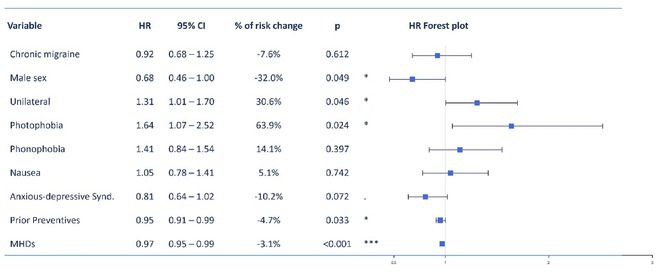
**FIGURE 2** Multivariable Cox Regression

**Conclusion:** The highest response probability occurred in the first trimester and was influenced by factors such as baseline headache burden and photophobia. Although late responses are possible, the low probabilities suggest considering a treatment change unless it represents the last therapeutic option.

**Disclosure:** Nothing to disclose.

## OPR‐008

### European multicenter study on the use of anti‐CGRP monoclonal antibodies in migraine: The 2‐year follow‐up

#### E. Caronna ^1^; J. Canales^1^; L. Dorado^23^; R. Álvarez Escudero^2^; I. Pavão Martins^3^; C. Sundal^4^; P. Irimia Sieira^5^; A. Lozano Ros^6^; A. Gago‐Viega^7^; F. Velasco Juanes^8^; R. Ruscheweyh^9^; S. Sacco^10^; D. Garcia‐Azorin^11^; J. Pascual^12^; R. Gil‐Gouveia^13^; M. Huerta Villanueva^14^; J. Rodriguez‐Vico^15^; S. Santos Lasaosa^16^; M. Ghadiri‐Sani^17^; R. De Icco^18^; J. Diaz de Terán^19^; S. Diaz Insa^20^; C. Gonzalez Oria^21^; P. Barbanti^22^; P. Pozo‐Rosich^1^

##### ^1^Headache Clinic, Neurology Department, Vall d’Hebron Hospital, Barcelona, Spain; ^2^Unidad de cefaleas, Hospital Universitario Central de Asturias; ^3^Hospital Universitario Sta Maria, Faculty of Medicine University of Lisbon &, Campus Neurologico, Lisbon, Portugal; ^4^NeuroClinicNorway, Dep.of Neurology, Norway; ^5^Department of Neurology. Clinica Universidad de Navarra. Pamplona. Spain; ^6^Headache Unit, Hospital General Universitario Gregorio Marañón, Madrid, Spain; ^7^Headache Unit. Neurology Department. Hospital Universitario La Princesa; ^8^Neurology Department. Hospital Universitario Cruces. Biocruces Bizkaia Health Research Institute. Bilbao. Spain; ^9^Department of Neurology, LMU University Hospital, LMU Munich, Munich, Germany; ^10^Department of Biotechnological and Applied Clinical Sciences, University of L’Aquila, L’Aquila, Italy; ^11^Headache Unit, Department of Neurology, Hospital Clínico Universitario de Valladolid, Spain; ^12^University Hospital Marqués de Valdecilla, Santander, Spain; ^13^Hospital da Luz Headache Center, Neurology Department, Hospital da Luz – Lisboa; ^14^Headache Unit, Hospital de Bellvitge, Barcelona, Spain; ^15^Headache Unit. Hospital Universitario Fundación Jiménez Díaz. Madrid; ^16^Headache Unit, University of Zaragoza, Zaragoza, Spain; ^17^The Walton Centre NHS Foundation Trust, Liverpool, UK; ^18^Department of Brain and Behavioral Sciences, University of Pavia, Pavia, Italy; 9. Headache Science & Neurorehabilitation Center, IRCCS Mondino Foundation, Pavia, Italy; ^19^Headache Unit, Neurology Department, La Paz University Hospital. Madrid (Spain); ^20^Hospital Universitario La Fe, Valencia, Spain; ^21^Unidad de Cefaleas, Hospital Universitario Virgen del Rocío, Sevilla, Spain; ^22^Headache and Pain Unit, IRCCS San Raffaele Roma, Italy; ^23^Department of neurosciences, Germans Trias i Pujol University Hospital

**Background and Aims:** To describe the long‐term effectiveness of monoclonal antibodies targeting calcitonin gene‐related peptide (anti‐CGRP mAbs) in a European cohort of migraine patients.

**Methods:** European multicenter, observational study based on prospective registries of adult patients with high‐frequency episodic or chronic (CM) migraine treated with anti‐CGRP mAbs. We collected demographic data, efficacy variables (monthly headache days‐MHD; monthly migraine days‐MMD) up to 24 months (M24). We compared patients reaching M24 (ON‐group) with those who discontinued the treatment due to lack of effectiveness at any time (OFF‐group).

**Results:** 1340 patients reached M24 (ON‐group: median age 48 years [41,55], 81.7% (1095/1340) females). Median basal frequencies at baseline were: 20 (13, 28) MHD and 15 (11, 21) MMD. At M24, the median reductions were: −10 (−17, −5) in MHD and −9 (−14, −4) MMD. At baseline, 936/1340 (69.9%) had CM; of these 746/936 (79.7%) still fulfilled CM diagnosis at M24, whereas 190/936 (20.3%) converted to EM. 1057 patients discontinued the treatment due to efficacy (OFF‐group). Compared to the ON‐group, there were no differences in sex or age, but the OFF‐group had statistically significant higher proportions of CM (ON: 69.9% vs. OFF: 82.9%), depression (ON: 24.0% vs. OFF: 38.0%), anxiety (ON: 30.7% vs. OFF: 41.0%) and obesity (ON: 7.2% vs. OFF: 19.1%) (*p* < 0.001).

**Conclusion:** For patients reaching 2 years of treatment, effectiveness is similar to the one reported at short‐term. Chronification, psychiatric and metabolic comorbidities hinder treatment response, making early migraine prevention and management of comorbid conditions essential for better care

**Disclosure:** EC has received honoraria from Novartis, Chiesi, Lundbeck, MedScape, Lilly; his salary has been partially funded by Río Hortega grant Acción Estratégica en Salud 2017–2020 from Instituto de Salud Carlos III (CM20/00217) and Juan Rodés fellowship, Subprograma Estatal de Incorporación de la Acción Estratégica en Salud 2023 (JR23/00065). PPR has received, in the last three years, honoraria as a consultant and speaker for: AbbVie, Biohaven, Chiesi, Eli Lilly, Lundbeck, Medscape, Novartis, Pfizer and Teva. Her research group has received research grants from AbbVie, Novartis and Teva; as well as, Instituto Salud Carlos III, EraNet Neuron, European Regional Development Fund (001‐P‐001682) under the framework of the FEDER Operative Programme for Catalunya 2014‐2020 ‐ RIS3CAT; has received funding for clinical trials from AbbVie, Amgen, Biohaven, Eli Lilly, Novartis, Teva. She is the Honorary Secretary of the International Headache Society.

## OPR‐009

### Neurosurgical interventions in idiopathic intracranial hypertension: A multicenter study on outcome and referral pattern

#### G. Bsteh ^1^; N. Hansen^3^; S. Zaic^2^; S. Hamann^4^; J. Korsbæk^3^; N. Krajnc^1^; S. Macher^1^; L. Molander^5^; K. Novak^6^; M. Wegener^4^; B. Pemp^7^; R. Jensen^3^; D. Beier^8^

##### ^1^Department of Neurology, Medical University of Vienna, Vienna, Austria; ^2^Medical University of Vienna, Comprehensive Center for Clinical Neurosciences & Mental Health, Vienna, Austria; ^3^Danish Headache Center, Department of Neurology, Rigshospitalet, Glostrup, Denmark; ^4^Department of Ophthalmology, Rigshospitalet, University of Copenhagen, Denmark; ^5^Department of Ophthalmology, Odense University Hospital, Odense, Denmark; ^6^Department of Neurosurgery, Medical University of Vienna, Vienna, Austria; ^7^Department of Ophthalmology, Medical University of Vienna, Vienna, Austria; ^8^Department of Neurology, Odense University Hospital, Denmark

**Background and Aims:** Neurosurgical interventions are recommended for fulminant or treatment‐refractory idiopathic intracranial hypertension (IIH), but evidence on their outcomes, particularly regarding referral patterns and indications, is limited. We evaluated clinical outcomes and referral patterns for neurosurgical interventions in IIH, identifying predictors of beneficial or adverse outcomes.

**Methods:** A retrospective multicenter study was conducted by the Danish‐Austrian IIH Consortium (DASH‐IIH) across three centers (Vienna, Odense, Copenhagen). Patients with IIH meeting revised Friedman criteria who underwent neurosurgical intervention between 2014 and 2024, with at least six months of follow‐up, were included. Outcomes assessed at six months post‐intervention included visual function, headache frequency (monthly headache days [MHD]), papilledema resolution, and severe adverse events (CTCAE grade ≥3).

**Results:** Thirty‐six female patients were included (mean age 32.5 years, median BMI 37.0, median CSF opening pressure 41 cmH₂O). Ventriculo‐peritoneal shunting (VPS) was performed in 27 (75%) patients and optic nerve sheath fenestration (ONSF) in 9 (25%). Acute or imminent visual loss was the primary indication in 83.3%, while 16.7% were referred for refractory headache. Visual function improved in 41.7%, papilledema resolved in 89.7%, and 30.6% experienced a ≥50% reduction in MHD (median reduction 4.5 days). Multivariate analysis showed no significant differences in outcomes or adverse events between VPS and ONSF. Referrals for refractory headache alone did not result in visual improvement (0%) and were significantly less likely to reduce headache frequency (OR 0.11, *p* = 0.012).

**Conclusion:** VPS and ONSF are effective for acute or imminent visual loss in IIH. However, refractory headache alone may be an inappropriate indication for neurosurgical referral.

**Disclosure:** Gabriel Bsteh: has participated in meetings sponsored by, received speaker honoraria or travel funding from Biogen, Celgene/BMS, Lilly, Merck, Novartis, Roche, Sanofi‐Genzyme and Teva, and received honoraria for consulting Biogen, Celgene/BMS, Merck, Novartis, Roche, Sanofi‐Genzyme and Teva. He has received unrestricted research grants from Celgene/BMS and Novartis.

## OPR‐010

### Acute headache treatment in idiopathic intracranial hypertension: Treating to the phenotype?

#### G. Bsteh ^1^; N. Krajnc^1^; S. Zaic^2^; N. Müller^1^; W. Marik^3^; M. Michl^4^; K. Novak^5^; S. Macher^1^; B. Pemp^4^; C. Wöber^1^

##### ^1^Department of Neurology, Medical University of Vienna, Vienna, Austria; ^2^Comprehensive Center for Clinical Neurosciences & Mental Health, Medical University of Vienna; ^3^Department of Neuroradiology, Medical University of Vienna, Vienna, Austria; ^4^Department of Ophthalmology, Medical University of Vienna, Vienna, Austria; ^5^Department of Neurosurgery, Medical University of Vienna, Vienna, Austria

**Background and Aims:** Effective acute headache treatment is essential for improving the quality of life in people with idiopathic intracranial hypertension (pwIIH), though current guidance to “treat to the phenotype” lacks robust data.

**Methods:** This retrospective analysis used standardized headache diaries from the Vienna Idiopathic Intracranial Hypertension (VIIH) database (1‐JUL‐2021 to 30‐JUN‐2023). Three classes of acute medications (acetaminophen [APAP], NSAIDs, and triptans) were analyzed, with NSAIDs and ibuprofen as references. Headache attacks were classified per ICHD‐3 as migraine (M), tension‐type (TTH), or other (O). A 2‐level nested logistic regression adjusted for individual covariance and propensity‐weighted for age, sex, and headache severity.

**Results:** We analyzed 35,640 medication‐outcome pairs from 23,507 headache attacks (45.3% M, 21.1% TTH, 33.6% O) in 156 patients (89.7% female, mean age 32.9 years). NSAIDs were most commonly used (M: 60.5%, TTH: 69.8%, O: 70.7%), followed by APAP and triptans. Triptans were the most effective across all headache types (OR for M: 4.8 [CI 3.9–6.1], TTH: 2.9 [CI 1.8–4.3], O: 3.1 [CI 2.2–4.3]). APAP was less effective for migraine (OR 0.81 [CI 0.74–0.90]) but similar to NSAIDs in TTH and O. In migraine, eletriptan and zolmitriptan (OR 6.0 and 5.8) were slightly more effective than sumatriptan (OR 5.0).

**Figure:**
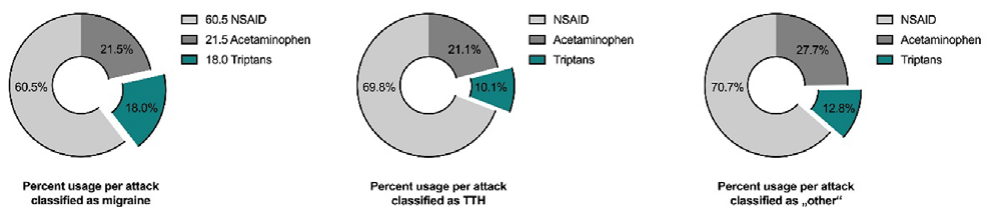
FIGURE 1

**Conclusion:** Triptans outperform NSAIDs and APAP in treating headaches in pwIIH, particularly for migraine‐type attacks. These findings support the preferential use of triptans and challenge the “treating to the phenotype” approach.

**Disclosure:** GB: has participated in meetings sponsored by, received speaker honoraria or travel funding from Biogen, Celgene/BMS, Lilly, Merck, Novartis, Roche, Sanofi‐Genzyme and Teva, and received honoraria for consulting Biogen, Celgene/BMS, Novartis, Roche, Sanofi‐Genzyme and Teva. He has received unrestricted research grants from Celgene/BMS and Novartis.

## OPR‐011

### CGRP increase in tear fluid of migraine patients is reversed by anti‐CGRP monoclonal antibodies

#### M. Romozzi ^1^; L. Di Nardo^2^; V. Trigila^1^; G. Cuffaro^3^; G. Savino^3^; L. Iannone^4^; C. Vollono^1^; P. Calabresi^1^

##### ^1^Department of Neurology, Fondazione Policlinico Univeristario Agostino Gemelli IRCCS, Rome, Italy; ^2^Immunology Facility, Fondazione Policlinico A. Gemelli IRCCS, Rome, Italy; ^3^Department of Ophthalmology, Fondazione Policlinico Univeristario Agostino Gemelli IRCCS, Rome, Italy; ^4^Department of Life Sciences, University of Modena and Reggio Emilia, Modena, Italy

**Background and Aims:** CGRP has emerged as a key player in migraine pathophysiology, but challenges remain in its use as a biomarker. The eye is richly innervated by trigeminal fibers, making CGRP measurement in tear fluid a possible direct assessment of trigeminal activation. This study aimed to compare CGRP in tear fluid of migraine patients compared to healthy controls (HCs).

**Methods:** Tear fluid was collected from migraine patients and HCs through Schirmer test strips; CGRP concentration was assayed using ELISA. Clinical characteristics of migraine, severity and disability scores were collected. As a proof‐of‐concept, tear CGRP levels were measured before starting anti‐CGRP monoclonal antibodies and after 6 months (T6).

**Results:** 51 patients with migraine (9 [17.6%] chronic, 16 with aura [31.4%]) and 24 age‐matched HCs were included. Tear CGRP concentrations were significantly elevated in migraine patients (7.4±7.6pg/mL) than HCs (3.5±4.4pg/mL) (*p* = 0.022). In the migraine group, tear CGRP levels were higher in the ictal phase (10.59±7.7pg/mL) compared to the interictal phase (5.8±7.3pg/mL) and in patients with aura (10.4 ± 9.2pg/mL) versus without (6.1±6.4pg/mL) (*p* = 0.042). The ROC curve analysis for CGRP revealed an AUC of 0.71 (95% CI = 0.57–0.84). A CGRP value of 0.84 pg/mL had 88% sensitivity in differentiating migraine patients and HCs. Tear CGRP concentration in five patients before starting anti‐CGRP mAbs (6.9±2.3pg/mL) and T6 (2.4±3.2pg/mL) showed a significant decrease (*p* = 0.026).

**Figure:**
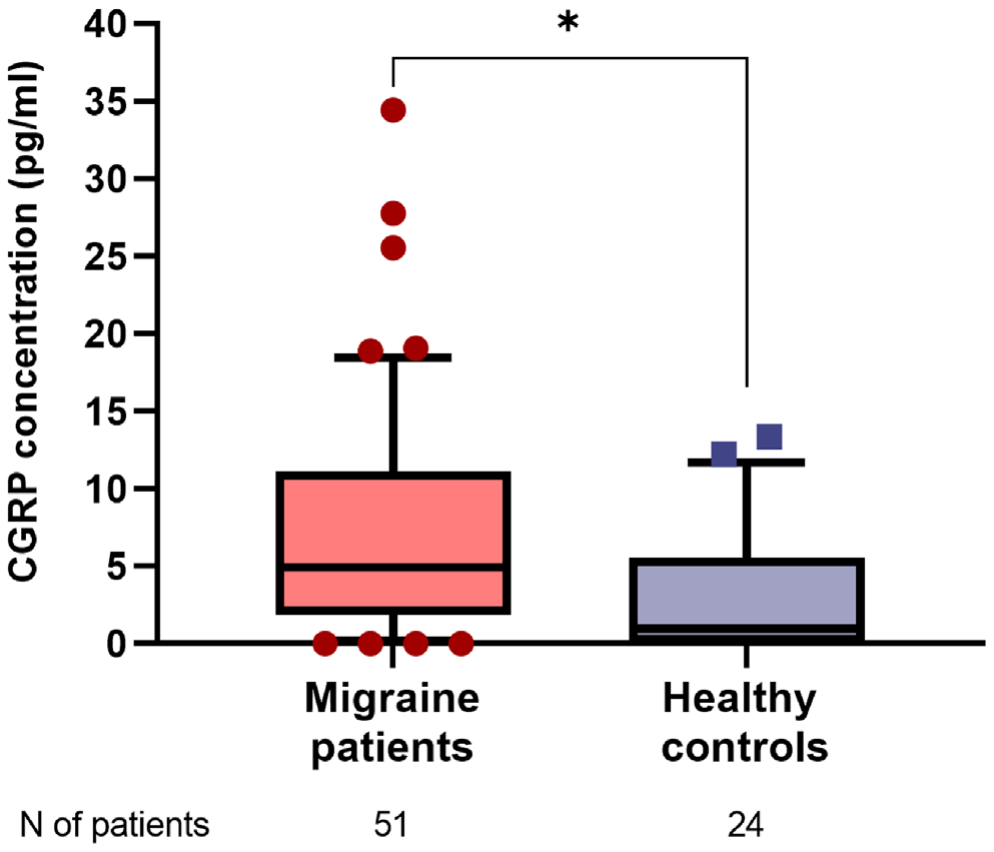
**FIGURE 1** CGRP levels in tear fluid of migraine patients compared to healthy controls. Data are reported in box plots as mean and range 10–90 percentile.

**Figure:**
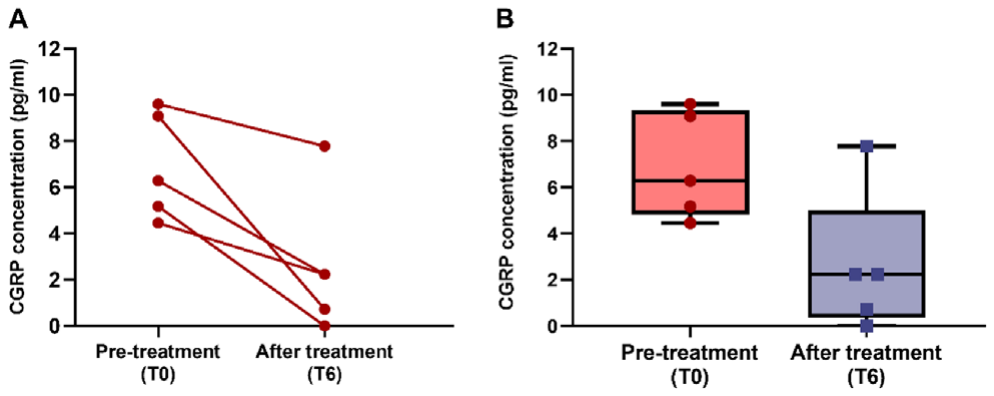
**FIGURE 2** CGRP levels in tear fluid in five migraine patients before starting treatment with anti‐CGRP monoclonal antibodies (baseline) and after 6 months of treatment. Patients‐single data points (A) and box blots (mean with min‐max).

**Conclusion:** Tear fluid CGRP is significantly elevated in migraine patients, particularly in ictal phase and in patients with aura. Measuring CGRP in tears offers a rapid, non‐invasive method with potential utility for diagnosis and monitoring treatment response.

**Disclosure:** Nothing to disclose.

## OPR‐012

### GLP‐1R agonists for the treatment of migraine: A pilot prospective observational study

#### S. Braca; C. Russo; A. Stornaiuolo; G. Cretella; A. Miele; C. Giannini; R. De Simone

##### Department of Neuroscience, Reproductive Sciences and Odontostomatology, University of Naples “Federico II”, Naples, Italy

**Background and Aims:** Migraine affects 14.7% of individuals worldwide and remains difficult to treat. Emerging evidence suggests that even slightly elevated intracranial pressure (ICP) can exacerbate migraine by reducing intracranial compliance and increasing trigeminal pathway sensitization. Glucagon‐like peptide‐1 receptor (GLP‐1R) agonists, which lower cerebrospinal fluid (CSF) production, have shown success in idiopathic intracranial hypertension (IIH) by reducing ICP and improving headache frequency. Therefore, this study investigates GLP‐1R agonists as a potential promising approach to alleviating migraine.

**Methods:** In this pilot prospective observational study, 26 obese migraine patients (BMI ≥30) at our tertiary Headache Centre received subcutaneous Liraglutide 1.2 mg daily for 12 weeks. Papilledema was excluded to rule out IIH. Mean monthly headache days were tracked via standardized diaries. The primary outcome was the change in headache days after 12 weeks; secondary outcomes included BMI reduction, MIDAS score improvement, and adverse events.

**Results:** Mean monthly headache days decreased from 20.04±6.38 to 8.81±6.01 (mean difference 11.23, *p* < 0.001), while MIDAS scores dropped from 62.58 to 27.23 (mean difference 35.35, *p* < 0.001). Although BMI declined from 34.01 to 33.65, this was not significant (*p* = 0.060). Analysis of covariance indicated no influence of BMI reduction on headache frequency (B = 0.190, *p* = 0.949). Mild gastrointestinal adverse events, primarily nausea and constipation, occurred in 10 patients (38%) but did not prompt discontinuation.

**Figure:**
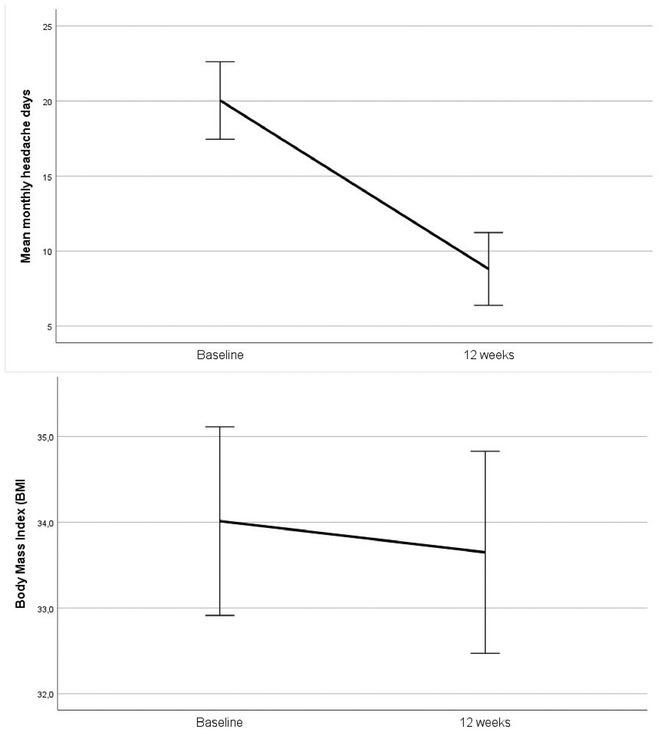
**FIGURE 1** Headache and BMI Reduction

**Conclusion:** GLP‐1R agonists appear to effectively reduce migraine burden regardless of weight loss, highlighting a possible pathophysiological role of CSF volume and pressure regulation in migraine. Further, larger studies are warranted to confirm these findings.

**Disclosure:** Nothing to disclose.

## Ageing and Dementia 1

## OPR‐013

### Abstract withdrawn

## OPR‐014

### Sentence comprehension deficits in Italian and English nfvPPA: A cross‐linguistic perspective

#### E. Canu ^1^; G. Santi^2^; F. Freri^3^; L. Lumaca^3^; Z. Miller^4^; D. Baquirin^4^; V. Castelnovo^1^; S. Cappa^5^; J. De Leon^4^; B. Tee^6^; M. Gorno‐Tempini^6^; M. Filippi^7^; F. Agosta^8^

##### ^1^Neuroimaging Research Unit, Division of Neuroscience, and Neurology Unit, IRCCS San Raffaele Scientific Institute, Milan, Italy; ^2^IRCCS Mondino Foundation, Pavia, Italy; ^3^Neuroimaging Research Unit, Division of Neuroscience, IRCCS San Raffaele Scientific Institute, Milan, Italy; ^4^Memory and Aging Center, University of California San Francisco, San Francisco, California, USA; ^5^Institute for Advanced Studies, IUSS, Pavia, Italy; ^6^Memory and Aging Center, and Global Brain Health Institute, University of California San Francisco, San Francisco, California, USA; ^7^Neurology Unit, Neurorehabilitation Unit, Neurophysiology Service, and Neuroimaging Research Unit, Division of Neuroscience, IRCCS San Raffaele Scientific Institute, and Vita‐Salute San Raffaele University, Milan, Italy; ^8^Neuroimaging Research Unit, Division of Neuroscience, and Neurology Unit, IRCCS San Raffaele Scientific Institute, and Vita‐Salute San Raffaele University, Milan, Italy

**Background and Aims:** There is a growing awareness on the need of cross‐linguistic assessment in Primary Progressive Aphasia (PPA). In particular, differences in language domains, such as morphology, might have an impact on the characterization of patients’ profile. In this work, we aim to compare the performance of English and Italian non‐fluent (nfvPPA) and semantic (svPPA) PPA patients in a sentence comprehension task.

**Methods:** 76 PPA patients (38 English, 28 Italian; 21 svPPA, 17 nfvPPA), matched for age, sex, Mini‐Mental State Examination and Clinical Dementia Rating scale, completed a sentences comprehension task adapted for each language. The task involved 24 oral sentences of different syntactic complexity, and participants had to indicate the matching picture. Composite scores were calculated for High (HSC), Medium (MSC), and Low (LSC) levels of syntactic complexity. Performances were analysed across svPPA and nfvPPA independently from their language, and across languages, using MANCOVA models.

**Results:** NfvPPA were generally more impaired than svPPA in HSC and MSC scores (*p* < 0.045) independent of language. Cross‐linguistically, Italian nfvPPA patients showed lower scores in MSC and LSC compared to English nfvPPA (all *p* < 0.010). No differences were observed between English and Italian svPPA patients (*p* = 0.772).

**Conclusion:** The sentence comprehension task confirmed syntactic processing deficits in nfvPPA patients in both languages. However, the task was more sensitive in identifying syntactic processing deficits in Italian sample, and specifically in nfvPPA patients. This difference is consistent with the known higher morphological complexity of Italian language. This study suggests the importance of tailored language assessment for efficient diagnostic process.

**Disclosure:** Funding. European Research Council (StG‐2016_714388_NeuroTRACK); Foundation Research on Alzheimer Disease. Co‐funding by the Next Generation EU [DM 1557 11.10.2022]. Disclosures. GS, FF, LL, ZM, DPB, JDL, BLT, VC, SFC, MLG have nothing to disclose; EC receive research supports from the Italian Ministry of Health; MF received compensation for consulting services or speaking activities from Alexion, Almirall, Bayer, Biogen, Celgene, Chiesi Italia SpA, Eli Lilly, Genzyme, Janssen, Merck‐Serono, Neopharmed Gentili, Novartis, Novo Nordisk, Roche, Sanofi Takeda, and TEVA; Advisory Boards for Alexion, Biogen, Bristol‐Myers Squibb, Merck, Novartis, Roche, Sanofi, Sanofi‐Aventis, Sanofi‐Genzyme, Takeda; scientific direction of educational events for Biogen, Merck, Roche, Celgene, Bristol‐Myers Squibb, Lilly, Novartis, Sanofi‐Genzyme; he receives research support from Biogen Idec, Merck‐Serono, Novartis, Roche, the Italian Ministry of Health, the Italian Ministry of University and Research, and FISM. FA received speaker honoraria from Biogen Idec, Roche, Eli Lilly, GE Healthcare; receives research supports from IMH, IMUR, AriSLA, ERC, EU JPND Research, Foundation Research on AD (France).

## OPR‐015

### Advancing in vivo diagnosis of limbic‐predominant age‐related TDP‐43 encephalopathy (LATE): A memory clinic cohort study

#### F. Roveta ^1^; F. Pizzini^2^; V. Natale^2^; M. Scheffler^3^; V. Garibotto^4^; A. Mendes^5^; C. Wang^5^; A. Lathuiliere^5^; C. Chicherio^5^; I. Rainero^1^; G. Frisoni^5^; F. Ribaldi^5^

##### ^1^Aging Brain and Memory Clinic, Department of Neuroscience “Rita Levi‐Montalcini”, University of Turin, Torino, Italy; ^2^Radiology, Department of Diagnostic and Public Health, Verona University, Verona, Italy; ^3^Division of Radiology, Geneva University Hospitals, Geneva, Switzerland; ^4^Division of Nuclear Medicine and Molecular Imaging, Diagnostic Department, Geneva University Hospitals, Geneva, Switzerland; ^5^Geneva Memory Center, Department of Rehabilitation and Geriatrics, Geneva University Hospitals, Geneva, Switzerland

**Background and Aims:** The amygdalar atrophy scale (AAS) is an MRI‐based visual rating system for assessing amygdalar atrophy, categorized into AAS0 (no atrophy), AAS1 (mild‐to‐moderate atrophy), and AAS2 (severe atrophy). AAS correlates with amygdala volume, complements the medial temporal atrophy (MTA) scale, and is linked to TDP‐43 pathology, a hallmark of limbic‐predominant age‐related TDP‐43 encephalopathy (LATE‐NC). This study evaluated the utility of AAS in identifying patients with probable LATE‐NC during memory clinic workups.

**Methods:** 1653 subjects who underwent baseline T1‐MRI, clinical, and neuropsychological assessments, were included and classified as cognitively unimpaired (CU), mild cognitive impairment (MCI), or dementia. Using AAS and Alzheimer's disease (AD) biomarkers, individuals were grouped as probable AD neuropathologic changes (ADNC, AAS0 with positive AD biomarkers, *N* = 146), LATE‐NC (AAS1‐2 with negative AD biomarkers, *N* = 36), and AD/LATE‐NC (AAS1‐2 with positive AD biomarkers, *N* = 107). Clinical, neuroimaging, and cognitive trajectories over 30 months were assessed.

**Results:** AAS1‐2 was more frequent in MCI and dementia than CU (*p* < 0.001) and correlated with age (rho = 0.41, *p* < 0.001). LATE‐NC exhibited milder cognitive impairment (MMSE: 26.2) than ADNC (24.2) and AD/LATE‐NC (22.5) (*p* < 0.001). MCI was more prevalent in LATE‐NC (67%) than ADNC (58%) or AD/LATE‐NC (51%), while dementia was less frequent (*p* < 0.001). Reduced volumes and cortical thickness in TDP‐43‐related brain regions were observed in the LATE‐NC and AD/LATE‐NC. LATE‐NC showed slower cognitive decline (*p* = 0.017).

**Conclusion:** Incorporating AAS into memory clinic workups may serve for differentiating suspected ADNC and LATE‐NC cases. Given its reliability and complementary nature to the MTA scale, the AAS offers promise as a diagnostic tool in clinical practice.

**Disclosure:** Nothing to disclose.

## OPR‐016

### Blood‐derived microRNAs associated with hippocampal structure and atrophy rate: Findings from the Rhineland study

#### K. Melas ^1^; V. Talevi^1^; M. Imtiaz^1^; D. Krüger^2^; T. Pena‐Centeno^2^; A. Fischer^2^; A. Aziz^1^; M. Breteler^1^

##### ^1^Population Health Sciences, German Centre for Neurodegenerative Diseases (DZNE), Bonn, Germany; ^2^Department for Epigenetics and Systems Medicine in Neurodegenerative Diseases, German Center for Neurodegenerative Diseases (DZNE), Göttingen, Germany

**Background and Aims:** MicroRNAs are critical for neuronal function and development. Understanding which microRNAs are involved in brain health and neurodegeneration requires determining their relation to key brain regions. Here, we examined the associations of blood‐derived microRNAs with hippocampal structure and atrophy, features related to cognition and dementia, in the general population.

**Methods:** Using data from 2062 participants of the population‐based Rhineland Study, we measured expression of microRNAs and their putative target genes at study baseline in whole blood using RNA sequencing. Hippocampal and total brain volumes were measured using 3T MRI at baseline and follow‐up (4.6 to 8.0 years later). We examined microRNA associations with left and right hippocampal volume, hippocampal asymmetry, and total brain volume cross‐sectionally using linear regression, and longitudinally using mixed‐effects models. Moreover, we identified genomic loci influencing microRNA expression and used them for two‐sample Mendelian Randomization analysis.

**Results:** Cross‐sectionally, five microRNAs were associated exclusively with left hippocampal volume. Longitudinally, another six microRNAs were associated with left hippocampal, right hippocampal, and total brain atrophy rates. Nineteen microRNAs were exclusively associated with total brain atrophy rate. Several identified microRNAs regulate target genes involved in brain development, memory, axon guidance, and synapse assembly. Mendelian Randomization suggested that larger hippocampal volume causes lower expression of one microRNA.

**Conclusion:** We identified microRNA signatures that were ‐ partly side‐specifically ‐ related to hippocampal structure and atrophy, suggesting different influences of microRNAs during brain development and aging. Some identified microRNAs have been previously linked to dementia and may be useful as presymptomatic blood‐based biomarkers of neurodegeneration.

**Disclosure:** Nothing to disclose.

## OPR‐017

### Creutzfeldt‐Jakob disease in Northern Portugal: Retrospective study of clinical characteristics over 10 years

#### L. Costa^1^; S. Lopes ^2^; J. Pinto^2^; A. Marques^3^; R. Cagigal^4^; I. Carvalho^5^; M. Seco^6^; L. Barbosa^7^; A. Fernandes^8^; D. Oliveira^9^; S. Costa^10^; S. Rocha^2^

##### ^1^Neurology Department, Local Health Unit of Alto Minho, Alto Minho, Portugal; ^2^Neurology Department, Local Health Unit of Braga, Braga, Portugal; ^3^Neurology Department, Local Health Unit of Trás‐os‐Montes and Alto Douro, Vila Real, Portugal; ^4^Neurology Department, Local Health Unit of Gaia and Espinho, Vila Nova de Gaia, Portugal; ^5^Neurology Department, Local Health Unit of Alto Ave, Guimarães, Portugal; ^6^Neurology Department, Local Health Unit of Matosinhos, Matosinhos, Portugal; ^7^Neurology Department, Local Health Unit of Tâmega and Sousa, Penafiel, Portugal; ^8^Neurology Department, Local Health Unit of São João, Porto, Portugal; ^9^Neurology Department, Local Health Unit of Entre Douro and Vouga, Santa Maria da Feira, Portugal ^10^Neurology Department, Local Health Unit of Santo António, Porto, Portugal

**Background and Aims:** Creutzfeldt‐Jakob disease (CJD) is the most common prion disease, most often in its sporadic type. It manifests as a combination of rapidly progressive dementia and other neurological signs such as myoclonus or ataxia. It is invariably fatal and is associated with significant demographic and clinical impact.

**Methods:** Retrospective multicentre study that included cases diagnosed with probable CJD in 10 centres in northern Portugal since 2011. Statistical analysis of demographic, clinical characteristics and ancillary testing.

**Results:** We included 61 patients diagnosed with probable sporadic CJD (30 men). Median age was 69 years (44–88). Half the patients were diagnosed 2 (23 days ‐ 24) months after symptom onset. The main presentation was dementia (95.1%), myoclonus (75.4%), extra‐pyramidal signs (59%) and ataxia (55.7%). Neuropsychiatric symptoms coexisted in 68.9%, most frequently apathy and depression. EEG showed periodic activity in 63.3% of cases; MRI showed T2/FLAIR hyperintensity and/or cortical restricted diffusion in 85.2% and/or of the basal nuclei in 68.9%. Protein 14‐3‐3 in CSF was positive in 96.5% of cases. Three patients had mutations in the PRNP gene. Median survival after diagnosis was 3 months (1–43), with 22 patients undergoing autopsy with diagnostic confirmation. Analysis using Mann‐Whitney test identified extrapyramidal signs associated with lower survival after diagnosis, with statistical significance (U = 262; *p* = 0.019).

**Conclusion:** We aimed to illustrate clinical heterogeneity of CJD, impacting timely diagnosis, clinical approach and survival.

**Disclosure:** Nothing to disclose.

## Cerebrovascular Diseases

## OPR‐018

### Tenecteplase in central retinal artery occlusion study

#### A. Aamodt ^1^; S. Ryan^1^; Ø. Jørstad^2^; M. Skjelland^1^; C. Simonsen^3^; P. Ijäs^4^; T. Bek^5^; D. Strbian^4^; Khanevski^6^; R. Lemmens^7^; I. Nakstad^8^; L. Christensen^9^; H. Ellekjær^10^; V. Matijošaitis^11^; T. Truelsen^12^; E. Rødahl^13^; J. Krohn^13^; M. Mazya^14^; K. Kraglund^1^; H. Lisether^15^; K. Devik^16^; V. Malmberg^17^; J. Valaikiene^18^; P. Ylikotila^19^; M. Moe^2^

##### ^1^Dept of Neurology, Oslo University Hospital Oslo, Norway; ^2^Dept of Ophthalmology, Oslo University Hospital Oslo, Norway; ^3^Dept of Neurology, Aarhus University Hospital, Aarhus, Denmark; ^4^Dept of Neurology, Helsinki University Hospital, Helsinki, Finland; ^5^Dept of Ophthalmology, Aarhus University Hospital, Aarhus, Denmark; ^6^Dept of Neurology, Haukeland University Hospital Bergen, Norway; ^7^Dept of Neurology, Leuven University Hospital Leuven, Belgium; ^8^Dept of Neurology, Vestre Viken Hospital Trust, Drammen, Norway; ^9^Dept of Neurology, Bispebjerg University Hospital, Copenhagen, Denmark; ^10^Stroke Unit, St. Olav Hospital, Trondheim, Norway; ^11^Department of Neurology, Kaunas University Hospital, Kaunas, Lithuania; ^12^Dept of Neurology, Rigshospitalet Copenhagen University Hospital, Copenhagen, Denmark; ^13^Dept of Ophthalmology, Haukeland University Hospital Bergen, Norway; ^14^Dept of Neurology, Karolinska University Hospital, Stockholm, Sweden; ^15^Dept of Neurology, Tønsberg Hospital, Tønsberg, Norway; ^16^Dept of Neurology, Namsos Hospital, Namsos, Norway; ^17^Dept of Neurology, Telemark Hospital, Skien, Norway; ^18^Vilnius University Center of Neurology, Vilnius University Hospital Santaros klinikos, Vilnius Lithuania; ^19^Division of Clinical Neurosciences Department of Cerebrovascular Disorders Turku University Hospital Turku, Finland

**Background and Aims:** Central retinal artery occlusion (CRAO) is an ophthalmologic emergency that, without prompt reperfusion, bears high risk of permanent blindness. No evidence‐based treatment is currently available. Whether prompt reperfusion with thrombolytic agents can improve the outcome in CRAO, as proved in ischemic stroke, remains unanswered. The main aim is to assess the effect of systemic tenecteplase within 4.5 hours of onset of central retinal artery occlusion

**Methods:** The trial is an ongoing prospective, randomised‐controlled, double‐dummy, double‐blind phase 3 multi‐centre trial of TNK 0.25 mg/kg + placebo vs. ASA + placebo (2 arms with 1:1 block randomisation). Patients are recruited after an ophthalmologist has confirmed CRAO and they can be treated within 4.5 hrs. After observation in the stroke unit, patients are re‐examined by an ophthalmologist and a neurologist as an out‐patient at 30 and 90‐day follow‐up. The primary outcome is the proportion of patients with ≤ 0.7 logMAR best‐corrected visual acuity (BCVA) corresponding to decimal best‐corrected visual acuity ≥ 0.2 at *p* in the affected eye at 30 days after treatment, representing an improvement in BCVA of at least 0.3 logMAR.

**Figure:**
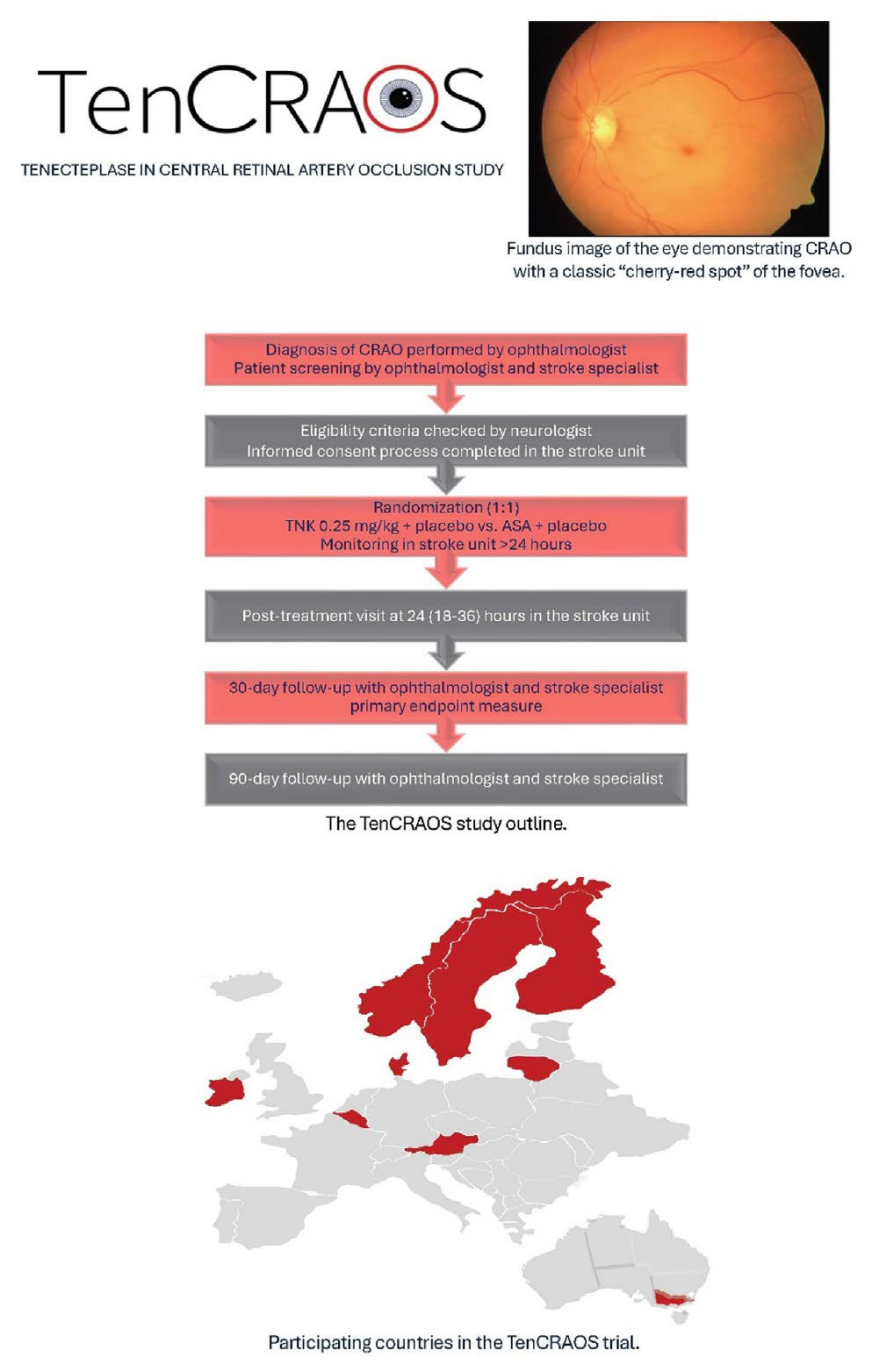
**FIGURE 1** Study outline, fundus image of the eye demonstrating CRAO and map with participating countries.

**Results:** 8 countries are participating with 29 centres activated during the course of the study. Currently there are 7 countries activated for recruitment. We have recruited 76 of 78 patients so far, 32 in Norway, 19 in Denmark, 11 in Finland, 7 in Belgium, 4 in Lithuania and 3 in Sweden. All patients have been included within the strict parameters of the study.

**Conclusion:** The main results will be presented at the conference.

**Disclosure:** Boehringer‐Ingelheim provided tenecteplase and intravenous placebo free of charge as well as financial support to the sites outside Norway but had no influence on the study conduct, analysis, or interpretation.

## OPR‐019

### Enhancing moyamoya diagnosis and care: A multi‐disciplinary study on Norway's first moyamoya cohort

#### A. Aamodt ^1^; J. Sømark^1^; M. Skjelland^1^; T. Nordenmark^2^; A. Sorteberg^3^; M. Wiedmann^3^

##### ^1^Dept. of Neurology, Oslo University Hospital, Oslo, Norway; ^2^Neuropsychological Section Department of Physical Medicine and Rehabilitation, Oslo University Hospital, Oslo, Norway; ^3^Dept. of Neurosurgery, Oslo University Hospital, Oslo, Norway

**Background and Aims:** Moyamoya angiopathy (MMA) is a rare and progressive cerebrovascular disorder of uncertain etiology, predominantly affects young women posing a high risk of stroke and disability without optimal care. To address these challenges, we established a dedicated multi‐disciplinary moyamoya task force and started a quality improvement project in 2021. The aim of this project was to evaluate the project.

**Methods:** Strong multidisciplinary collaboration, common moyamoya clinic, standardizing of imaging including H2[15O]‐PET, quality registry and international collaboration were key elements in the development of common pathway (Figure 1). Information portal and courses were established with extensive user involvement to strengthen health literacy. Dissemination was performed through podcasts, blog‐interviews and lectures. Functional outcomes and patient reported outcome measures were collected. Additionally, yearly interviews of patients, next‐of‐kin and health care providers were performed.

**Figure:**
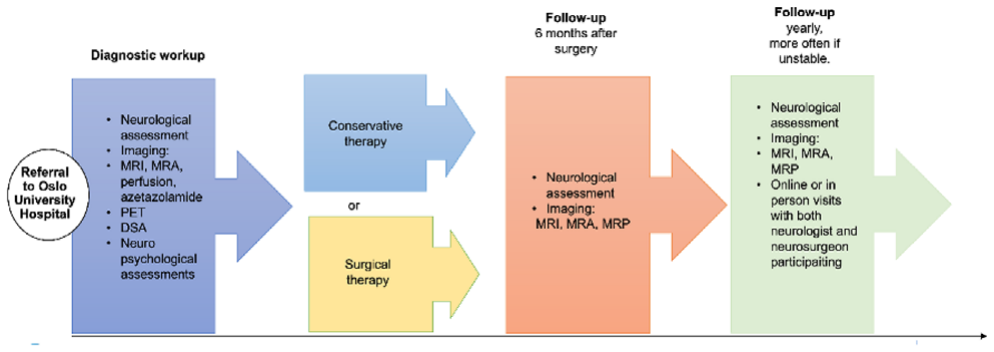
**FIGURE 1** Moyamoya pathway

**Results:** A quality registry encompassing 100 MMA patients (1/2 treated with revascularization surgery) was established. In the present substudy, 56 patients were included: 89.2% moyamoya disease, 10.8% moyamoya syndrome. Mean age 47.2 (SD 11.46) years, 78.9% women, mean mRS 1.7, EQ‐5D‐5L VAS distribution 61.8 of 100 (SD 21.3), mean total mental fatigue score 18.0 (>10 indicates mental fatigue). The participants reported the project to have considerable impact on several aspects and how to cope with the situation (Figure 2).

**Figure:**
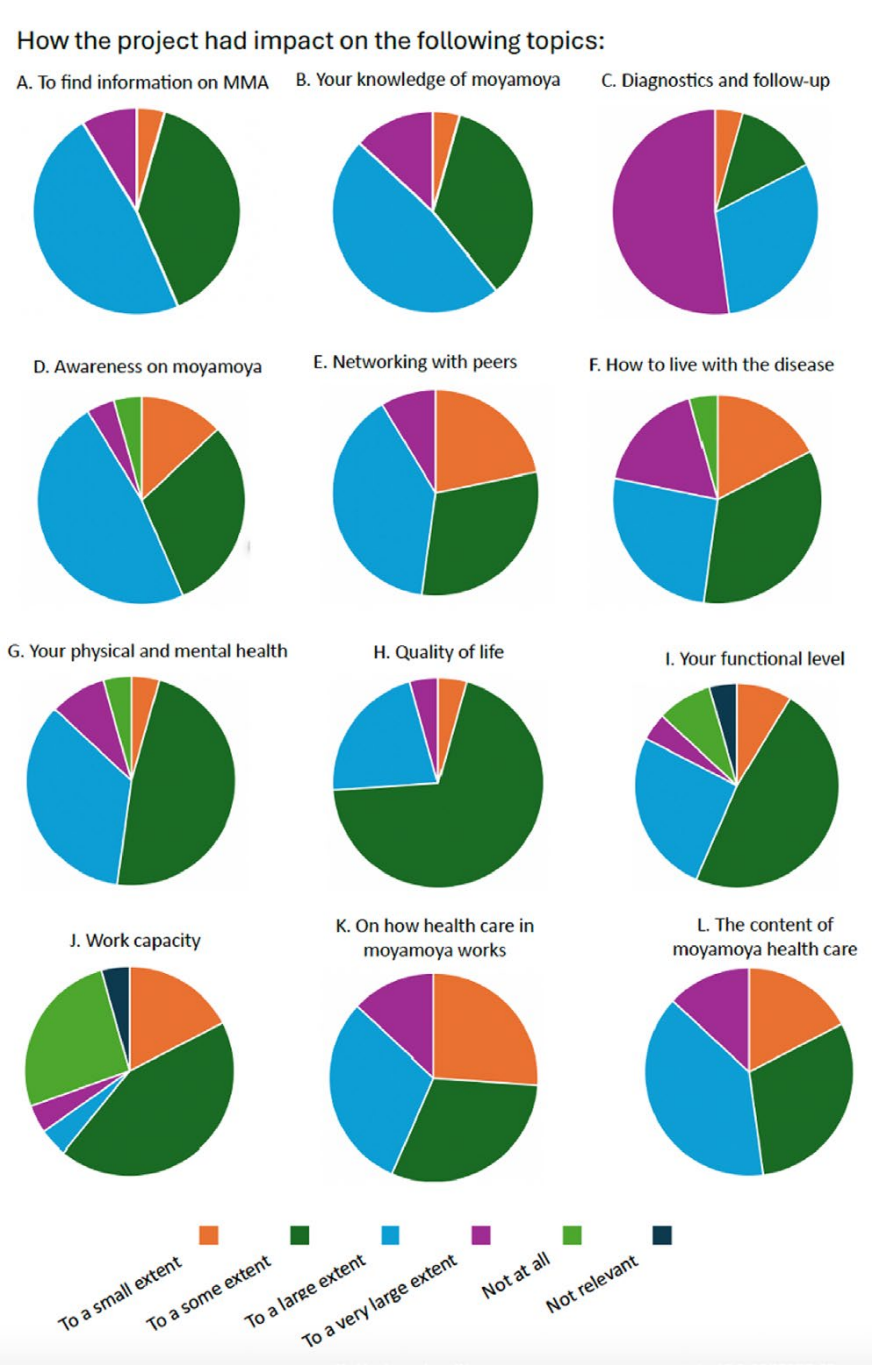
**FIGURE 2** Results of the quality improvement project ‐ evaluation from patients, next‐of‐kin and health‐care providers.

**Conclusion:** The implemented MMA model with strong user involvement was feasible and resulted in improved quality of care.

**Disclosure:** None

## OPR‐020

### Abstract withdrawn

## OPR‐021

### Clinical impact of endovascular treatment implementation in primary stroke centers of catalonia

#### J. Mayol ^1^; Á. García‐Tornel^2^; M. Rodrigo‐Gisbert^2^; F. Rizzo^2^; M. Olivé‐Gadea^2^; M. Requena^2^; M. Terceño^3^; Y. Silva^3^; X. Ustrell^4^; A. Flores^4^; F. Purroy^5^; G. Mauri‐Capdevila^5^; S. Abilleira^6^; M. Rubiera^2^; M. Ribó^2^

##### ^1^Neurology Department, Hospital Universitari Vall d'Hebron, Barcelona, Spain; ^2^Stroke Unit, Hospital Universitari Vall d'Hebron, Barcelona, Spain; ^3^Stroke Unit, Hospital Universitari Doctor Josep Trueta, Girona, Spain; ^4^Stroke Unit, Hospital Universitari Joan XXIII, Tarragona, Spain; ^5^Stroke Unit, Hospital Universitari Arnau de Vilanova, Lleida, Spain; ^6^10Stroke Programme, Catalan Health Department, Barcelona, Spain

**Background and Aims:** We aim to evaluate whether the implementation of endovascular thrombectomy (EVT) capabilities in areas covered by primary stroke centers (PSC) improves outcomes in patients with ischemic stroke associated with anterior circulation vessel occlusion.

**Methods:** We analyzed registry data to identify ischemic stroke patients with anterior circulation vessel occlusions in catchment areas of 3 PSC that transitioned to thrombectomy‐capable centers (TCCs, 1 full time, 2 during working hours). The study compared the proportion of patients treated with EVT, complete reperfusion(mTICI2C‐3), symptomatic intracranial hemorrhage(sICH) and time from first hospital admission to puncture (ATP) before (January 2017–June 2020) and after (July 2020–December 2023) the implementation was fully active (COVID‐19 pandemic).

**Results:** Of the 1,467 patients included, 859 (59%) were evaluated after the implementation. The proportion of patients treated with EVT increased from 406/608(67%) before the implementation to 667/859(77%) after the implementation (OR 1.78; 95% CI 1.369–2.182), with a decrease in the time from first ATP times of 25 minutes (95% CI 3.370–46.443). Among treated patients, no significant differences were observed in rates of complete reperfusion (222[55.4%] vs 363[54.4%]; OR 0.861; 95% CI 0.668–1.110) or sICH (21[5.2%] vs 51[7.6%]; OR 1.518; 95% CI 0.899–2.563).

**Figure:**
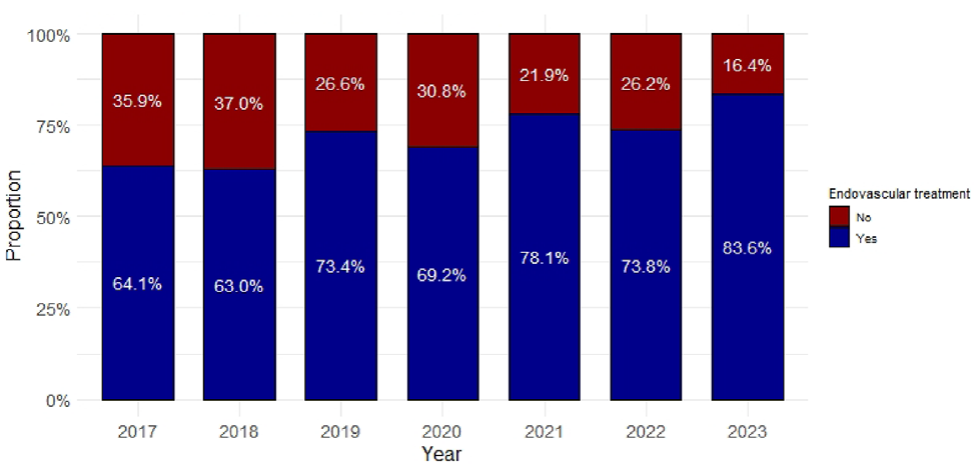
**FIGURE 1** Proportion of vessel occlusions treated with thrombectomy

**Figure:**
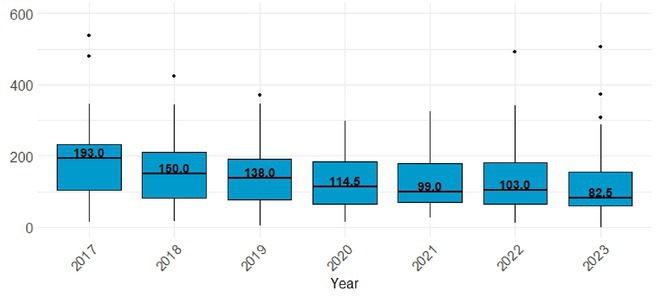
**FIGURE 2** Time from first hospital admission to arterial puncture (minutes)

**Conclusion:** Integration of EVT capabilities into PSCs has significantly enhanced access to EVT in densely populated areas by increasing treatment rates and reducing ATP times. These operational advancements have been achieved without compromising reperfusion outcomes or safety standards. Further expansion of TCCs could improve both access and timeliness of EVT, helping to address geographic disparities and improving quality of stroke care for patients.

**Disclosure:** Nothing to disclose.

## OPR‐022

### Timing of anticoagulation following decompressive surgery for cerebral vein and sinus thrombosis: An observational study

#### M. C. Taveira ^1^; S. Aaron^2^; J. M. Ferreira^3^; J. M. Coutinho^4^; P. Canhão^1^; A. Conforto^5^; A. Arauz^6^; M. Carvalho^7^; J. Masjuan^8^; V. K. Sharma^9^; J. Putaala^10^; M. Uyttenboogaart^11^; D. J. Werring^12^; R. Bazan^13^; S. Mohindra^14^; J. Weber^15^; B. A. Coert^16^; P. Kirubakaran^2^; M. Sanchez van Kammen^4^; P. Singh^2^; D. Aguiar de Sousa^1^; J. M. Ferro^1^

##### ^1^Centro de Estudos Egas Moniz, Faculdade de Medicina, Universidade de Lisboa, Portugal; ^2^Neurology Unit, Department of Neurological Sciences, Christian Medical College & Hospital, Vellore, Tamil Nadu, India; ^3^Serviço de Neurologia, ULS São José, Lisboa, Portugal; ^4^Department of Neurology, Amsterdam University Medical Centers, Amsterdam, The Netherlands; ^5^Hospital das Clínicas, Universidade de São Paulo, São Paulo, Brazil; ^6^Stroke Clinic, Instituto Nacional de Neurología y Neurocirugía Manuel Velasco Suárez, Mexico City, Mexico; ^7^Serviço de Neurologia, Unidade Local de Saúde São João; Departamento de Neurociências Clínicas e Saúde Mental, Faculdade de Medicina da Universidade do Porto, Porto, Portugal; ^8^Servicio de Neurología, Hospital Universitario Ramón y Cajal, IRYCIS, Departamento de Medicina, Universidad de Alcalá. Red RICORS, Madrid, Spain; ^9^Department of Medicine, Yong Loo Lin School of Medicine, National University of Singapore, Singapore; ^10^Department of Neurology, Helsinki University Hospital and University of Helsinki, Helsinki, Finland; ^11^Department of Neurology and Medical Imaging Center University Medical Center Groningen, University of Groningen, Groningen, The Netherlands; ^12^Stroke Research Centre, UCL Queen Square Institute of Neurology, London, UK; ^13^Faculdade de Medicina Campus de Botucatu, Universidade Estadual Paulista Julio de Mesquita Filho, Botucatu, São Paulo, Brazil; ^14^Department of Neurosurgery, Post Graduate Institute of Medical Education & Research (PGIMER), Chandigarh, India; ^15^Department of Neurosurgery, Steinenberg Clinic, Reutlingen, Germany; ^16^Department of Neurosurgery, Amsterdam University Medical Centers, University of Amsterdam, Amsterdam, The Netherlands

**Background and Aims:** Anticoagulation is the mainstay therapy for acute cerebral venous thrombosis (CVT). Decompressive surgery is necessary in patients with large parenchymal lesions and impending herniation, requiring a temporary suspension of anticoagulation. This study sought to identify the optimal timing for initiating/resuming anticoagulation following decompressive surgery.

**Methods:** Data were collected from the Decompressive Surgery for CVT Study 2 (DECOMPRESS2), a multinational cohort study of 118 patients with CVT treated by decompressive surgery. We assessed the frequency of new hemorrhagic and venous thrombotic events in patients who started/resumed anticoagulation <24h and > = 24 following surgery. Death and disability were evaluated by the modified Rankin scale (mRS >2) at discharge and at one year follow up.

**Results:** Of the 90 patients available for analysis, 35 (39%) started/resumed anticoagulation within 24 hours following surgery while 55 (61%) did so later than 24 hours. Overall frequency of patients with new hemorrhagic or venous thrombotic events was 26.7% (24 patients). Distribution of major hemorrhagic events was 8 (23%) bleedings in the <24‐hour group, and 9 (16%) in the > = 24h. No CVT recurred. Two venous thrombotic events occurred in <24h (6%) and 5 in the > = 24h (9%) group. Timing of anticoagulation was not associated with death or disability at discharge (OR 1.65. 95% CI 0.30 to 9.01, *p* = 0.56), or one year follow up (OR 2.19, 95% CI 0.78 to 6.10, *p* = 0.14).

**Conclusion:** The results suggest that timing of anticoagulation therapy following decompressive surgery does not significantly influence the risk of new bleeding or venous thrombotic events or disability.

**Disclosure:** Nothing to disclose.

Neuropathies

## OPR‐023

### Chat‐GPT‐4o in diagnosis and management of real‐ life polyneuropathy cases: Comparative analysis with neurologists

#### A. De Lorenzo ^1^; G. Moretti^2^; A. Bertini^3^; A. De Lorenzo^2^; R. Collet‐Vidiella^4^; A. Fasolino^5^; E. Fortanier^6^; R. Hadden^7^; B. Islam^8^; K. Kleopa^9^; L. Leon Cejas^10^; L. Leonardi^11^; V. Mira^12^; S. Peric^13^; Y. Rajabally^14^; T. Sevilla^15^; P. Tomaselli^16^; S. Tozza^17^; C. Pisciotta^2^; D. Pareyson^2^; E. Nobile‐Orazio^18^; P. Doneddu^19^

##### ^1^Neuromuscular and Neuroimmunology Unit, IRCCS Humanitas Research Hospital, University of Milan, Milan, Italy; ^2^Neuromuscular and Neuroimmunology Unit, IRCCS Humanitas Research Hospital, 20089 Rozzano, Milan, Italy; ^3^Fondazione IRCCS Istituto Neurologico Carlo Besta, Milan, Italy + University of Milan, Milan, Italy; ^4^Neuromuscular Diseases Unit, Department of Neurology, Hospital de La Santa Creu I Sant Pau, Universitat Autonoma de Barcelona; Biomedical Research Institute Sant Pau, 08041, Barcelona, Spain; ^5^UOC Neurophysiopathology, AORN Cardarelli, Via Antonio Cardarelli 9, Naples, 80131, Italy; ^6^Referral Centre for Neuromuscular Diseases and ALS, Hospital La Timone, Aix‐Marseille University, Marseille, France; ^7^Neurology Department, King's College Hospital, London, UK. + Department of Basic & Clinical Neuroscience, Institute of Psychiatry, Psychology & Neuroscience, King's College London, London, UK; ^8^Department of Neurology and Neurophysiology, BRB Hospital, Dhaka, Bangladesh; ^9^Neuroscience Department, The Cyprus Institute of Neurology and Genetics and Center for Neuromuscular Diseases, The Cyprus Institute of Neurology and Genetics, Nicosia, Cyprus; ^10^Department of Neurology, Hospital Británico de Buenos Aires, Argentina; ^11^Neuromuscular and Rare Disease Centre, Neurology Unit, Sant’Andrea Hospital, Rome, Italy; ^12^AP‐HM, Timone Hospital, Department of Neurology and Pathology Of Movement, Marseille, France; ^13^University Clinical Centre of Serbia – Neurology Clinic, University of Belgrade – Faculty of Medicine, Belgrade, Serbia; ^14^Aston Medical School, Aston University, Birmingham, UK; ^15^Neurology Department, Hospital Universitari i Politècnic La Fe & IIS La Fe, Valencia, Spain and Centro de Investigación Biomédica en Red de Enfermedades Raras (CIBERER), Madrid, Spain and Neurology Department, Universitat de València, Valencia, Spain; ^16^Department of Neurosciences and Behaviour Sciences, Neuromuscular Disorders, University of São Paulo, Ribeirao Preto 14040‐900, Brazil; ^17^Department of Neuroscience and Reproductive and Odontostomatological Sciences, University Federico II of Naples, Via S Pansini 5, 80131, Naples, Italy; ^18^Neuromuscular and Neuroimmunology Unit, IRCCS Humanitas Research Hospital, 20089 Rozzano, Milan, Italy and Department of Medical Biotechnology and Translational Medicine, Milan University, 20133, Milano, Italy; ^19^Neuromuscular and Neuroimmunology Unit, IRCCS Humanitas Research Hospital, 20089 Rozzano, Milan, Italy and Department of Biomedical Sciences, Humanitas University, 20072 Pieve Emanuele, Milan, Italy.

**Background and Aims:** Accurate diagnosis and management of polyneuropathies remain challenging, particularly for non‐specialist neurologists. Generative Pre‐trained Transformer (GPT) models show potential to enhance diagnostic accuracy despite their general‐purpose design. This study evaluated GPT‐4o's performance in diagnosing polyneuropathies and guiding confirmatory testing compared to specialist and non‐specialist neurologists.

**Methods:** Data from 100 confirmed polyneuropathy cases were collected from tertiary care centers. Cases were presented to GPT‐4o using a zero‐shot chain‐of‐thought prompt to generate a leading diagnosis, two differentials, and a confirmatory test. Diagnoses and tests from 26 neurologists (14 specialists, 12 non‐specialists) across 19 centers in 10 countries were collected before and after reviewing GPT‐4o's output. Accuracy was compared using paired t‐tests, and inter‐output reliability was assessed with Cohen's kappa.

**Figure:**
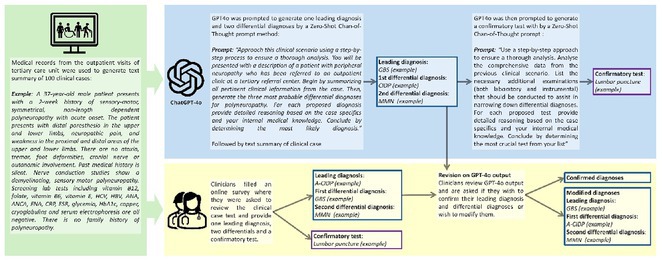
**FIGURE 1** Flowchart representation of materials and methods

**Results:** GPT‐4o demonstrated high inter‐output consistency (kappa = 0.8, *p* < 0.001) and outperformed non‐specialists in leading diagnosis accuracy (65.5% vs 54.4%, *p* = 0.007) but it was inferior to specialists (73.9%, *p* = 0.024). Including differential diagnoses, GPT‐4o outperformed non‐specialists (82.0% vs. 68.5%, *p* < 0.001) but remained below specialists (88.1%, *p* = 0.042). Non‐specialists improved their accuracy after reviewing GPT‐4o's suggestions (54.4% to 57.0%, *p* = 0.007), whereas specialists showed a non‐significant increase (73.9% to 75.0%, *p* = 0.069). GPT‐4o errors included over‐reliance on laboratory findings or past history (38%), overlooking clinical information (16%), vague conclusions (16%), limited internal knowledge (9%), and reasonable but incorrect responses (22%). GPT‐4o matched experts in recommending diagnostic tests (68.0% vs 67.3%, *p* = 0.874) and surpassed non‐specialists (45.3%, *p* < 0.001).

**Conclusion:** GPT‐4o shows promise as diagnostic support tool, improving non‐specialists’ accuracy and guiding confirmatory testing. Its supervised integration could help bridge expertise gaps in neurological care.

**Disclosure:** Nothing to disclose.

## OPR‐024

### Plasma lipidomic patterns associated with disease activity in chronic inflammatory demyelinating polyradiculoneuropathy

#### D. Fitzner; K. auf dem Brinke; J. Lattau; L. Borsch; J. Zschüntzsch

##### Department of Neurology, University Medical Center Göttingen

**Background and Aims:** Chronic inflammatory demyelinating polyneuropathy (CIDP) is an immune‐mediated neuropathy that leads to significant disability and substantial healthcare costs. Although the exact pathogenic mechanisms remain unclear, it is known that inflammation results in segmental demyelination, accompanied by the release of myelin lipids into the extracellular space. This study aims to investigate the plasma lipidomic profile of CIDP patients to identify lipid patterns associated with disease activity.

**Methods:** We employed high‐throughput shotgun lipidomics to analyze and compare the plasma lipidome of 30 patients with CIDP (mean age ± SD: 60.7 ± 12.2 years) with that of 30 individuals diagnosed with non‐demyelinating neurological disorders (OND; mean age ± SD: 52.8 ± 10.3 years). Lipid classes and subspecies were quantified in absolute [pmol] and relative concentrations [mol%], and their correlation with CIDP disease activity and clinical disability scores (R‐ODS, INCAT and MRC) was assessed. To control confounders such as age and weight, strongly correlated lipids were excluded.

**Results:** The analysis identified 669 molecular lipid species across 15 lipid classes; with a significant elevation in the diacylglycerol (DAG) class in CIDP patients. Furthermore, specific lipid subspecies, including triacylglycerol (TAG), DAG, and ether‐linked phosphatidylcholine (PC O), were significantly correlated with disease activity. A distinct lipid subspecies set including phosphatidylcholine (PC), lyso‐phosphatidylcholine (LPC), phosphatidylinositol (PI), sphingomyelin (SM), and cholesterol ester (CE) showed strong associations with clinical disability scores.

**Figure:**
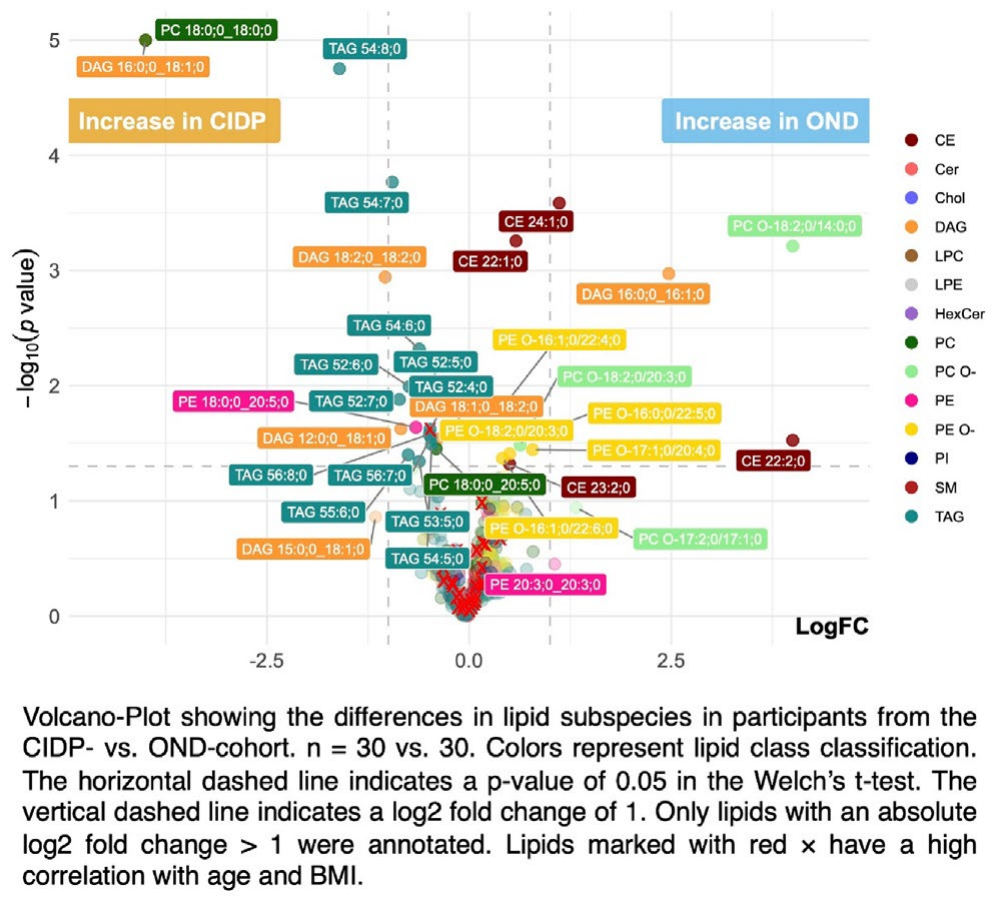
**FIGURE 1** Differences of lipid subspecies in control vs. CIDP patients

**Conclusion:** These findings indicate that CIDP is characterized by distinct lipidomic profiles, offering potential lipid biomarkers for disease activity and severity. Such biomarkers could enhance diagnostic precision and inform clinical management.

**Disclosure:** We declare no conflict of interest.

## OPR‐025

### Elevated serum concentrations of GFAP in hereditary transthyretin amyloidosis since pre‐symptomatic stages

#### D. Plantone ^1^; M. Luigetti^2^; C. Manco^1^; A. Romano^2^; L. Leonardi^3^; V. Guglielmino^4^; F. Forcina^5^; M. Ceccanti^6^; M. Inghilleri^6^; F. Manganelli^7^; S. Tozza^7^; M. Sciarrone^4^; F. Vitali^4^; A. Sabino^4^; D. Righi^1^; A. Stufano^8^; M. Stromillo^1^; N. De Stefano^1^; P. Calabresi^4^; G. Primiano^2^

##### ^1^Dept. of Medicine, Surgery and Neuroscience, University of Siena; ^2^Dipartimento di Neuroscienze, Organi di Senso e Torace, Fondazione Policlinico Universitario Agostino Gemelli IRCCS, Rome, Italy; ^3^Neuromuscular and Rare Disease Centre, Neurology Unit, Sant'Andrea Hospital, Rome, Italy; ^4^Dipartimento di Neuroscienze, Università Cattolica del Sacro Cuore, Rome, Italy; ^5^Dipartimento di Neuroscienze, Salute Mentale e Organi di Senso (NESMOS), Sapienza Università di Roma, Rome, Italy; ^6^Dipartimento di Neuroscienze Umane, Sapienza Università di Roma, Rome, Italy; ^7^Department of Neuroscience, Reproductive and Odontostomatological Science, University of Naples 'Federico II', Naples, Italy; ^8^Interdisciplinary Department of Medicine, University of Bari Aldo Moro, Bari, Italy

**Background and Aims:** Hereditary transthyretin amyloidosis (ATTRv) is a rare disorder caused by pathogenic TTR gene variants. Glial fibrillary acidic protein (GFAP) and neurofilament light chain (NfL) are potential biomarkers for astrocyte activation and neuroaxonal damage, respectively. This study investigates serum GFAP (sGFAP) and NfL (sNfL) levels in ATTRv patients, pre‐symptomatic subjects, and healthy controls (HCs) to evaluate their utility as biomarkers of disease progression and CNS involvement.

**Methods:** Our multicenter cross‐sectional study included 111 ATTRv patients (56 symptomatic, 55 pre‐symptomatic subjects) and 183 HCs. Serum levels of sGFAP and sNfL were measured using ultrasensitive immunoassays. Statistical comparisons were performed using ANCOVA models (age and sex adjusted), with correlations examined between serum biomarkers and disease severity (Neuropathy Impairment Score, NIS).

**Results:** sGFAP levels were elevated in symptomatic (median: 238.35 pg/ml) and pre‐symptomatic subjects (median: 105.50 pg/ml) vs. HCs (median: 75.5 pg/ml, *p* < 0.001). sNfL was elevated only in symptomatic patients (median: 43.68 pg/ml) compared to pre‐symptomatic subjects (median: 9.36 pg/ml) and HCs (median: 7.54 pg/ml, *p* < 0.001). Both biomarkers correlated significantly with NIS, reflecting disease severity. Female HCs had higher sGFAP levels than males (median 88.6 pg/ml vs. 59.8 pg/ml; *p* 0.011).

**Figure:**
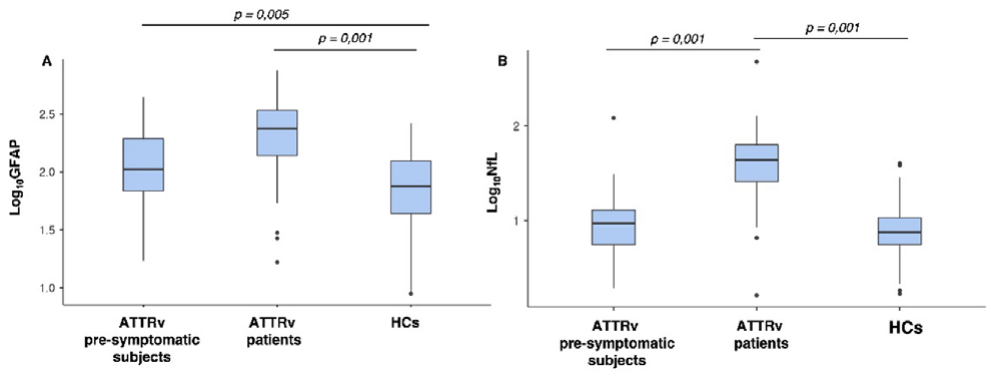
**FIGURE 1** Comparison of sGFAP and sNfL levels (expressed as Log10‐GFAP and Log10‐NfL, respectively) across the three groups. (A) sGFAP levels were significantly higher in both ATTRv patients and pre‐symptomatic subjects compared to healthy controls (HCs).

**Figure:**
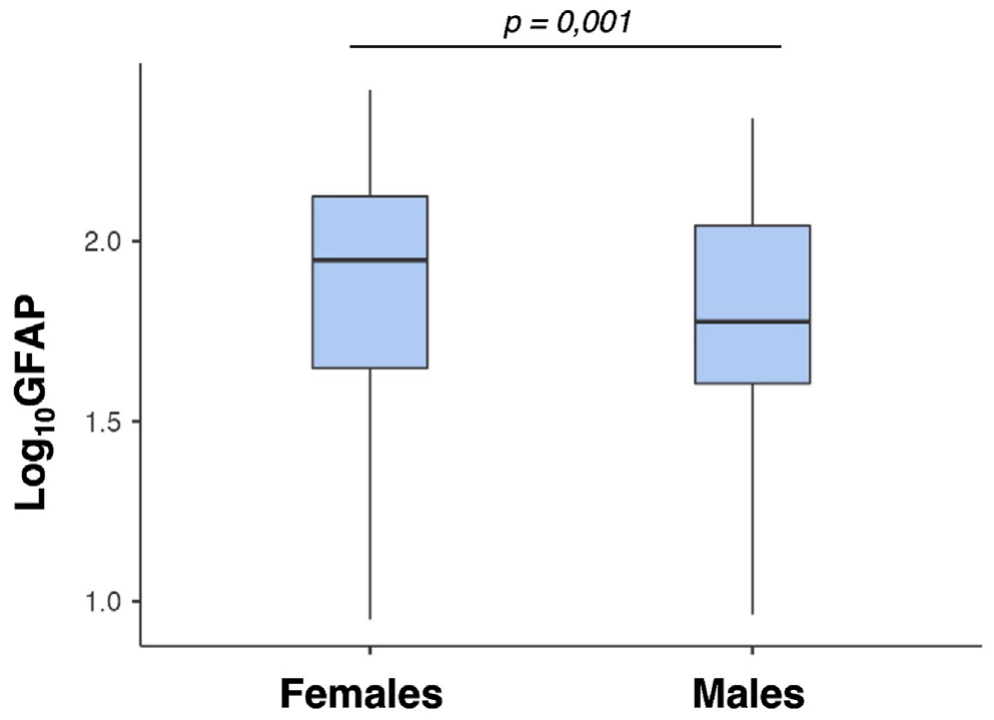
**FIGURE 2** Comparison of sGFAP levels (expressed as Log10‐GFAP) between females and males in the HCs cohort, demonstrating significantly higher sGFAP values in females compared to males.

**Figure:**
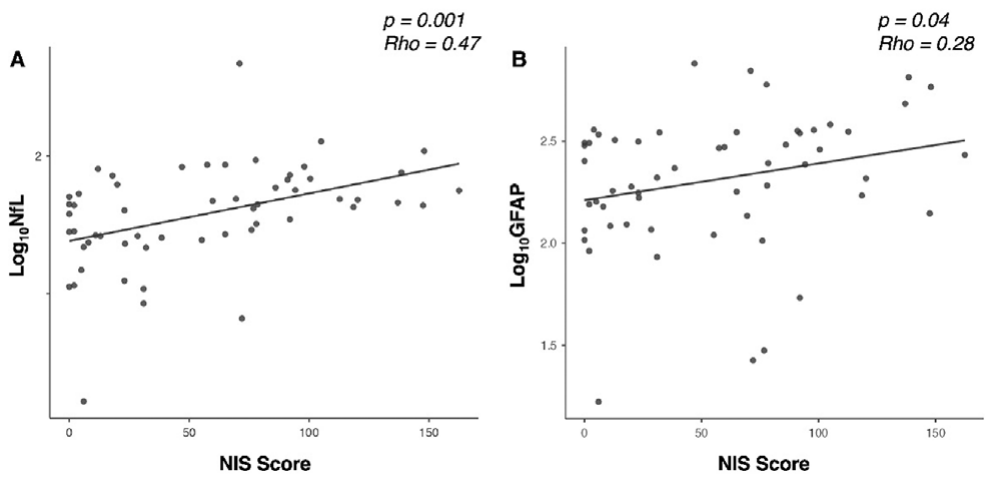
**FIGURE 3** Correlation between sNfL and sGFAP levels (expressed as Log10‐NfL and Log10‐GFAP, respectively) with NIS scores, demonstrating a significant positive relationship between both biomarkers and clinical impairment.

**Conclusion:** sGFAP and sNfL mark distinct ATTRv stages, with sGFAP indicating early preclinical changes and sNfL correlating with neurological progression. Sex differences in sGFAP levels among HCs suggest that sex should be considered as a covariate in biomarker analyses.

**Disclosure:** Nothing to disclose.

## OPR‐026

### Baseline characteristics of patients with transthyretin amyloidosis with polyneuropathy: Results from overTTuRe

#### L. Galan^1^; K. Hahn ^2^; J. Smith^3^; E. Wittbrodt^4^; A. Bhimjiyani^5^; B. Pilebro^6^

##### ^1^Neurology Department, Clínico San Carlos Hospital, IdiSSC, Madrid, Spain; ^2^Amyloidosis Center Charité Berlin (ACCB), Charité ‐ Universitätsmedizin Berlin, Berlin, Germany; ^3^The Wallenberg Laboratory/Department of Molecular and Clinical Medicine, Institute of Medicine, Gothenburg University, Gothenburg, Sweden; ^4^Cardiovascular, Renal and Metabolism Evidence Strategy, BioPharmaceuticals Medical, AstraZeneca, Wilmington, DE, US; ^5^Cardiovascular, Renal and Metabolism Evidence Strategy, BioPharmaceuticals Medical, AstraZeneca, Gothenburg, Sweden; ^6^Heart Centre, Cardiology, Department of Public Health and Clinical Medicine, Umeå University, Umeå, Sweden

**Background and Aims:** Transthyretin amyloidosis with polyneuropathy (ATTR‐PN) is a rare, debilitating, and ultimately fatal disease, which manifests as progressive peripheral nerve damage. The condition is associated with misdiagnosis and diagnostic delays before patients receive appropriate treatment. The objective of this analysis was to report on baseline characteristics of patients with ATTR‐PN from OverTTuRe.

**Methods:** OverTTuRe is a multi‐country, retrospective, observational study generating real‐world evidence from adult patients diagnosed with ATTR amyloidosis. The study population included patients recorded in the Swedish Transthyretin Amyloidosis Registry (SveaTTR, 2000–2024) and patients sampled through chart review at 11 Spanish hospitals (2009–2023). Analyses from Germany are ongoing and will be presented. Patients were assigned to ATTR‐PN and mixed phenotypes according to baseline clinical presentation and based upon clinician judgement (Spain) and neurological and cardiac symptoms recorded in SveaTTR (Sweden).

**Results:** In total, 279 patients were included from Sweden and 257 from Spain. Baseline patient characteristics are presented in the table. Peripheral neuropathy was the most common neurological manifestation at diagnosis in both countries. Non‐neurological manifestations were defined differently in both data sources but clearly indicated a greater prevalence in patients with mixed phenotype.

**Figure:**
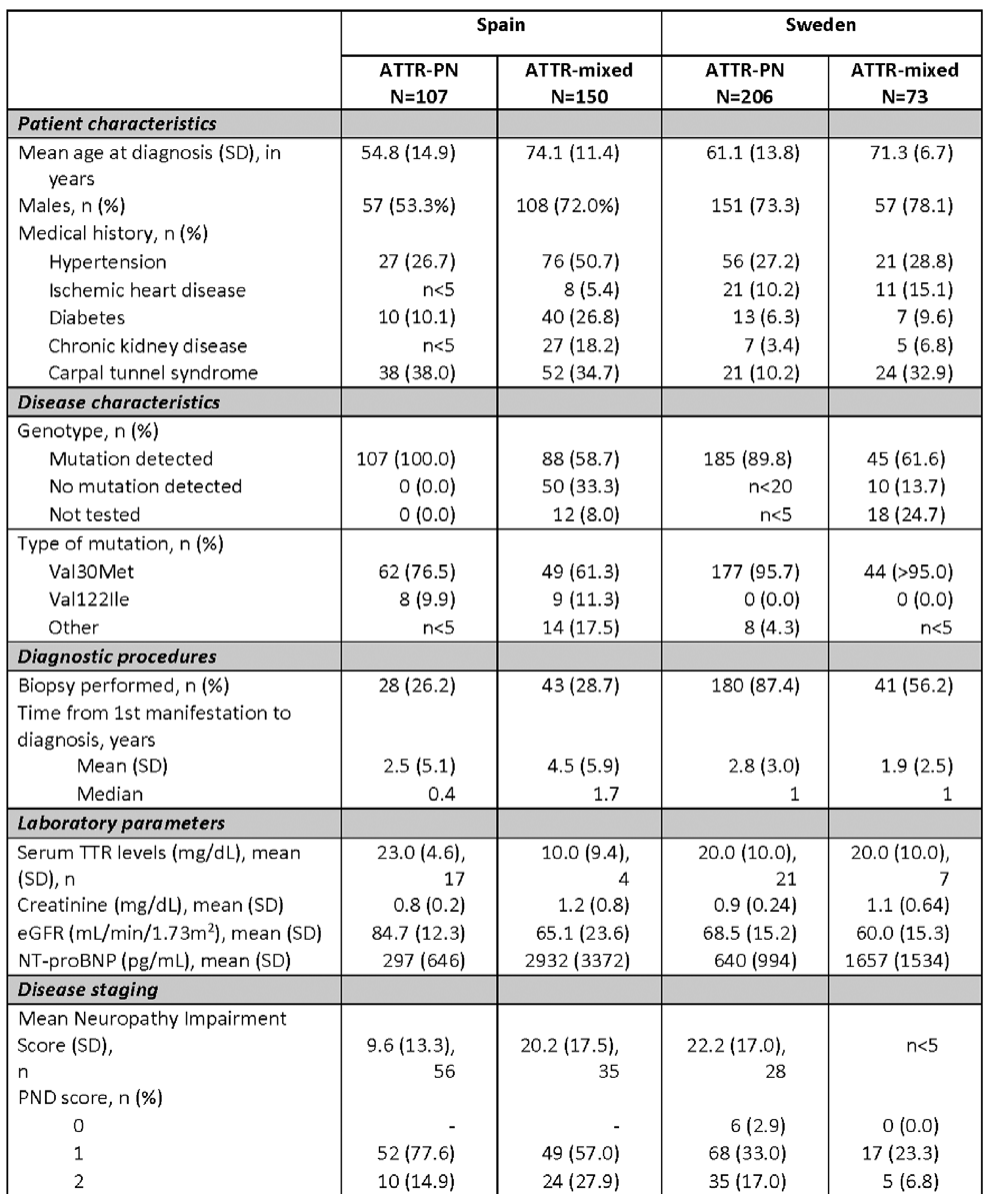
**TABLE** Baseline characteristics of patients with ATTR‐PN and ATTR‐Mixed at diagnosis in Spain and Sweden. (1 of 2)

**Figure:**
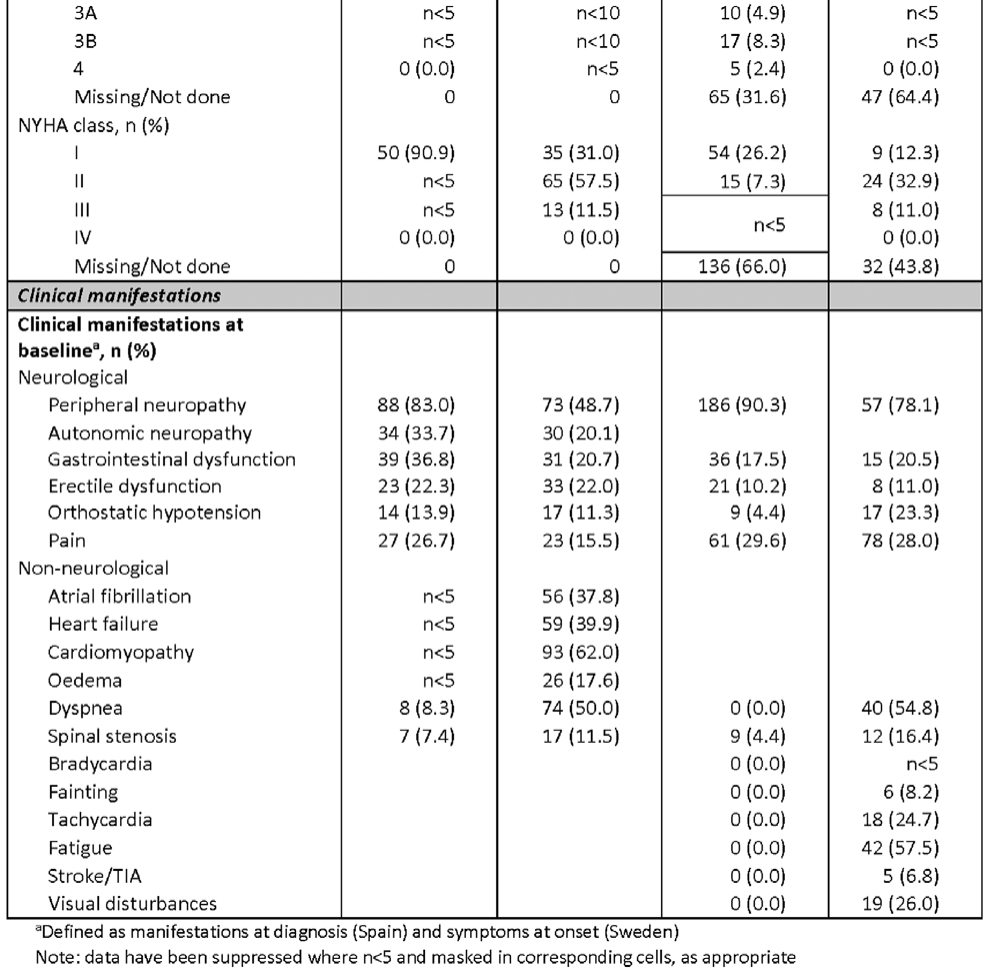
**TABLE** Baseline characteristics of patients with ATTR‐PN and ATTR‐Mixed at diagnosis in Spain and Sweden. (2 of 2)

**Conclusion:** These results provide contemporary real‐world insights into the characteristics of patients with ATTR‐PN from two different countries, highlighting that a considerable proportion of patients show evidence of a mixed phenotype with cardiac manifestations already present at the time of diagnosis. The next phase of OverTTuRe will aim to longitudinally assess the development of ATTR‐related manifestations and disease progression over time.

**Disclosure:** Nothing to disclose.

## OPR‐027

### Proteomic profiling of Guillain‐Barré Syndrome using aptamers: Identifying and Validating Potential Biomarkers

#### R. Collet‐Vidiella ^1^; L. Martín‐Aguilar^1^; C. Lleixà^1^; M. Caballero‐Ávila^1^; C. Tejada‐Illa^1^; E. Pascual‐Goñi^1^; P. Llarch^1^; L. Llucià‐Carol^2^; M. Sedano‐Tous^3^; C. Casasnovas^4^; G. Gutiérrez‐Gutiérrez^5^; J. Pardo‐Fernández^6^; Á. Carbayo^1^; E. Gallardo^1^; A. Vesperinas^1^; L. Llansó^1^; I. Fernández‐Cadenas^2^; L. Querol^1^

##### ^1^Neuromuscular Diseases Unit, Neurology Department, Hospital de la Santa Creu i Sant Pau, Universitat Autònoma de Barcelona, Spain; ^2^Stroke Pharmacogenomics and Genetics, Sant Pau Institute of Research (IR‐Sant Pau), Barcelona, Spain; ^3^Hospital Universitario Marqués de Valdecilla, Santander, Spain; ^4^Hospital Universitario de Bellvitge, Barcelona, Spain; ^5^Hospital Universitario Infanta Sofía, Madrid, Spain; ^6^Hospital Clínico Universitario de Santiago, Santiago de Compostela, Spain

**Background and Aims:** There is a lack of large‐scale serum proteomic data in Guillain‐Barré syndrome (GBS). Our aim is to examine proteomic differences at disease onset and after one year to identify potential biomarkers and relevant pathways, some of which could be druggable.

**Methods:** We analysed serum samples from 20 GBS patients across Spanish centres, comparing their proteomic profiles at disease onset and after one year, as well as against 15 healthy controls (HC). A multiplex aptamer‐based proteomic platform (Somalogic) was used to quantify 6383 serum proteins. Enrichment analysis identified disease‐associated pathways, and candidate proteins were validated with conventional laboratory methods.

**Results:** Nineteen proteins were differentially expressed between onset and remission, with overexpression of pathways related to B cell activation, cell cycle regulation, and deubiquitination at onset. Compared to HC, 177 proteins were differentially expressed at onset in GBS, particularly in pathways related to muscle sarcomere, antimicrobial response, and lipid metabolism. Serum Amyloid A1 (SAA1), an acute response protein, showed the largest change between onset and remission, confirmed by Meso Scale Discovery: GBS patients had a geometric mean SAA1 concentration of 14.529 ng/mL at onset, decreasing to 4.613 ng/mL at 12 months (*p* < 0.001) and 2.523 ng/mL in HC (*p* < 0.001). None of the differentially expressed sarcomeric proteins tested so far were detected using ELISA.

**Figure:**
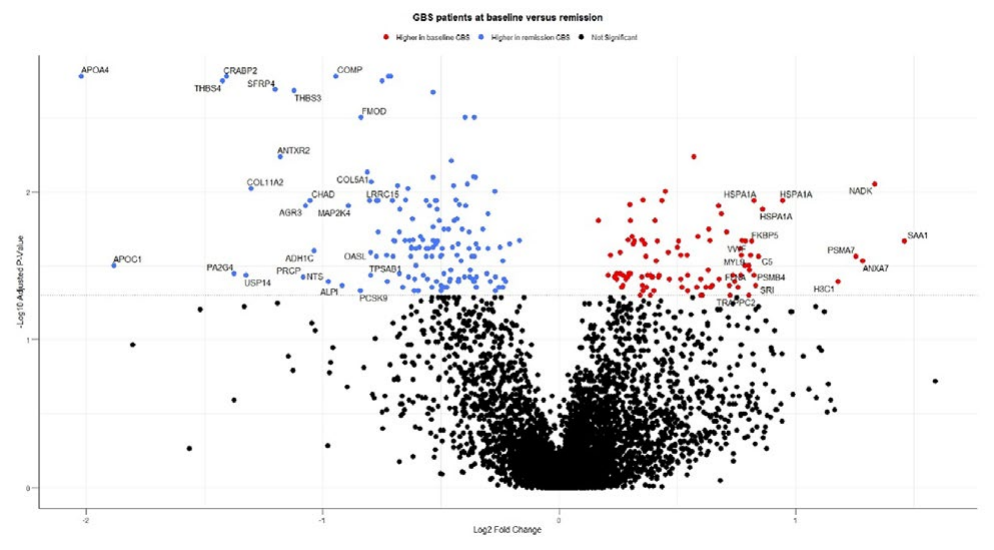
**FIGURE 1** Volcano plot showing differentially expressed proteins in the serum of patients with Guillain‐Barré Syndrome at baseline comparing with the same patients at one year.

**Figure:**
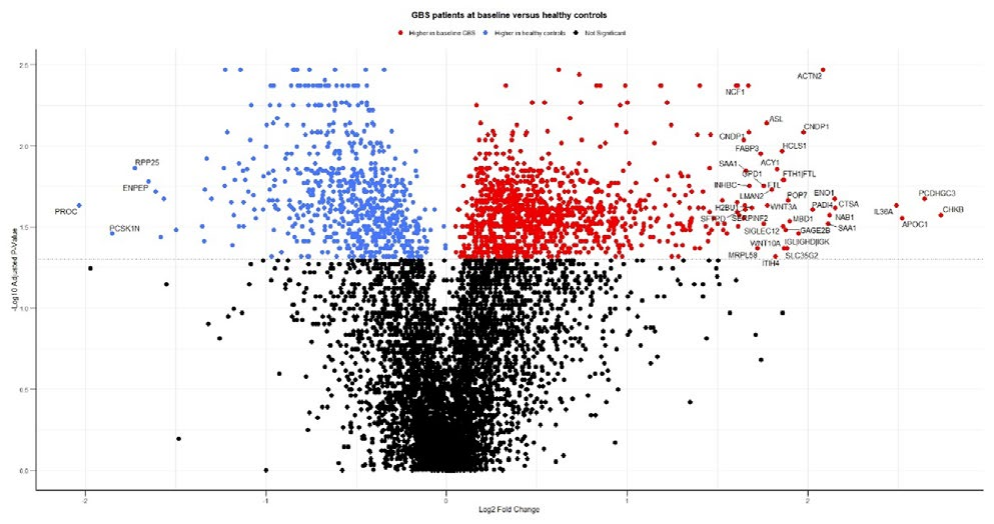
**FIGURE 2** Volcano plot showing differentially expressed proteins in the serum of patients with Guillain‐Barré Syndrome at baseline comparing with healthy controls.

**Figure:**
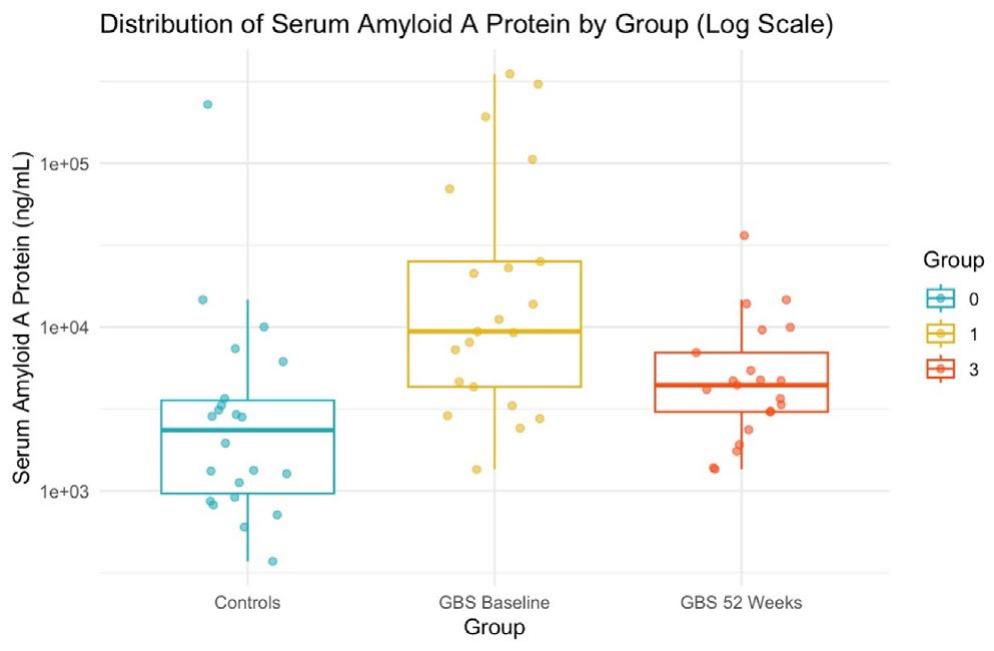
**FIGURE 3** Serum amyloid A1 (SAA1) levels in Healthy Controls and Guillain‐Barré syndrome patients at onset, and at one year.

**Conclusion:** This first large‐scale plasma proteomic analysis in GBS patients highlights multiple disease‐associated proteins and pathways. SAA1 emerges as a potential biomarker in GBS, but further validation is needed to confirm its clinical utility. Further analysis in additional candidate proteins is also ongoing.

**Disclosure:** Nothing to disclose.

Neuroimmunology 1

## OPR‐028

### Exploring PML risk assessment: A comparative study of STRATIFY JCV™ DxSelect™ and IMMUNOWELL™ JCV IgG tests in RRMS

#### E. D'Amico ^1^; F. Marinelli^2^; P. Di Filippo^1^; C. Avolio^1^; A. Zanghì^1^

##### ^1^University of Foggia, Italy; ^2^Neurology Unit, MS center, F. Spaziani Hospital, ASL Frosinone, Italy

**Background and Aims:** In the clinical management of Multiple Sclerosis (MS) patients, the primary concern associated with natalizumab therapy remains the risk of developing Progressive Multifocal Leukoencephalopathy (PML), a rare but potentially fatal opportunistic infection of the central nervous system caused by the J.C. virus (JCV).

**Methods:** This study compared two tests for assessing the risk of PML in patients with relapsing‐remitting MS (RRMS) treated with natalizumab (branded, Tysabri®): the STRATIFY JCV™ DxSelect™ test and the IMMUNOWELL™ JCV IgG test. The main objective was to determine the comparability of these tests in classifying PML risk. Demographic data, clinical characteristics, treatment history, and results from both STRATIFY JCV™ DxSelect™ and IMMUNOWELL™ JCV IgG tests were collected on the same day. Patients were classified into three risk categories (low, intermediate, high) based on each test's threshold values.

**Results:** The analysis showed 85.5% agreement between the two tests for risk classification. Ten discordant cases were identified, mainly between intermediate and high‐risk categories. IMMUNOWELL™ tended to classify more patients in higher risk categories than STRATIFY JCV™ DxSelect™. No significant association was found between discordance and prior use of immunosuppressant drugs and number of administrations>24. The agreement between tests, assessed with the weighted Kappa coefficient, was moderate (κ = 0.6222).

**Conclusion:** Our study first described in a real‐world setting that the IMMUNOWELL™ JCV test tends to classify more patients in higher risk categories compared to STRATIFY JCV™ DxSelect™. Further longitudinal studies are needed to evaluate the clinical impact of these differences in PML risk assessment.

**Disclosure:** Nothing to disclose.

## OPR‐029

### Clinical manifestation progression and long term outcome of anti‐KLHL11 encephalitis

#### M. Jin ^1^; T. Brand^1^; E. Erdag Turgeon^1^; A. C. Havelaar^1^; S. Veenbergen^2^; J. de Vries^1^; J. Kerstens^1^; R. van Steenhoven^1^; M. Nagtzaam^1^; S. Franken^1^; M. Titulaer^1^

##### ^1^Department of Neurology, Erasmus Medical Center, Rotterdam, The Netherlands; ^2^Department of Immunology, Erasmus Medical Center, Rotterdam, The Netherlands

**Background and Aims:** Anti‐Kelch‐like protein 11 (KLHL11) encephalitis was discovered in 2019 in middle‐aged males with testicular seminoma and rhombencephalitis. This study aimed to comprehensively define phenotype and outcome of KLHL11‐encephalitis.

**Methods:** We tested 1361 patients with features of possible KLHL11‐encephalitis (680 serum; 1164 cerebrospinal fluid) referred to our national reference center. The KLHL11 antibodies were screened using in‐house HEK293 KLHL11 overexpression cell‐based assay and positive samples were further analyzed by immunohistochemistry. Detailed clinical and follow‐up information was collected.

**Results:** Seventeen patients with KLHL11‐encephalitis were identified. The median age of patients was 59 (range 28–76) years old, 12 individuals (71%) were male. Common phenotypes were cerebellar ataxia (*n* = 12, 71%), brainstem encephalitis (*n* = 12, 71%), opsoclonus myoclonus (*n* = 8, 47%), limbic encephalitis (*n* = 3, 18%) and respiratory or consciousness disorder (*n* = 4, 24%). Meningitis was observed in 1 patient. Concurrent antibodies included NMDAR (*n* = 2), GFAP (*n* = 1) and CASPR2 (*n* = 1). MRI was abnormal in 8 cases (47%), showing hyperintensity in rhombencephalon (*n* = 3, 18%) or limbic system (*n* = 4, 24%). Tumors were identified in 11 (65%) cases, including seminoma (*n* = 5, 29%), teratoma (*n* = 2, 12%), small cell lung cancer, renal cell carcinoma, urinary tract cancer, unknown primary (all *n* = 1). Fifteen patients received first‐line immunotherapy (88%), and 6 second‐line immunotherapy (35%). Improvement or stabilization was achieved in 10/15 (67%) patients. The median follow‐up duration was 20 (1.5–180) months and 6 patients died in this period.

**Figure:**
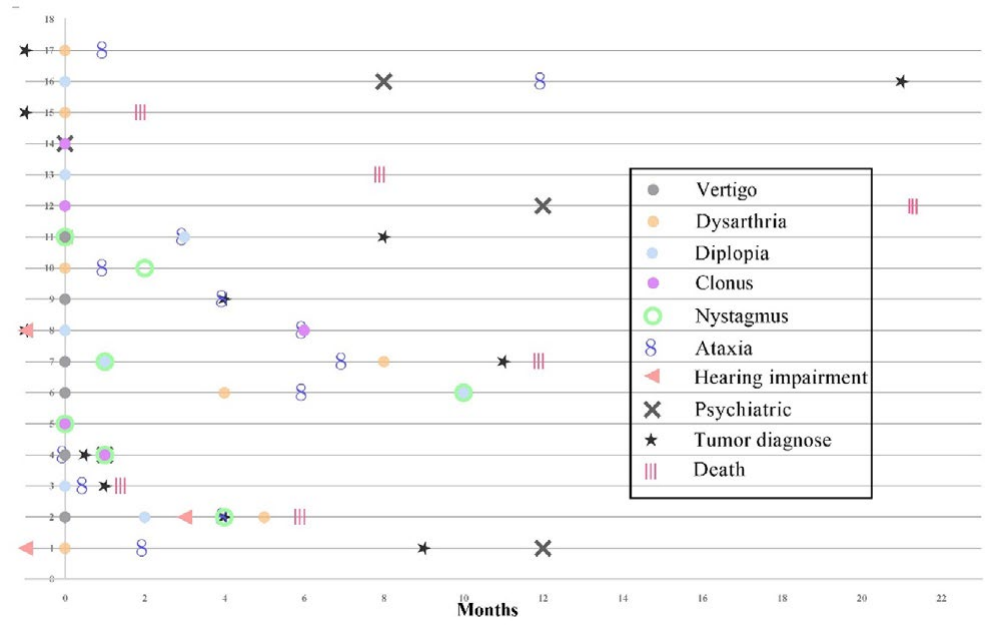
**FIGURE 1** Timeline of KLHL11 patient's initial symptoms, tumor diagnose and death.

**Figure:**
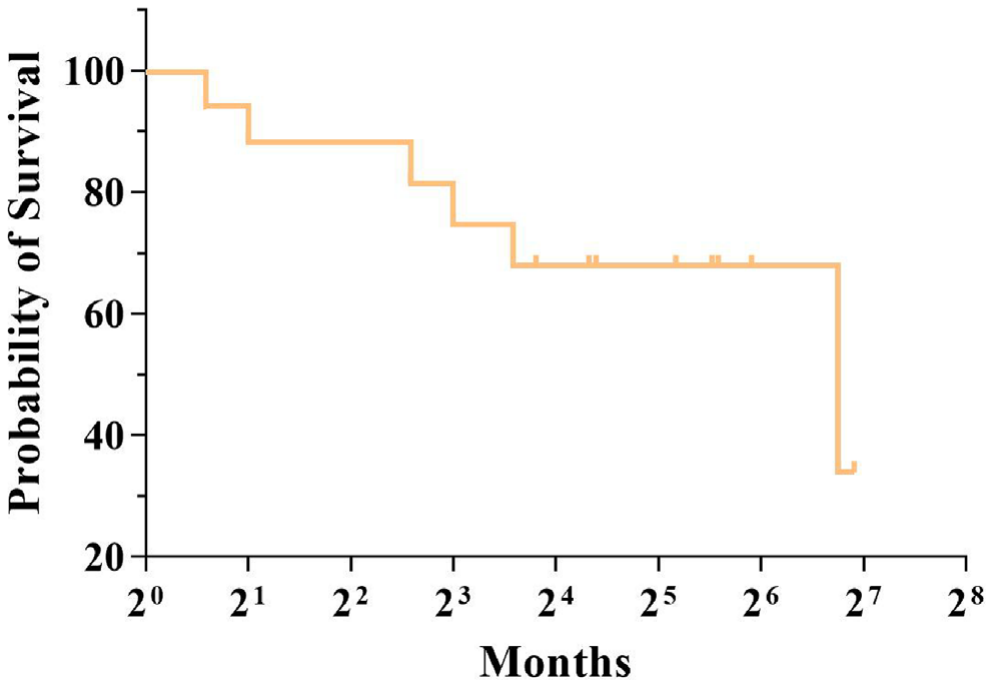
**FIGURE 2** Kaplan‐Meier curve shows the survival probability of KLHL11 patients

**Conclusion:** KLHL11‐encephalitis mainly related to infratentorial involvement, but can present as limbic encephalitis. Early diagnosis enables early oncological and immunological treatment, improving outcomes.

**Disclosure:** M.J. is supported in part by the Chinese scholarship council program (Project ID 202308430038) and the First Affiliated Hospital of Nanchang University.

## OPR‐030

### Age‐related dynamics of GFAP blood levels in normal ageing: Implications for biomarker studies in neurological diseases

#### R. Demjaha ^1^; S. Seiler^2^; E. Hofer^3^; C. Tafrali^1^; M. Martínez‐Serrat^1^; L. Pirpamer^5^; P. Opriessnig^2^; S. Ropele^2^; D. Pinter^4^; S. Hechenberger^4^; B. Helmlinger^4^; D. Leppert^6^; P. Benkert^6^; J. Kuhle^6^; C. Enzinger^2^; R. Schmidt^2^; M. Khalil^1^

##### ^1^Medical University of Graz, Department of Neurology, Neurology Biomarker Research Unit, Graz, Austria; ^2^Medical University of Graz, Department of Neurology, Graz, Austria; ^3^Medical University of Graz, Institute for Medical Informatics, Statistics and Documentation, Graz, Austria; ^4^Medical University of Graz, Research Unit for Neuronal Plasticity and Repair, Graz, Austria; ^5^University of Basel, Medical Image Analysis Center (MIAC) and Department of Biomedical Engineering, Basel, Switzerland; ^6^University Hospital and University of Basel, Multiple Sclerosis Centre and Research Center for Clinical Neuroimmunology and Neuroscience (RC2NB), Departments of Biomedicine and Clinical Research, Basel, Switzerland

**Background and Aims:** GFAP is an astrocytic biomarker that is upregulated in various neurological conditions, including multiple sclerosis (MS). In MS, higher serum GFAP (sGFAP) are associated with disability worsening and MS‐related MRI changes. For correct interpretation of sGFAP, detailed knowledge of its variability with age and its temporal dynamics in neurologically inconspicuous individuals is crucial. This has not been investigated.

**Methods:** 316 (mean age = 64.5±10.7 years, range = 38–82, 184 female) neurologically inconspicuous individuals participating in a community‐dwelling cohort study, with longitudinal data (mean follow‐up duration = 5.6±1.0 years) available from 89 participants, were included. Participants underwent comprehensive diagnostic work‐up including a detailed neurological examination, 3T‐brain‐MRI, cognitive, and laboratory evaluation. sGFAP was measured using a single molecule array.

**Results:** sGFAP significantly increases with age (*r* = 0.5, *p* < 0.001) ( < 50 years (pg/mL, mean±SD) (73.1±25.4, 50–60 years (86.8±35.1), 60–70 years 136.9±48.4),>70 years (154.6±60.7) and tendentially higher sGFAP levels are found in females compared to males (*p* = 0.05). The increase of sGFAP with age is accompanied with an increase in the variability of this marker in the older age groups (*p* < 0.05). Longitudinal analyses showed a significant difference between males and females (*p* = 0.02), with a larger sGFAP increase in females.

**Conclusion:** sGFAP levels increase with age, which is accompanied by a higher variability of this marker in older individuals. This high variability of sGFAP in neurologically normal individuals needs to be taken into account when interpreting this marker in neurological disorders and requires the establishment of normative values, e.g. based on percentiles or Z‐scores. Analyses of potential relationships between sGFAP, brain‐MRI, cognitive measures, and other factors are ongoing.

**Disclosure:** R.D: received travel funding from Janssen, Novartis and Sanofi D.P: is in the advisory board for “Cognition and MS” for Novartis and received speaking honoraria from Biogen, Novartis, MedAhead and Bristol‐Myers Squibb S.H; B.H: received speaking honoraria from Roche and Bristol‐Myers Squibb D.L: was Chief Medical Officer of GeNeuro until end of 2023 J.K: has received speaker fees, research support, travel support, and/or served on advisory boards by Swiss MS Society, Swiss National Research Foundation (320030_212534/1), University of Basel, Progressive MS Alliance, Alnylam, Bayer, Biogen, Bristol Myers Squibb, Celgene, Immunic, Merck, Neurogenesis, Novartis, Octave Bioscience, Quanterix, Roche, Sanofi, Stata DX. C.E: has received funding for traveling and speaker honoraria from Biogen Idec, Bayer Schering Pharma, Merck Serono, Novartis, Genzyme and Teva Pharmaceutical Industries Ltd./Sanofi‐Aventis, Shire; received research support from Merck Serono, Biogen Idec, and Teva Pharmaceutical Industries Ltd./Sanofi‐Aventis; and serves on scientific advisory boards for Bayer Schering Pharma, Biogen Idec, Merck Serono, Novartis, Genzyme, Roche, and Teva Pharmaceutical Industries Ltd./Sanofi‐ Aventis M.K: received travel funding and speaker honoraria from Bayer, Biogen, Novartis, Merck, Sanofi and Teva and serves on scientific advisory boards for Biogen, Bristol‐Myers Squibb, Gilead, Merck, Novartis, and Roche. He received research grants from Biogen, Novartis and Teva Others: no disclosures.

## OPR‐031

### Conformational antibodies to proteolipid protein‐1 in patients with CNS autoimmune demyelinating disorders

#### S. Masciocchi ^1^; P. Businaro^1^; G. Greco^1^; S. Scaranzin^1^; A. Malvaso^1^; C. Morandi^1^; E. Zardini^1^; M. Risi^2^; M. Giannoccaro^3^; V. De Giuli^4^; C. Zanetta^5^; R. Lanzillo^6^; A. Bisecco^2^; M. Di Filippo^7^; A. Toriello^8^; I. Volonghi^9^; T. Bocci^10^; M. Paoletti^11^; E. Colombo^12^; M. Filippi^5^; A. Pichiecchio^11^; E. Marchioni^13^; D. Franciotta^1^; M. Gastaldi^1^

##### ^1^Neuroimmunology Research Section, IRCCS Mondino Foundation, Pavia, Italy; ^2^Department of Advanced Medical and Surgical Sciences, University of Campania “Luigi Vanvitelli”, Naples, Italy; ^3^Department of Biomedical and Neuromotor Sciences, University of Bologna (DIBINEM), Bologna, Italy; ^4^Neurology Unit, ASST Cremona, Cremona, Italy; ^5^Stroke Unit, Neurophysiology Service, and Neuroimaging Research Unit, Division of Neuroscience, San Raffaele Scientific Institute, Vita‐Salute San Raffaele University, Milan, Italy; ^6^University of Naples, Naples, Italy; Multiple Sclerosis Unit, Policlinico Federico II University Hospital, Naples, Italy; ^7^Section of Neurology, Department of Medicine, University of Perugia, Perugia, Italy; ^8^Department of Medicine, Surgery and Dentistry “Scuola Medica Salernitana”, Neuroscience Section, University of Salerno, Salerno, Italy; ^9^Department of Clinical and Experimental Sciences, Neurology Unit, University of Brescia, Brescia, Italy; ^10^III Neurology Clinic, ASST‐Santi Paolo e Carlo University Hospital, 20142 Milan, Italy; ^11^Neuroradiology Department, IRCCS Mondino Foundation, Pavia, Italy; ^12^Multiple Sclerosis Unit, IRCCS Mondino Foundation, Pavia, Italy; ^13^Neuroncology and Neuroinflammation Unit, IRCCS Mondino Foundation, Pavia, Italy

**Background and Aims:** Antibodies to proteolipid‐protein‐1 (PLP1‐IgG), a major central myelin protein also expressed in the PNS as the isoform DM20, have been previously identified mostly in patients with MS, with unclear clinical implications. However, most studies relied on non‐conformational immunoassays and included few patients with non‐MS CNS autoimmune demyelinating diseases (ADD).

**Methods:** After devising a live cell‐based assay (CBA) for PLP1‐IgG, we tested a retrospective cohort of ADD patients (*n* = 284; non‐MS = 160 ADD; MS = 124) and controls (*n* = 177) for these antibodies. We validated our findings on a prospective cohort of suspect ADD patients (*n* = 820). PLP1‐IgG‐positive samples were tested for IgG subclasses, DM20‐IgG, and on rat brain tissue‐based assay (TBA). PLP1‐IgG‐positive MS and MOGAD patients’ clinical features were compared with those of the PLP1‐IgG‐negative.

**Results:** PLP1‐IgG were found in 0/177 controls and 42/1104 ADD patients mainly diagnosed as other‐ADD (19/42) with frequent myelitis/encephalomyelitis (14/19) and co‐existing PNS involvement (13/19). PLP1‐IgG were also found in MOGAD (11/42), more frequently with PNS involvement (*p* = 0.01), and in MS (12/42), more frequently with atypical features (*p* < 0.001). PLP1‐IgG a) co‐localized with their target on CBA‐TBA, where their binding was abolished after immunoadsorption and fixation‐induced conformational epitope alteration; b) mostly pertained to the IgG1/IgG3 subclass (68.3%) and were able to induce CDC; c) co‐reacted with DM20 in all 12 patients with PNS involvement tested.

**Conclusion:** Conformational PLP1‐IgG are mainly found in non‐MS ADD, where they allow to identify patients with peculiar phenotypes. DM20 co‐reactivity provides a rationale for the PNS involvement. PLP1‐IgG might also represent detrimental prognostic markers in MOGAD and MS.

**Disclosure:** Nothing to disclose.

## Sunday, June 22 2025

## Child Neurology/Developmental Neurology

## OPR‐032

### The role of brain biomarkers in the clinical course of status epilepticus in the pediatric population

#### M. Cebuc ^3^; S. Hadjiu^1^; I. Istratuc^5^; E. Capestru^5^; M. Sprîncean^5^; N. Revenco^2^; S. Groppa^4^; C. Călcîi^5^

##### ^1^Department of Paediatric Neurology, Nicolae Testemițanu State University of Medicine and Pharmacy, Chișinău, Republic of Moldova; ^2^Mother and Child Institute IMSP, Chișinău, Republic of Moldova; ^3^Department of Neurology No1, Diomid Gherman Institute of Neurology and Neurosurgery, Chișinău, Republic of Moldova; ^4^National Epileptology Center, Emergency Institute of Medicine, Chișinău, Republic of Moldova; ^5^Neurobiology and Medical Genetics Laboratory, Nicolae Testemițanu State University of Medicine and Pharmacy, Chișinău, Republic of Moldova

**Background and Aims:** Biomarker study offers a modern approach in the multimodal evaluation of status epilepticus (SE) as per the cytokines’ role in epileptogenesis through neuroinflammation and microglial activation. The research aims to assess the cytokine profile of paediatric patients with SE providing insights into future neurotherapeutic strategies.

**Methods:** This retrospective case‐control study examined 55 paediatric patients with SE of various etiologies and 54 control subjects who were admitted at the Mother and Child Institute from 2019 to 2023. The TGF, Il‐6, Il‐1 alpha, Il‐1 beta and the Il‐1Ra/Il‐1 F3 ratio were investigated by the ELISA immunoenzymatic method. The data analysis was performed using IBM‐SPSS statistical software.

**Results:** Upon the t‐student analysis, the average values of TGF‐beta 1 showed statistically significant differences between the SE and control subjects (4409,4 pg/ml vs. 179,9pg/ml, *p* < 0.001) supporting greater degree of neuroinflammation. Similar results supporting this statement were obtained in the evaluation of Il‐6 (1431 pg/ml vs 82 pg/ml, *p* < 0.001), Il‐1 beta (90.62 pg/ml vs 5.84 pg/ml, *p* < 0.001), Il‐1 alpha (367.1 pg/ml vs 7.2 pg/ml, *p* < 0.001) serum concentration. The calculated ratio between Il‐1 Ra/ Il‐1 F3, used to profile the capacity of neurons to maintain homeostasis, revealed greater disbalance in SE subjects (480 pg/ml vs 86 pg/ml, *p* < 0.021).

**Conclusion:** The analysis of cytokine levels suggests disruption of immunomodulatory processes with a strong proinflammatory state and compromised protective mechanisms, which could contribute to the pathogenesis of status epilepticus in children. Further research is required to explore the usage of cytokine profiles to guide care in SE.

**Disclosure:** Nothing to disclose.

## OPR‐033

### Early predictors of long‐term clinical outcomes in pediatric multiple sclerosis: A 12‐year longitudinal study

#### M. Rocca ^1^; M. Margoni^2^; A. Meani^3^; E. Pagani^3^; P. Preziosa^1^; L. Moiola^4^; M. Pozzato^5^; E. Tavazzi^6^; F. Mattioli^7^; V. Torri Clerici^8^; M. Filippi^9^

##### ^1^Neuroimaging Research Unit, Division of Neuroscience, and Neurology Unit, IRCCS San Raffaele Scientific Institute, and Vita‐Salute San Raffaele University, Milan, Italy; ^2^Neuroimaging Research Unit, Division of Neuroscience, Neurology Unit, and Neurorehabilitation Unit, IRCCS San Raffaele Scientific Institute, Milan, Italy; ^3^Neuroimaging Research Unit, Division of Neuroscience, IRCCS San Raffaele Scientific Institute, Milan, Italy; ^4^Neurology Unit, Division of Neuroscience, IRCCS San Raffaele Scientific Institute, Milan, Italy; ^5^Neuroimmunology Unit, Multiple Sclerosis Centre, ASST Valle Olona, Gallarate, VA, Italy; ^6^Multiple Sclerosis Centre, IRCCS Mondino Foundation, Pavia, Italy; ^7^Neuropsychology Unit, Spedali Civili of Brescia, Brescia, Italy; ^8^Fondazione IRCCS Istituto Neurologico “C. Besta” U.O. Neuroimmunologia e Malattie Neuromuscolari, Milan, Italy; ^9^Neurology Unit, Neurorehabilitation Unit, Neurophysiology Service, and Neuroimaging Research Unit, Division of Neuroscience, IRCCS San Raffaele Scientific Institute, and Vita‐Salute San Raffaele University, Milan, Italy

**Background and Aims:** This study investigates clinical and MRI predictors of key long‐term clinical outcomes in pediatric MS over a median follow‐up of 12.3 years.

**Methods:** Timing of first and subsequent relapses, 6‐month confirmed Expanded Disability Status Scale (EDSS) worsening and EDSS worsening at last follow‐up were recorded. Conventional, volumetric and diffusion tensor MRI sequences were acquired to assess lesion count, brain and choroid plexus (CP) volumetric measures, normal‐appearing (NA) WM fractional anisotropy (FA), mean, axial and radial diffusivities.

**Results:** At follow‐up, 69% (36/52) of patients experienced a clinical relapse, 21% (11/52) had a confirmed disability worsening event and 35% (18/52) had EDSS worsening. Higher number of infratentorial lesions (hazard ratio[HR] = 1.09, 95%‐confidence interval[CI] = 1.02;1.18), presence of spinal cord lesions (HR = 2.45, 95%‐CI = 1.18;5.10), higher CP volume (HR = 1.58, 95%‐CI = 0.93;2.68), and lower thalamic volume (HR = 0.77, 95%‐CI = 0.60;0.99) at baseline predicted a shorter time to first relapse, whereas use of high‐efficacy treatment (HET) showed protective effect (HR = 0.20, 95%‐CI = 0.06;0.68). Higher EDSS (HR = 1.29, 95%‐CI = 0.96;1.73), brain WM lesion volume (HR = 1.52, 95%‐CI = 0.95;2.44), the presence of spinal cord lesions (HR = 1.04, 95%‐CI = 1.01;1.06), and higher CP volume (HR = 1.35, 95%‐CI = 1.00;1.83) at baseline increased relapse risk, whereas HET lowered relapse risk (HR = 0.21, 95%‐CI = 0.11;0.40). Younger age (HR = 0.82, 95%‐CI = 0.68;0.98) and lower NAWM FA (HR = 0.67, 95%‐CI = 0.51–0.88) were associated to a shorter time to a confirmed disability worsening event. Lower NAWM FA also predicted greater EDSS worsening (β = −.26, 95%‐CI = −0.50; −0.03) at follow‐up.

**Conclusion:** MRI markers of focal and diffuse inflammatory activity and thalamic atrophy predict long‐term disease outcomes in pediatric MS. HETs delayed time to first relapse and reduced overall relapse risk.

**Disclosure:** MAR consulting fees from Biogen, Bristol Myers Squibb, Eli Lilly, Janssen, Roche, and speaker honoraria from AstraZaneca, Biogen, Bristol Myers Squibb, Bromatech, Celgene, Genzyme, Horizon Therapeutics Italy, Merck Serono SpA, Novartis, Roche, Sanofi and Teva. MM grants and personal fees from Sanofi Genzyme, Merck Serono, Roche, Biogen, Amgen, Novartis. MA, EP, FM nothing. PP speaker honoraria from Roche, Biogen, Novartis, Merck, Bristol Myers Squibb, Genzyme, Horizon, Sanofi. LM consulting and speaking fees from Biogen, Bristol‐Myers Squibb, Novartis, Roche, Sanofi‐Genzyme, Merck‐Serono, Biogen, Alexion. MP grants and personal fees from Roche, Merck Serono, Janssen‐Cilag, Sanofi, Biogen, Novartis, Amgen, Alexion, Almirall. ET speaking fees from Biogen Idec, Bristol‐Meyers‐Squibb. VTC consulting or speaking fees from Biogen Idec, Teva, Novartis, Genzyme, Almirall. MF consulting or speaking fees from Alexion, Almirall, Bayer, Biogen, Celgene, Chiesi Italia SpA, Eli Lilly, Genzyme, Janssen, Merck‐Serono, Neopharmed Gentili, Novartis, Novo Nordisk, Roche, Sanofi Takeda, and TEVA; Advisory Boards for Alexion, Biogen, Bristol‐Myers Squibb, Merck, Novartis, Roche, Sanofi, Sanofi‐Aventis, Sanofi‐Genzyme, Takeda; scientific direction of educational events for Biogen, Merck, Roche, Celgene, Bristol‐Myers Squibb, Lilly, Novartis, Sanofi‐Genzyme; research support from Biogen Idec, Merck‐Serono, Novartis, Roche.

## OPR‐034

### Mapping upper cervical cord abnormalities in early pediatric multiple sclerosis

#### P. Preziosa ^1^; M. Margoni^2^; P. Valsasina^3^; M. Rubin^4^; M. Gueye^4^; L. Moiola^5^; M. Filippi^6^; M. Rocca^1^

##### ^1^Neurology Unit, and Neuroimaging Research Unit, Division of Neuroscience, IRCCS San Raffaele Scientific Institute, and Vita‐Salute San Raffaele University, Milan, Italy; ^2^Neuroimaging Research Unit, Division of Neuroscience, Neurology Unit, and Neurorehabilitation Unit, IRCCS San Raffaele Scientific Institute, Milan, Italy; ^3^Neuroimaging Research Unit, Division of Neuroscience, IRCCS San Raffaele Scientific Institute, Milan, Italy; ^4^Neuroimaging Research Unit, Division of Neuroscience, and Neurology Unit, IRCCS San Raffaele Scientific Institute, Milan, Italy; ^5^Neurology Unit, IRCCS San Raffaele Scientific Institute, Milan, Italy; ^6^Neurology Unit, Neurorehabilitation Unit, Neurophysiology Service, and Neuroimaging Research Unit, Division of Neuroscience, IRCCS San Raffaele Scientific Institute, and Vita‐Salute San Raffaele University, Milan, Italy

**Background and Aims:** Spinal cord lesions and atrophy, particularly in the cervical region, are common in adult multiple sclerosis (MS) patients and correlate with clinical disability. In pediatric MS, spinal cord damage remains largely unexplored, with two studies reporting neither significant atrophy nor microstructural abnormalities compared to healthy controls (HC). In this study, we aimed to investigate the relationship between upper cord lesions, cord area, and clinical disability in pediatric MS.

**Methods:** Thirty‐eight pediatric MS patients and 13 age‐ and sex‐matched HC underwent clinical and 3T MRI evaluations. Global and voxel‐wise assessment of upper cord lesions and area were performed using brain 3D T1‐weighted scans.

**Results:** Twelve (32%) pediatric MS patients (67% females, median disease duration [interquartile range] = 1.0 [0.4;2.5] year) had 1 or more cervical lesions. No significant differences in upper cord area were observed between HC and MS patients (estimated mean difference [EMD] = 1.7, 95% confidence interval [CI] = −3.4;6.9, *p* = 0.508), or between patients with and without cord lesions (EMD = 3.9, 95% CI = −2.6;10.2, *p* = 0.238). Voxel‐wise analysis revealed no cord atrophy in pediatric MS compared to HC. Increased cord area at C2‐C3 level was observed in patients with cord lesions compared to those without lesions and HC (*p* < 0.001, uncorrected, conjunction analysis). Voxels indicating increased cord area were located in the posterior columns and tended to co‐localize with lesions.

**Conclusion:** No significant upper cord atrophy was observed in pediatric MS patients. Regional area increase in patients with lesions likely reflects inflammation and edema, highlighting the need for prompt, effective treatment in pediatric MS patients.

**Disclosure:** MM grants and personal fees from Sanofi Genzyme, Merck Serono, Roche, Biogen, Amgen, Novartis. PV, MR, MG nothing. PP speaker honoraria from Roche, Biogen, Novartis, Merck, Bristol Myers Squibb, Genzyme, Horizon, Sanofi, and grants from Italian Ministry of Health and FISM. LM consulting and speaking fees from Biogen, Bristol‐Myers Squibb, Novartis, Roche, Sanofi‐Genzyme, Merck‐Serono, Biogen, Alexion. MF compensation for consulting or speaking activities from Alexion, Almirall, Bayer, Biogen, Celgene, Chiesi Italia SpA, Eli Lilly, Genzyme, Janssen, Merck‐Serono, Neopharmed Gentili, Novartis, Novo Nordisk, Roche, Sanofi Takeda, and TEVA; Advisory Boards for Alexion, Biogen, Bristol‐Myers Squibb, Merck, Novartis, Roche, Sanofi, Sanofi‐Aventis, Sanofi‐Genzyme, Takeda; scientific direction of educational events for Biogen, Merck, Roche, Celgene, Bristol‐Myers Squibb, Lilly, Novartis, Sanofi‐Genzyme; research support from Biogen Idec, Merck‐Serono, Novartis, Roche, the Italian Ministry of Health, the Italian Ministry of University and Research, and FISM. MAR consulting fees from Biogen, Bristol Myers Squibb, Eli Lilly, Janssen, Roche, and speaker honoraria from AstraZaneca, Biogen, Bristol Myers Squibb, Bromatech, Celgene, Genzyme, Horizon Therapeutics Italy, Merck Serono SpA, Novartis, Roche, Sanofi and Teva; grants from the MS Society of Canada, the Italian Ministry of Health, the Italian Ministry of University and Research, and FISM.

## OPR‐035

### Comprehensive analysis of clinical, imaging, and genetic features in pediatric metachromatic leukodystrophy: A 15‐year follow up

#### P. Karimzadeh

##### Pediatric Neurology Research Center, Shahid Beheshti University of Medical Sciences

**Background and Aims:** MLD is a rare lysosomal disorder inherited in an AR pattern, which results from the abnormal accumulation of sulfatides in the CNS. Sulfatides, serve as physiological substrates for (ARSA) enzyme, and play a critical role in the structural integrity of myelin. Mutations in the ARSA gene and less frequently in the PSAP gene lead to the accumulation of sulfatides causing progressive myelin damage in the CNS and PNS.

**Methods:** This cross‐sectional observational study was conducted in the Mofid Children's Hospital and enrolled 30 patients with the diagnosis of MLD referred to the Neurometabolic Clinic March 2009‐March 2024. The diagnosis was based on clinical, MRI findings, EMG‐NCV, ARSA enzyme deficiency and was confirmed through direct ARSA gene sequencing. In cases with indecisive ARSA gene sequencing, PSAP direct gene sequencing was performed.

**Results:** In this study, 10 patients showed behavioral disorders and ADHD before MLD presentation. 3 patients presented as motor delay and brain MRI findings were in normal limits. (30.0%) passed away, HPSC was conducted in 7 patients. Among these, 4 patients died post‐transplantation. Of the 3 surviving patients, symptoms stabilized in 2 cases, while in 1 case, where the transplant was received from a carrier brother, symptoms continued to worsen.

**Conclusion:** The current study provides significantly important clinical, laboratory, electromyoneurography, and genetic findings of the pediatric MLD population in a Neurometabolic center in Mofid Children's Hospital in Iran. A critical assessment of the disease characteristics enables a clearer understanding of the pathogenesis for possible of curative therapeutic approaches.

**Disclosure:** Nothing to disclose.

## OPR‐036

### Echocardiographic evaluation of left ventricular function in children with SMA under nusinersen treatment

#### S. Mao ^1^; X. Fu^2^; Y. Feng^3^

##### ^1^Department of Neurology, Children's Hospital, Zhejiang University School of Medicine; ^2^Department of Ultrasonography, Children's Hospital, Zhejiang University School of Medicine; ^3^Department of Neurology, Children's Hospital, Zhejiang University School of Medicine

**Background and Aims:** Spinal muscular atrophy (SMA) is a genetic neuromuscular disease associated with cardiovascular abnormalities. The impact of nusinersen treatment on myocardial function in children with SMA remains unclear. This study aimed to evaluate changes in left ventricular (LV) function in children with SMA before and after nusinersen treatment using echocardiography.

**Methods:** A prospective observational study was conducted on 35 children with SMA who received six doses of nusinersen within 10 months at a tertiary hospital in China. 35 healthy controls were included for comparison. LV function was assessed using echocardiography at baseline and after 10 months of treatment. LV dyssynchrony and myocardial strain were measured using two‐dimensional speckle tracking echocardiography.

**Results:** The mean age of the SMA children was 6.58 ± 3.11 years. Before treatment, the global longitudinal strain (GLS) in the SMA group was significantly lower than in the control group (*p* < 0.001), and LV systolic synchronization was poorer (*p* < 0.001). Following nusinersen treatment, GLS increased (*p* < 0.001) and synchrony improved (*p* = 0.004) in the SMA group. However, even after 10 months of treatment, GLS in the SMA group remained lower than in the control group (*p* = 0.011), and LV synchronization was still inferior (*p* = 0.028).

**Conclusion:** Short‐term nusinersen treatment improved LV function in children with SMA, as evidenced by changes in LV myocardial strain indicators. Further research is warranted to explore the treatment of myocardial injury in SMA patients.

**Disclosure:** nothing to disclose.

## MS and Related disorders 1

## OPR‐037

### Ocrelizumab versus Natalizumab in multiple sclerosis: A propensity‐score study

#### E. Barbuti ^1^; A. Castiello^2^; V. Pozzilli^3^; A. Carotenuto^2^; I. Tomasso^1^; M. Moccia^4^; S. Ruggieri^5^; G. Borriello^6^; R. Lanzillo^2^; V. Brescia Morra^2^; C. Pozzilli^1^; M. Petracca^1^

##### ^1^Department of Human Neurosciences, Sapienza University, Rome, Italy; ^2^Department of Neurosciences, Reproductive Science and Odontostomatology, University of Naples “Federico II”, Naples, Italy; ^3^Unit of Neurology, Neurophysiology and Neurobiology, Campus Bio‐Medico University, Rome, Italy; ^4^Department of Molecular Medicine and Medical Biotechnology, University of Naples “Federico II”, Naples, Italy; ^5^Department of Neurosciences, San Camillo‐Forlanini Hospital, Rome, Italy; ^6^MS Center, San Pietro Fatebenefratelli Hospital, Rome, Italy

**Background and Aims:** Ocrelizumab (OCR) and Natalizumab (NTZ) are highly effective treatments widely used in Multiple Sclerosis (MS). We aim to compare long‐term clinical effectiveness, safety and treatment persistence between the two drugs.

**Methods:** We retrospectively analyzed data from relapsing and progressive patients who started treatment between 2010 and 2019 at “La Sapienza” and “Federico II” Universities. Between‐group differences in age, sex, previous treatment status, clinical and MRI activity at baseline, phenotype and disease duration were adjusted via propensity‐score nearest‐neighbor matching, while differences in the length of follow‐up were adjusted with pairwise censoring. Cox proportional hazard regression models were used with Evidence of disease activity (EDA‐3) and its components (relapses, MRI activity, and confirmed disability progression) as outcomes. Treatment discontinuation and occurrence of adverse events (AEs) were tested using logistic regressions.

**Results:** We identified 308 patients (140 on OCR, 168 on NTZ) with a mean (SD) follow‐up of 75.7 (30.8) months. Patients treated with OCR were older, less active, and less frequently naïve at baseline than NTZ‐treated patients. The PS‐matching procedure retained 140 (70 pairs) patients with a mean follow‐up of 55.9 (14.3) months. No significant differences were found between NTZ and OCR regarding relapses, MRI activity or confirmed disability progression. OCR was associated with a higher risk of AEs, though treatment discontinuation rates were comparable.

**Figure:**
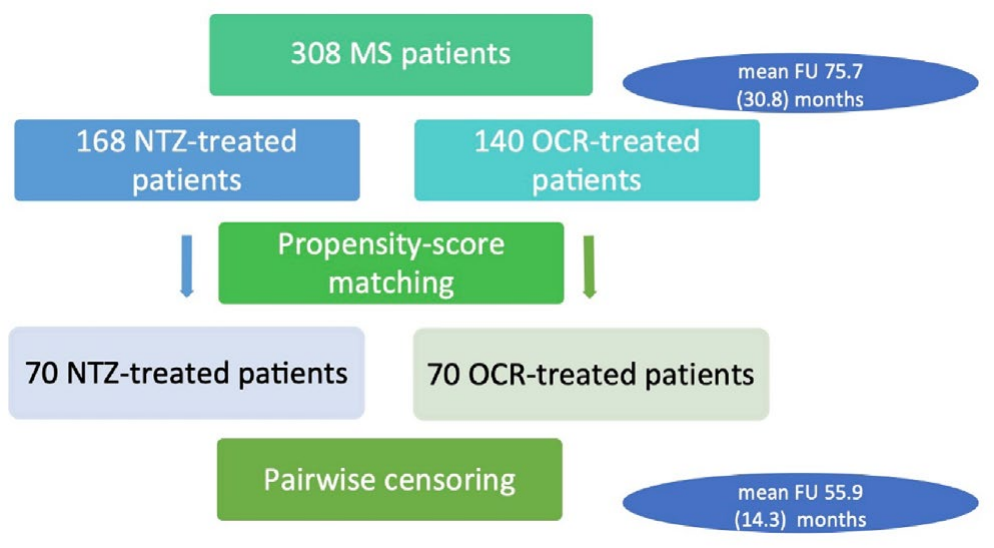
**FIGURE 1** Study flow‐chart. FU: follow‐up, MS: multiple sclerosis, NTZ: natalizumab, OCR: ocrelizumab.

**Figure:**
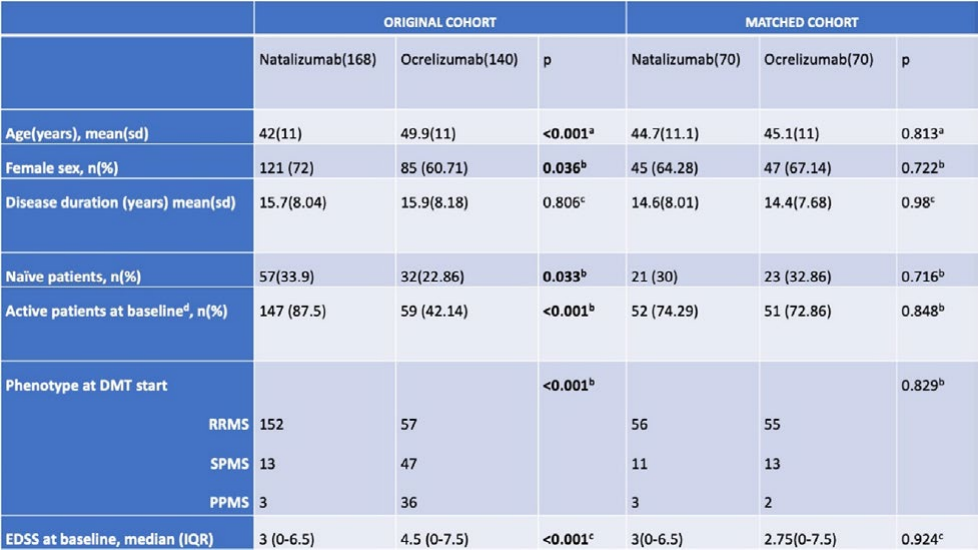
**TABLE 1** Demographic and clinical characteristics of the total cohort and of the matched cohort. a. t‐test for independent values b. X2 c. Mann–Whitney DMT: disease modifying treatment, EDSS: expanded disability status scale, IQR: interquartile range.

**Figure:**
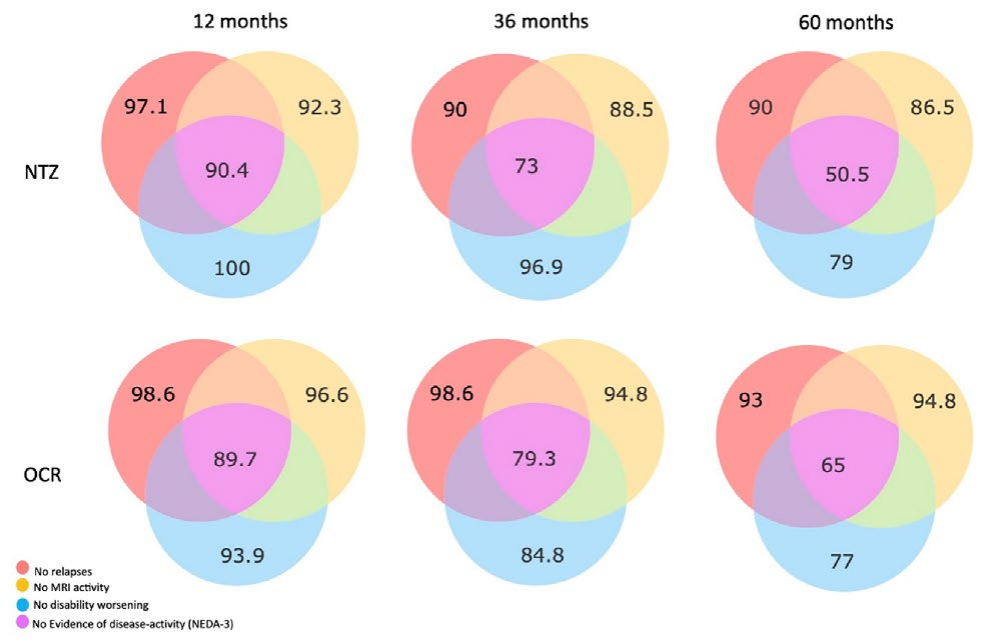
**FIGURE 2** Probability of being free from relapses, MRI activity, EDSS progression and to be NEDA‐3 at 12, 36 and 60 months.

**Conclusion:** This study provides evidence of comparable effectiveness and treatment persistence between OCR and NTZ over 5 years, with OCR being associated with a higher incidence of mild/moderate AEs.

**Disclosure:** Antonio Carotenuto disclosed research grants from ECTRIMS‐MAGNIMS and Almirall, Marcello Moccia has received from MUR PNRR Extended Partnership (MNESYS no. PE00000006, and DHEAL‐COM no. PNC‐E3‐2022‐23683267), ECTRIMS‐ MAGNIMS, UK MS Society, and Merck and salary as Editorial Board Member from Neurology (AAN, MN, US) and Multiple Sclerosis Journal (Sage, UK); Vincenzo Brescia Morra research grants from Italian MS Federation and Roche, Carlo Pozzilli from Biogen, Teva, Novartis, and Genzyme, Maria Petracca from Baroni Foundation and the Italian Ministry of University and Research. Antonio Carotenuto, Marcello Moccia, Vincenzo Bresciamorra, Carlo Pozzilli, Serena Ruggieri, Giovanna Borriello, Roberta Lanzillo received honoraria and funding Novartis, Janssen, Roche, Merck, BMS, Biogen, Almirall, Sanofi‐ Genzyme, Teva, Bayer, Mylan, Viatris, Actelion, HEALTH&LIFE S.r.l., AIM Education S.r.l., FARECOMUNICAZIONE E20. Elena Barbuti, Alessia Castiello, Valeria Pozzilli, Ilaria Tomasso have nothing to disclose.

## OPR‐038

### Real‐world effectiveness and safety of ofatumumab in multiple sclerosis: Data from a 12‐months follow‐up study

#### G. Ferrazzano ^1^; R. Fantozzi^2^; S. Haggiag^3^; D. Landi^4^; F. Napoli^4^; M. Buscarinu^5^; L. Malimpensa^1^; A. Bianco^6^; G. Borriello^7^; E. Barbuti^8^; F. Marinelli^9^; F. Monteleone^10^; F. Marchione^11^; N. Falcone^12^; M. Altieri^1^; G. Leodori^1^; D. Belvisi^1^; F. Buttari^2^; V. Pozzilli^13^; A. Cicia^6^; A. Cortese^14^; F. Sica^10^; A. Landi^12^; E. Ferraro^14^; e. all^1^

##### ^1^Department of Systems Medicine, Tor Vergata University, Via Montpellier, 1, 00133 Rome, Italy; ^2^IRCCS Neuromed, Pozzilli, Italy; ^3^MS Centre, Department of Neurosciences, S. Camillo‐Forlanini Hospital, Rome, Italy; ^4^Multiple Sclerosis Clinical and Research Unit, Department of Systems Medicine, Tor Vergata University, 00133 Rome, Italy; ^5^Department of Neurosciences, Mental Health and Sensory Organs (NESMOS), Sapienza University of Rome, 00189 Rome, Italy; ^6^Multiple Sclerosis Center, Fondazione Policlinico Universitario “A. Gemelli” IRCCS, 00168 Rome, Italy; ^7^Department of Public Health, Federico II University, Naples, Italy; ^8^MS center, Sant'Andrea Hospital, Rome, Italy; ^9^Neurology Unit ‐ MS Center, F. Spaziani hospital, ASL Frosinone, Italy; ^10^Santa Maria Goretti Hospital, 04100 Latina, Italy; ^11^San Camilllo De Lellis Hospital, Rieti, Italy; ^12^Belcolle Hospital, Viterbo, Italy; ^13^Unit of Neurology, Neurophysiology and Neurobiology, Department of Medicine and Surgery, University Campus Bio‐Medico, Rome, Italy; ^14^Multiple Sclerosis Centre, S. Filippo Hospital, Rome, Italy

**Background and Aims:** Ofatumumab (OFA) is a highly effective therapeutic option for multiple sclerosis (MS), but real‐world data on its efficacy and safety remain limited. This study evaluated the efficacy and safety of OFA in a real‐world cohort of relapsing MS patients over 12 months. Additionally, frailty, an age‐related vulnerability assessed using a frailty index (FI), was examined to better characterize the patient population selected for OFA treatment.

**Methods:** Clinical and MRI data were retrospectively collected from 12 MS clinics in Central Italy. Outcomes included the annualized relapse rate (ARR), relapse occurrence, radiological activity, and safety profile. Frailty was categorized using FI.

**Results:** A total of 242 MS patients were included (66% female; mean age 38.9±10.3 years; disease duration 7.7±7.6 years). Of these, 95 were treatment‐naïve, and 147 were switchers (60.8% first switch). The Expanded Disability Status Scale (EDSS) remained stable during follow‐up (*p* > 0.05). Only four relapses occurred, all within the first six months (mean time to relapse: 3.0±1.8 months). ARR significantly decreased from 0.9 to 0.02 (*p* < 0.001). MRI activity was detected in 10 patients within six months and in 3 of the 77 patients at 12 months. Adverse events included flu‐like symptoms (34.3%), injection‐site reactions (8.2%), and infections (18.5%). Among 239 patients assessed for frailty, 187 were fit (FI≤0.10), 30 were less fit (0.10 < FI≤0.21), and 22 were frail (FI>0.21).

**Conclusion:** This real‐world study confirms OFA as an effective and safe therapy, primarily prescribed to patients with low disability and mild frailty.

**Disclosure:** The authors declare no conflicts of interest related to this study.

## OPR‐039

### Perivascular cuffs suggest local B‐ and T‐cell interaction in association with lesion formation in multiple sclerosis

#### H. Engelenburg; E. Westenbrink; E. Runderkamp; C. Hsiao; I. Huitinga; J. Hamann; J. Smolders

##### Neuroimmunology Research Group, Netherlands Institute for Neuroscience, Amsterdam, The Netherlands

**Background and Aims:** Perivascular aggregation of mononuclear cells is a prevalent pathological observation in multiple sclerosis (MS), known as cuffing. Still, little is known about the characteristics of cuffs and their relationship to lesion initiation and progression. Here, we aimed to study ongoing processes in the perivascular compartment of the MS brain in spatial association with white matter (WM) lesion presence.

**Methods:** We characterized *n* = 255 donors from the Netherlands Brain Bank for the presence of perivascular cuffing and investigated the association of this trait with clinical and pathological characteristics. With immunohistochemistry, we quantified proportional abundance of different cell types and functional markers in *n* = 457 cuffs present in different WM lesion‐types in a cohort of *n* = 18 MS brain donors.

**Results:** Donors with detected cuffing (25%) showed a younger age at death, an increased presence of microglia nodules, and a higher brainstem lesion count. Regarding cell type composition, a similar abundance of T cells characterized cuffs in lesions compared to normal‐appearing WM (NAWM). However, CD79a+ B‐lineage cells had a higher abundance in perivascular cuffs in lesions compared to NAWM. Moreover, we show a positive relationship between the ratio of CD4+ to CD8+ cells and the abundance of CD38+ cells, and these CD38+ cells correlated with the abundance of PCNA+ cells, supporting local activation.

**Conclusion:** We show that B‐cell presence spatially associates with lesion presence in MS. The correlation between T‐cell distribution and CD38+ B‐cell presence highlights the importance of perivascular T‐ and B‐cell interaction for lesion formation in MS.

**Disclosure:** JS received research support and/or speaker and/or consulting fee of Biogen, Merck, Novartis, Roche, and Sanofi‐Genzyme. IH and HH received research support from Biogen.

## OPR‐040

### Impact of vascular risk factors on motor performance and sensorimotor network integrity in multiple sclerosis patients

#### M. Albergoni ^1^; P. Preziosa^2^; G. Ritroso^1^; N. Tedone^3^; E. Pagani^1^; L. Storelli^1^; P. Valsasina^1^; M. Filippi^4^; M. Rocca^2^

##### ^1^Neuroimaging Research Unit, Division of Neuroscience, IRCCS San Raffaele Scientific Institute, Milan, Italy; ^2^Neurology Unit, and Neuroimaging Research Unit, Division of Neuroscience, IRCCS San Raffaele Scientific Institute, and Vita‐Salute San Raffaele University, Milan, Italy; ^3^Neuroimaging Research Unit, Division of Neuroscience, IRCCS San Raffaele Scientific Institute, and Vita‐Salute San Raffaele University, Milan, Italy; ^4^Neurology Unit, Neurorehabilitation Unit, Neurophysiology Service, and Neuroimaging Research Unit, Division of Neuroscience, IRCCS San Raffaele Scientific Institute, and Vita‐Salute San Raffaele University, Milan, Italy

**Background and Aims:** Vascular risk factors (VRFs) are associated with more severe disability and neurodegenerative processes in multiple sclerosis (MS). This study explored the impact of VRFs on motor performance, as well as integrity of brain and spinal cord sensorimotor regions in MS patients.

**Methods:** In this cross‐sectional study, 268 MS patients and 180 healthy controls (HC) were grouped by VRF presence (HC‐VRF[+]), MS‐VRF[+]) or absence (HC‐VRF[‐], MS‐VRF[‐]). Disability and motor performance were assessed using the Expanded Disability Status Scale, Timed 25‐Foot Walk and Nine‐Hole Peg Test. Volumetric and diffusion‐weighted MRI data were used to assess brain sensorimotor network and spinal cord structural integrity. Group differences in clinical and MRI measures and interactions between disease status and VRFs were explored. In MS patients, associations between clinical and MRI data were analyzed, focusing on VRF influence.

**Results:** Seventy‐four (41%) HC and 179 (68%) MS patients had VRFs, with smoking being the most prevalent factor. MS‐VRF(+) patients were significantly more disabled and showed worse motor performance compared to MS‐VRF(‐) (pFDR≤0.004). A significant interaction between VRF and disease status on motor performance, deep gray matter volume, anterior cerebellar motor area volume, and medial lemniscus mean diffusivity was found (pFDR≤0.039). In MS patients, the interaction between VRFs and medial lemniscus fractional anisotropy significantly influenced disability (β = 1.931, *p* = 0.042).

**Conclusion:** VRFs are associated with worse disability, motor impairment, and structural damage of specific sensorimotor structures of the brain in MS patients, while sparing spinal cord integrity. Preventing strategies targeting modifiable VRFs could mitigate their impact on MS progression.

**Disclosure:** Matteo Albergoni, Gloria Ritroso, Nicolò Tedone, Elisabetta Pagani, Loredana Storelli, Paola Valsasina have nothing to disclose. PP received speaker honoraria from Roche, Biogen, Novartis, Merck, Bristol Myers Squibb, Genzyme, Horizon and Sanofi. He received research support from Italian Ministry of Health and Fondazione Italiana Sclerosi Multipla (FISM). MF received compensation for consulting services or speaking activities from Alexion, Almirall, Bayer, Biogen, Celgene, Chiesi Italia SpA, Eli Lilly, Genzyme, Janssen, Merck‐Serono, Neopharmed Gentili, Novartis, Novo Nordisk, Roche, Sanofi Takeda, and TEVA; Advisory Boards for Alexion, Biogen, Bristol‐Myers Squibb, Merck, Novartis, Roche, Sanofi, Sanofi‐Aventis, Sanofi‐Genzyme, Takeda; scientific direction of educational events for Biogen, Merck, Roche, Celgene, Bristol‐Myers Squibb, Lilly, Novartis, Sanofi‐Genzyme; he receives research support from Biogen Idec, Merck‐Serono, Novartis, Roche, the Italian Ministry of Health, the Italian Ministry of University and Research, and FISM. MAR received consulting fees from Biogen, Bristol Myers Squibb, Eli Lilly, Janssen, Roche, and speaker honoraria from AstraZaneca, Biogen, Bristol Myers Squibb, Bromatech, Celgene, Genzyme, Horizon Therapeutics Italy, Merck Serono SpA, Novartis, Roche, Sanofi and Teva, she receives research support from the MS Society of Canada, the Italian Ministry of Health, the Italian Ministry of University and Research, and FISM.

## OPR‐041

### Assessing structural differences in late‐onset compared to adult‐onset multiple sclerosis: A multiparametric MRI study

#### N. Tedone ^1^; P. Preziosa^2^; A. Meani^3^; D. Mistri^3^; F. Esposito^4^; M. Filippi^5^; M. Rocca^2^

##### ^1^Neuroimaging Research Unit, Division of Neuroscience, IRCCS San Raffaele Scientific Institute, and Vita‐Salute San Raffaele University, Milan, Italy; ^2^Neuroimaging Research Unit, Division of Neuroscience, and Neurology Unit, IRCCS San Raffaele Scientific Institute, and Vita‐Salute San Raffaele University, Milan, Italy; ^3^Neuroimaging Research Unit, Division of Neuroscience, IRCCS San Raffaele Scientific Institute, Milan, Italy; ^4^Neurology Unit, Division of Neuroscience, IRCCS San Raffaele Scientific Institute, Milan, Italy; ^5^Neurology Unit, Neurorehabilitation Unit, Neurophysiology Service, and Neuroimaging Research Unit, Division of Neuroscience, IRCCS San Raffaele Scientific Institute, and Vita‐Salute San Raffaele University, Milan, Italy

**Background and Aims:** Previous studies have identified MRI differences between late‐onset multiple sclerosis (LOMS) and adult‐onset MS (AOMS), but a comprehensive analysis of global and regional white matter (WM) and gray matter (GM) metrics is lacking. By using a multiparametric approach, we compared the structural MRI profiles of LOMS and AOMS, focusing on global and regional assessment of WM lesions, GM volume and WM diffusivity abnormalities.

**Methods:** 3T MRI scans were acquired for 40 LOMS, 195 sex‐ and disease duration (DD)‐matched AOMS and 175 sex‐ and age‐matched healthy controls (HC, divided into HC‐AOMS = 125, HC‐LOMS = 50). We applied false discovery rate (FDR) for conventional MRI analyses and family‐wise error correction (FWE) for voxel‐wise analyses, with a *p* < 0.05 considered significant.

**Results:** Both MS groups showed significant reductions in all volumetric measurements and higher T2 lesion volumes (T2‐LV) compared to HC, with LOMS showing greater T2‐LV than AOMS (FDR‐p ≤ 0.015). Compared to AOMS, LOMS had higher frequency of WM lesions in the anterior thalamic radiation and forceps major/minor (FWE‐p ≤ 0.002). Compared to HC, both MS groups showed reduced fractional anisotropy and increased mean, axial, and radial diffusivity in most WM tracts, along with widespread GM volume loss (FWE‐p < 0.05). No differences were found between LOMS and AOMS in WM diffusivity metrics, but LOMS showed greater GM volume loss compared to AOMS in the left paracentral lobule, insula, bilateral putamen, and right pre‐/post‐central gyrus (FWE‐p ≤ 0.040).

**Conclusion:** Compared to DD‐matched AOMS, LOMS had a worse structural profile, mostly characterized by more severe GM volume loss and higher WM lesion burden.

**Disclosure:** Funding: Supported by Fondazione Italiana Sclerosi Multipla (FISM2023/S/1). Competing interests. NT, AM, DM and FE nothing to disclose. PP received speaker honoraria from Roche, Biogen, Novartis, Merck, Bristol Myers Squibb, Genzyme, Horizon and Sanofi. He received research support from Italian Ministry of Health and Fondazione Italiana Sclerosi Multipla (FISM). MF received compensation for consulting services or speaking activities from Alexion, Almirall, Bayer, Biogen, Celgene, Chiesi Italia SpA, Eli Lilly, Genzyme, Janssen, Merck‐Serono, Neopharmed Gentili, Novartis, Novo Nordisk, Roche, Sanofi Takeda, and TEVA; Advisory Boards for Alexion, Biogen, Bristol‐Myers Squibb, Merck, Novartis, Roche, Sanofi, Sanofi‐Aventis, Sanofi‐Genzyme, Takeda; scientific direction of educational events for Biogen, Merck, Roche, Celgene, Bristol‐Myers Squibb, Lilly, Novartis, Sanofi‐Genzyme; he receives research support from Biogen Idec, Merck‐Serono, Novartis, Roche, the Italian Ministry of Health, the Italian Ministry of University and Research, and FISM. MAR received consulting fees from Biogen, Bristol Myers Squibb, Eli Lilly, Janssen, Roche, and speaker honoraria from AstraZaneca, Biogen, Bristol Myers Squibb, Bromatech, Celgene, Genzyme, Horizon Therapeutics Italy, Merck Serono SpA, Novartis, Roche, Sanofi and Teva, she receives research support from the MS Society of Canada, the Italian Ministry of Health, the Italian Ministry of University and Research, and FISM.

## Infectious Diseases

## OPR‐042

### Time from clinical suspicion to lumbar puncture; A novel approach to assessing compliance with meningitis guidelines

#### C. McArthur ^1^; E. Drazich‐Taylor^2^; A. Thorowgood^3^; M. Cao^1^

##### ^1^Department of Neurology, Norfolk and Norwich University Hospital NHS Foundation Trust, Norwich, UK; ^2^Dementia Research Centre, University College London, London, UK; ^3^Norwich Medical School, University of East Anglia, Norwich, UK

**Background and Aims:** Bacterial meningitis is a rapidly evolving and time critical condition, with mortality as high as 30%. The diagnosis is pathological with National Institute for Health and Care Excellence recommending a lumbar puncture (LP) within 1 hour (h) of hospital admission, ideally prior to antibiotics. Despite this, the largest UK study to date reports a median time to LP of 16.5h and that most patients are started on antimicrobial beforehand. In this study, we used the novel approach of assessing time of clinical suspension of meningitis to LP as a means of evaluating clinical efficacy.

**Methods:** A retrospective analysis was conducted of 101 patients admitted to a single, tertiary NHS hospital with suspected meningitis over 19 months consecutively. Results reported using descriptive statistics, R‐Studio.

**Results:** The median time for first attempt of LP from clinical suspicion was 15.6h, with median time of successful LP of 21.2h. Overall, 5.9% had a LP attempt within 1h and 23.5% had a successful LP within 4h. Of those delayed beyond one hour, 31.2% were related to preventable factors, including staffing.

**Figure:**
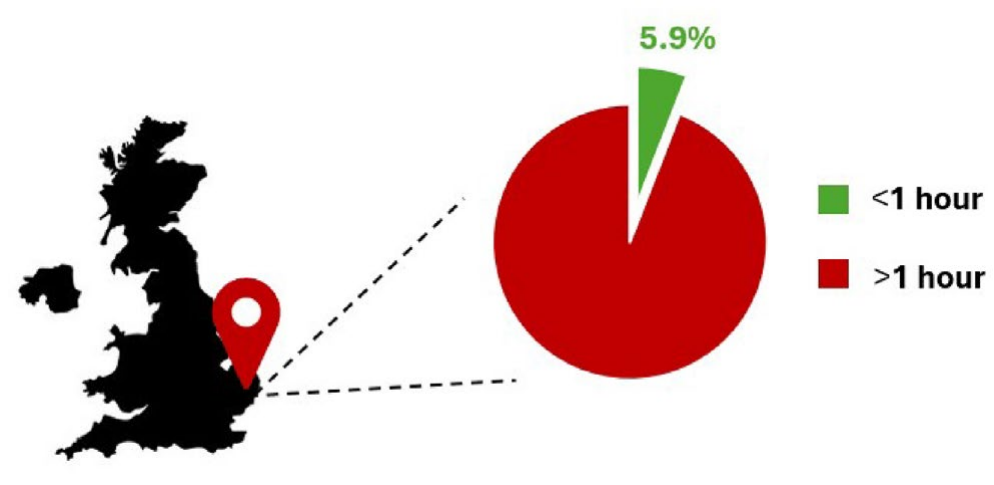
**FIGURE 1** Time From Clinical Suspicion of Meningitis to First Lumbar Puncture Attempt. Time in hours, expressed as percentage (%). Less than ().

**Conclusion:** This study emphasises that even when applying time to LP from clinical suspicion, the average consistently exceeds national guidelines. This, combined with decreased diagnostic accuracy of LP following antibiotics, may lead to continuing unnecessary therapy, longer hospital stays and increased antibiotic resistance risk. Contributing factors included shortage of trained staff, procedural challenges and unnecessary imaging. This study stresses the need for further, prospective, investigations into delays of LP's, which will translate to improved practice.

**Disclosure:** Nothing to disclose.

## OPR‐043

### Status epilepticus and stroke‐like symptoms: The role of syphilis testing in atypical presentations

#### C. del Valle‐Vargas ^1^; A. Vargas Verdaguer^1^; C. Vazquez Flores^1^; G. García Amor^1^; R. Ugena Garcia^2^; R. Benitez Diaz^3^; M. Blázquez^3^; S. Muñoz^4^; M. Lozano Sanchez^1^; C. Guerrero Castaño^1^; M. Millan Torne^1^

##### ^1^Neurology, Hospital Germans Trias i Pujol, Badalona, Spain; ^2^Reumatology, Hospital Germans Trias i Pujol, Badalona, Spain; ^3^Infectious disease, Hospital Germans Trias i Pujol, Badalona, Spain; ^4^Microbiology, Hospital Germans Trias i Pujol

**Background and Aims:** Neurosyphilis can occur at any stage of syphilis. Its diagnosis in the era of widespread penicillin use is challenging, due to its varied neurological manifestations and low suspicion index. Late‐stage neurosyphilis presenting with status epilepticus is rare, especially in our setting.

**Methods:** We present a case of late‐stage neurosyphilis presenting with general paresis, meningovascular involvement, stroke‐mimic and status epilepticus.

**Results:** A 70‐year‐old male with history of diabetes, bladder cancer, and benign prostatic hyperplasia was referred under a stroke code for sudden left hemispheric dysfunction, including hemiplegia, speech disturbance, and forced oculocephalic deviation. Neuroimaging excluded acute ischemia but revealed chronic ischemic lesions and intracranial stenoses consistent with large‐vessel vasculitis (Figure 1). Family history disclosed gait disturbances with parkinsonian features, and behavioral changes over the prior year. The patient later developed forced left oculocephalic deviation and ipsilateral facial myoclonus. EEG confirmed status epilepticus (Figure 2), which resolved with antiseizure therapy, though the patient remained aphasic with delayed responses, only single‐step command following, and frontal release signs. Cerebrospinal fluid analysis revealed mild pleocytosis (158 cells/mm^3^, 89% lymphocytes) and hyperproteinorrhachia (1.459 g/L), with negative cultures and molecular tests. A diagnosis of late‐stage neurosyphilis was made thanks to syphilis screening (RPR: 1/64; VDRL: reactive). A PET/CT scan excluded involvement of other organs. IV penicillin and corticosteroids were initiated, resulting in a favorable recovery to baseline.

**Figure:**
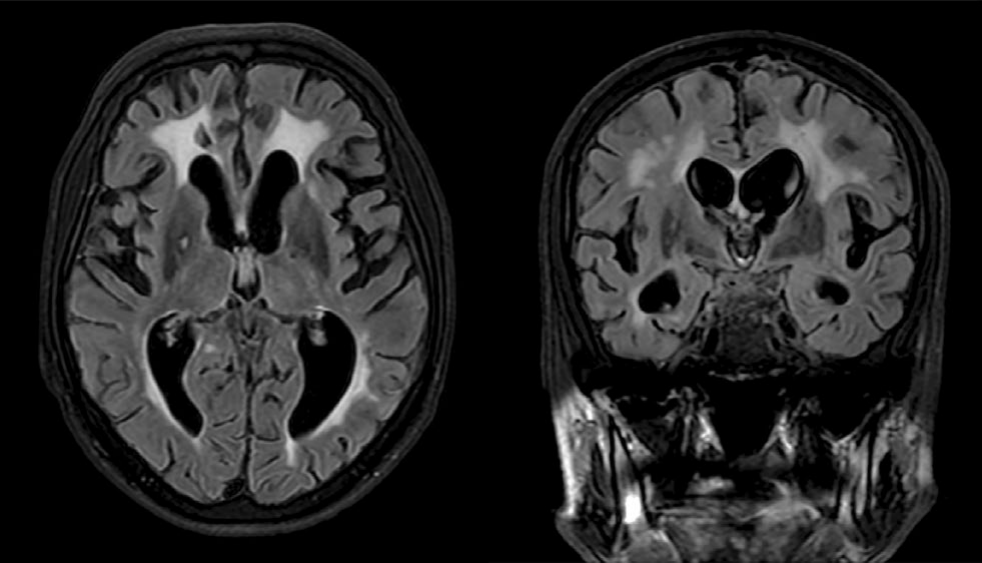
**FIGURE 1** Brain MRI severe brain atrophy, mainly hippocampus and temporal lobes. Also hydrocephalus of chronic characteristics, with confluent T2 hyperintensity affecting the periventricular white matter, related to demyelination of probable chronic hypoxic origin

**Figure:**
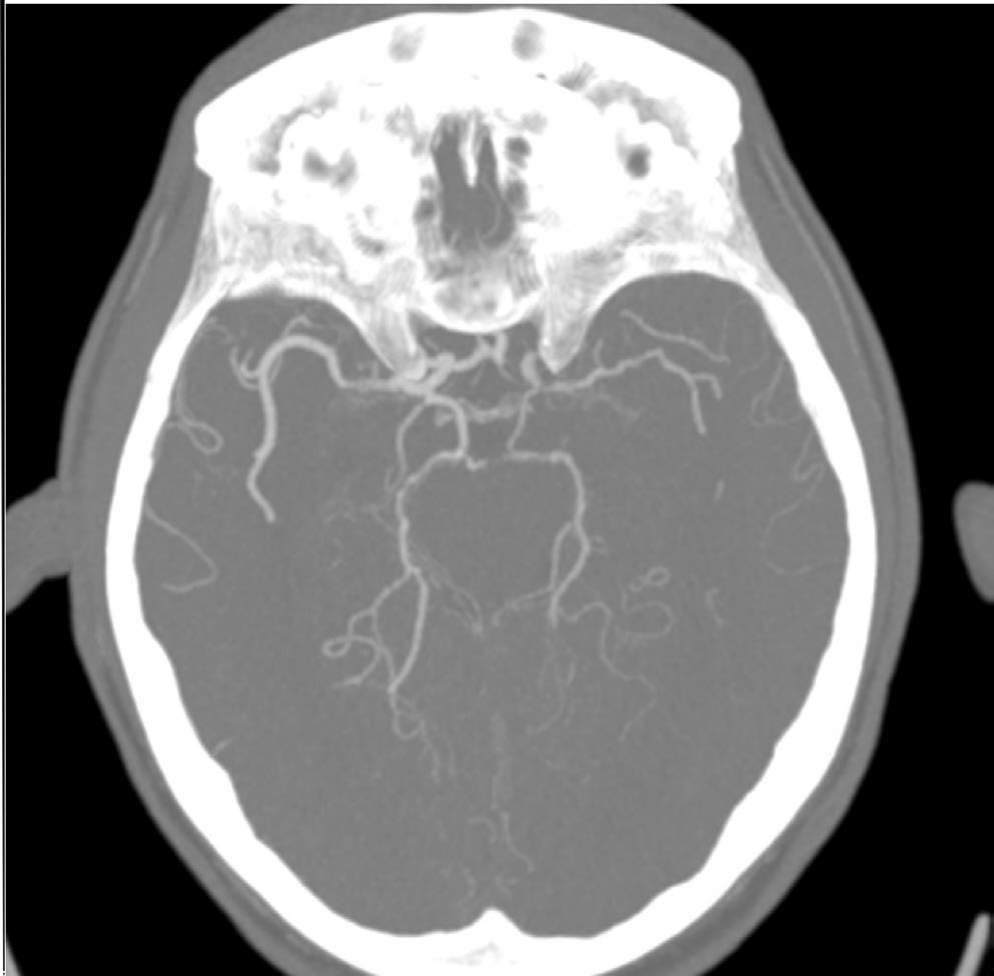
**FIGURE 2** CT angiography showed multifocal intracranial arterial narrowing and beading, involving both the anterior and posterior circulation.

**Figure:**
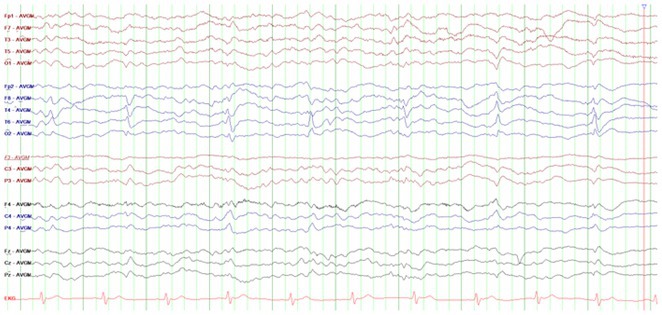
**FIGURE 3** Initial EEG with periodic lateralized discharges (LPDs) in the form of sharp waves in the right posterior quadrant (right occipitotemporoparietal region).

**Conclusion:** Although rare, neurosyphilis should be considered in unexplained status epilepticus or stroke, particularly if preceded by meningitis or encephalitis symptoms and lacking traditional risk factors, to ensure timely diagnosis and effective treatment.

**Disclosure:** Nothing to disclose.

## OPR‐044

### Predictors of cerebral complications in pediatric tuberculous meningitis patients: A systematic review and meta‐analysis

#### E. Bittencurt Thomaz de Assis ^1^; Otero de Melo dos Reis^2^; H. Kalaiarasan Swamy^1^; K. Amorim Alves^2^; A. Stepanov Nikolaevich^1^; A. Turdieva^1^; J. Papaterra Limongi^3^

##### ^1^Baltic Federal University, Kaliningra, Russian Federation; ^2^Universidad Nacional de Rosario, Rosario, Argentina; ^3^Universidade de Sao Paulo, Sao Paulo, Brazil

**Background and Aims:** This systematic review and meta‐analysis included three studies with four groups analyzing BTK inhibitors for RMS. A comprehensive search of PubMed, Embase, and Cochrane databases was conducted following PRISMA guidelines. Risk of bias was assessed, and a meta‐analysis was performed using Review Manager 4.1.

**Methods:** This systematic review and meta‐analysis included 13 studies focusing on three key categories: molecular, clinical, and imaging factors. A comprehensive search of PubMed, Embase, and Cochrane databases was conducted following PRISMA guidelines. Risk of bias was assessed, and a meta‐analysis was performed using Review Manager 4.1.

**Results:** A total of 2,336 pediatric patients were included. Stage III TBM was a strong predictor of adverse outcomes (OR 3.27; 95% CI 1.56–6.83; *I* ^2^ 85%; *p* = 0.002). Hydrocephalus also increased risk (OR 1.57; CI 95% 1.06–2.33; *I* ^2^ 35%; *p* = 0.02), especially non‐communicating hydrocephalus. Glasgow Coma Scale (GCS) scores <7 were seen in 30–50% of patients with complications, correlating with stroke risk. Elevated inflammatory markers like IL‐6, TNF‐α, and CSF protein >1 g/L were associated with higher complications, while CSF glucose <40 mg/dL was significantly lower in affected patients. Clinical manifestations, such as hemiparesis or hemiplegia, were observed in 45% to 64% of patients with cerebrovascular complications. Lastly, malnutrition was identified as a critical factor influencing prognosis, whereas a shorter illness duration ( < 1 month) was found to exert a protective effect.

**Figure:**
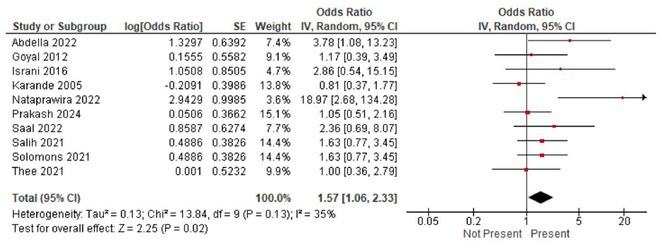
**FIGURE 1** Hydrocephalus

**Figure:**
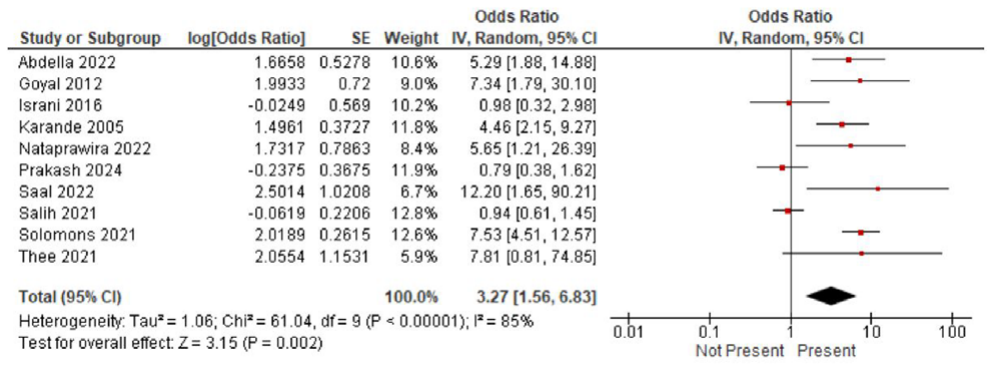
**FIGURE 2** TBM Stage III

**Conclusion:** This review highlights molecular and clinical predictors of cerebrovascular complications in pediatric TBM, emphasizing their role in improving early diagnosis and guiding targeted management to enhance outcomes.

**Disclosure:** Nothing to disclose.

## OPR‐045

### Unmasking undiagnosed HIV through cerebral toxoplasmosis: Two case illustrations

#### R. Iatan ^1^; I. Somoiag^1^; R. Badea^2^; C. Tiu^2^

##### ^1^Department of Neurology, University Emergency Hospital, Bucharest, Romania; ^2^“Carol Davila” University of Medicine and Pharmacy, Neurology Department, Bucharest, Romania

**Background and Aims:** Cerebral toxoplasmosis is one of the most frequently reported opportunistic central nervous system (CNS) infections in individuals with advanced HIV/AIDS. Despite advances in antiretroviral therapy, missed or delayed HIV diagnoses can result in life‐threatening presentations of toxoplasmosis.

**Methods:** We retrospectively reviewed the clinical, radiological, and laboratory data of two patients who presented to our institution with acute neurological deficits and were subsequently diagnosed with HIV‐related cerebral toxoplasmosis. Diagnostic evaluations included computed tomography (CT), magnetic resonance imaging (MRI), comprehensive serological panels, and targeted therapeutic interventions.

**Results:** A 47‐year‐old woman with a three‐month history of fever, night sweats, and weight loss presented with acute mental status changes, global aphasia, and mild right hemiparesis. Neuroimaging revealed multiple contrast‐enhancing lesions, prompting suspicion of metastatic disease or an infectious etiology. Serology confirmed newly diagnosed HIV and markedly elevated Toxoplasma gondii IgG titers. Despite prompt high‐dose trimethoprim‐sulfamethoxazole and corticosteroid treatment, her condition deteriorated rapidly, culminating in multisystem organ failure and death. A 50‐year‐old man presented with progressive cognitive impairment and mild tetraparesis. Initially presumed to have hemorrhagic metastases, he was later found to have uncontrolled HIV infection. Strongly positive T. gondii serologies and characteristic ring‐enhancing lesions on magnetic resonance imaging established the diagnosis of neurotoxoplasmosis. Targeted antiparasitic therapy led to partial neurological improvement.

**Figure:**
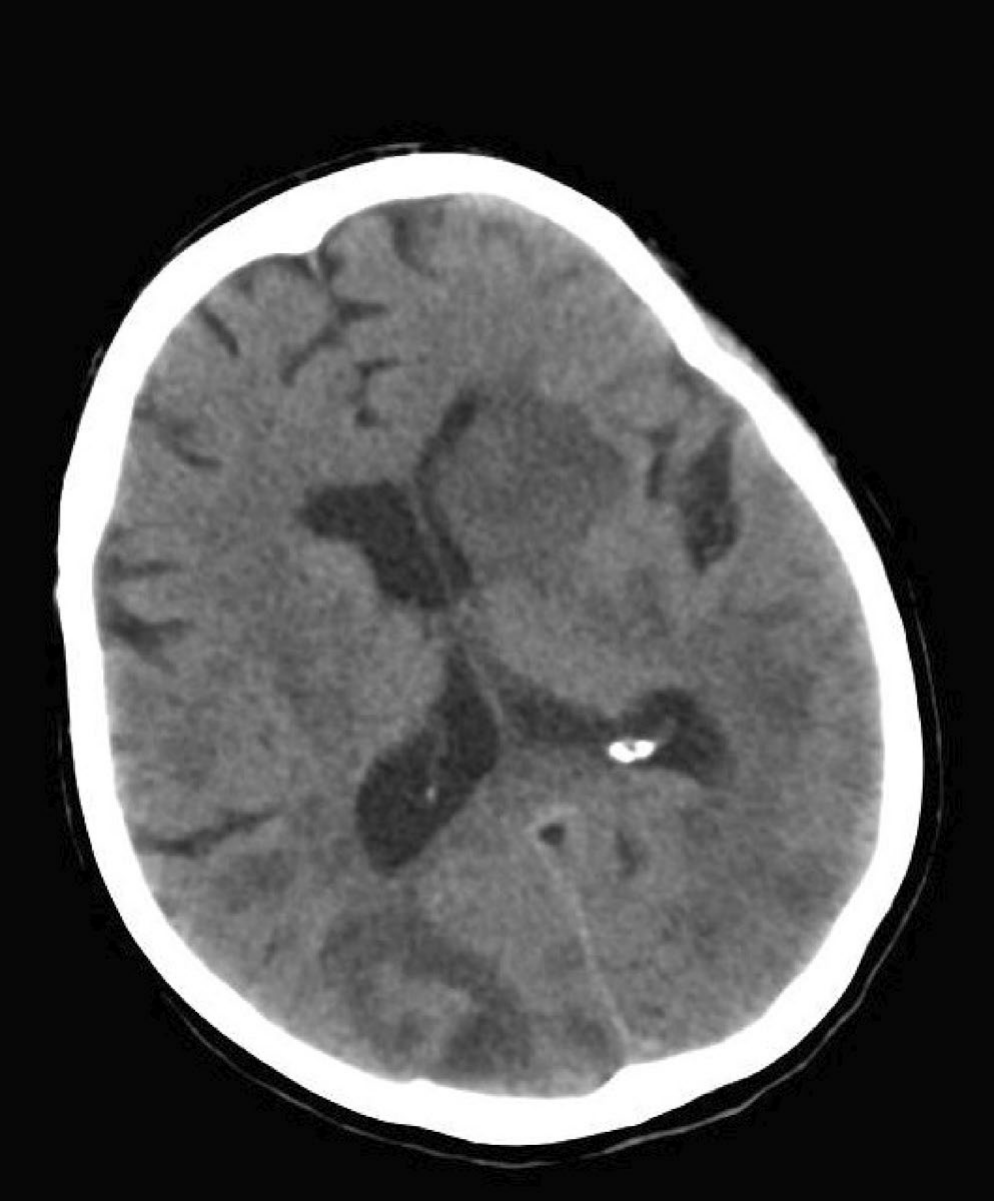
**FIGURE 1** CASE 1 Noncontrast enhanced computed tomography demonstrating multiple hypodense lesions, most prominent in the left basal ganglia and right parietal lobe.

**Figure:**
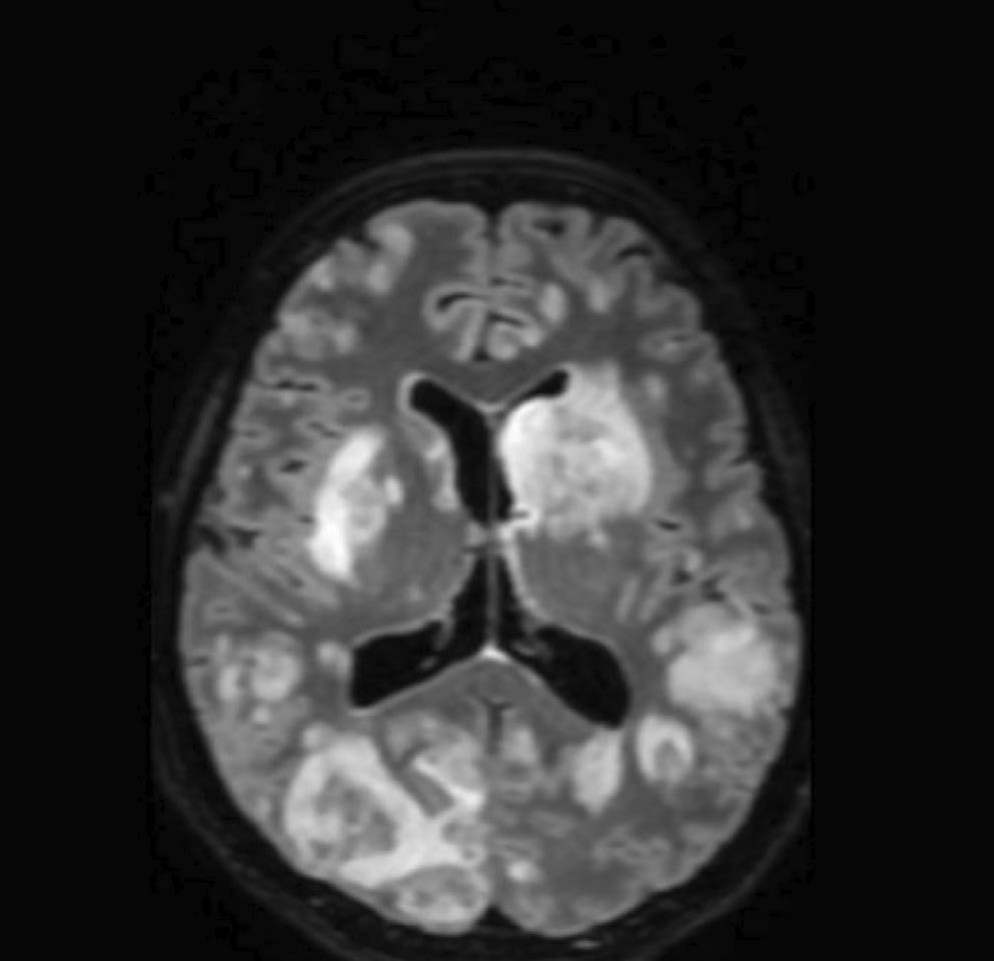
**FIGURE 2** CASE 1 Multiple FLAIR‐hyperintense lesions, with the concentric target sign seen in the deep parenchymal lesions.

**Figure:**
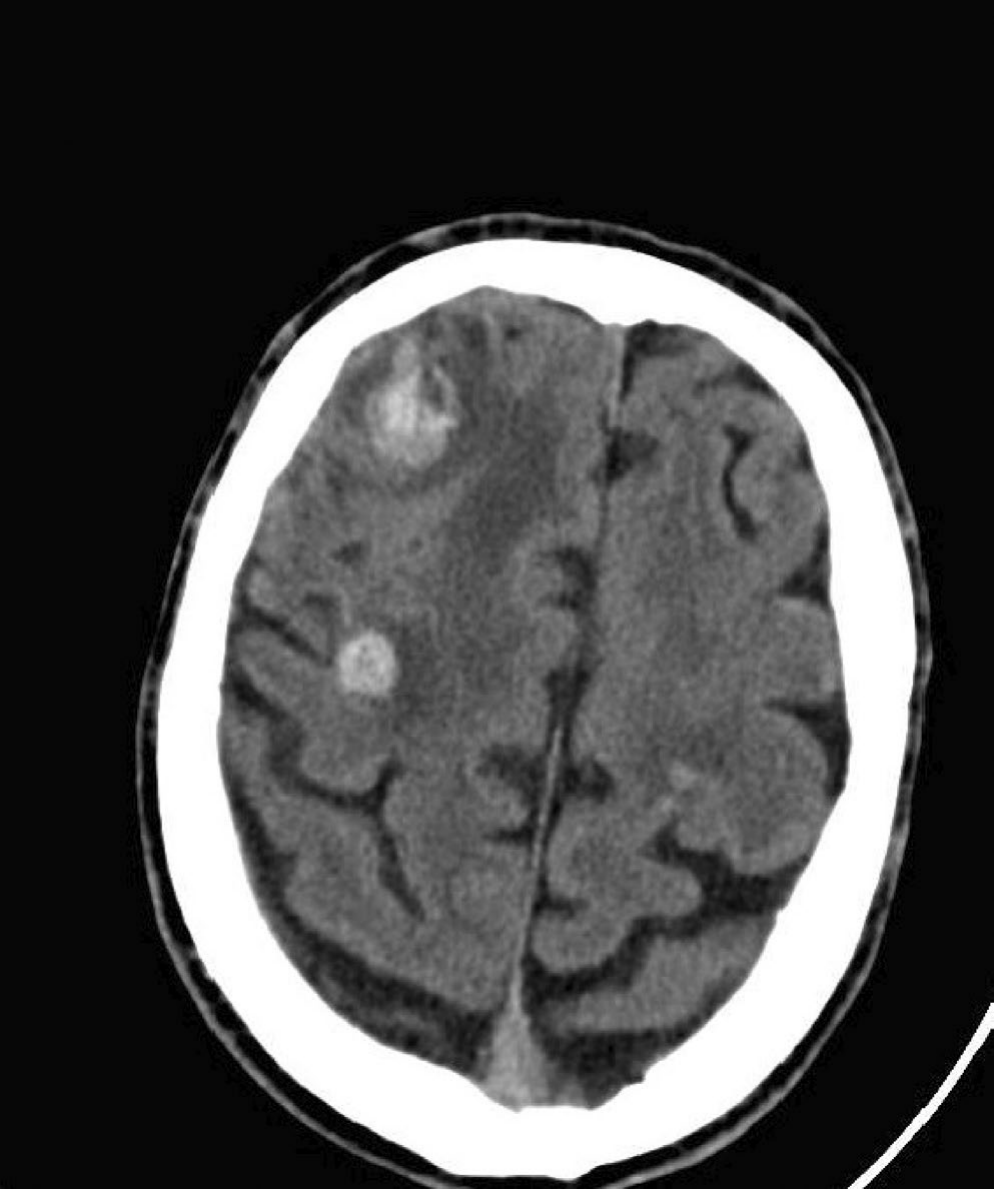
**FIGURE 3** CASE 2 Noncontrast enhanced computed tomography revealing several intraparenchymal hyperdense rounded lesions and surrounding vasogenic edema.

**Conclusion:** These cases illustrate the diagnostic complexity and potentially fulminant course of cerebral toxoplasmosis in the setting of undisclosed or undertreated HIV/AIDS. Early identification of immunosuppression, prompt neuroimaging, and serological testing are paramount to initiate life‐saving therapy and mitigate the risk of severe neurological sequelae.

**Disclosure:** Nothing to disclose.

## OPR‐046

### The hidden face of tuberculosis: A retrospective study of central nervous system manifestations in Nepal

#### S. Joshu ^1^; S. Kharel^1^; R. Ojha^2^; B. Gajurel^2^

##### ^1^Maharajgunj Medical Campus, Institute of Medicine, Kathmandu, Nepal; ^2^Department of Neurology, Tribhuvan University Teaching Hospital, Kirtipur, Nepal

**Background and Aims:** Mycobacterium tuberculosis is the cause of (CNS‐TB), a group of neurological syndromes with a comparatively high death and morbidity rate. Meningitis is the most frequent symptom of CNS‐TB, followed by tuberculoma, tuberculous brain abscess, and Pott's illness. About 1% of cases of tuberculosis are attributed to CNS‐TB. The objective is to know the clinico‐demographic profile of patients with central nervous system tuberculosis (CNS TB) along with their hospital outcomes.

**Methods:** This is a single‐centered retrospective study conducted among adult CNS‐TB patients in our center over one year.

**Results:** A total of 61 patients (57.4% males) were diagnosed with CNS TB with a mean age of 42.10 (16.96) years. The majority were TB meningitis (55.7%) followed by tuberculoma 13 (21.3%) with few cases of tubercular abscess and spinal TB. Common presenting symptoms included fever (62.9%), headache (73.3%), weight loss (31.1%), vomiting (49.1%), seizures (26.2%), altered mental status (47.5%), and only a few cases of facial deviation and of visual loss. On examinations, neck stiffness was positive at 26.2%, and focal neurological deficit was found at 32.8%. Bacteriological and radiological imaging were done. The majority were treated with ATT of which three had side effects like ATT‐induced hepatitis and ethambutol toxicity and only seven hydrocephalus cases had a shunt surgery done. The majority were discharged (86.9%) while 16.4% of cases were intubated and one case had mortality.

**Conclusion:** Early diagnosis through various investigations and appropriate management strategy is the cornerstone for the treatment of CNS‐TB. More multi‐center studies focusing on larger sample sizes are necessary.

**Disclosure:** Nothing to disclose.

## Movement Disorders 1

## OPR‐047

### The language of gait: Interpreting emotional states through gait features

#### E. Canu ^1^; M. Putzolu^2^; E. Sarasso^3^; A. Gardoni^1^; E. Ravizzotti^4^; S. Mezzarobba^4^; S. Basaia^1^; L. Avanzino^5^; F. Agosta^6^; M. Filippi^7^; E. Pelosin^8^

##### ^1^Neuroimaging Research Unit, Division of Neuroscience, IRCCS San Raffaele Scientific Institute, Milan, Italy; ^2^Department of Experimental Medicine, Section of Human Physiology, University of Genoa, Genoa, Italy; ^3^Neuroimaging Research Unit, Division of Neuroscience, IRCCS San Raffaele Scientific Institute, Milan, Italy; and DINOGMI, University of Genoa, Genoa, Italy; ^4^Department of Neuroscience, Rehabilitation, Ophthalmology, Genetics and Maternal Child Health, University of Genoa, Genoa, Italy; ^5^IRCCS Ospedale Policlinico San Martino, and Department of Experimental Medicine, Section of Human Physiology, University of Genoa, Genoa, Italy; ^6^Neuroimaging Research Unit, Division of Neuroscience, and Neurology Unit, IRCCS San Raffaele Scientific Institute, and Vita‐Salute San Raffaele University, Milan, Italy; ^7^Neurology Unit, Neurorehabilitation Unit, Neurophysiology Service, and Neuroimaging Research Unit, Division of Neuroscience, IRCCS San Raffaele Scientific Institute, and Vita‐Salute San Raffaele University, Milan, Italy; ^8^Department of Neuroscience, Rehabilitation, Ophthalmology, Genetics and Maternal Child Health, University of Genoa, and IRCCS Ospedale Policlinico San Martino, Genoa, Italy

**Background and Aims:** The complex interplay between gait alterations and emotions in Parkinson's disease (PD) requires further investigation. This study aimed at investigating whether the observation of emotional gait conditions can modulate spatio‐temporal gait parameters and gait‐related functional brain correlates in healthy subjects (HC) and PD patients by evoking those emotions.

**Methods:** We first administered a questionnaire containing videos of an actress walking with different gait patterns according to specific emotions (e.g. happiness, sadness, fear/anxiety and neutral) in order to select the videos with the mostly recognized emotional gait patterns in a cohort of 110 HC. Then, we administered the selected videos to 19 HC and 21 PD, which were asked to imitate the emotional gait patterns observed in the videos and to report the intensity and valence of the evoked emotions. The spatio‐temporal gait parameters were monitored using six inertial sensors. All subjects observed the same videos during a functional MRI (fMRI) task in order to obtain neural correlates of emotional gait observation.

**Results:** In both HC and PD, happiness promoted an improvement in gait kinematics (e.g., increased stride length, turn velocity, upper limb and trunk movement amplitude) and an enhanced recruitment of the sensorimotor network during the fMRI task in PD. Sadness and anxiety were associated with a worsening of spatiotemporal gait parameters and to an extensive reduction of fMRI activity of sensorimotor areas, mirror neuron system and cerebellum.

**Conclusion:** This study suggests that positive and negative emotions specifically influence gait kinematics and fMRI activity of the sensorimotor system.

**Disclosure:** MP, AG ER, SM, LA, EP nothing to disclose. ES, EC, SB received grants from the Italian Ministry of Health. FA is Associate Editor of NeuroImage: Clinical, has received speaker honoraria from Biogen Idec, Italfarmaco, Roche, Zambon and Eli Lilly, and receives or has received research supports from the Italian Ministry of Health, the Italian Ministry of University and Research, AriSLA (Fondazione Italiana di Ricerca per la SLA), the European Research Council, the EU Joint Programme—Neurodegenerative Disease Research (JPND) and Foundation Research on Alzheimer Disease (France). MF is Editor‐in‐Chief of the Journal of Neurology, Associate Editor of Human Brain Mapping, Neurological Sciences, and Radiology; received compensation for consulting services from Alexion, Almirall, Biogen, Merck, Novartis, Roche, Sanofi; speaking activities from Bayer, Biogen, Cel‐ gene, Chiesi Italia SpA, Eli Lilly, Genzyme, Janssen, Merck‐Serono, Neo‐ pharmed Gentili, Novartis, Novo Nordisk, Roche, Sanofi, Takeda and TEVA; participation in Advisory Boards for Alexion, Biogen, Bristol‐Myers Squibb, Merck, Novartis, Roche, Sanofi, Sanofi‐Aventis, Sanofi‐Genzyme, Takeda; scientific direction of educational events for Biogen, Merck, Roche, Celgene, Bristol‐Myers Squibb, Lilly, Novartis, Sanofi‐Genzyme; he receives research support from Biogen Idec, Merck‐Serono, Novartis, Roche, the Italian Ministry of Health, the Italian Ministry of University and Research and Fondazione Italiana Sclerosi Multipla.

## OPR‐048

### Bridging pixel precision and clinical intuition: Quantifying ‘movement disorders phenomenology’ with 2D pose estimation

#### I. Varela ^1^; U. Serratos Hernández^2^; N. Sirmpilatze^3^; S. Miñano^3^; K. Sampson^2^; P. Termsarasab^5^; S. Frucht^4^; A. Sadnicka^1^

##### ^1^Gatsby Computational Neuroscience Unit, London, UK; ^2^Institute of Neurology, University College London, UK; ^3^Sainsbury Wellcome Center, London, UK; ^4^Langone Health, New York University, USA; ^5^Department of Medicine, Mahidol University, Salaya Thailand

**Background and Aims:** Identifying phenomenology in movement disorders is a core step in patient management. However, semantic definitions can be ambiguous, and clinical scales are often subjective and incompletely capture movement [1,2]. Human pose estimation offers objective motion capture with the potential to develop a set of tools that can complement existing clinical expertise. We have developed a pipeline for extracting quantifiable metrics from clinical recordings, focusing on hyperkinetic disorders detailed in 'Phenomenology in Movement Disorders' [3].

**Methods:** Multiple pose estimation applications were compared and MMPose had the highest‐performing models for this context [4]. We analysed all videos featuring hyperkinetic movement disorders and after applying clinical and technical inclusion/exclusion criteria 1176 segments were extracted from the 650 source videos resulting in 2h37m of content. 2D pose estimation was implemented and post‐processing ensured keypoint reliability.

**Figure:**
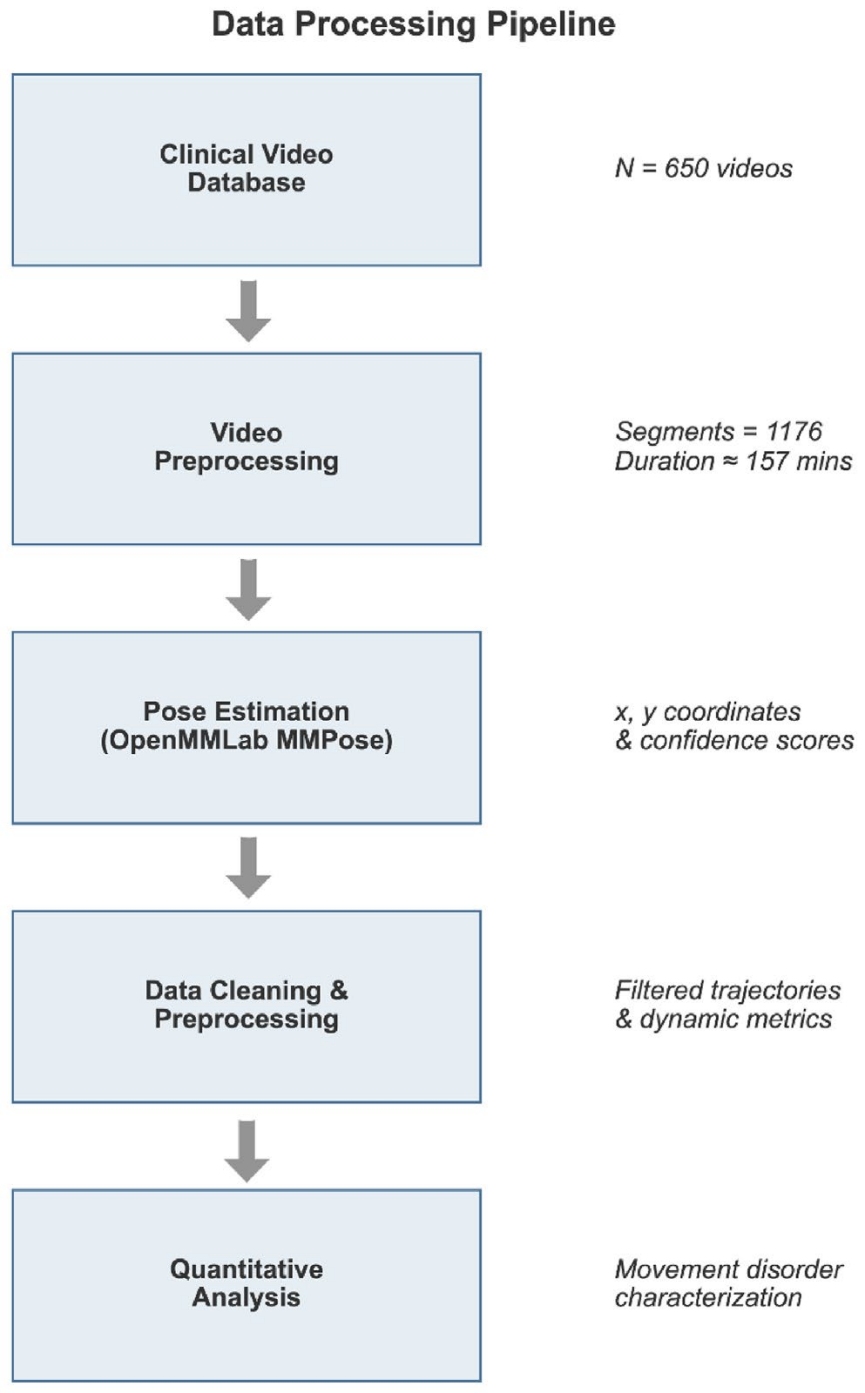
**FIGURE 1** Analysis pipeline transforming clinical videos into quantifiable movement metrics. Key steps include video preprocessing, pose estimation with MMPose, trajectory filtering, and disorder‐specific analysis.

**Results:** Our curation yielded structured data across five phenotypes: chorea (*n* = 119), dystonia (*n* = 261), myoclonus (*n* = 228), tics (*n* = 211), and tremor (*n* = 357). Initial analysis demonstrates phenotype‐specific signatures: tremor exhibited characteristic frequency peaks, chorea showed shifting generalised dyskinetic patterns, and tics displayed spatial‐temporal similarity.

**Figure:**
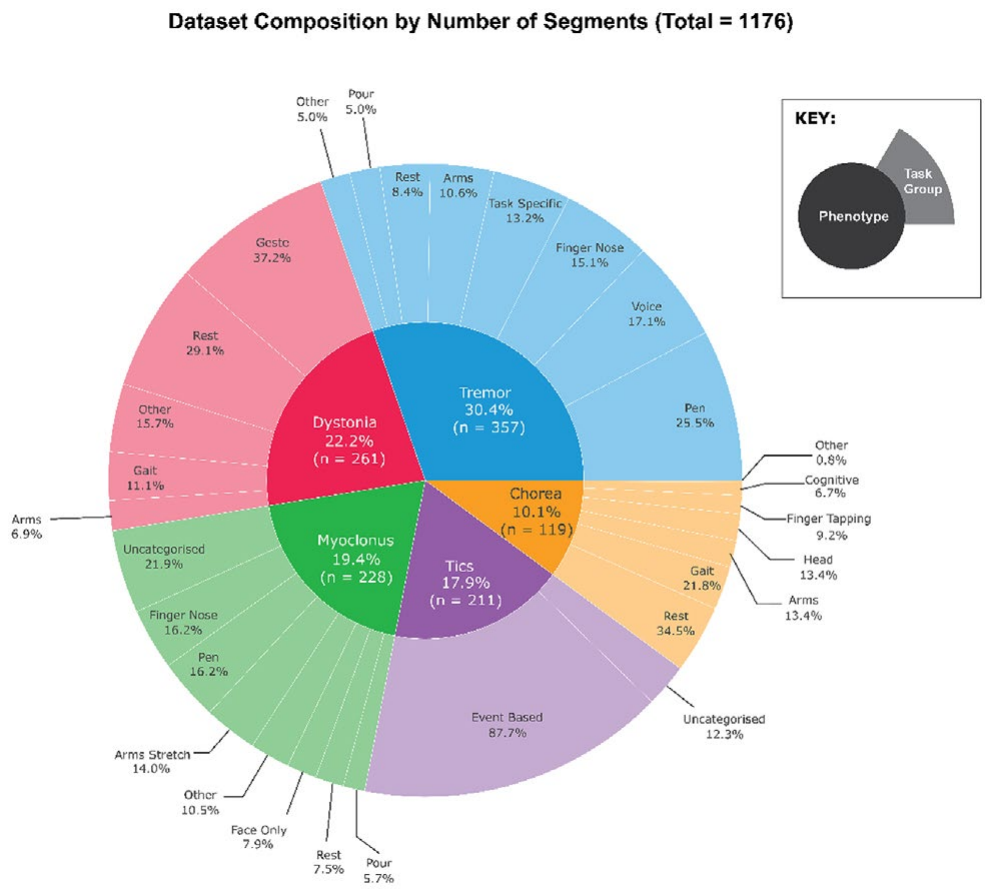
**FIGURE 2** Data composition by segment number showing phenomenology categories (inner ring) and breakdown by tasks (outer ring). ‘Uncategorised’ indicates not fitting directories for other clinical examination tasks or actions. ‘Other’ groups categories with >5%.

**Figure:**
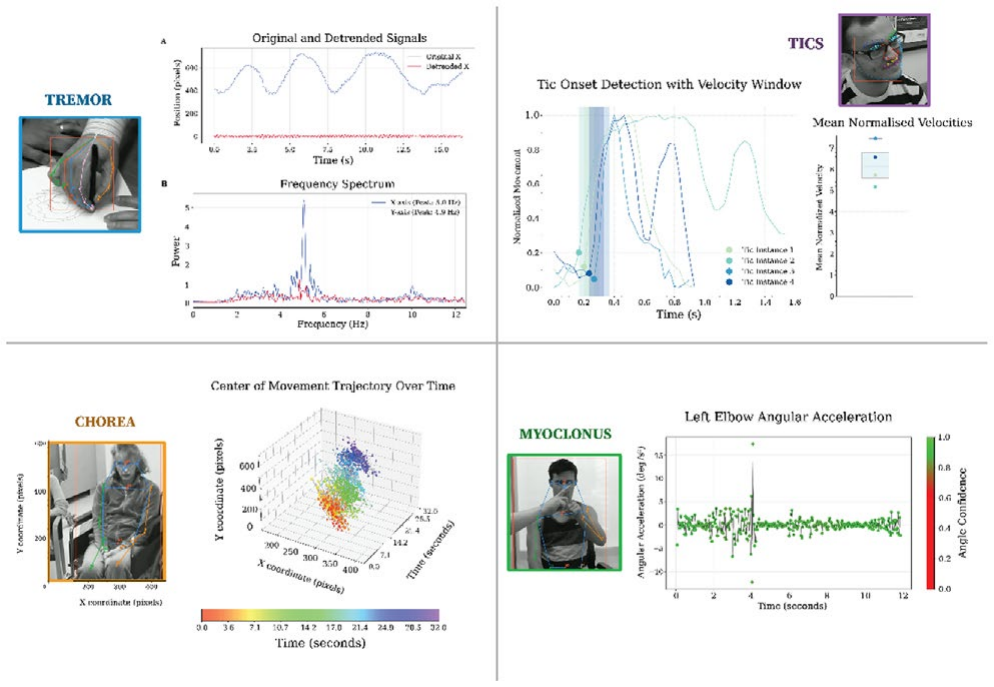
**FIGURE 3** Representative analysis of phenotype movement patterns: tremor frequency analysis showing 4–5 Hz peaks, tic onset velocity profiles, chorea center‐of‐movement trajectory showing dispersed movement progression, and myoclonus angular acceleration spikes.

**Conclusion:** This proof‐of‐concept application establishes a framework for automated movement pattern analysis with ongoing work exploring the reliability and specificity of different kinematic features across phenotypes. 2D pose estimation can bridge the gap between subjective clinical assessment and objective movement quantification. Beyond improving diagnostic precision, this methodology offers new possibilities for treatment monitoring and research in movement disorders.

**Disclosure:** Nothing to disclose.

## OPR‐049

### Concordance of imaging and clinical based STN‐DBS programming improves motor outcomes of directional stimulation in PD

#### L. Rigon ^1^; F. Bove^2^; A. Izzo^3^; N. Montano^3^; L. Brusa^4^; R. Cerroni^5^; A. De Biase^2^; L. Di Biase^6^; G. D'Alessandris^3^; D. Genovese^2^; P. Pecoraro^6^; A. Peppe^7^; M. Rizzo^8^; A. Stefani^5^; A. Suppa^9^; A. Bentivoglio^2^; P. Calabresi^2^; C. Piano^2^

##### ^1^IRCCS San Camillo Hospital, Venice, Italy; ^2^Department of Neuroscience, Università Cattolica del Sacro Cuore, Rome, Italy; ^3^Neurosurgery Unit, Fondazione Policlinico Universitario A. Gemelli IRCCS, Rome, Italy; ^4^Neurology Unit, S. Eugenio Hospital, Rome, Italy; ^5^Department of System Medicine, UOSD Parkinson, University of Rome Tor Vergata, Rome, Italy; ^6^Operative Research Unit of Neurology, Fondazione Policlinico Universitario Campus Bio‐Medico, Via Alvaro del Portillo, 200 ‐ 00128 Roma, Italy; ^7^IRCCS Santa Lucia Foundation, Rome, Italy; ^8^Neurology Unit, Azienda Ospedaliera Ospedali Riuniti Villa Sofia e Cervello, Palermo, Italy; ^9^Department of Human Neurosciences, Sapienza University of Rome, Rome, Italy

**Background and Aims:** Advances in STN‐DBS technology, among which directional stimulation, improved Parkinson's disease (PD) treatment efficacy, while increasing the clinical programming complexity. Lead localization software may aid the stimulation contact selection process. We aimed to assess the concordance between imaging‐suggested (IGP) and conventional‐programming (CP) selected stimulation contacts one year after surgery and its impact on motor outcomes.

**Methods:** Sixty‐four PD patients with bilateral STN‐DBS were enrolled. Lead localization was reconstructed with BrainlabTM software. For each electrode, the vertical contact level and, when applicable, the directionality predicted by the lead reconstruction software to be the most effective were established and compared to the stimulation parameters clinically activated one year post‐surgery. IGP/CP concordance ratio was calculated for both stimulation level and directional contacts. Post‐operative modifications of PD motor symptoms severity were compared among groups of concordant and discordant IGP/CP programming.

**Figure:**
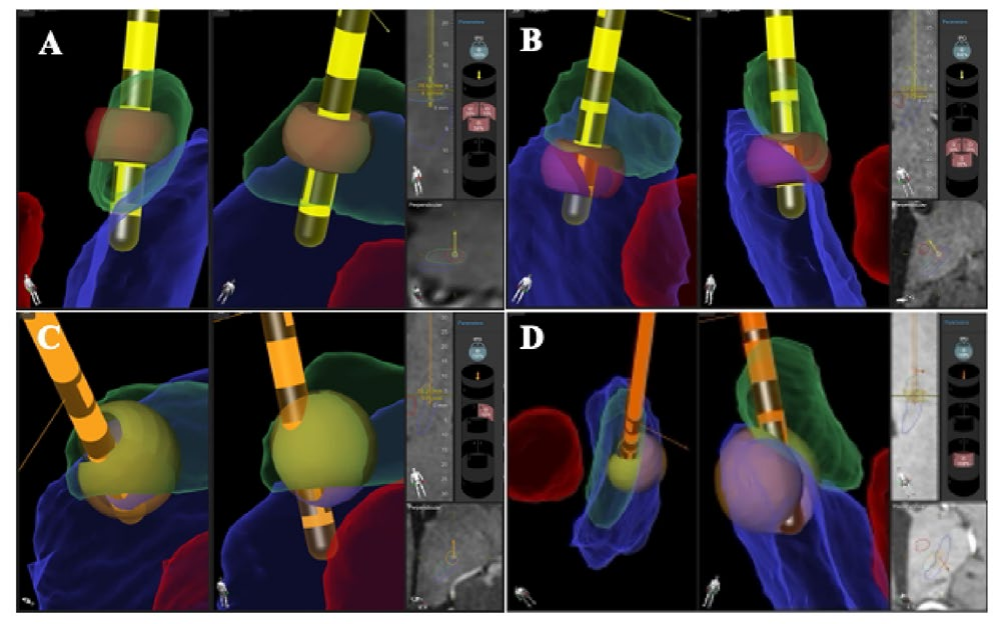
**FIGURE 1** Examples of assessments of level contact and directionality CP/IGP concordance from our cohort. A: CP/IGP concordant contact level; B: CP/IGP discordant contact level; C: CP/IGP concordant directionality; D: CP/IGP discordant directionality.

**Results:** One‐year post‐surgery, IGP/CP concordance was 80% for active stimulation vertical contact level and 51% for directionality. No significant difference in motor outcomes was found between IGP/CP concordant and discordant patients for contact level activation, whereas patients with concordant IGP/CP active directional stimulation (c‐Direction) showed superior motor outcomes at one‐year follow‐up than those discordant (d‐Direction) (UPDRS‐III stimulation‐induced improvement: c‐Direction = −25.66±13.74 vs d‐Direction = −12.54±11.86; *p* = 0,011).

**Figure:**
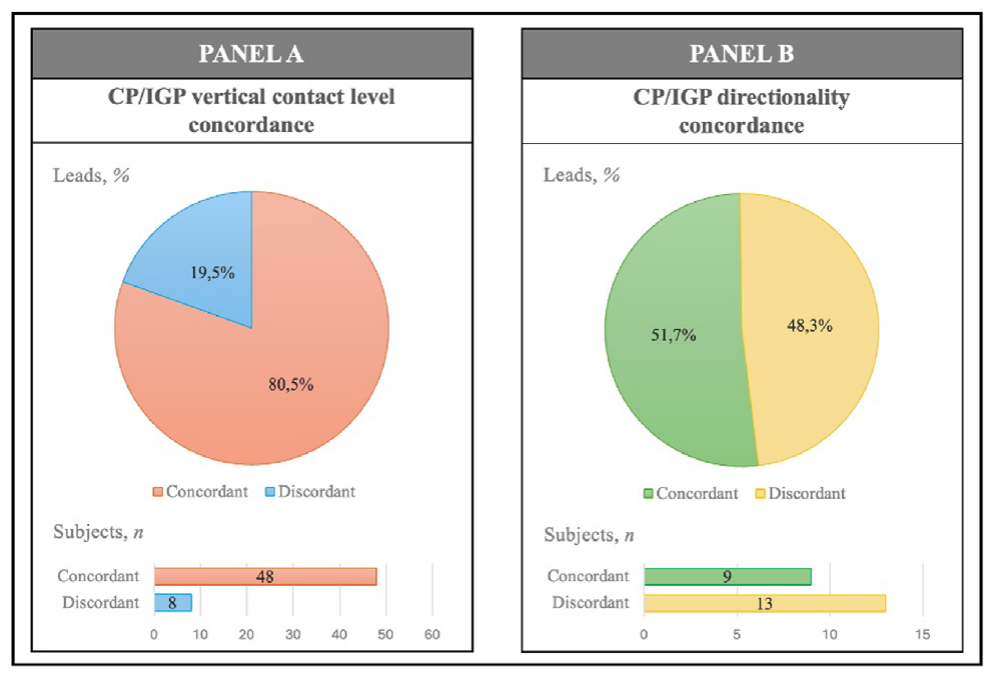
**FIGURE 2** CP/IGP concordance analysis for vertical contact level (panel A) and directionality (panel B). In each panel, CP/IGP concordance regarding leads is displayed on top while that regarding subjects is depicted on the bottom.

**Figure:**
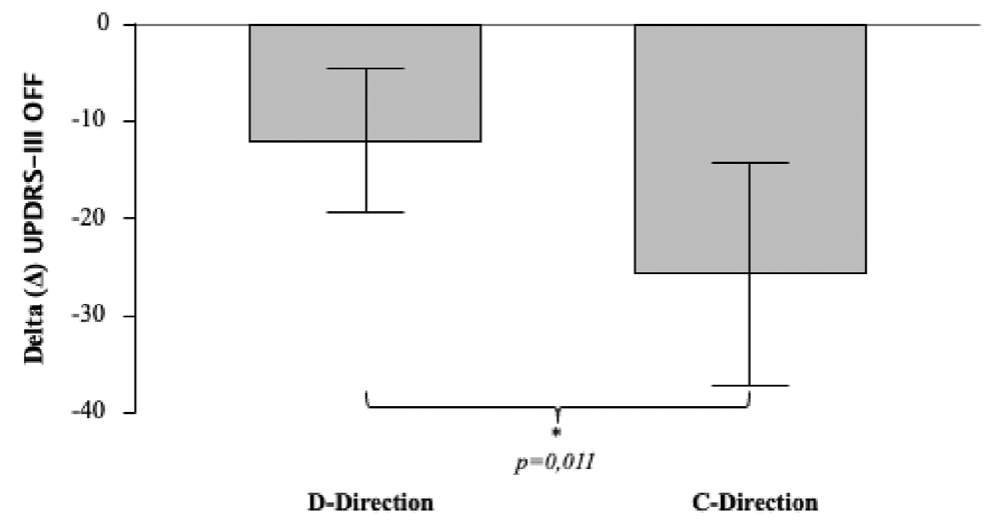
**FIGURE 3** Motor outcome comparison per directionality concordance. Stimulation‐induced motor improvement is expressed as Delta (Δ) UPDRS‐III OFF = (post‐operative UPDRS‐III ONstim/OFFmed) ‐ (preoperative UPDRS‐III OFFmed).

**Conclusion:** Visual reconstruction software correctly predicted the most clinically effective stimulation contact levels in most patients. Imaging therefore facilitates classic STN‐DBS clinical programming while assuring similar outcomes. Moreover, better motor outcomes were reached by patients with concordant IGP/CP directional parameters, suggesting that visualization can represent an added value in particular for directional stimulation programming.

**Disclosure:** Nothing to disclose.

## OPR‐050

### Elucidating the pathophysiology of GBA‐PD using functional brain connectome

#### S. Basaia ^1^; T. Cusolito^2^; E. Sarasso^3^; E. Sibilla^1^; E. Canu^1^; R. Balestrino^4^; T. Stojkovic^5^; I. Stankovic^5^; A. Tomic^5^; V. Markovic^5^; E. Stefanova^5^; F. Molinari^2^; V. Kostic^5^; F. Agosta^6^; M. Filippi^7^

##### ^1^Neuroimaging Research Unit, Division of Neuroscience, IRCCS San Raffaele Scientific Institute, Milan, Italy; ^2^Biolab, PoliTo(BIO)Med Lab, Department of Electronics and Telecommunications, Politecnico di Torino, Torino, Italy; ^3^Neuroimaging Research Unit, Division of Neuroscience, and Department of Rehabilitation and Functional Recovery, IRCCS San Raffaele Scientific Institute, Milan, Italy; and DINOGMI, University of Genoa, Genoa, Italy; ^4^Neurology Unit, IRCCS San Raffaele Scientific Institute, Milan, Italy; ^5^Clinic of Neurology, Faculty of Medicine, University of Belgrade, Belgrade, Serbia; ^6^Neuroimaging Research Unit, Division of Neuroscience, and Neurology Unit, IRCCS San Raffaele Scientific Institute, and Vita‐Salute San Raffaele University, Milan, Italy; ^7^Neurology Unit, Neurorehabilitation Unit, Neurophysiology Service, and Neuroimaging Research Unit, Division of Neuroscience, IRCCS San Raffaele Scientific Institute, and Vita‐Salute San Raffaele University, Milan, Italy

**Background and Aims:** To investigate functional brain network alterations in Parkinson's disease (PD) subjects carrying glucocerebrosidase (GBA) mutation (GBA‐positive) and PD non‐carriers (GBA‐negative) using graph analysis and connectomics.

**Methods:** Thirteen GBA‐positive, 39 GBA‐negative PD patients and 60 age‐ and sex‐matched controls underwent clinical evaluation, 3DT1‐weighted and resting‐state functional MRI (rs‐fMRI). Functional connectome for each subject was obtained from rs‐fMRI scans as the Pearson's correlation coefficient between time‐series in 83 cortical and subcortical brain regions identified by the Desikan Atlas. Graph analysis and connectomics assessed global and local functional topologic network properties (betweenness centrality, nodal degree, nodal strength, mean distance and path length), and regional functional connectivity using Network Based Statistics (NBS). All analyses were adjusted for age, sex, and the UPDRS‐III score (within the patients' group only).

**Results:** Relative to controls, GBA‐positive PD patients showed severe global functional network alterations (lower betweenness centrality and nodal degree), while GBA‐negative patients showed relatively preserved functional brain architecture. GBA‐positive patients demonstrated reduced graph analysis measures in frontal, sensorimotor, parietal and temporal areas relative to controls, and in occipital areas relative to GBA‐negative PD patients. Those patients showed only decreased connectivity in the right frontal lobe relative to controls. Considering NBS analysis, functional connectivity breakdown in the parietal lobe, left temporal, and occipital, sensorimotor, and right frontal differentiated GBA‐positive PD patients from controls (*p* = 0.04).

**Conclusion:** Functional graph analysis and connectome measures may be a useful tool for monitoring and predicting PD progression in accordance with the genetic background.

**Disclosure:** Funding. Ministry of Education, Science, and Technological Development, Republic of Serbia [grant 175090] and Italian Ministry of Health [grant RF‐2018‐12366746]. Disclosures. SB, ES, EC received grants from the Italian Ministry of Health. TC, ES, RB, IS, AT, FM nothing to disclose. TS received speaker honoraria from Actavis and Alzheimer's Association International Research Grant. ES received speaker honoraria from Actavis. VSK received speaker honoraria from Actavis and Solveo. FA received speaker honoraria from Biogen Idec, Roche, Eli Lilly and GE Healthcare; and grants from Italian Ministry of Health, Italian Ministry of University and Research, AriSLA, European Research Council, EU Joint Programme—Neurodegenerative Disease Research, and Foundation Research on Alzheimer Disease (France). MF received compensation for consulting services or speaking activities from Alexion, Almirall, Bayer, Biogen, Celgene, Chiesi Italia SpA, Eli Lilly, Genzyme, Janssen, Merck‐Serono, Neopharmed Gentili, Novartis, Novo Nordisk, Roche, Sanofi Takeda, and TEVA; Advisory Boards for Alexion, Biogen, Bristol‐Myers Squibb, Merck, Novartis, Roche, Sanofi, Sanofi‐Aventis, Sanofi‐Genzyme, Takeda; scientific direction of educational events for Biogen, Merck, Roche, Celgene, Bristol‐Myers Squibb, Lilly, Novartis, Sanofi‐Genzyme; he receives research support from Biogen Idec, Merck‐Serono, Novartis, Roche, the Italian Ministry of Health, the Italian Ministry of University and Research, and FISM.

## OPR‐051

### Genetic insights into functional neurological and somatoform disorders: Pilot findings from the first GWAS

#### V. Fominykh ^1^; P. Jahołkowski^1^; E. Koch^1^; L. Luitva^2^; Á. Skúladóttir^3^; D. Mikkelsen^4^; M. Lundberg^4^; A. Schork^4^; O. Pedersen^5^; S. Ostrowski^6^; C. Erikstrup^7^; K. O'Connell^1^; T. Werge^4^; L. Milani^2^; H. Steffanson^3^; A. Shadrin^1^; O. Andreassen^1^

##### ^1^Institute of Clinical Medicine, University of Oslo and Section for Precision Psychiatry, Oslo University Hospital, Oslo, Norway; ^2^Estonian Genome Centre, Institute of Genomics, University of Tartu, Tartu, Estonia; ^3^deCODE Genetics, Reykjavik, Iceland; ^4^Institute of Biological Psychiatry, Mental Health Services Copenhagen, Copenhagen University Hospital, Copenhagen, Denmark and iPSYCH, The Lundbeck Foundation Initiative for Integrative Psychiatric Research, Denmark; ^5^Department of Clinical Immunology, Zealand University Hospital, Køge, Denmark; ^6^Department of Clinical Immunology, Copenhagen University Hospital, Rigshospitalet, Copenhagen, Denmark & Department of Clinical Medicine, Faculty of Health and Medical Sciences, University of Copenhagen, Copenhagen, Denmark; ^7^Department of Clinical Immunology, Aarhus University Hospital, Aarhus, Denmark & Department of Clinical Medicine, Aarhus University, Aarhus, Denmark

**Background and Aims:** The mechanisms underlying functional neurological disorders (FND) are poorly known, even though FND is common, with significant impact on healthcare. Positive family history and small‐scale candidate‐gene studies indicated a genetic role in FND. However, large‐scale genetic studies, which can provide a necessary first step in the genetics of disorders, have not yet been carried out.

**Methods:** We performed the first large‐scale genome‐wide association study (GWAS) meta‐analysis in FND and somatoform disorders (SD) using data from Nordic countries (Estonia, Denmark, Finland, Iceland, Norway), the UK and the USA (FND N cases = 4269, N controls = 1852274, SD N cases = 18536, N controls = 1819203). GWASes were performed separately in each cohort (covariates: age, sex, first 20 PCs and genotyping batch). We used cleansumstat pipeline for harmonization, METAL for meta‐analysis and LDSC tool for heritability and correlation assessment.

**Results:** We found one genome‐wide significant locus on chromosome 8 in FND GWAS and two loci, on chromosome 8 and 16, in SD GWAS. LDSC analysis of the GWAS data showed that the estimated observed‐scale SNP heritability for FND is 14 % and for SD is 7%. The FND and SD were strongly genetically correlated (rg = 0.942) suggesting a similar genetic basis. Significant LDSC correlations were revealed with all major psychiatric disorders, chronic pain, neuroticism and migraine.

**Conclusion:** Current results can help us to understand functional disorder pathophysiology, as well as genetic role in FND and SD. We showed shared genetic aetiology for FND and SD with chronic pain, migraine, neuroticism and major psychiatric disorders.

**Disclosure:** This work was made with support from RCN grant 324252. We want to acknowledge the participants and investigators of the FinnGen, MOBA, UK and Estonia biobank, AllofUs studies, DBDS genetic consortium and deCODE, Iceland.

## Muscle and Neuromuscular Junction Disorder 1

## OPR‐052

### Association between new‐onset myasthenia gravis and COVID‐19 infection and vaccination. A population‐based study

#### T. Kab^1^; K. Pardo^1^; M. Hellmann^1^; T. Friedman^1^; I. Lotan^1^; t. Shochat^2^; A. Wilf‐Yarkoni ^1^

##### ^1^Department of Neurology, Rabin Medical Center, Petah Tikva, Israel; ^2^Statistical Unit, Rabin Medical Center, Petah Tikva, Israel

**Background and Aims:** The potential link between myasthenia gravis (MG) and COVID‐19 infection or vaccination remains unclear. This study aimed to evaluate the relationship between these factors and new‐onset MG.

**Methods:** A case‐control study was conducted using two cohorts from Clalit Health Services' database. We applied a machine learning algorithm to reduce diagnostic misclassification. The study examined adults aged 18 or older between January 2020 and December 2022 for COVID‐19 infection (Infection Cohort) and between January 2021 and December 2022 for COVID‐19 vaccination (Vaccination Cohort). For each new MG case, three matched controls by age and sex were selected. Prior exposure to either infection or vaccination was assessed within 90 and 180 days for cases and controls.

**Results:** In the infection cohort, 253 new MG cases were identified. A multivariate logistic regression model showed an odds ratio (OR) of 1.44 (95% CI 0.708–2.92) within 90 days post‐infection and 1.67 (95% CI 0.98–2.84) within 180 days. In the vaccination cohort, 177 new MG cases were detected, with an OR of 1.76 (95% CI 1.049–2.95) within 90 days post‐vaccination and 2.45 (95% CI 1.51–3.95) within 180 days.

**Conclusion:** The study suggests no increased risk of new‐onset MG following COVID‐19 infection, but vaccination appears to be associated with a higher risk of developing MG, particularly within 180 days of vaccination.

**Disclosure:** Nothing to disclose.

## OPR‐053

### Targeted immunotherapy with ANX005 reduces ventilation requirements in Guillain‐Barré syndrome (GBS)

#### H. Kroon ^1^; Q. Mohammad^2^; J. Navarro^3^; G. Morrison^1^; P. Lin^1^; R. Gerwien^4^; P. Collins^1^; K. Azad^5^; Z. Islam^6^; K. Gorson^7^

##### ^1^Annexon Biosciences, Brisbane, CA, USA; ^2^National Institute of Neurosciences & Hospital, Dhaka, Bangladesh; ^3^José R. Reyes Memorial Medical Center, Manila, Philippines; ^4^Gerwien Analytical Solutions, Newington, CT, USA; ^5^Dhaka Medical College and Hospital, Dhaka, Bangladesh; ^6^Gut–Brain Axis Laboratory, icddr,b, Dhaka, Bangladesh; ^7^St. Elizabeth's Medical Center, Boston, MA, USA

**Background and Aims:** Guillain‐Barré syndrome (GBS) is a neuromuscular emergency requiring hospitalization and necessitating mechanical ventilation in severe cases. GBS‐02, a randomized, double‐blind, placebo‐controlled Phase 3 trial, assessed the safety and efficacy of ANX005, a C1q inhibitor that inhibits the classical complement pathway, in GBS. This analysis evaluates ANX005's impact on duration of ventilation, a critical disease burden marker.

**Methods:** Ventilation duration was analyzed using a zero‐inflated negative binomial (ZINB) model. Study patients who die or die early while on mechanical ventilation could bias ventilation duration. Therefore, death and treatment exposure at time of ventilation were considered intercurrent events, with those never ventilated assigned a duration of zero days, while those who died on ventilation were imputed with 182 days (trial length). This includes one patient in each active arm who died without ventilation. Subgroups who required ventilation during the study or were ventilated before treatment were assessed with a Kruskal–Wallis test.

**Results:** The ZINB model showed significant reduction in ventilation duration with ANX005 compared to placebo (28‐day median reduction, *p* = 0.0356 for ANX005 30 mg/kg; 34‐day median reduction, *p* = 0.0011 for ANX005 75 mg/kg). In ventilated patients without imputation, median duration of ventilation was reduced by 15 days for ANX005 30 mg/kg (*p* = 0.0079) and 20 days for ANX005 75 mg/kg (*p* = 0.0080). Similar reductions were observed when analyses were restricted to patients receiving treatment during ventilation (*p* = 0.0034).

**Conclusion:** These results demonstrate that ANX005 significantly reduces ventilation duration in GBS, even for patients already ventilated at treatment initiation, highlighting its potential to improve critical outcomes in severe GBS.

**Disclosure:** The study was sponsored by Annexon Biosciences (Brisbane, CA, USA). HAK: Employee and shareholder of Annexon Biosciences QDM: Consultancy/advisory role with Annexon Biosciences JN: Consultancy/advisory role with Annexon Biosciences GM: Employee and shareholder of Annexon Biosciences PL: Employee and shareholder of Annexon Biosciences RG: Consultancy/advisory role with Annexon Biosciences PC: Employee and shareholder of Annexon Biosciences KAKA: No disclosures ZI: Research funding from Fogarty International Center, National Institute of Neurological Disorders and Stroke of the National Institutes of Health, USA, and Annexon Biosciences KCG: Consultancy/advisory role with Annexon Biosciences, Argenx, Janssen, and Sanofi.

## OPR‐054

### Real‐time MRI and quantitative muscle ultrasound for reliable assessment of dysphagia in myositis and muscular dystrophy

#### R. Zeng^1^; A. Rietvelt^2^; O. Al‐Bourini^3^; R. Kroon^2^; A. Olthoff^4^; M. Weidenmüller^4^; P. Carstens^1^; I. Kommerell^1^; S. Schütz^1^; C. Horlings^2^; J. Kalf^2^; B. de Swart^2^; B. van Engelen^2^; T. Friede^5^; S. Hofer^6^; J. Frahm^6^; A. Seif Amir Hosseini^3^; C. Saris^2^; J. Schmidt ^7^

##### ^1^Department of Neurology, Neuromuscular Center, University Medical Center Göttingen, Göttingen, Germany; ^2^Department of Neurology, Radboudumc Research Institute for Medical Innovation, Nijmegen, The Netherlands; ^3^Department of Diagnostic and Interventional Radiology, University Medical Center Göttingen, Göttingen, Germany; ^4^Department of Otorhinolaryngology, Phoniatrics and Pedaudiology, University Medical Center Göttingen, Göttingen, Germany; ^5^Department of Medical Statistics, University Medical Center Göttingen, Göttingen, Germany; ^6^Biomedical NMR, Max Planck Institute for Multidisciplinary Sciences, Göttingen, Germany; ^7^Department of Neurology and Pain Treatment, Immanuel Klinik Rüdersdorf, University Hospital of the Brandenburg Medical School Theodor Fontane, Rüdersdorf bei Berlin, Germany

**Background and Aims:** Dysphagia frequently debilitates neuromuscular patients. Its reliable identification is important for diagnosis and treatment. We studied real‐time MRI and quantitative muscle ultrasound (QMUS) for characterizing dysphagia in two different neuromuscular disorders.

**Methods:** This prospective cohort study included 18 patients with inclusion body myositis IBM and 13 with oculopharyngeal muscular dystrophy (OPMD) from two Neuromuscular centers (Nijmegen/NL; Göttingen/DE). Swallowing function was assessed by real‐time MRI (RT‐MRI), FEES (flexible endoscopic evaluations of swallowing) and clinical assessments. T1‐mapping and QMUS were used to analyze tissue properties in swallowing muscles. Outcomes were compared between the two muscle diseases. RT‐MRI values were compared to 22 matched non‐myopathic controls.

**Results:** RT‐MRI revealed significantly prolonged oral transit times in OPMD vs. controls (difference between means = 581.2 ms, 95% CI 225.9–936.4, *p* = 0.002). Pharyngeal transit time was significantly prolonged in IBM vs. controls (difference between means = 1132.8 ms, 95% CI 482.2–1783, *p* = 0.001). A cricopharyngeal bar as a well‐established morphological indicator of dysphagia was identified in 80% patients with IBM compared to 53% in OPMD. Fatty degeneration of the tongue in OPMD significantly correlated between MRI‐T1 values and ultrasound echogenicity (Spearman's ρ = −0.52, *p* = 0.005). ROC revealed excellent discrimination between diseases by combining RT‐MRI, T1‐mapping and QMUS (AUC = 0.95, 95% CI 0.86–1.00), while FEES and clinical assessments failed to differentiate specific patterns of dysphagia.

**Figure:**
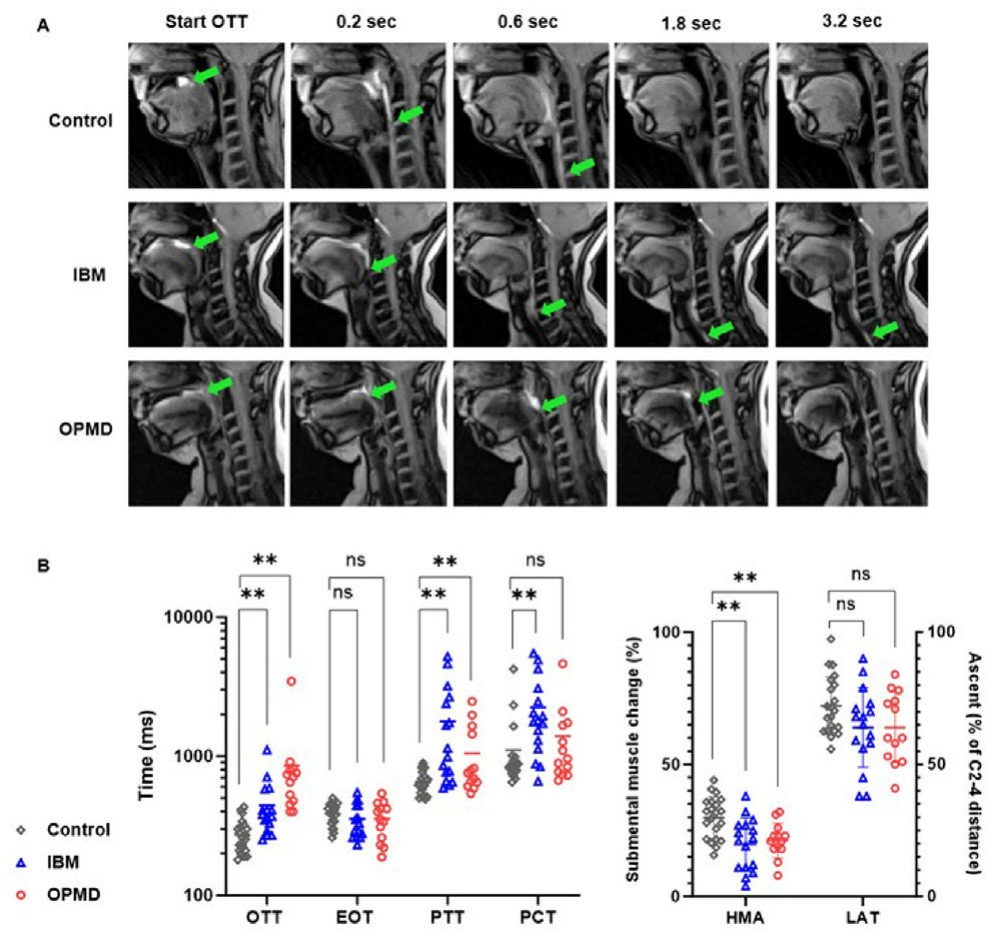
**FIGURE 1** Real‐time MRI assessment of swallowing in patients with IBM and OPMD versus control subjects.

**Figure:**
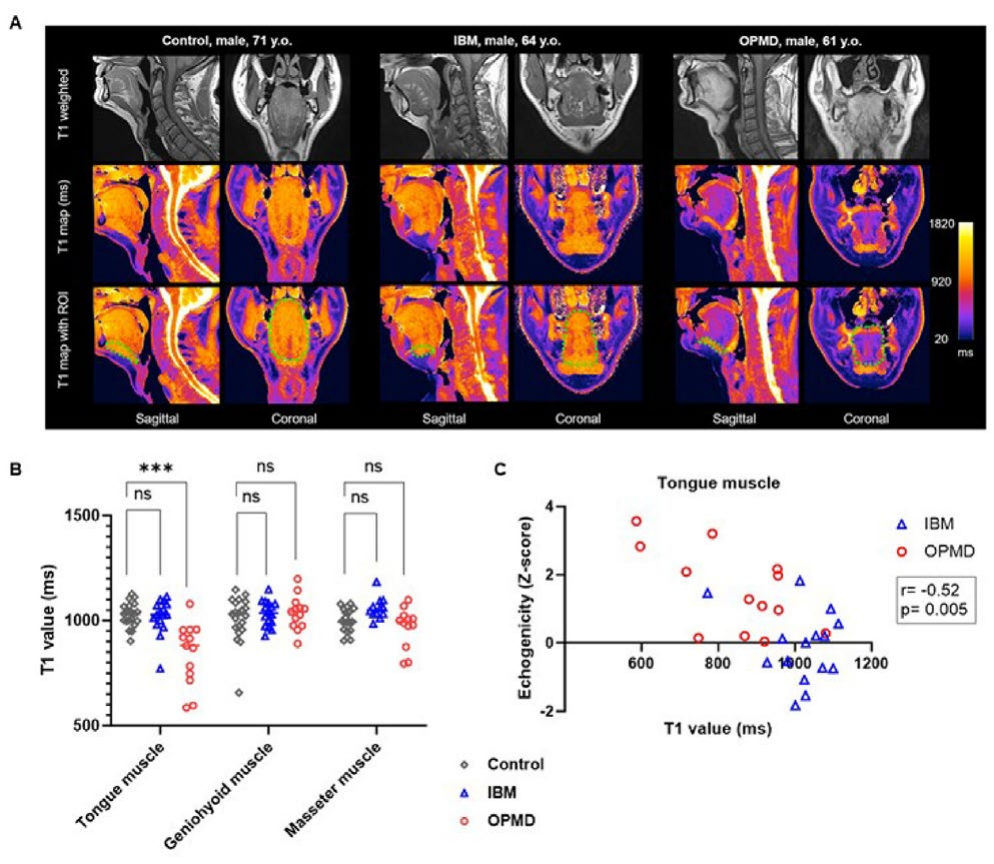
**FIGURE 2** T1 mapping of swallowing muscles in patients with IBM and OPMD versus control subject.

**Figure:**
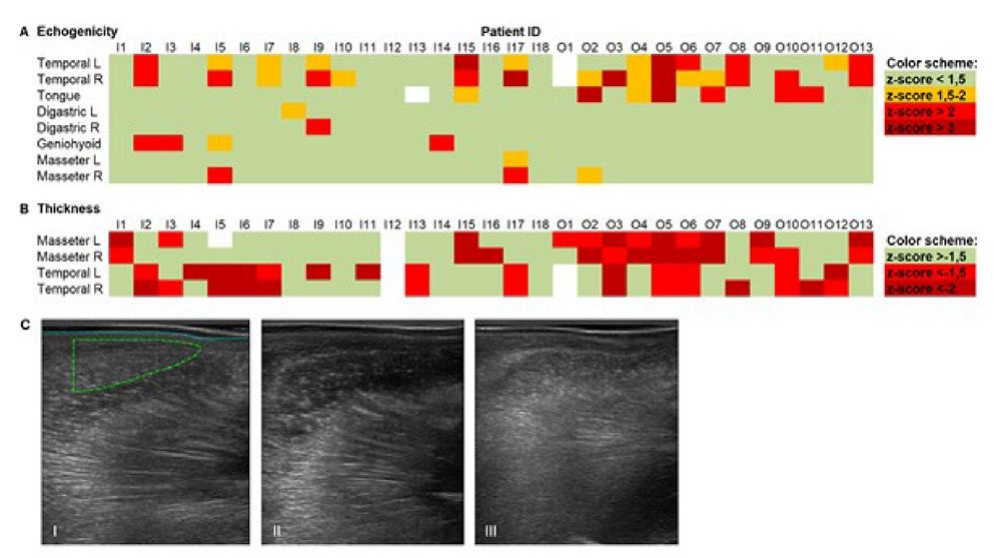
**FIGURE 3** Quantitative muscle ultrasound (QMU) of orofacial muscles in IBM and OPMD patients.

**Conclusion:** This study supports the value of novel MRI and ultrasound techniques for clinical use by identifying the pathophysiology and severity of impaired swallowing. Differentiating the phenotypes of dysphagia can aid in the diagnosis and treatment of affected patients.

**Disclosure:** Nothing to disclose.

## OPR‐055

### A multicenter, randomized, double blind, phase 3a study of telitacicept in generalized myasthenia gravis

#### M. Zhao ^1^; J. Yin^1^; H. Deng^2^; M. Zhang^3^; Z. Xu^4^; L. MIn^5^; B. Bu^6^; S. Liu^7^; Z. Yan^8^; G. Xie^9^; A. Xie^10^; M. Liu^10^

##### ^1^Department of Neurology, Beijing Hospital, National Center of Gerontology, Institute of Geriatric Medicine, Chinese Academy of Medical Sciences; ^2^Department of Neurology, First Hospital of Jilin University; ^3^Department of Neurology, First Hospital of Shanxi Medical University, Taiyuan; ^4^Department of Neurology, Affiliated Hospital of Zunyi Medical University; ^5^Department of Neurology, First Affiliated Hospital of Jinzhou Medical University; ^6^Department of Neurology, Tongji Hospital, Tongji Medical College, Huazhong University of Science and Technology; ^7^Department of Neurology, China‐japan Friendship Hospital, Jilin University; ^8^Department of Neurology, Jining First People's Hospital; ^9^Department of Neurology, Li Huili Hospital, Ningbo Medical Center; ^10^Department of Neurology, The First Affiliated Hospital of Qingdao University

**Background and Aims:** The recent phase 2 study suggested the safety and good tolerability of telitacicept, a dual BAFF/APRIL inhibitor, in treating gMG. The phase 3a study aimed to further validate these preliminary findings.

**Methods:** Eligible patients were aged ≥18 years with diagnosis of gMG, a Myasthenia Gravis Foundation of America classification of II–IVa, a Myasthenia Gravis‐Activities of Daily Living (MG‐ADL) score ≥6, a quantitative myasthenia gravis (QMG) score ≥8, and standard of care treatment. Patients were randomly assigned 1:1 to either receive telitacicept 240mg or matched placebo subcutaneously once a week for 24 weeks. The primary efficacy endpoint was the mean change from baseline to week 24 in MG‐ADL score. Secondary efficacy endpoints included mean change in QMG score from baseline to week 24, mean change in MG‐ADL score and QMG score from baseline to week 12. Safety and tolerability were assessed.

**Results:** Between 2023 and 2024, 114 of the 148 patients screened were enrolled in 52 hospitals in mainland China. The mean reduction in MG‐ADL score from baseline to week 24 was 6.4 and 1.6 (*p* < 0.001) in the telitacicept 240mg and placebo groups, respectively. In addition, at Week 24, QMG score, Myasthenia Gravis Composite (MGC) scale, Myasthenia Gravis Quality of Life 15‐item‐revised (MG‐QOL15r) questionnaire, MG clinical absolute score were significantly improved in the telitacicept 240mg group than the placebo group (*p* < 0.001). Safety analysis revealed telitacicept did not increase the risk of infection compared with placebo.

**Figure:**
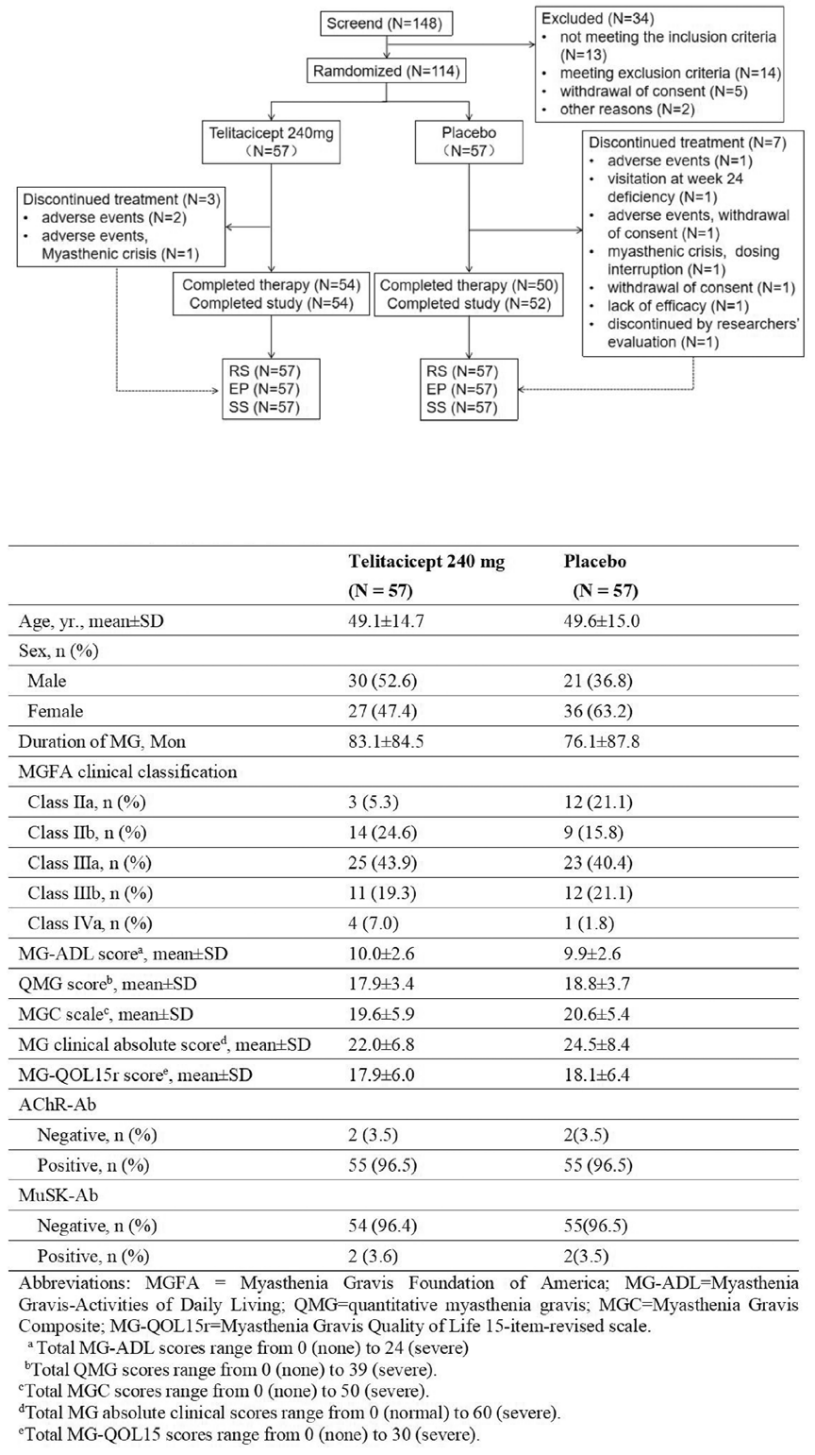
**FIGURE 1** Consort flow diagram. **TABLE 1** Study Population and Baseline Characteristics.

**Figure:**
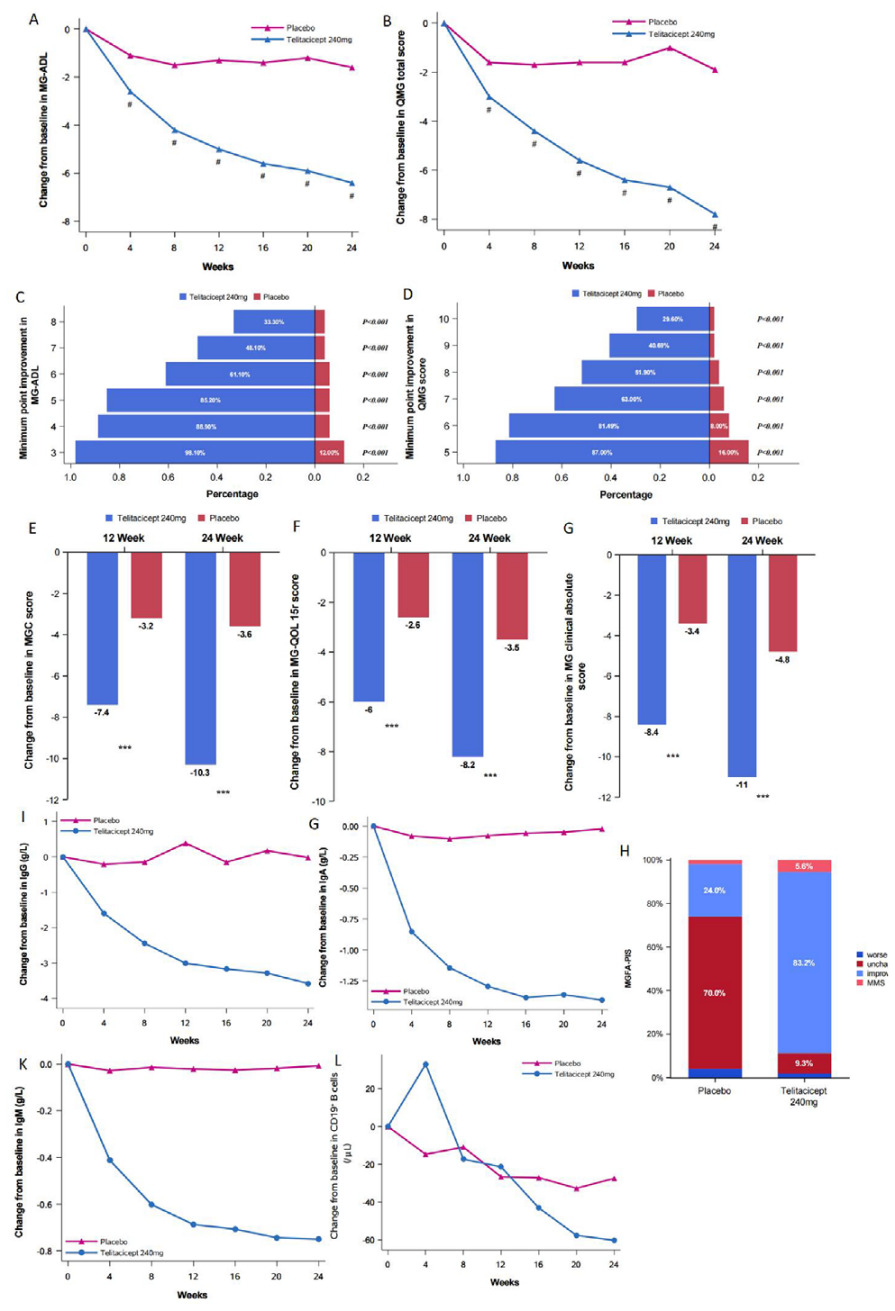
**FIGURE 2** Mean change in MG‐ADL，QMG，MGC，MG‐QOL 15r，MG clinical absolute score from baseline to week 24(A‐G). MG‐PIS at week 24 (H). Mean percentage change in immunoglobulin levels and CD19+ B‐cell counts (I‐L).)

**Figure:**
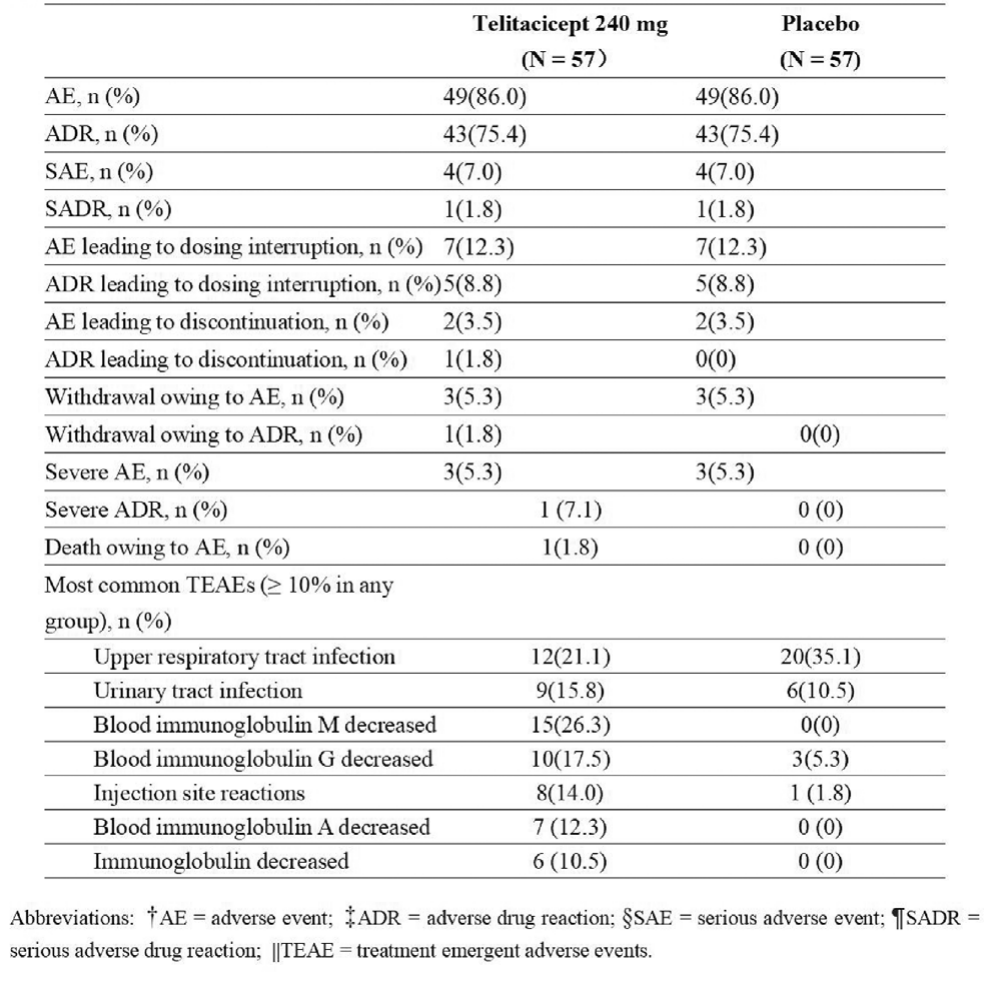
**TABLE 2** Summary of Adverse Events in All Patients.

**Conclusion:** Telitacicept 240mg treatment showed significant clinical benefit and safety in gMG patients in phase 3a study.

**Disclosure:** Nothing to disclose.

## OPR‐056

### BCMA/CD19 dual CAR‐T cells for refractory myasthenia gravis

#### T. Chang ^1^; Z. Ruan^1^; Y. Li^2^; F. Ning^1^; H. Li^2^; S. Wang^2^; Q. Liu^3^

##### ^1^Department of Neurology, the SeconAffiliated Hospital, the Air Force Medical University; ^2^Institute of Biophysics, Chinese Academy of Sciences; ^3^Department of Neurology, Tianjin Neurological Institute, Tianjin Medical University General Hospital

**Background and Aims:** Myasthenia gravis (MG) is a potentially fatal autoimmune disease, with up to 15% of MG refractory to conventional immunotherapy. This study aims to evaluate the safety and efficacy of low‐dose BCMA/CD19 dual chimeric antigen receptor T cells (CAR‐T) for patients with refractory MG.

**Methods:** Three patients with acetylcholine receptor antibody‐positive refractory MG received 5x105 BCMA/CD19 dual CAR‐T cells per kg without lymphodepletion. Follow‐ups were conducted at 1‐, 2‐, and 3‐months post‐infusion to assess changes in MG clinical scores (QMG, MG‐ADL, MGC and MG‐QOL15 scales). Bone marrow and blood samples were collected during follow‐ups for flow cytometry and single‐cell sequencing to assess CAR‐T cell expansion and immune cell profile.

**Results:** BCMA/CD19 dual CAR‐T cells expanded in patients, peaking around day 10 post‐infusion, accompanied with rapid B cell depletion and improvement in clinical symptoms. Baseline MG‐ADL scores were 8, 7 and 15 in the three patients. At the second month post‐infusion, all patients achieved complete symptom remission, with MG‐ADL scores reduced to 0, and one patient discontinued all medications. Patients showed a good safety profile, with 2 patients developing transient fever on days 7–8 post‐infusion, and no other CAR‐T‐related complications were identified. The analysis of longitudinal bone marrow and blood samples demonstrated sustained depletion of B cells and plasma cells until the 3‐month of follow‐up, along with decreasing acetylcholine receptor autoantibody titers.

**Figure:**
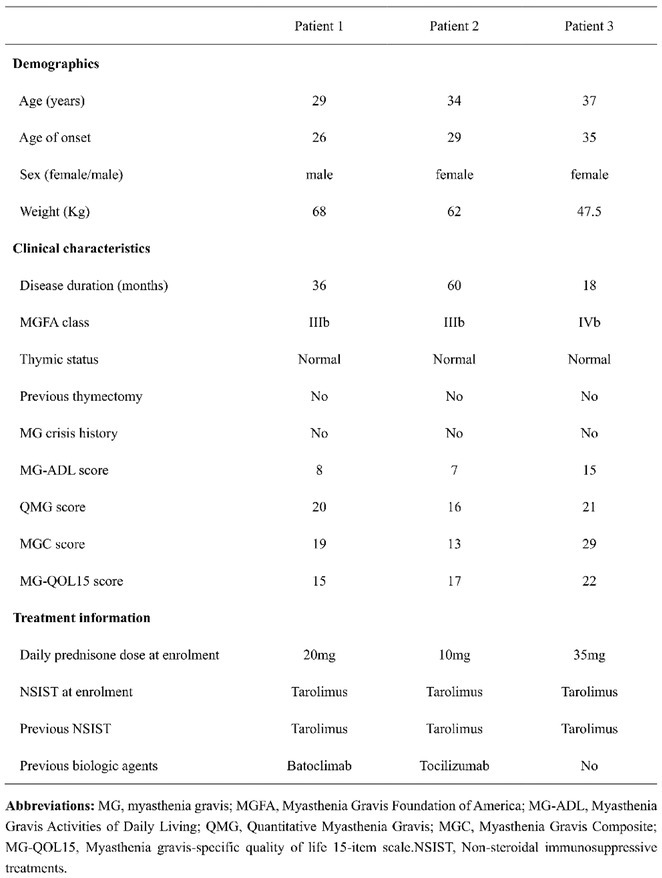
**TABLE** The baseline characteristics and treatment details of the 3 MG patients

**Figure:**
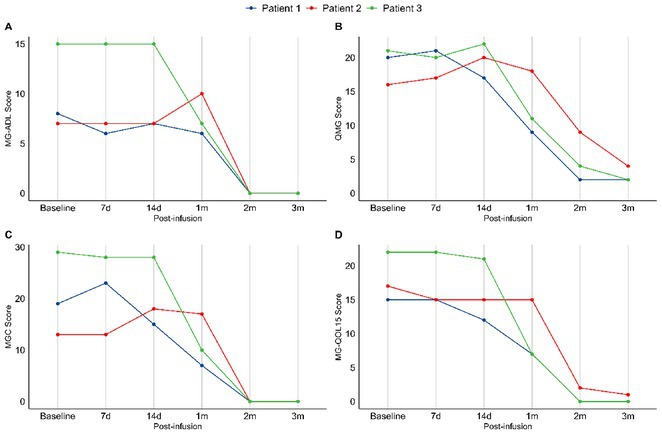
**FIGURE 1** Kinetic parameters of Patient MG‐1, MG‐2 and MG‐3, including MG‐ADL scale score, QMG score, MGC score and the MGQOL15 questionnaire.

**Conclusion:** Low‐dose BCMA/CD19 dual CAR‐T cells are safe and can deplete B cells and plasma cells in patients with refractory MG without lymphodepletion, resulting in rapid clinical improvement.

**Disclosure:** Nothing to disclose.

## Sleep‐wake Disorders 1

## OPR‐057

### Investigating hypoxic burden in alzheimer's disease: A pilot study

#### E. Rollo; B. Tafuri; V. Gnoni; L. Tamburrino; D. Urso; A. Giugno; D. Vilella; C. Zecca; R. De Blasi; G. Logroscino

##### Center for Neurodegenerative Diseases and the Aging Brain, University of Bari Aldo Moro at Pia Fondazione “Card. G. Panico”, Tricase, Italy

**Background and Aims:** Obstructive Sleep Apnea (OSA) is a frequent comorbidity in Alzheimer's Disease (AD), with chronic intermittent hypoxia being one of the hypothesized mechanisms connecting these two conditions. Hypoxic Burden (HB), an indicator of both duration and depth of oxygen desaturations, is a novel OSA severity marker. The primary aim of this pilot study was to explore the correlation between HB and AD biomarkers, clinical, neuropsychological, and neuroimaging measures in AD patients.

**Methods:** AD stage 3 and stage 4 patients were consecutively enrolled at Center for Neurodegenerative Diseases and Aging Brain, Tricase. All patients underwent 3T brain Magnetic Resonance Imaging (MRI), cerebrospinal fluid (CSF) AD biomarkers analysis, and a comprehensive neuropsychological assessment. Sleep questionnaires and overnight polysomnography were performed in all patients. A MATLAB code was employed for HB calculation, and Brain MRI morphometry was analyzed using the FreeSurfer software.

**Results:** The cohort consisted of 22 patients (63.6% females, mean age 69.8 ± 7.5). OSA was diagnosed in 40.9% of patients. Mean HB was 46.4 ± 57.4. HB inversely correlated with left hippocampus (*r* = −0.621, *p* = 0.014) and left amygdala (*r* = −0.538, *p* = 0.038) volumes, after controlling for age, sex and disease duration. After controlling for the same factors, HB positively correlated with Dimensional Apathy Scale (*r* = 0.74, *p* = 0.014), Epworth Sleepiness Scale (*r* = 0.648, *p* = 0.043) and Pittsburgh Sleep Quality Index (*r* = 0.643, *p* = 0.045) scores.

**Conclusion:** Higher HB is associated with left hippocampus and amygdala atrophy, key structures involved in memory consolidation and emotion regulation. These results suggest that chronic hypoxia, measured by a novel OSA metric, may contribute to neurodegeneration in AD.

**Disclosure:** Nothing to disclose.

## OPR‐058

### Prevalence of idiopathic REM sleep behavior disorder in the Spanish community

#### G. Mayà ^1^; M. Qi YE^2^; E. Senar^2^; M. Poblete^2^; C. Gaig^1^; L. Molina‐Porcel^3^; S. Casablancas^2^; A. Iranzo^1^

##### ^1^Sleep Unit, neurology department, Hospital Clinic; ^2^CAP Casanova, CAPSBE Consorci d’Atenció Primària de Salut Barcelona Esquerra, Spain; ^3^Alzheimer's diseases and other cognitive disorders, neurology department, Hospital Clinic

**Background and Aims:** To study the prevalence and clinical characteristics of idiopathic REM sleep behavior disorder (IRBD) in a representative Caucasian sample from the elderly community of Barcelona, Spain, attending primary care centers.

**Methods:** Participants were individuals aged 60 years or older who underwent routine visits in a primary care center (CAP Casanova) between 7th February 2023 and 29th August 2024. They underwent a two‐stage study; a validated screening single question for IRBD diagnosis (RBD1Q) followed by, in those who endorsed positive answer, clinical assessment by a neurologist plus video‐polysomnography (V‐PSG).

**Results:** Of 332 individuals (62% women, mean age 71.8 ± 7.9 years, range 60–93), 33 (9.9%) endorsed positively the RBD1Q. All 33 were interviewed by a sleep neurologist, and 20 of these 33 accepted a v‐PSG. V‐PSG ruled out RBD in 16 subjects who had obstructive sleep apnea (*n* = 10), periodic limb movement disorder in sleep (*n* = 4) and normal sleep (*n* = 2). IRBD was diagnosed in four individuals without motor or cognitive complaints, giving an estimated prevalence of 1.20% (95% CI = 0.03–2.37). They were three men and one woman between 64 and 76 years of age, with an interval between estimated RBD onset and V‐PSG of 7.5 years (range 0–5–10 years). IRBD patients had constipation (*n* = 3), hyposmia (*n* = 2), apathy (*n* = 1), and depression (*n* = 1).

**Conclusion:** The prevalence of IRBD is 1.2% in the elderly community of Barcelona, similar than in other studies in the Caucasian elderly community.

**Disclosure:** Nothing to disclose.

## OPR‐059

### Sleep and longevity: Insights from sleep macroarchitecture and nocturnal heart rate variability

#### I. Filchenko; J. van der Meer; C. Bernasconi; M. Schmidt; C. Bassetti

##### Department of Neurology, University Hospital, Inselspital, Bern, Switzerland

**Background and Aims:** Healthy aging is priority in public health. Emerging evidence indicates that sleep‐wake disorders are causally linked to adverse health outcomes, suggesting that sleep represents a key preventive target. We aimed to identify sleep phenotypes associated with selected incident comorbidities using real‐world data.

**Methods:** This is an analysis of the Bernese Sleep‐Wake Registry (*n* ≈ 11000). Using polysomnography, we quantified sleep macroarchitecture according to AASM criteria and nocturnal heart rate variability (21 parameter) as the marker of autonomic functioning. 36 incident comorbidities at polysomnography and during the follow‐up were classified into eight major groups. Association between comorbidities and sleep parameters were explored with multiple logistic regression adjusted for age and sex.

**Figure:**
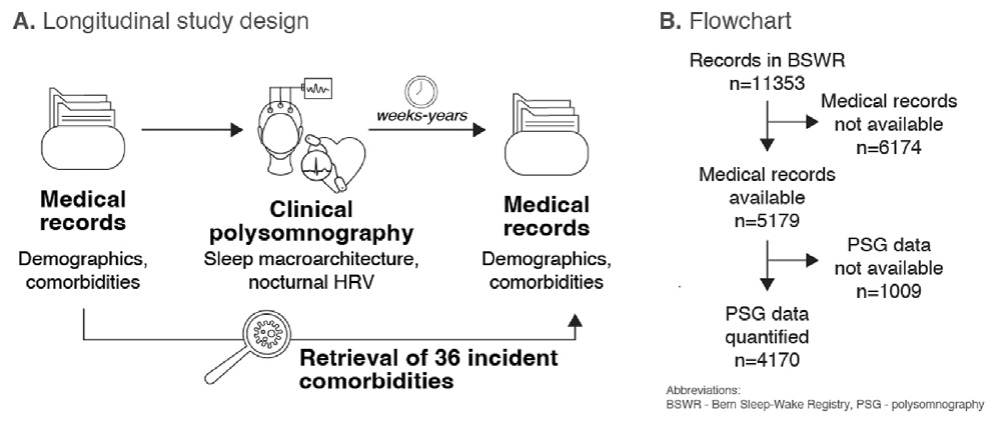
**FIGURE 1** Study design and flowchart.

**Figure:**
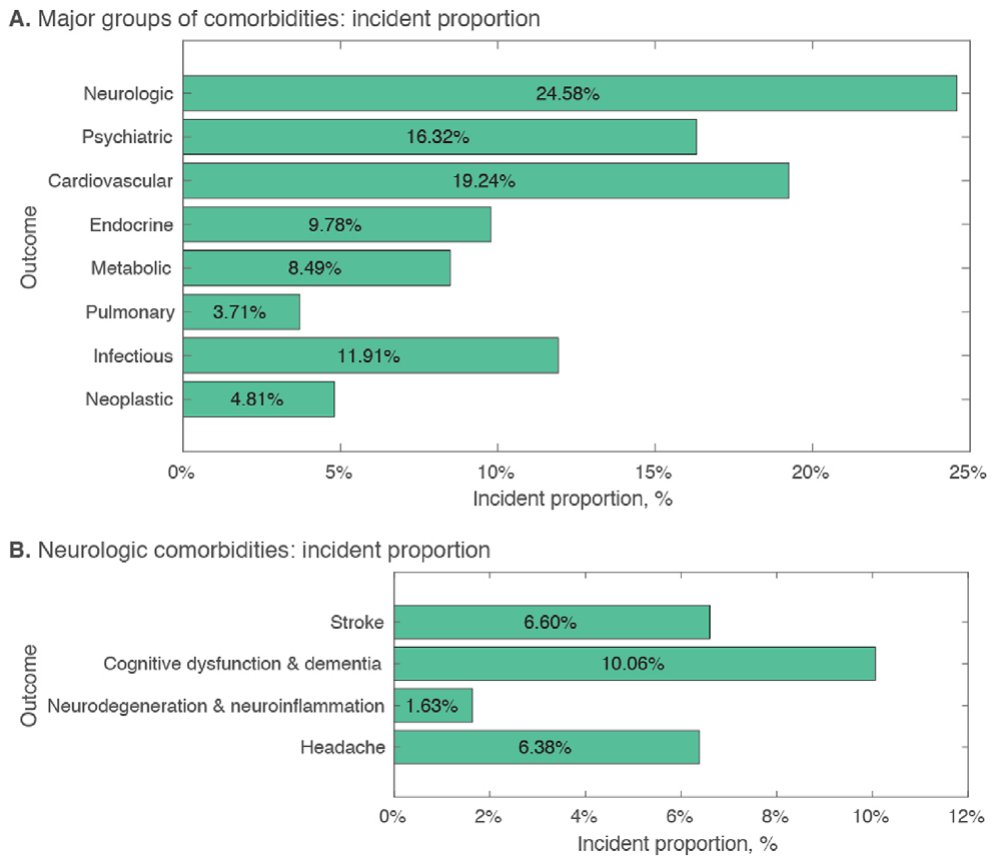
**FIGURE 2** Incident proportion of eight major groups of comorbidities that emerging during the follow‐up.

**Results:** 4170 participants were included in the analysis (age: 48±19 years, 63% men, total observation time: 13217 person‐years). Sleep macroarchitecture showed limited associations with incident comorbidities, with sleep‐disordered breathing being linked to cardiovascular, endocrine, and metabolic diseases. In contrast, HRV was prevalently associated with incident comorbidities. Specifically, neurologic diseases were related to high and complex HRV. In a subanalysis, stroke demonstrated strong associations with HRV. Psychiatric diseases (i.e., depression in 92% cases) were associated with low HRV, accompanied by reduced complexity and parasympathetic dominance. Metabolic diseases were linked to high HRV with a high very low‐frequency component.

**Figure:**
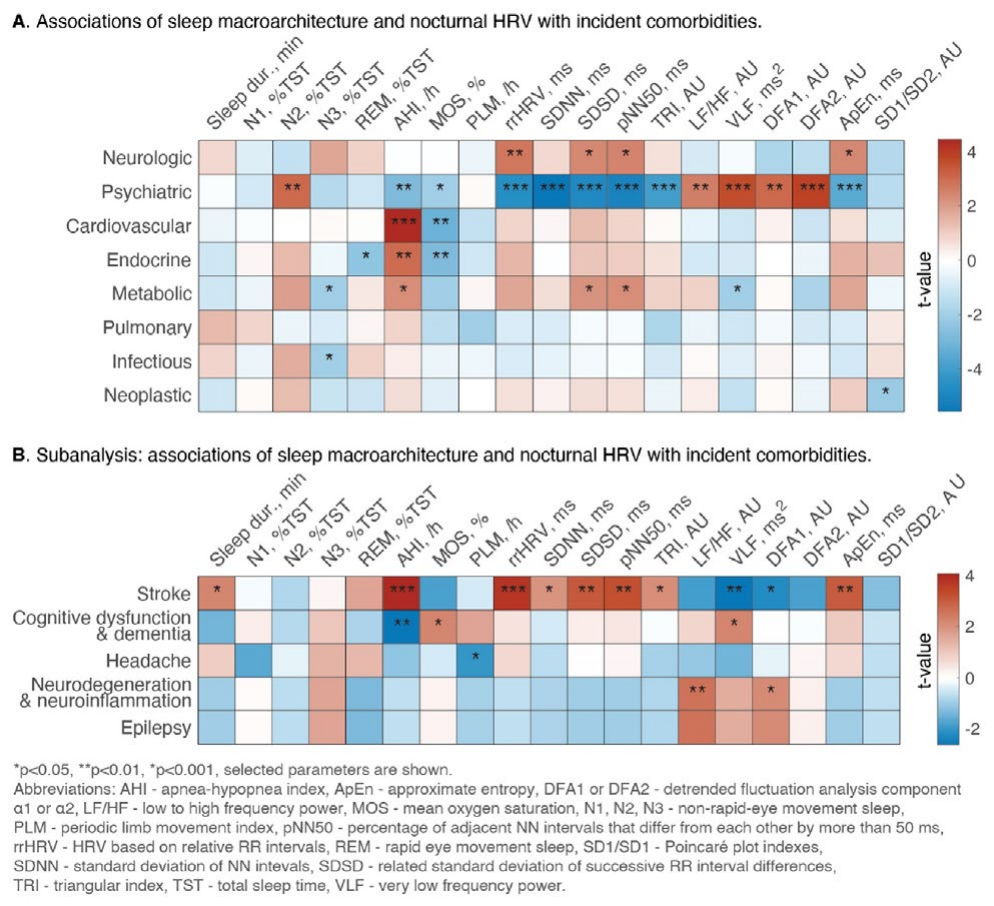
**FIGURE 3** Main findings. Heatmap of the t‐values of the multiple logistic regression adjusted for age and sex.

**Conclusion:** These findings highlight sleep profiles linked to unfavorable health outcomes in a longitudinal analysis. Nocturnal HRV emerges as a relevant marker for neurologic and psychiatric diseases, suggesting its potential in brain health. We will further explore the link between health outcomes and sleep microarchitecture.

**Disclosure:** Nothing to disclose.

## OPR‐060

### REM‐sleep saw‐tooth waves: Cortical topography and associations with cognition

#### I. Filchenko ^1^; C. Bernasconi^1^; C. Bassetti^1^; A. Eberhard‐Moscicka^2^; M. Schmidt^1^

##### ^1^Department of Neurology, University Hospital, Inselspital, Bern, Switzerland; ^2^Department of Psychology, University of Bern, Bern, Switzerland

**Background and Aims:** Saw‐tooth waves (STWs) are a hallmark of REM sleep, however, their association with cognition remains poorly understood. This exploratory analysis comprehensively addressed this research gap.

**Methods:** The “Sleep and cognitive functioning” study included volunteers in good or excellent health condition (Eastern Cooperative Oncology Group grade of 0‐1). Demographics, medical history, cognition, and sleep architecture by polysomnography (electroencephalography [EEG] was recorded either with 6 electrodes or 256 electrodes [high‐density EEG, hd‐EEG]) were assessed at study inclusion (Figure 1AB). STWs were detected using a feature‐based data‐driven algorithm in MATLAB (Figure 1C). Associations between STW properties as dependent variables and cognition were explored using multivariate linear regression adjusted for age and arousal index.

**Figure:**
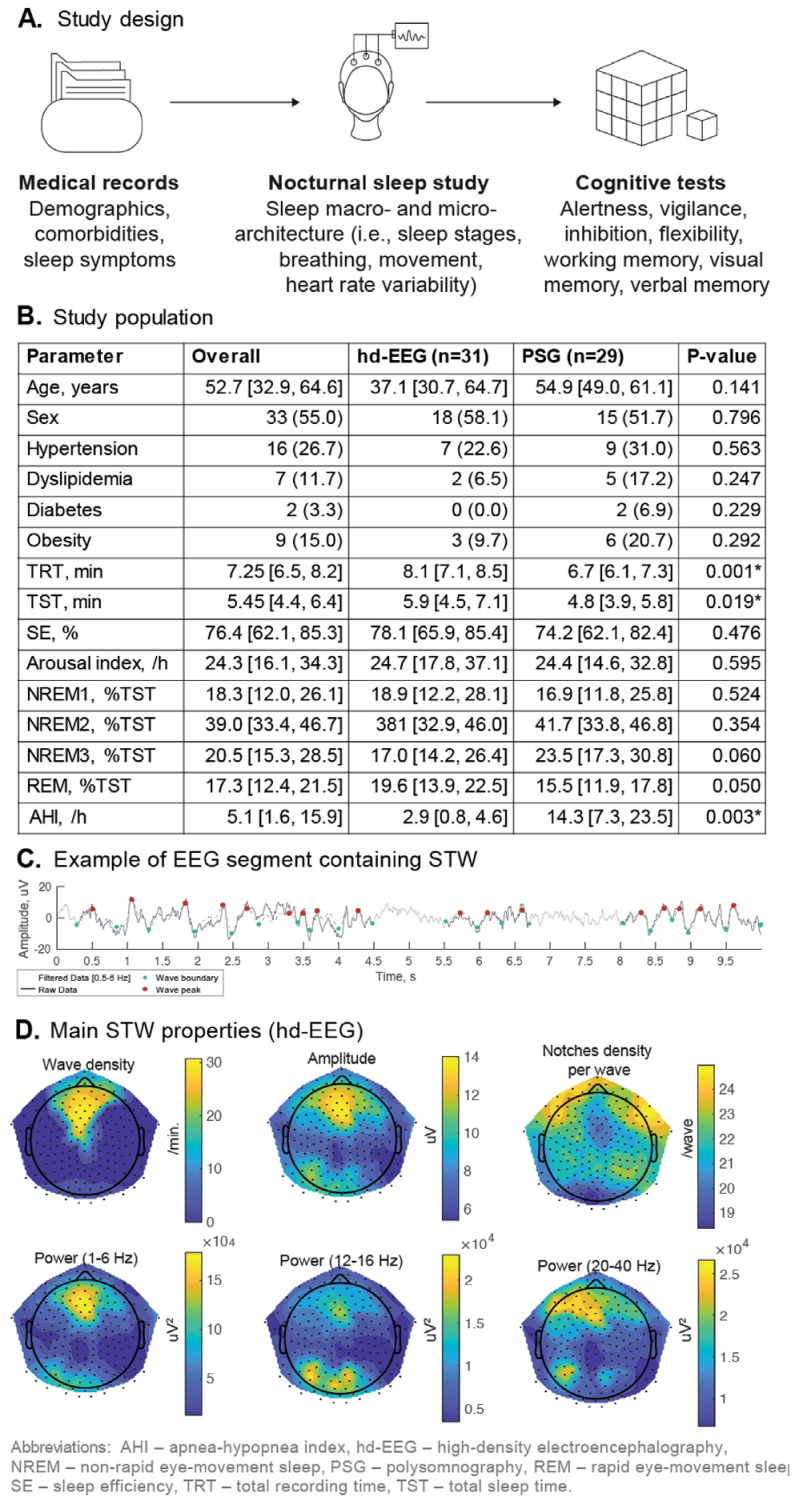
**FIGURE 1** Study design (A, B) and STW detection (C, D).

**Results:** 60 participants were included (hd‐EEG in 52%; Figure 1B). STW were expressed in the fronto‐central areas, with 0.5–6 Hz and 20–40 Hz activity dominating in the frontal regions and 12–16 Hz activity ‐ in the posterior regions. EEG analysis based on 6 electrodes showed limited associations between STW and cognition (e.g., low SWT amplitude with vigilance or alertness; Figure 2), whereas high‐density EEG analysis identified multiple associations of interest with a distinct topography (e.g., visual memory with high EEG spectral power in lateral regions; Figure 3).

**Figure:**
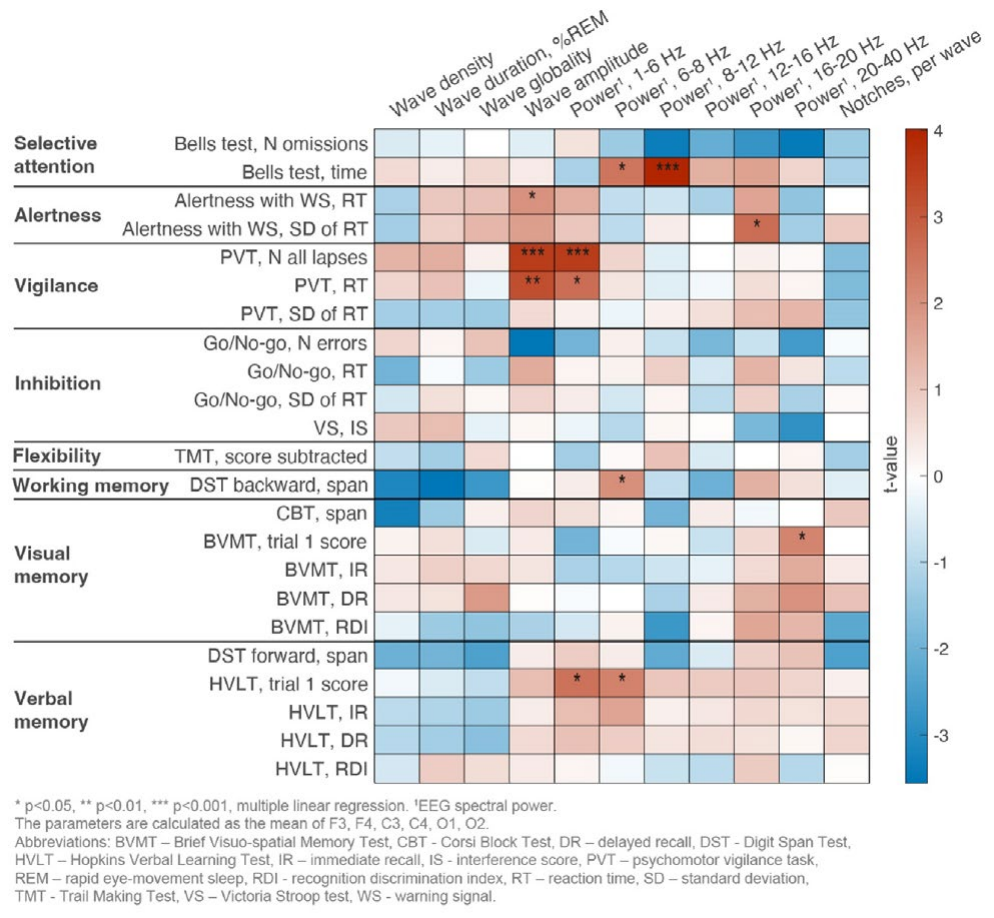
**FIGURE 2** Associations between STW and cognition (basic analysis, *n* = 60).

**Figure:**
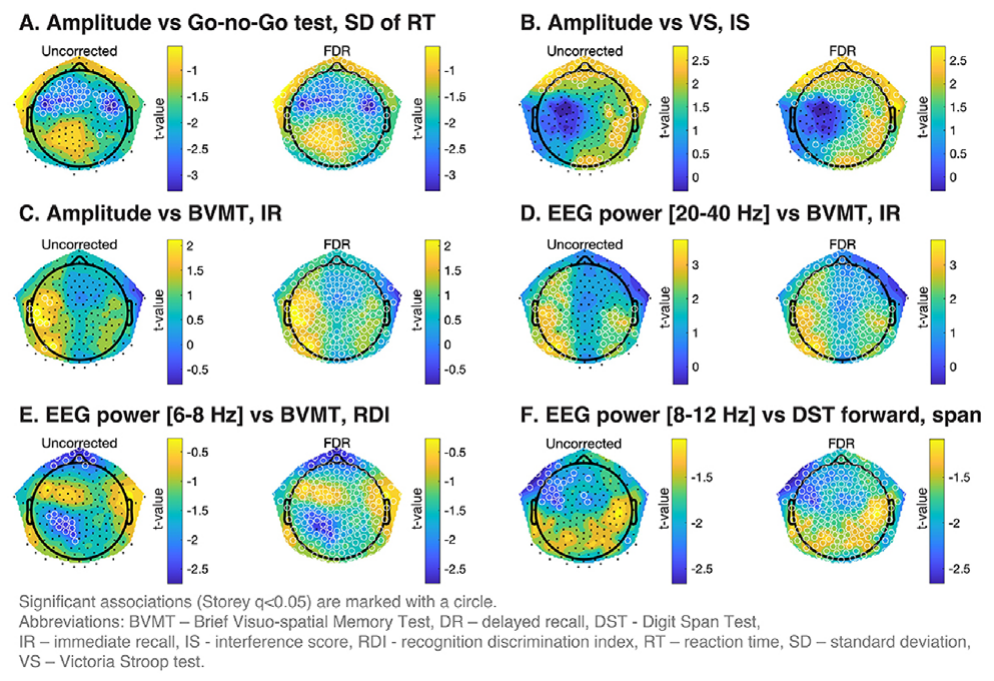
**FIGURE 3** Selected associations between STW and cognition (topographic analysis, *n* = 30).

**Conclusion:** This is the first description of the automatically detected STW in PSG and in hd‐EEG. STW activity appears to have distinct associations with cognition, showing a positive link with memory but a negative link with attention and executive functioning.

**Disclosure:** European Stroke Research Foundation 2021.

## OPR‐061

### Phenotypic traits of sleep‐apnea post‐stroke: Insights from the analysis of the pan‐European ESADA cohort

#### S. Baillieul ^1^; R. Tamisier^1^; J. Pépin^1^; F. Fanfulla^2^; D. Testelmans^3^; G. Trakada^4^; H. Gouveris^5^; P. Steiropoulos^6^; W. Randerath^7^; G. Parati^8^; M. Drummond^9^; S. Mihaicuta^10^; J. Hedner^11^; L. Grote^11^; S. Bailly^1^

##### ^1^Grenoble Alpes University, HP2 ‐ Inserm U1300, CHU Grenoble Alpes, Grenoble, France; ^2^stituto Scientifico di Pavia e Montescano IRCCS, Unità Operativa di Fisiopatologia Respiratoria e Medicina del Sonno, Pavia, Italy; ^3^Department of Pulmonology, Leuven University Center for Sleep and Wake Disorders (LUCS), University Hospitals Leuven, Leuven, Belgium; ^4^Division of Pulmonology, Department of Clinical Therapeutics, National and Kapodistrian University of Athens, Pulmonary Medicine, Athens, Greece; ^5^Department of Otolaryngology, Head and Neck Surgery & Sleep Medicine Center, University Medical Center Mainz, Mainz, 55131, Germany; ^6^Department of Pneumonology, Medical School, Democritus University of Thrace, 68100, Alexandroupolis, Greece; ^7^Clinic of Pneumology and Allergology, Center for Sleep Medicine and Respiratory Care, Bethanien Hospital, Solingen, Germany; ^8^Department of Cardiovascular, Neural and Metabolic Sciences, IRCCS Istituto Auxologico Italiano, Piazzale Brescia 20, 20149 Milan, Italy; ^9^Sleep and Non‐Invasive Ventilation Unit, Hospital São João, Porto Faculty of Medicine, Porto University, Porto, Portugal; ^10^Center for Research and Innovation in Precision Medicine of Respiratory Diseases, Department of Pulmonology, “Victor Babes” University of Medicine and Pharmacy Timisoara, Timisoara, Romania; ^11^Centre for Sleep and Wake Disorders, Institute of Medicine, Sahlgrenska Academy, Gothenburg University, Gothenburg, Sweden

**Background and Aims:** Obstructive sleep apnea (OSA) is highly prevalent post‐stroke and is associated with a worse prognosis. International guidelines recommend OSA screening post‐stroke, but epidemiological evidence of their application is lacking. We aimed to describe the prevalence and phenotypic traits of stroke patients in a prospective real‐life cohort of patients with suspected OSA.

**Methods:** Adult patients (age 18–80 years) with suspected OSA were prospectively included in the European Sleep Apnea Database (ESADA, 39 sleep medicine centers). Exclusion criteria are previous diagnosis of OSA, limited life expectancy, and alcohol or drug abuse. Demographic and anthropometric data, Epworth Sleepiness Scale score, and medical history, including stroke, were recorded. OSA diagnosis was based on polysomnography or cardio‐respiratory polygraphy.

**Results:** Among the 33,359 patients prospectively included between 2007 and 2022, 793 (2.4%) patients presented a history of stroke. Stroke patients were significantly older (median [IQR] age = 63.0[55.0;71.0] years vs. 54.0[44.0;62.0], *p* < 0.001), predominantly males (73.5% vs. 70.1%, *p* = 0.04), and presented a significantly higher apnea‐hypopnea index (26.7[13.0;47.0] events/h vs. 24.0[9.6;46.3], *p* = 0.003), and higher rate of comorbid insomnia (5.2% vs. 3.4%, *p* = 0.005). Stroke patients presented a higher prevalence of hypertension (68.6% vs. 43.7%), ischemic heart disease (21.7% vs. 7.7%), or diabetes (27.9% vs. 15.2%) (all *p* < 0.001). No difference was observed in body mass index, ESS scores, and prevalence of other sleep comorbidities.

**Conclusion:** Stroke patients referred to OSA screening present specific phenotypic traits, including greater OSA severity and prevalence of comorbid insomnia. Structured care pathways are required to improve OSA screening post‐stroke.

**Disclosure:** Nothing to disclose.

## Monday, June 23 2025

## Neuro‐oncology

## OPR‐062

### Deciphering glioblastoma metabolic signature: Molecular profiling and NADH‐FLIM imaging

#### A. Di Stefano ^1^; M. Morelli^2^; F. Lessi^2^; C. Sandro^2^; G. Ferri^2^; M. Menicagli^2^; P. Aretini^2^; F. Pieri^1^; L. Schweizer^3^; M. Ronellenfitsch^4^; N. Younan^5^; F. Ducray^6^; E. Tabouret^7^; S. Cuzzubbo^8^; F. Bielle^9^; M. Sanson^10^; A. Picca^10^; L. Garofano^11^; A. Iavarone^11^; C. Mazzanti^2^

##### ^1^Neurosurgery Division, Azienda USL Toscana Nord‐ovest, Spedali Riuniti di Livorno, Livorno, Italy; ^2^Fondazione Pisana per la Scienza, San Giuliano Terme, Italy; ^3^Molecular and Computational Neuropathology, Johann Wolfgang Goethe University Medical School, Institute of Neurology (Edinger Institute), Neuroscience Center, Frankfurt, Germany; ^4^Dr. Senckenberg Institute of Neurooncology, University Hospital Frankfurt, Goethe University, Frankfurt am Main, Germany; ^5^Neurology Department, Foch Hospital, Suresnes, France; ^6^Neuro‐Oncology Unit, Hospices Civils de Lyon, Lyon, France; ^7^Department of Neuro‐Oncology, Aix‐Marseille Université, CHU Timone, AP‐HM, Marseille, France; ^8^Neurology Department, APHP, University Hospital Saint Louis, Paris, France; ^9^Service de Neuropathologie, Hôpitaux Universitaires La Pitié Salpêtrière‐Charles Foix, AP‐HP, Sorbonne Université, Paris, France; ^10^Sorbonne Université, INSERM Unité 1127, CNRS UMR 7225, Paris Brain Institute, Paris, France, Paris, France; ^11^Sylvester Comprehensive Cancer Center, University of Miami Miller School of Medicine, Miami, FL, USA

**Background and Aims:** Recent breakthroughs in single‐cell analysis reveal distinct metabolic cellular states of glioblastoma (GBM) and allow the detection of GBMs which are dependent on oxidative phosphorylation (OXPHOS). The aims of the study were to explore the prevalence of molecular/metabolic subtypes of GBM and to evaluate the efficacy of Fluorescence Lifetime Imaging (FLIM) microscopy of NADH in discerning the metabolic subtype of FFPE GBM tumor tissue.

**Methods:** We analyzed 3’ mRNA NGS FFPE tumor tissue of IDHwt glioblastomas. We selected OXPHOS and glycolytic/plurimetabolic (GPM) cases for the NADH‐FLIM microscopy study on FFPE slides.

**Results:** RNA Sequencing of 95 newly diagnosed IDHwt GBM patients found 37 OXPHOS (39%), 16 GPM (15%), 4 NEU (4%), 27 not classifiable NC (28%) and 12 PPR cases (14%). Sixteen out of the 37 OXPHOS patients exhibited top‐scoring mitochondrial activity and were defined as OXPHOS_high. Longitudinal analysis of 11 pairs of GBM untreated and recurrent tumors found that 3 OXPHOS_high patients maintained the OXPHOS status at recurrence. We assessed FGFR3‐TACC3 (F3T3) status in 62 cases and we found that all 4 FGFR3‐TACC3+ overexpressed OXPHOS genes. Four OXPHOS patients and 3 GPM cases were selected for the NADH‐FLIM study: NADH bound/free curves significantly differed between metabolic subtypes [*p* < 0.0001]. PCA analysis of NADH FLIM features showed different clustering according to the OXPHOS and GPM subtype.

**Conclusion:** F3T3 fusions are tightly associated with the OXPHOS signature and the OXPHOS status can be maintained at recurrence. OXPHOS and GPM glioblastoma clusters significantly differed in NADH bound/free distribution curves. Supplementary cases are currently under study.

**Disclosure:** Nothing to disclose.

## OPR‐063

### Next steps after the INDIGO trial: May we use IDH‐inhibitors in oligodendrogliomas grade 3 after surgical resection?

#### E. Pronello ^1^; F. Bruno^1^; A. Pellerino^1^; B. Raschio^1^; E. Marchesani^1^; T. Sciortino^2^; M. Conti Nibali^2^; A. Gastino^3^; D. Garbossa^4^; L. Bello^2^; L. Bertero^5^; R. Soffietti^1^; R. Rudà^1^

##### ^1^Division of Neuro‐Oncology, Department of Neuroscience “Rita Levi Montalcini”, University and City of Health and Science Hospital, Turin; ^2^Neurosurgical Oncology Unit, “Galeazzi ‐ Sant'Ambrogio” IRCCS, Milan; ^3^Division of Radiotherapy, Department of Oncology; ^4^Division of Neurosurgery, Department of Neuroscience “Rita Levi Montalcini”, University and City of Health and Science Hospital; ^5^Pathology Unit, Department of Medical Sciences, University of Turin, Italy

**Background and Aims:** Oligodendrogliomas IDH‐mutant 1p19q‐codeleted grade 3 (OG3) are traditionally associated with worse outcome compared to OG2. Standard treatments include maximal safe resection followed by adjuvant radio‐chemotherapy, a standard of care established in the pre‐molecular era.

**Methods:** We retrospectively reviewed a dataset of patients with a diagnosis of molecularly defined OG3‐OG2 treated from 1996 to 2024 in our institution.

**Results:** We included 80 patients (OG2: 60%; OG3: 40%). Median age was 40 (OG2) and 49 years (OG3). Gross‐total resection prevailed among OG2 (31.3% vs 6.3%, *p* = 0.007). After surgery, 54.2% of OG2 underwent observation, while 45.8% received temozolomide (TMZ); 68.8% of OG3 received upfront TMZ and 31.3% underwent chemoradiation (RT+TMZ). Median follow‐up was 118 months with a median progression‐free survival (mPFS) of 53.9 in OG2 and 54.9 months in OG3. mPFS did not significantly differ in OG3 patients receiving TMZ upfront vs early RT+TMZ (66.2 vs 41.5 months). Median overall survival (mOS) was similar in OG2 and OG3 (237.6 months vs not reached, *p* = 0.326) and did not significantly differ among OG3 treated with TMZ upfront + RT at recurrence and those with early RT+TMZ after surgery (not reached vs 121.7 months, *p* = 0.866). Multivariable analysis showed histological grading did not significantly affect survival outcomes.

**Conclusion:** In our series, OG3 showed survival outcomes comparable to OG2 and those treated with TMZ upfront and RT at recurrence, had similar mPFS and mOS of those receiving early chemoradiation. These findings, along with results from the INDIGO trial on IDH inhibitors, suggest potential for IDH inhibitors in OG3 post‐surgical treatment.

**Disclosure:** Nothing to disclose.

## OPR‐064

### Rapid molecular classification of brain tumors through DNA methylation analysis with nanopore sequencing

#### E. Bondareva; S. Moser; T. Rötzer‐Pejrimovsky; R. Höftberger; C. Haberler; N. Amberg

##### Division of Neuropathology and Neurochemistry, Department of Neurology, Medical University of Vienna, Vienna, Austria

**Background and Aims:** DNA methylation is a key epigenetic marker in cancer diagnostics, reflecting somatically acquired and cell‐of‐origin changes. In brain tumors, methylation profiling aligns with histopathological classifications. Conventional diagnostics using hematoxylin‐eosin staining, immunohistochemistry (IHC), and molecular profiling via EPIC array or TSO500 panel sequencing take 2–4 weeks, delaying integrated tumor board diagnosis and precision therapy onset. Our study evaluates nanopore sequencing as a real‐time diagnostic tool, enabling molecular classification of brain tumors within 24 hours. This accelerates targeted IHC and allows us to obtain integrated diagnosis within approximately five days.

**Figure:**
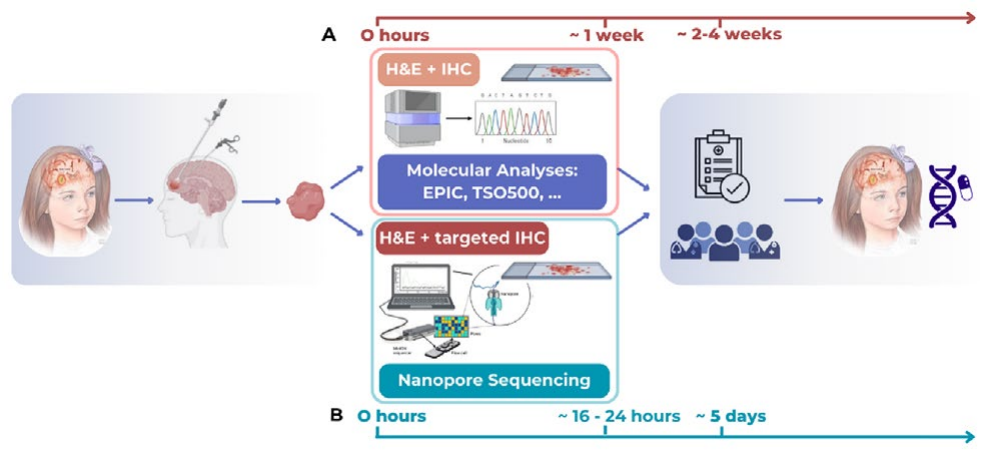
**FIGURE 1** A. Routine brain tumors neuropathological analysis, B. Nanopore sequencing workflow at our department. H&E: hematoxylin‐eosin staining, IHC: immunohistochemistry, EPIC: Infinium MethylationEPIC array, TSO500: TruSight Oncology 500 panel sequencing.

**Methods:** We analyzed 18 tumor samples using nanopore sequencing. DNA was extracted, quantified, and sequenced on the MinION device. Data analysis was performed using the NanoDX Pipeline (Euskirchen et al.), which includes base‐calling, mapping, quality control, copy number variations estimation, and methylation classification based on the Capper et al. reference cohort.

**Figure:**
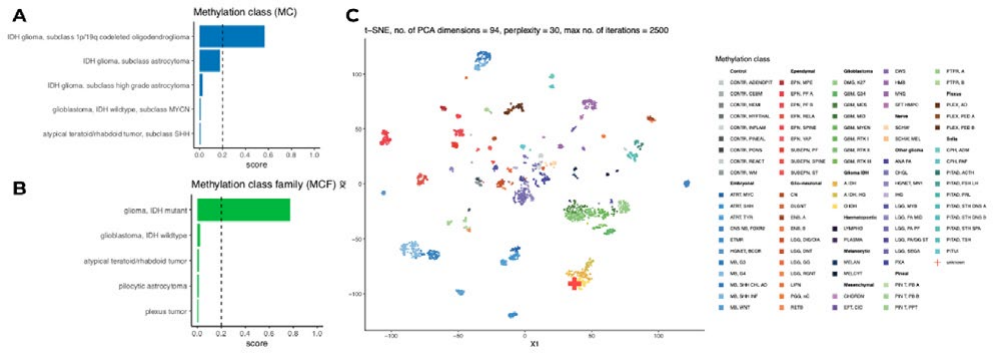
**FIGURE 2** A. Tumor Methylation classes and B. Methylation classes family with the highest prediction scores and recommended probability threshold >0.2. C. Dimensionality reduction plot indicating the tumor's location within the reference dataset of the classifier.

**Figure:**
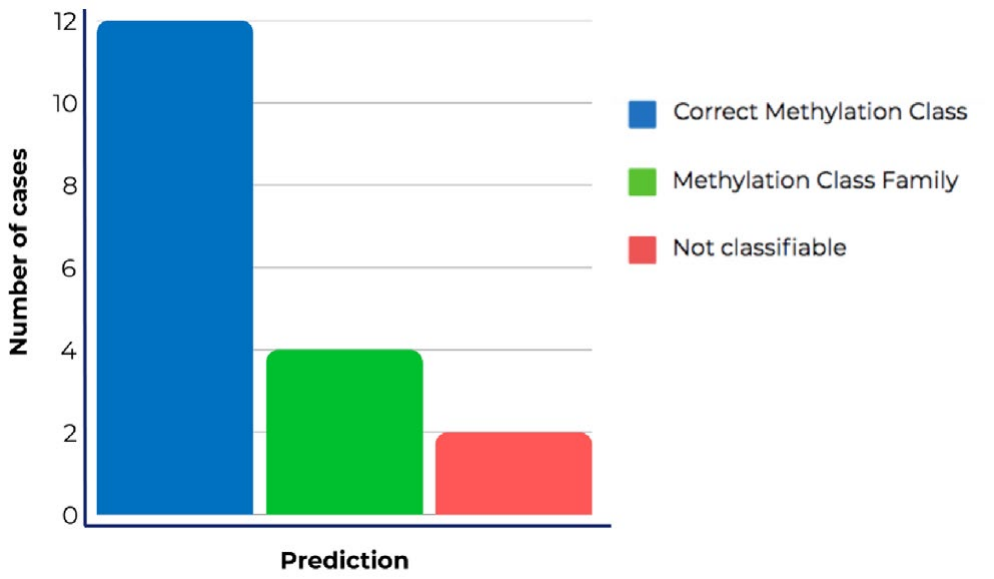
**FIGURE 3** Concordance of the nanopore classifier prediction with Infinium MethylationEPIC array‐based reference classifier.

**Results:** The nanopore classifier was concordant with the EPIC‐based reference in 12 cases (66.7%). In 4 cases (22.2%), the model predicted a different methylation class within the same methylation class family. In 2 cases (11.1%), no prediction was made due to low confidence.

**Conclusion:** Nanopore sequencing significantly reduces brain tumors diagnostic turnaround, potentially enabling precision therapy onset within five days. While promising, classifier refinement is needed for rare tumors and samples with low tumor fractions. Our ongoing research aims to determine whether the early achievement of integrated diagnosis leads to better clinical outcomes. Obtained results highlight the potential of real‐time sequencing to bridge the gap between traditional histology and precision oncology.

**Disclosure:** Nothing to disclose.

## OPR‐065

### Molecularly defined vs histologically defined glioblastoma: An AINO study

#### F. Bruno ^1^; A. Pellerino^1^; L. Bertero^2^; A. Silvani^3^; B. Pollo^4^; T. Ius^5^; S. Pizzolitto^6^; M. Conti Nibali^7^; G. Berzero^8^; P. Navarria^9^; L. Gurrieri^10^; A. Di Stefano^11^; M. Maccari^12^; G. Lombardi^12^; V. Internò^13^; E. Pronello^1^; M. Caffo^14^; Q. D'Alessandris^15^; R. Soffietti^1^; D. Garbossa^16^; L. Bello^17^; R. Rudà^1^

##### ^1^Division of Neuro‐Oncology, Department of Neuroscience ‘Rita Levi Montalcini’, University of Turin, Turin, Italy; ^2^Pathology Unit, Dept. of Medical Sciences, University and City of Health and Science, Turin, Italy; ^3^Department of Neuro‐Oncology, Fondazione IRCCS Istituto Neurologico Carlo Besta, Milano; ^4^Neuropathology Unit, Fondazione IRCCS Istituto Neurologico Carlo Besta, Milan, Italy; ^5^Neurosurgery Unit, Head‐Neck and Neuroscience Department, University Hospital of Udine, Piazzale S. Maria della Misericordia 15, 33100 Udine, Italy; ^6^Pathology Unit, University Hospital S. Maria della Misericordia ‐ Azienda Sanitaria‐Universitaria Friuli Centrale (ASU FC), p.le S. Maria della Misericordia, 15, 33100 Udine, Italy; ^7^Neurosurgical Oncology Unit, “Galeazzi – Sant’Ambrogio” IRCCS, Milan, Italy; ^8^Neurology Unit, IRCCS San Raffaele Scientific Institute, 20132 Milan, Italy; ^9^Department of Radiotherapy and Radiosurgery, IRCCS Humanitas Research Hospital, Via Manzoni, Rozzano, Milan, Italy; ^10^Clinical and Experimental Oncology, Immunotherapy, Rare Cancers and Biological Resource Center, IRCCS Istituto Romagnolo per lo Studio dei Tumori (IRST) “Dino Amadori”, 47014 Meldola, Italy; ^11^Neurosurgery Unit, Spedali Riuniti di Livorno, Usl Toscana Nord‐Ovest, Italy; ^12^Department of Oncology, Oncology 1, Veneto Institute of Oncology IOV‐IRCCS, Padua, Italy; School of Specialization in Medical Oncology, Department of Surgery, Oncology and Gastroenterology, University of Padua, Padua, Italy; ^13^Oncology Unit San Paolo Hospital, Bari, Italy; ^14^Department of Biomedical and Dental Sciences and Morphofunctional Imaging, Neurosurgical Clinic, University of Messina, 98125 Messina, Italy; ^15^UOC Neurochirurgia, Fondazione Policlinico Universitario Agostino Gemelli IRCCS, Università Cattolica del S. Cuore, Largo A. Gemelli, 8, 00168, Rome, Italy; ^16^Division of Neurosurgery, Dept. of Neuroscience “Rita Levi Montalcini”, University and City of Health and Science Hospital, Turin, Italy; ^17^Department of Oncology and Hemato‐Oncology, University of Milan, Italy

**Background and Aims:** Molecularly defined glioblastomas (mGBMs) are IDH‐wildtype astrocytomas with either EGFR amplification, pTERT mutation, or +7/‐10 chromosomal changes, regardless of histological grade. The Italian Association of Neuro‐Oncology (AINO) conducted a multicentric study to compare mGBMs with histologically defined glioblastomas (hGBMs).

**Methods:** This retrospective study compared 70 mGBM patients (with grade 2 histology and no enhancement on MRI) with a cohort of 66 hGBM patients.

**Results:** Median age of mGBM vs hGBM was similar (59 vs 60 years). Seizures prevailed in mGBMs (46/70, 66% vs 13/66, 20%). MGMT methylation rates were comparable (23/53, 43% vs 30/66, 45%). 23/59 (39%) mGBMs showed EGFR amplification and 55/66 (83%) pTERT mutation. After surgery, mGBMs received radiotherapy (RT) +/‐ temozolomide (TMZ) in 39 (56%), observation in 15 (21%), upfront TMZ in 13 (19%), while all hGBMs underwent RT/TMZ. Median progression‐free survival (mPFS) and overall survival (mOS) were longer in mGBMs (mPFS: 13 vs. 9 months, *p* = 0.011; mOS: 27 vs. 22 months, *p* = 0.036). MGMT methylation did not affect the mGBM outcome (mPFS: 13 months; mOS: 38 vs. 25 months, *p* = 0.291), but significantly influenced hGBM outcome (mPFS: 12 vs. 8 months, *p* < 0.001; mOS: 32 vs. 15 months, *p* < 0.001). Patients with isolated pTERT mutation showed better trends in mOS with RT/TMZ (45 vs. 17 months, *p* = 0.06).

**Conclusion:** In our study, GBMs had a higher incidence of seizures and significantly longer mPFS and mOS; also, MGMTp methylation did not affect their outcome. A trend for better mOS was seen in mGBM patients with isolated pTERT mutation after RT/TMZ.

**Disclosure:** Nothing to disclose.

## Ageing and Dementia 2

## OPR‐066

### The Triglyceride‐Glucose Index as predictor of cognitive decline in alzheimer's spectrum disorders

#### B. Gumina ^1^; A. Galli^3^; C. Tolassi^3^; G. Brolis^4^; I. Libri^1^; E. Bazzoli^5^; T. Outeiro^6^; S. Caratozzolo^1^; A. Pilotto^2^; A. Padovani^2^

##### ^1^Neurology Unit, Department of Clinical and Experimental Sciences, University of Brescia, Italy 2 Department of continuity of care and frailty, Neurology Unit, ASST Spedali Civili Hospital, Brescia, Italy; ^2^Neurology Unit, Department of Clinical and Experimental Sciences, University of Brescia, Italy, Brain Health Center, University of Brescia, Italy; ^3^Neurology Unit, Department of Clinical and Experimental Sciences, University of Brescia, Italy, Laboratory of Digital Neurology and Biosensors, University of Brescia, Italy; ^4^Neurology Unit, Department of Clinical and Experimental Sciences, University of Brescia, Italy; ^5^Nutri Neuro Med, Desenzano del Garda, Italy; ^6^University Medical Center Goettingen, Department of Experimental Neurodegeneration, Center for Biostructural Imaging of Neurodegeneration, Goettingen, Germany. 8 Translational and Clinical Research Institute, Faculty of Medical Sciences, Newcastle University

**Background and Aims:** Metabolic disorders influence Alzheimer's disease (AD) pathogenesis, but their impact on progression remains unclear. This study assessed insulin resistance as a progression marker.

**Methods:** Single‐center retrospective study (2014–2024) analyzed non‐diabetic neurodegenerative patients with a CSF based diagnosis of AD or other neurodegenerative conditions (NDD). Patients underwent baseline clinical, biochemical and follow‐up clinical assessment (≥ 6 months). TyG index stratified patients into tertiles (low, medium, high). Baseline clinical features and CSF biomarkers were compared using chi‐squared tests or non‐parametric ANCOVA. Cox regression models were implemented considering cognitive decline and disease progression as outcome (MMSE loss >2.5 points/year), adjusted for age, sex, MMSE at baseline, disease duration, AD therapy, and BMI.

**Results:** Final sample of 315 patients entered the study: 210 AD (mean age 71.51, Male % 79) and 115 NDD (mean age 69.19, % male 60). Only in AD high TyG was linked to worse BBB markers and interacted with the APOE ε4ε4, with no effect in NDD. AD patients with high TyG exhibited more cardiovascular risk factors, comparable baseline characteristics (sex, education, APOE genotype, and CSF biomarkers). During follow‐up, in the MCI‐AD subgroup (*n* = 161; 77% male), high TyG was significantly associated with faster cognitive decline over three years (HR = 4.08, 95% CI [1.06–15.73]). A similar, though non‐significant, trend was observed for MCI‐to‐dementia conversion (*p* = 0.086). No significant TyG‐APOE interaction was found for progression. TyG showed no impact on clinical progression in NDD group.

**Conclusion:** Insulin resistance predicts cognitive decline in early phases of AD, aiding risk stratification and guiding early interventions.

**Disclosure:** Nothing to disclose.

## OPR‐067

### Mortality after hip fracture surgery in patients with dementia: Large Swedish study of over 111 000 patients

#### D. Religa ^1^; M. Axenhus^2^; S. Hägg^3^; M. Hedström^4^; M. Eriksdotter^1^

##### ^1^Department of Neurobiology, Care Sciences and Society, Karolinska Institutet, Stockholm and Inflammation and Aging Theme, Karolinska University Hospital, Stockholm, Sweden; ^2^Karolinska Institutet, Stockholm, Sweden and Department of Orthopaedic Surgery, Danderyd Hospital, Stockholm, Sweden; ^3^Department of Medical Epidemiology and Biostatistics, Karolinska Institutet, Stockholm, Sweden; ^4^Department of Clinical Science, Intervention and Technology (CLINTEC), Karolinska Institutet, Stockholm and Trauma and Reparative Medicine Theme (TRM), Karolinska University Hospital, Stockholm, Sweden

**Background and Aims:** This study aimed to investigate whether dementia is associated with increased mortality following hip fractures and how different dementia subtypes affect mortality outcomes.

**Methods:** Utilizing data from the Swedish Hip Fracture Register (SHR), Swedish Registry for Cognitive/Dementia Disorders (SveDem), National Patient Register (NPR), and National Prescribed Drug Register (PDR), we conducted a retrospective analysis of 111,353 patients who underwent hip fracture surgery between 2010 and 2018. Multivariable Cox regression analyses were used to evaluate mortality risk factors.

**Results:** Of the study sample, 22% had dementia. Dementia patients exhibited higher mortality rates at 30 days with 13% vs. 6%, (*p* < .001), 4 months with 27% vs. 12%, (*p* < .001). and at 1 year with 39% vs. 20%, post‐fracture (*p* < .001). Higher ASA grades, poor baseline walking ability, and long‐term care residency were also associated with increased mortality. Parkinson´s disease dementia was associated with a higher mortality compared to other dementias during the first 4 months post operatively.

**Conclusion:** The study found that patients with dementia had significantly higher mortality rates at 30 days, 4 months, and 1‐year post‐hip fracture compared to those without a diagnosis of dementia. Subtypes such as Parkinson's disease dementia and dementia with Lewy bodies posed particularly high risks, highlighting the need for tailored postoperative care in these patients.

**Disclosure:** None

## OPR‐068

### Distinct resting‐state EEG biomarkers predict amyloid status and conversion to Alzheimer's dementia in a memory center

#### G. Cecchetti ^1^; S. Basaia^2^; M. Cursi^3^; E. Spinelli^4^; E. Canu^2^; F. Caso^5^; R. Santangelo^5^; G. Fanelli^3^; G. Magnani^5^; F. Agosta^4^; M. Filippi^6^

##### ^1^Neurology Unit, Neurophysiology Service, and Neuroimaging Research Unit, Division of Neuroscience, IRCCS San Raffaele Scientific Institute, and Vita‐Salute San Raffaele University, Milan, Italy; ^2^Neuroimaging Research Unit, Division of Neuroscience, IRCCS San Raffaele Scientific Institute, Milan, Italy; ^3^Neurophysiology Service, IRCCS San Raffaele Scientific Institute, Milan, Italy; ^4^Neuroimaging Research Unit, Division of Neuroscience, and Neurology Unit, IRCCS San Raffaele Scientific Institute, and Vita‐Salute San Raffaele University, Milan, Italy; ^5^Neurology Unit, IRCCS San Raffaele Scientific Institute, Milan, Italy; ^6^Neurology Unit, Neurorehabilitation Unit, Neurophysiology Service, and Neuroimaging Research Unit, Division of Neuroscience, IRCCS San Raffaele Scientific Institute, and Vita‐Salute San Raffaele University, Milan, Italy

**Background and Aims:** This study examined the diagnostic and prognostic potential of resting‐state EEG (RS‐EEG) biomarkers in Alzheimer's disease (AD) by differentiating patients based on cerebrospinal fluid (CSF) amyloid status and predicting the conversion of mild cognitive impairment (MCI) to AD dementia.

**Methods:** A total of 295 cognitively impaired patients were grouped by CSF β‐amyloid 42/40 ratio into A+ (*n* = 184) and A‐ (*n* = 111). Among them, 106 had MCI, further classified as MCI A+ (*n* = 61) and MCI A‐ (*n* = 45). A subset of 39 MCI A+ patients was tracked for two years, with 23 converting to AD dementia. RS‐EEG data were analyzed through current source densities (CSD) and linear lagged connectivity (LLC) within the Default Mode Network (DMN) and Salience Network (SN) using sLORETA. Support vector machine (SVM) analysis was applied to classify patients based on selected EEG features.

**Results:** In both the DMN and the SN, A+ patients showed a global slowing of cortical electrical activity; MCI A+ patients showed higher theta density and connectivity. MCI converters demonstrated reduced alpha density (DMN only) and lower alpha connectivity in both networks. An SVM model using the top five features achieved 60% accuracy in predicting MCI A+ conversion.

**Conclusion:** RS‐EEG biomarkers, notably theta CSD connectivity, are promising early AD indicators, with AI‐driven analysis further enhancing their potential. Alpha connectivity shows prognostic value for MCI‐to‐AD conversion. Larger studies with higher‐density EEGs are needed to validate these findings.

**Disclosure:** Funding. Next Generation EU/National Recovery and Resilience Plan, Investment PE8‐Project Age‐It.

## OPR‐069

### The two cut‐offs approach for plasma p‐tau217 to detect Alzheimer's Disease in preclinical and prodromal stages

#### G. Giacomucci ^1^; C. Crucitti^1^; A. Ingannato^1^; S. Bagnoli^1^; E. Marcantelli^1^; S. Padiglioni^2^; V. Moschini^3^; C. Morinelli^3^; S. Mazzeo^4^; S. Sorbi^5^; B. Nacmias^1^; V. Bessi^1^

##### ^1^Department of Neuroscience, Psychology, Drug Research and Child Health, University of Florence; ^2^Research and Innovation Centre for Dementia‐CRIDEM, AOU Careggi; ^3^SOD Neurologia, Dipartimento Neuromuscolo‐Scheletrico e degli Organi di Senso, AOU Careggi; ^4^IRCCS Policlinico San Donato, Milan; ^5^IRCCS Fondazione Don Carlo Gnocchi, Florence

**Background and Aims:** Plasma p‐tau217 is becoming a notable biomarker for its accuracy in detecting Alzheimer's Disease (AD) pathology even in preclinical stages. Our study aimed to try out the applicability of plasma p‐tau217 in the identification of patients with Subjective Cognitive Decline (SCD) and Mild Cognitive Impairment (MCI) carrying AD pathology in a real‐world setting.

**Methods:** We included 187 patients (50 SCD, 87 MCI and 50 AD‐demented) undergoing neurological and neuropsychological examination, CSF and blood collection to dose plasma p‐tau217 with Lumipulse G600II assay. Patients were classified according to the Revised Criteria of Alzheimer's Association Workgroup as Core1+ or Core1‐.

**Results:** MCI Core1+ had higher plasma p‐tau217 levels than MCI Core 1‐ (*p* < 0.001); just like SCD Core1+ compared to SCD Core1‐ (*p* = 0.023). Plasma p‐tau217 was highly accurate for discriminating between Core1+ and Core1‐ patients (AUC = 0.92) with an optimal cut‐off value of 0.274 pg/ml, revealing a good accuracy (86.29% [95%CI 81.20‐91.39]), PPV (88.18% [95%CI 83.40‐92.96]) and NPV (83.09% [95%CI 77.52‐88.63]). When applying a two cut‐offs approach (0.245 pg/ml and 0.516 pg/ml), plasma p‐tau217 showed higher accuracy (91.11% [95%CI 86.31‐95.91]), a PPV of 96.25% [95%CI 93.05.99.45] and a NPV of 83.63% [95%CI 77.40‐89.88%]).

**Figure:**
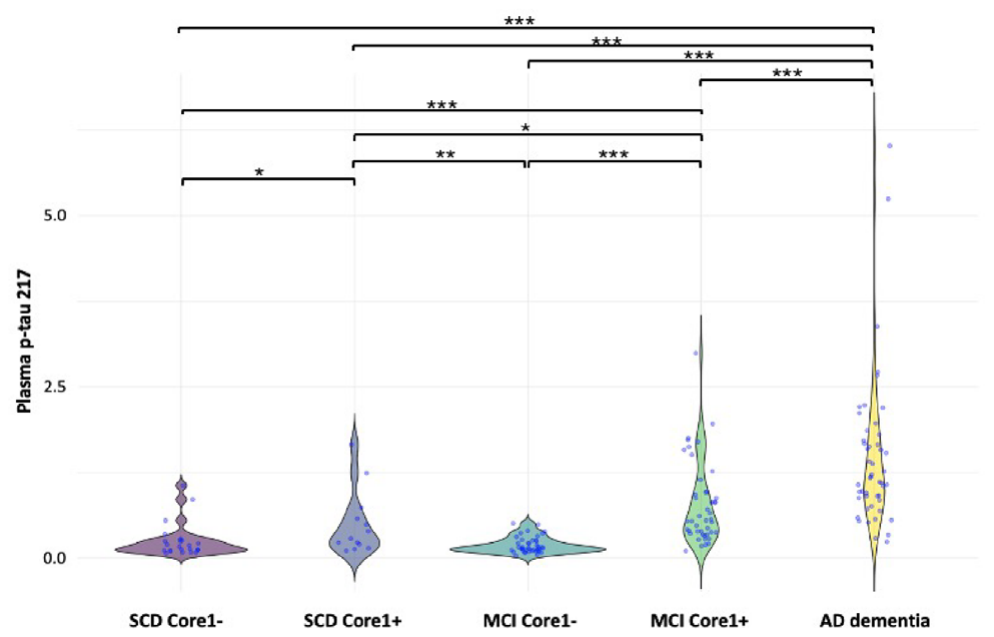
FIGURE 1

**Figure:**
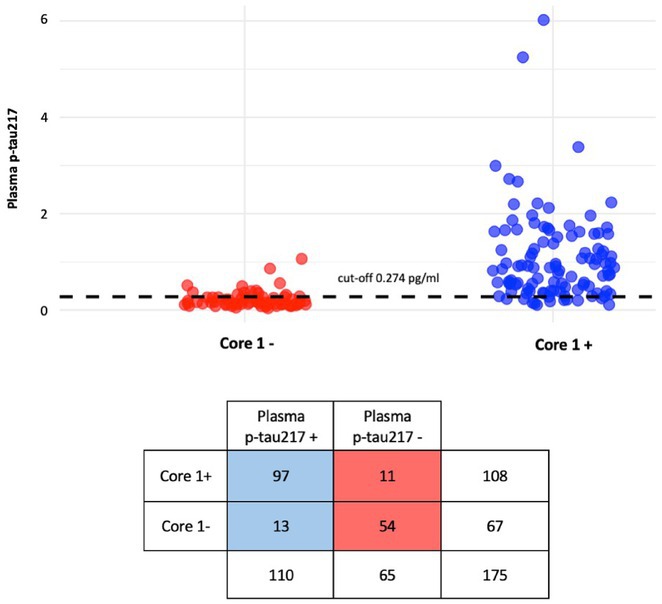
FIGURE 2

**Figure:**
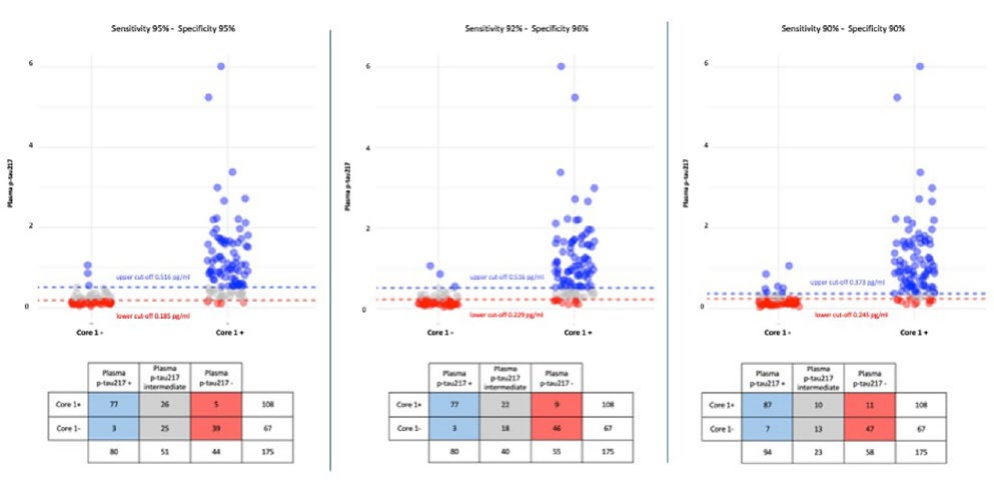
FIGURE 3

**Conclusion:** Plasma p‐tau 217 represents a meaningful biomarker to differentiate carriers of AD pathology from non‐carriers, also in the prodromal and preclinical stages of the disease, considering a real‐world population. The two cut‐offs approach provides for stronger accuracy, PPV and NPV than single cut‐off, making more reliable the clinical application of plasma p‐tau217 for the early detection of AD in real‐world settings.

**Disclosure:** Nothing to disclose.

## OPR‐070

### Progression of multimodal MRI biomarkers in Frontotemporal Dementia

#### M. Michelutti ^1^; H. Huppertz^3^; S. Anderl‐Straub^2^; D. Urso^4^; B. Tafuri^4^; S. Nigro^4^; P. Manganotti^1^; L. Werner^2^; J. Lombardi^2^; M. Otto^5^; G. Logroscino^4^; H. Müller^2^; J. Kassubek^2^

##### ^1^Neurology Unit, Department of Medical, Surgical and Health Sciences, University of Trieste, Trieste, Italy; ^2^Department of Neurology, University Hospital Ulm, Ulm, Germany; ^3^Swiss Epilepsy Clinic, Klinik Lengg, Zürich, Switzerland; ^4^Center for Neurodegenerative Diseases and the Aging Brain, University of Bari Aldo Moro at Pia Fondazione “Card. G. Panico”, Tricase, Italy; ^5^Department of Neurology, University Hospital Halle, Martin Luther University, Halle (Saale), Germany.

**Background and Aims:** We investigated longitudinal changes in brain white matter microstructure and gray matter volumetry in non‐fluent (nfPPA) and semantic (svPPA) variants of primary progressive aphasia (PPA), behavioral variant frontotemporal dementia (bvFTD), and bvFTD with motor neuron disease (bvFTD‐MND) using diffusion tensor imaging (DTI) and atlas‐based volumetry (ABV).

**Methods:** MRI datasets from 29 nfPPA, 27 svPPA, 65 bvFTD, 18 bvFTD‐MND patients and 39 controls, were analyzed. White matter fractional anisotropy (FA) was assessed in Tracts of Interest (TOIs) using Tract‐Wise Fractional Anisotropy Statistics (TFAS) and without a priori assumptions via Whole Brain‐based Spatial Statistics (WBSS). Gray matter volumetric differences in Regions of Interest (ROIs) were also calculated. Longitudinal scans from 10 nfPPA, 6 svPPA, and 19 bvFTD patients over 12 months were analyzed to assess progression. FA maps were correlated with FTLD‐CDR scores.

**Results:** At baseline, white matter degeneration was revealed in frontal, temporal, and callosal regions in nfPPA and in the inferior longitudinal fasciculus (ILF) in svPPA. bvFTD and bvFTD‐MND showed widespread FA reductions in the frontotemporal lobes and anterior corpus callosum, with additional corticospinal involvement in bvFTD‐MND. Longitudinally, nfPPA showed frontal and callosal progression, bvFTD exhibited progression along frontal, callosal, and posterior temporal tracts, while svPPA showed localized left ILF progression. Correlations with FTLD‐CDR scores were observed in left frontal (nfPPA), posterior temporal (svPPA, bvFTD), callosal (bvFTD) white matter as well as in the basal ganglia (bvFTD).

**Figure:**
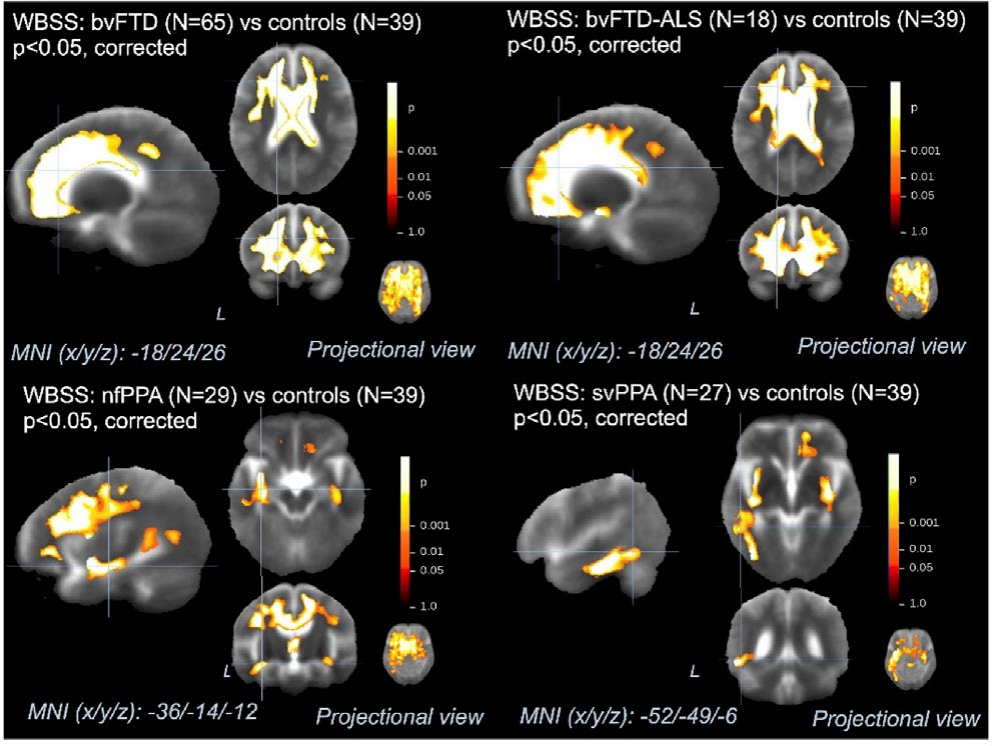
**FIGURE 1** Cross‐sectional Fractional Anisotropy Changes in bvFTD, bvFTD‐MND, nfPPA and svPPA compared to controls.

**Figure:**
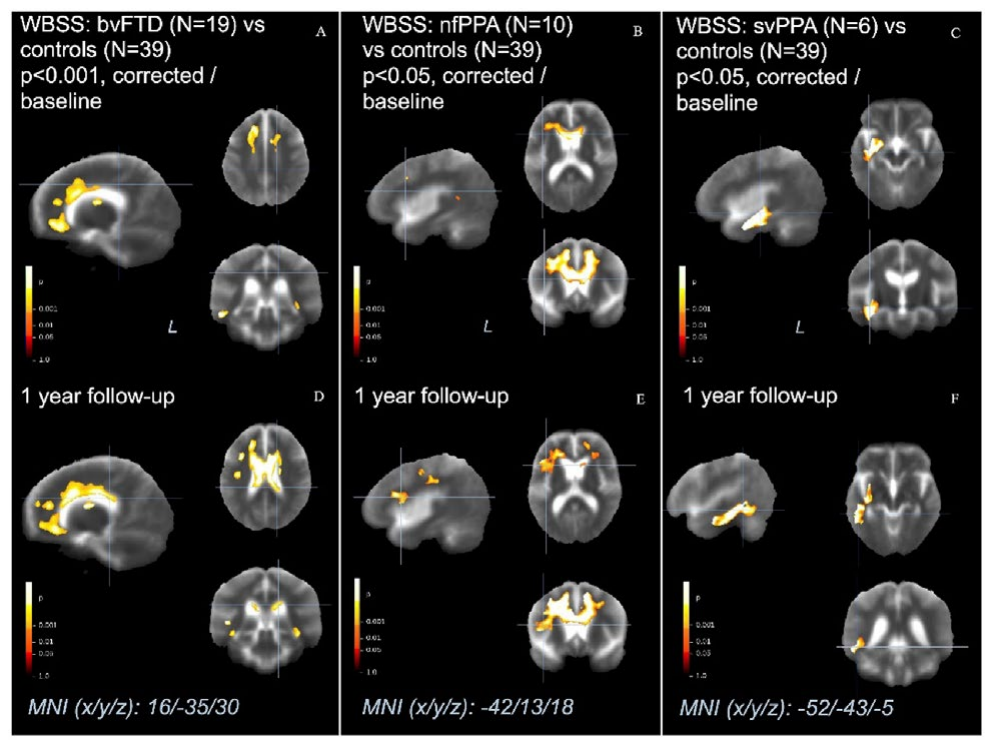
**FIGURE 2** Longitudinal Fractional Anisotropy Changes in bvFTD, nfPPA and svPPA compared to controls.

**Conclusion:** Distinct degeneration patterns emerged across syndromes, supporting early differential diagnosis and allowing tracking of disease progression.

**Disclosure:** This study was conducted as part of the first author's EAN Clinical Fellowship 2024.

## OPR‐071

### Preventing dementia in Italy: Estimations of modifiable risk factors and public health implications

#### S. Salemme ^1^; F. Asta^2^; G. Bellomo^2^; B. Contoli^2^; F. Lombardo^2^; V. Minardi^2^; N. Vanacore^2^; M. Masocco^2^

##### ^1^Department of Biomedical, Metabolic and Neural Sciences, University of Modena and Reggio Emilia, Modena, Italy; ^2^National Center for Disease Prevention and Health Promotion, Italian National Institute of Health, Rome, Italy

**Background and Aims:** Dementia represents a growing global public health challenge, with over 50 million cases worldwide in 2020. Preventing dementia by targeting modifiable risk factors is crucial, particularly in ageing populations like Italy. This study aimed to update Population Attributable Fractions (PAFs) and introduce Potential Impact Fractions (PIFs) for dementia risk factors, providing national and regional estimates to inform public health interventions.

**Methods:** Using 2017‐2019 data from two national surveillance systems, PASSI and PASSI d'Argento, we estimated PAFs for 11 modifiable risk factors identified by the 2020 Lancet Commission. PIFs were calculated to simulate dementia case reductions under partial risk factor reductions. Regional PAFs were compared with health policies outlined in Italian Regional Prevention Plans. Statistical analyses incorporated communality adjustments to account for interdependent risk factors.

**Results:** The combined national PAF was 39.6% (95% CI: 20.8‐55.9), with midlife hypertension (6.5%) and physical inactivity (5.8%) as leading contributors (Fig. 1). Cardiovascular factors explained over 50% of preventable cases. Regional PAFs ranged from 31.7% to 47.5%, showing a north‐south gradient (Fig. 2). A 10% reduction in risk factors could prevent 54,495 dementia cases nationally, with regional PIFs ranging from 3.7% to 6.0%. Significant disparities were found in regional health policy alignments with identified risk factors, particularly for air pollution (Fig. 3).

**Figure:**
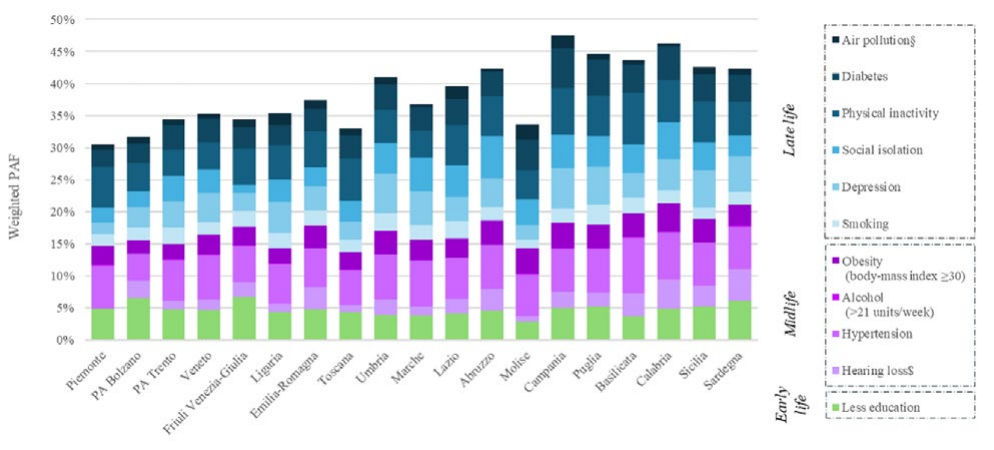
**FIGURE 1** Regional estimates of weighted Population Attributable Fractions of dementia cases. Years 2017‐2019.

**Figure:**
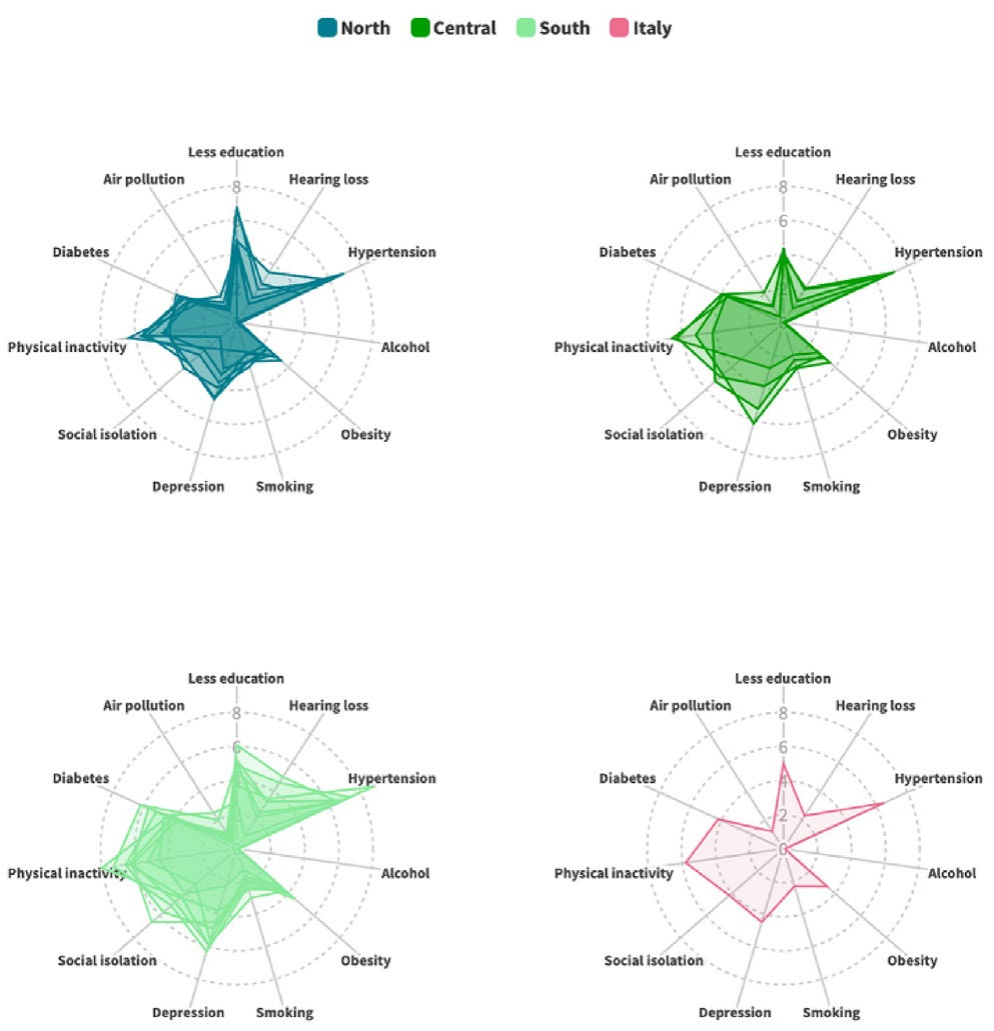
**FIGURE 2** Regional estimates of weighted Population Attributable Fractions of dementia cases by macroarea. Years 2017‐2019.

**Figure:**
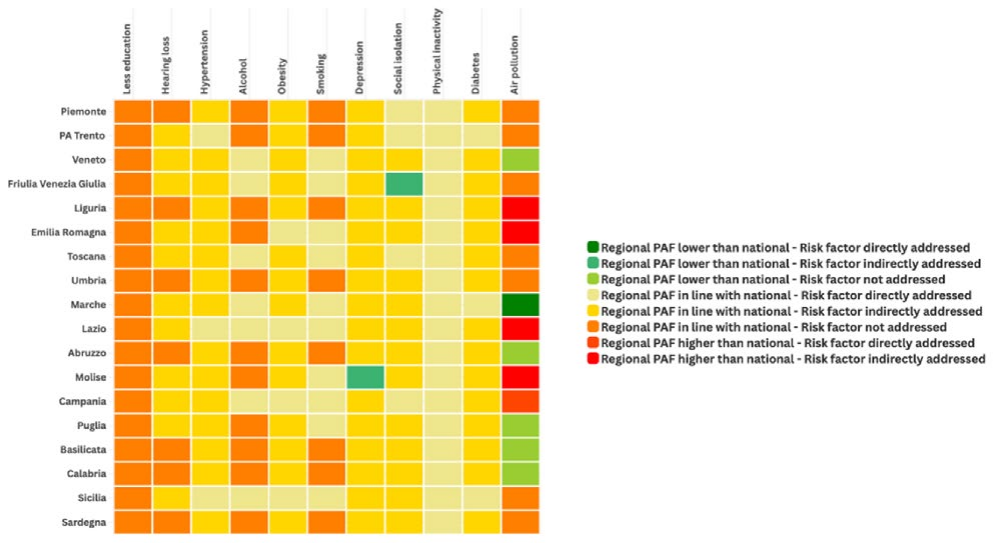
**FIGURE 3** Coherence between regional PAFs for dementia and population‐level interventions by risk factor.

**Conclusion:** This study underscores the potential to reduce dementia incidence in Italy through targeted interventions, particularly addressing cardiovascular risk factors. Regional variations in PAFs and policy alignment highlight the need for tailored, evidence‐based strategies.

**Disclosure:** This research was supported by the Italian Ministry of Health.

## Headache and Pain

## OPR‐072

### Calcitonin gene‐related peptide induces headache exacerbations in people with idiopathic intracranial hypertension

#### A. Yiangou ^1^; T. Do^2^; M. Thaller^1^; M. Byrne^3^; J. Mitchell^1^; G. Tsermoulas^3^; L. Hill^4^; S. Mollan^1^; S. Lucas^5^; M. Ashina^2^; A. Sinclair^1^

##### ^1^Metabolism and Systems Science, College of Medicine and Health, University of Birmingham, Birmingham, UK; ^2^Department of Neurology, Danish Headache Center, Copenhagen University Hospital—Rigshospitalet, Copenhagen, Denmark; ^3^Department of Neurosurgery, University Hospitals Birmingham NHS Foundation Trust, Birmingham, UK; ^4^School of Biomedical Sciences, Institute of Clinical Sciences, University of Birmingham, Birmingham, UK; ^5^School of Sport, Exercise and Rehabilitation Sciences, University of Birmingham, Birmingham, UK

**Background and Aims:** Calcitonin gene‐related peptide (CGRP), known for its role in migraine pathogenesis, may also underlie headache generation in idiopathic intracranial hypertension (IIH), which commonly presents with migraine‐like features.

**Methods:** A randomized, double‐blind, placebo‐controlled, two‐way crossover trial was conducted. Seventeen adults with IIH and no prior migraine were randomly assigned to receive a 20‐min continuous intravenous infusion of CGRP (1.5 μg/min) or placebo (isotonic saline) on two separate experimental days. Primary outcome was the difference in incidence of typical IIH headache exacerbations with migraine features between CGRP and placebo during a 12‐h observational period post‐infusion. Secondary outcomes were the differences in area under the curve (AUC) for headache intensity, intracranial pressure (ICP) and cerebrovascular haemodynamics.

**Results:** Twelve (71%) participants developed migraine‐like headaches after CGRP infusion, compared with three (18%) after placebo (*p* = 0.0077). The AUC for headache intensity was higher following CGRP infusion (*p* = 0.0157). Although the AUC of mean ICP remained unchanged, ICP amplitude increased significantly after CGRP (*p* = 0.0052). Cerebrovascular haemodynamics were significantly altered after CGRP (increased: heart rate (*p* < 0.0001), tissue oxygenation index (*p* = 0.0413), oxygenated haemoglobin (*p* < 0.0001) and decreased: mean arterial pressure (*p* = 0.0099), middle cerebral artery blood velocity (*p* = 0.0455)).

**Conclusion:** These findings suggest that CGRP is a potent inducer of migraine‐like headaches in IIH and may represent a promising therapeutic target.

**Disclosure:** A.Y. reports receiving speaker fees from Teva, UK. A.Y. is funded by an Association of British Neurologists and Guarantors of the Brain fellowship. S.P.M. has received honoraria for speaker events from Heidelberg engineering; Chugai‐Roche Ltd and Teva. Honoraria for advisory boards for Invex Therapeutics, Gensight and ocular therapeutix. Consultancy fees Neurodiem and Invex Therapeutics. Research funding from the UK Space Agency. M.A. has received personal fees from AbbVie, Amgen, Astra Zeneca, Eli Lilly, GlaxoSmithKline, Lundbeck, Novartis, Pfizer, and Teva Pharmaceuticals outside of the submitted work; has received research support from Lundbeck Foundation, Novartis, and Novo Nordisk Foundation; and has served as associate editor of Cephalalgia, associate editor of The Journal of Headache and Pain, and associate editor of Brain. A.J.S. is funded by a Sir Jules Thorn Award for Biomedical Science. A.J.S. reports personal fees from Invex therapeutics in her role as Director with stock holdings, during the conduct of the study; other from Allergan, Novartis, Cheisi and Amgen. All other authors declare no competing interests. All declared interests are outside the area of this submitted work.

## OPR‐073

### Insula deep brain stimulation for refractory neuropathic pain treatment: A randomized, sham‐controlled cross‐over trial

#### P. Cunha^1^; L. Dongyang^1^; J. Lapa^2^; G. Kubota^1^; J. Rosi Junior^1^; A. Fernandes^1^; R. Thibes^3^; D. Pinheiro^4^; R. Iglesio^1^; K. Duarte^1^; J. Sato^3^; V. Silva^1^; L. Lucato^1^; E. Figueiredo^6^; C. Carlotti Junior^6^; L. Yeng^1^; M. Teixeira^1^; D. Ciampi de Andrade ^7^

##### ^1^Pain Center, Department of Neurology, University of São Paulo, São Paulo, Brazil; ^2^Department of Medicine, Federal University of Sergipe, Aracaju, Sergipe, Brazil Neurosurgery Unit, Hospital de Cirurgia, Aracaju, Sergipe, Braz; ^3^Center for Computing Mathematics and Cognition of the Federal University of ABC, Santo André, Brazil; ^4^Universidade Federal de São Paulo | UNIFESP Departamento de Neurologia e Neurocirurgia; ^6^Department of Neurosurgery, University of São Paulo, São Paulo, Brazil; ^7^Center for Neuroplasticity and Pain (CNAP), Department of Health Science and Technology, Faculty of Medicine, Aalborg University, Aalborg, Denmark

**Background and Aims:** Neuropathic pain (NeP) affects a considerable portion of the population and is often refractory to pharmacological interventions, which makes important to find alternative treatments. This study investigated the safety and efficacy of deep brain stimulation (DBS) targeting the posterior‐superior insula in patients with chronic NeP who had previous response to repetitive transcranial magnetic stimulation of the same region.

**Methods:** A phase II randomized, double‐blind, sham‐controlled, cross‐over trial was performed with ten participants. DBS electrodes were stereotactically implanted in posterior‐superior insula. The study had three phases: double‐blind (three months), single‐blind (three months), and open‐label (six months). Pain intensity was measured using a verbal numeric rating scale. The primary outcome was defined as achieving a > = 30% reduction in average pain intensity. Secondary outcomes included assessments of pain interference, quality of life, and neuropsychiatric issues.

**Results:** Active DBS resulted in an 82.3% likelihood of achieving the primary outcome, with long‐term responders reporting a mean pain reduction of 81.3%. Significant improvements in pain‐related interference with sleep and mood were found, with probabilities exceeding 95% during follow‐up. Quality‐of‐life scores, specially the related to physical health, also improved significantly. No major adverse events occurred, and the intervention was well tolerated.

**Figure:**
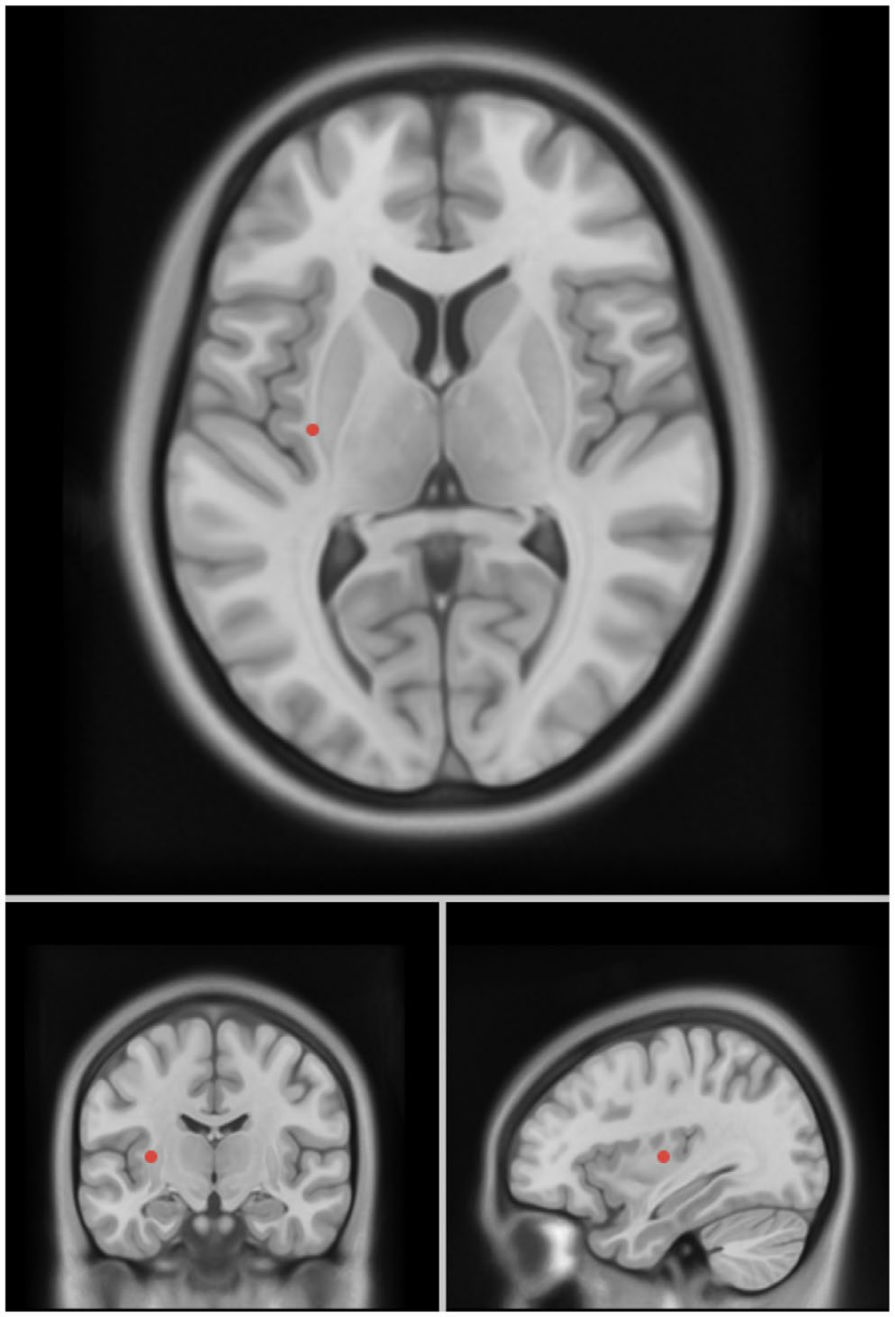
**FIGURE 1** “Hot spot” for PSI‐DBS

**Figure:**
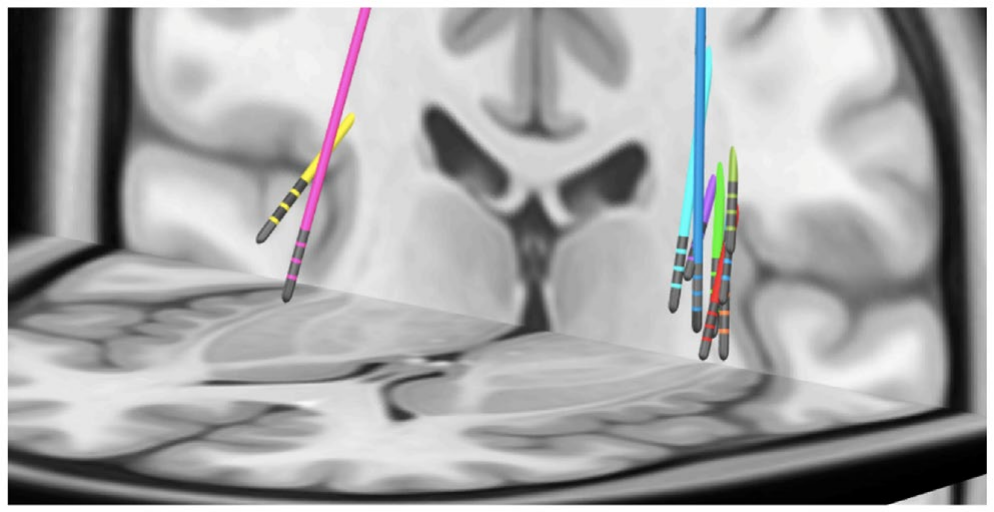
**FIGURE 2** Electrodes reconstruction scenario

**Conclusion:** These findings suggest that posterior‐superior insula DBS is a feasible and promising treatment for chronic refractory neuropathic pain, with a favorable safety profile. Larger phase III trials are recommended to confirm efficacy and assess broader applicability.

**Disclosure:** Nothing to disclose.

## OPR‐074

### Occipital nerve stimulation for chronic cluster headache: A double‐blind, randomized, placebo‐controlled study

#### I. Fogh‐Andersen ^1^; J. Sørensen^1^; A. Pedersen^2^; R. Jensen^2^; K. Meier^1^

##### ^1^Center for Experimental Neuroscience (CENSE), Department of Clinical Medicine, Aarhus University Hospital, Aarhus, Denmark; ^2^Danish Headache Centre, Righospitalet‐Glostrup, Copenhagen, Denmark

**Background and Aims:** Chronic cluster headache (CCH) is a debilitatingly painful disorder that can be very difficult to treat sufficiently. Occipital nerve stimulation (ONS) has shown promising results in attack prevention in patients with CCH, but evidence from controlled trials is scarce. Conventional (tonic) ONS elicits paresthesias, hampering blinded comparison to placebo. Using paresthesia‐free burst ONS, we conducted a randomized, placebo‐controlled trial.

**Methods:** The study is an investigator‐initiated, double‐blind, randomized, placebo‐controlled clinical trial involving patients with CCH. It comprised a four‐week baseline, a 12‐week trial with transcutaneous electrical nerve stimulation, ONS implantation, a 12‐week randomized, double‐blind burst ONS treatment period, and a 12‐week open‐label tonic ONS treatment period. The primary outcome was the proportion of participants reporting a ≥30% reduction in attack frequency in the randomized and open‐label trial phases.

**Results:** Thirty‐eight patients underwent ONS implantation and were randomly assigned to burst ONS (*n* = 19) or placebo (*n* = 19). After the randomized trial phase, the proportion of ≥30% responders was 18.81% (95%CI 0.28%‐37.87%) in the burst ONS group and 50.02% (26.87%‐73.09%) in the placebo group. The likelihood of reaching the primary endpoint was 31.20% (1.29%‐61.23%, *p* = 0.042) higher in the placebo group. After the open‐label phase, 42.09% (19.91%‐64.34%) in the burst ONS group and 51.11% (27.32%‐74.88%) in the placebo group had ≥30% frequency reduction.

**Conclusion:** ONS reduced attack frequency but was not superior to placebo. The results indicate that a part of the preventive effect of ONS may be attributed to a placebo response and call for attention to sufficient placebo control when planning further studies.

**Disclosure:** The study was funded by a grant from the Novo Nordisk Foundation (NNF19OC0058805). JCHS has received a restricted research grant (for the institution) from the Novo Nordisk Foundation. KM has received teaching fees from Medtronic and consulting fees from Salvia BioElectronics. KM and JCHS are co‐owners and co‐founders of the neuromodulation database company Neurizon. RJ received restricted research grants (for the institution) from Lundbeck Pharma and the Novo Nordisk Foundation. RJ has received personal fees for educational and teaching activities from Pfizer, Teva, Novartis, Abbvie, Lundbeck Pharma, and Eli‐Lilly, and a fee (for the institution) for serving on the Lundbeck Pharma Advisory Board. RJ is the chair of the Master of Headache Disorders, Director of the Danish Headache Center, and unpaid activities as Director in Lifting the Burden. ASP has received a restricted research grant (for the institution), conference attendance from Lundbeck Pharma, and personal fees from Pfizer for teaching activities. ISFA has nothing to declare.

## OPR‐075

### PACAP‐38 is increased in cluster headache; A new treatment target? A prospective, case‐control study

#### M. Kulas Søborg ^1^; N. Lund^1^; A. Snoer^1^; M. Barloese^2^; R. Højland Jensen^1^; A. Petersen^1^

##### ^1^Danish Headache Center, Department of Neurology, University of Copenhagen, Rigshospitalet‐Glostrup, Glostrup, Denmark; ^2^Centre for Functional and Diagnostic Imaging and Research, Hvidovre Hospital, Hvidovre, Denmark

**Background and Aims:** Pituitary adenylate cyclase‐activating peptide‐38 (PACAP‐38) is an essential neuropeptide in central nociception but estimates of its activity in cluster headache is sparse. We aimed to investigate whether plasma‐levels of PACAP‐38 differ between disease states (i.e., bout, remission, chronic) and compared to headache‐free controls. Additionally, we assessed a possible correlation between plasma‐levels of PACAP‐38 and calcitonin gene related peptide (CGRP).

**Methods:** Emanating from the Danish cluster headache biobank, plasma samples collected from 312 participants were analyzed for plasma‐levels of PACAP‐38 and CGRP in a prospective, observational, case‐control‐study. Headache‐free controls and participants with chronic cluster headache were sampled once, while participants with episodic cluster headache were sampled twice (in‐ and out of bout). Plasma‐levels were measured with validated immunoassays.

**Results:** Plasma derived from 205 patients with cluster headache according to ICHD‐3‐criteria and 101 sex‐ and age‐matched headache‐free controls. PACAP‐38 plasma‐levels were significantly higher in all three disease states of cluster headache as to headache‐free controls with collectively a mean PACAP‐level 34.3% (95%CI: 20.1‐48.6%, *p* < 0.0001) higher than controls. We did not demonstrate a correlation between plasma‐levels of PACAP‐38 and CGRP (Spearman's *r* = 0.08, *p* = 0.10).

**Conclusion:** This large‐scale study demonstrated increased PACAP‐38 levels in all disease states of CH compared to headache‐free controls, strengthening the hope of a possible effect of PACAP‐38‐targeting treatment in future trials.

**Disclosure:** The study was funded partly by an investigator‐initiated grant from Lundbeck Pharma. Lundbeck Pharma paid for the analyses of the samples. Payments were made directly to the laboratorium, Celerion, Switzerland. Lundbeck Pharma did not influence the design, conduct, or interpretation of this study.

## OPR‐076

### Inflammatory biomarkers are affected in cluster headache

#### N. Lund ^1^; C. Westgate^2^; M. Søborg^1^; T. Hansen^3^; R. Jensen^1^; A. Petersen^1^

##### ^1^Danish Headache Center, Dept. of Neurology, Rigshospitalet‐Glostrup, University of Copenhagen, Denmark; ^2^Danish Headache Center, Dept. of Neurology, Rigshospitalet‐Glostrup, University of Copenhagen, Denmark. Translational Research Center, Rigshospitalet, Glostrup, Denmark; ^3^Danish Headache Center, Dept. of Neurology, Rigshospitalet‐Glostrup, University of Copenhagen, Denmark and Neuogenomic, Translational Research Centre, Rigshospitalet Glostrup, Denmark

**Background and Aims:** The role of the inflammatory system in cluster headache (CH) has long been a topic of debate. Only few methodologically solid studies exist, and their findings are diverging. We therefore aimed to explore its role by measuring 45 different cytokines in a large cohort of CH patients and controls.

**Methods:** People with episodic CH (ECH) and chronic CH (CCH) from the Danish Cluster Headache Biobank were included and compared with headache‐free controls, matched for age and sex. Serum was analyzed by the validated Olink Target 48 Cytokine kit.

**Results:** In total, 99 CCH patients, 110 ECH patients both in bout and in remission, and 100 controls partici‐pated. For all patient groups, oncostatin m, a pro‐inflammatory cytokine, was elevated compared with controls (2.87 pg/ml (*p* < 0.0001), 3.06 pg/ml (*p* < 0.0001), 2.99 pg/ml (*p* < 0.001) and 1.91 pg/ml for CCH, ECH bout, ECH remission and HC, respectively). Additionally, the cytokine profile of CCH and ECH in bout exhibited distinct alterations from controls with overall elevated cytokine levels for CCH and overall reduced cytokine levels for ECH (Figure 1).

**Figure:**
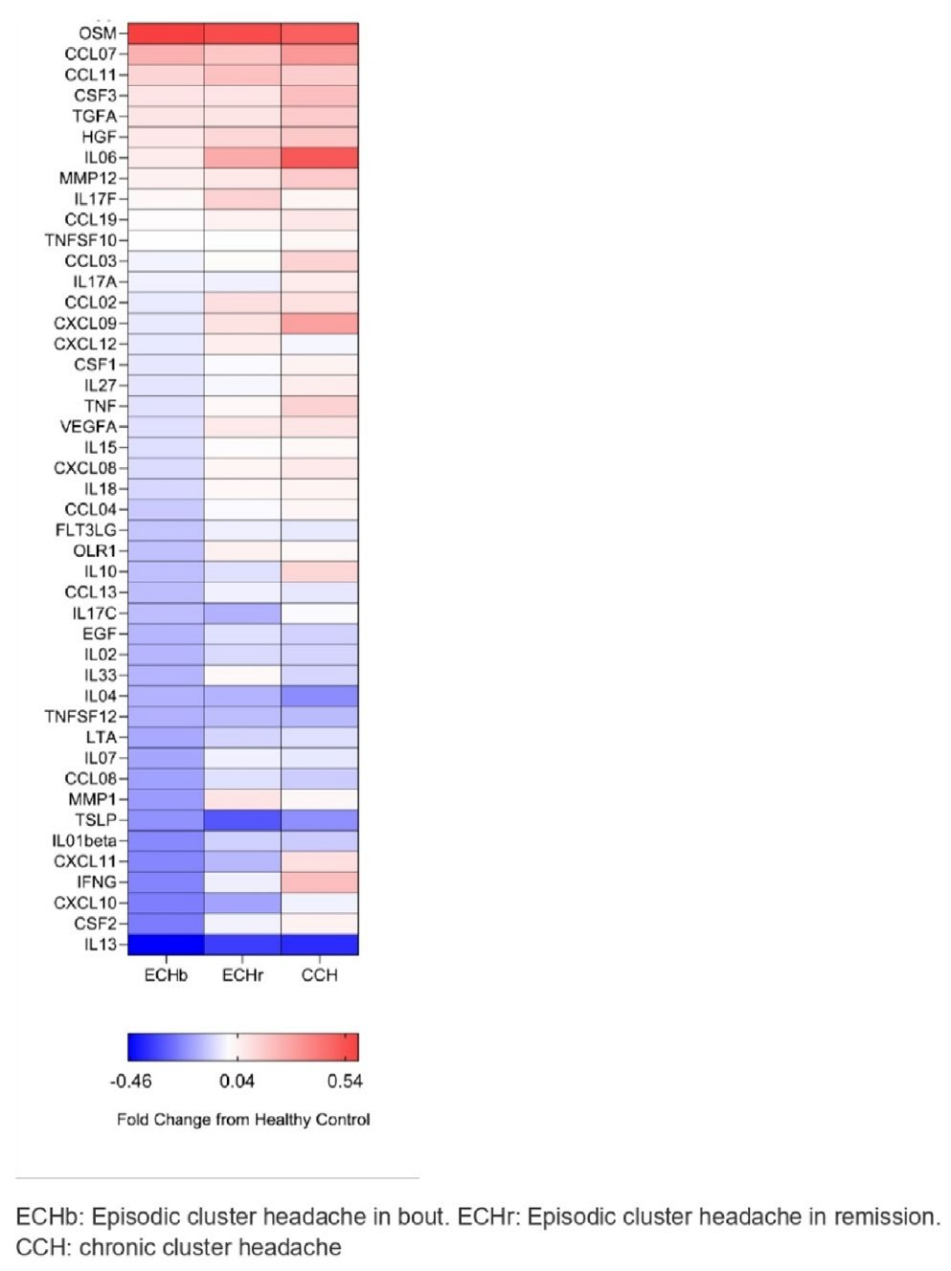
**FIGURE 1** Heat‐map showing the median fold change of cytokines between different stages of cluster headache and matched headache‐free controls.

**Conclusion:** In this large study of inflammatory biomarkers, we confirm the involvement of the inflammatory system in CH. With its receptors’ presence in the trigeminal ganglion, we identified oncostatin m as a potential new target of interest. The distinct alterations between CCH and ECH were unexpected and may have clinical implications in relation to treatment and prognosis.

**Disclosure:** Lundbeck Pharma financed the analyses of the samples, but had no influence on sample handling, data analyses nor interpretation of results and abstract writing. NL has received a personal research grant from the Capital region of Denmark.

## OPR‐077

### Exploration of prolonged remission and the natural course of cluster headache: An interview‐based cohort study

#### W. Naber ^1^; P. van Tilborg^1^; A. Zuidgeest^1^; L. Wilbrink^2^; W. Mulleners^3^; R. Brandt^1^; R. Fronczek^1^

##### ^1^Department of Neurology, Leiden University Medical Center (LUMC), Leiden, The Netherlands; ^2^Department of Neurology, Zuyderland Medical Center, Sittard‐Geleen, The Netherlands; ^3^4. Department of Anesthesiology, Expertise Center for Pain & Palliative Medicine, Radboud University Medical Center, Nijmegen, The Netherlands

**Background and Aims:** This study aims to gain insight into the intriguing yet sparsely documented phenomenon of cluster headache (CH) remission.

**Methods:** In this cross‐sectional cohort study all persons with CH were invited to complete a screening survey. Participants in prolonged remission were invited for an interview. Prolonged remission was defined as (i) no current CH prophylactic treatment and (ii) an attack free period of ≥ 5 years and/or twice the mean between‐episode time.

**Results:** Of the invited persons, 43.2% (778/1801) responded, 625 were included in survey analysis and 125 met prolonged remission‐criteria during interview. Median age at CH onset was 29 years (IQR: 20‐42) and at remission onset 55 years (48‐63). CH lasted 23 (15‐33) years before remission. In 62% (N = 78), remission occurred abruptly. Of those with gradual remission (38%, N = 47), attack frequency (65%) and intensity (59%) decreased and between‐episode intervals increased (52%) prior to remission. A higher probability of prolonged remission was observed in participants with ECH (hazard ratio (HR) = 6.60), who had quit smoking (HR = 2.53), had a higher attack intensity (HR = 1.28) and a higher age of CH onset (HR = 1.05).

**Figure:**
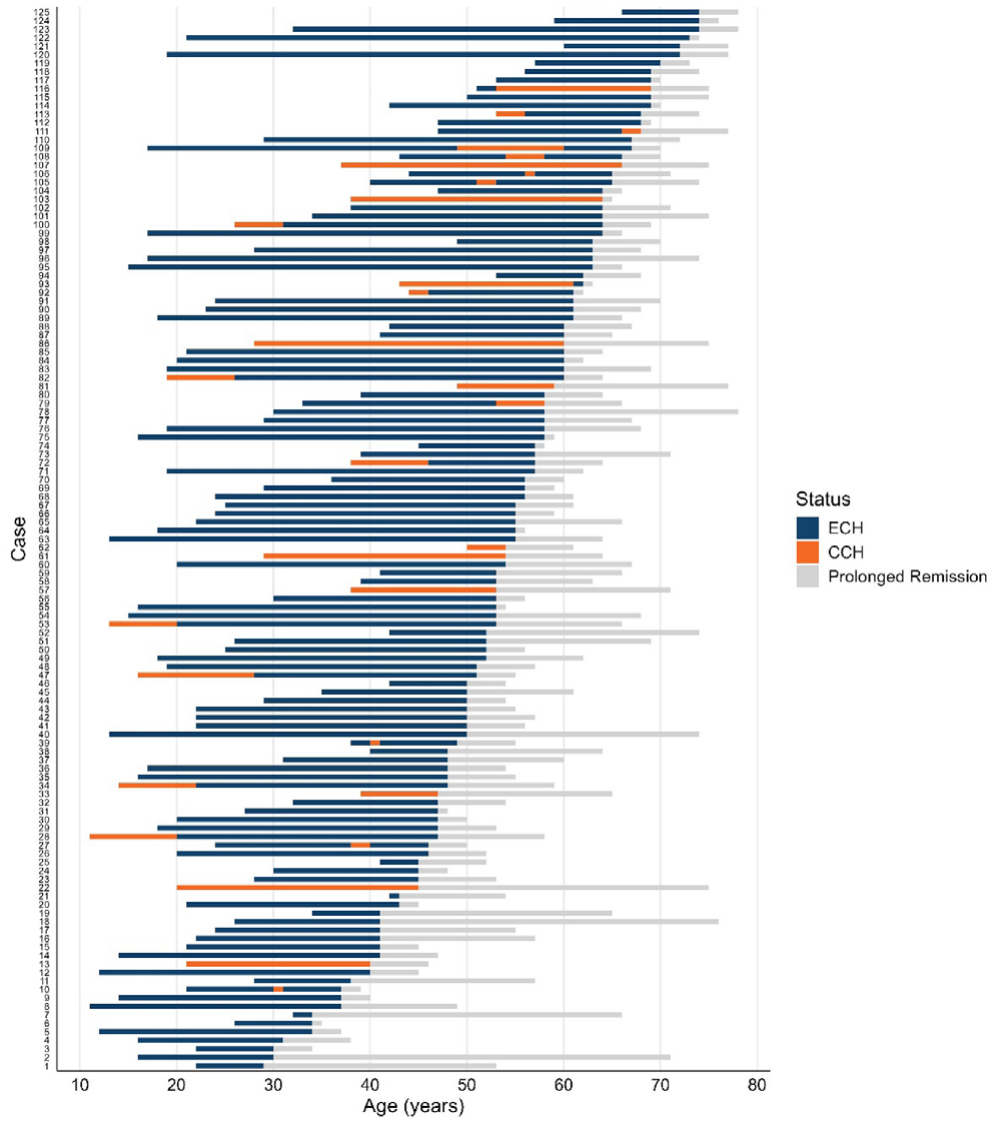
**FIGURE 1** Disease course of interviewed participants with prolonged remission.

**Figure:**
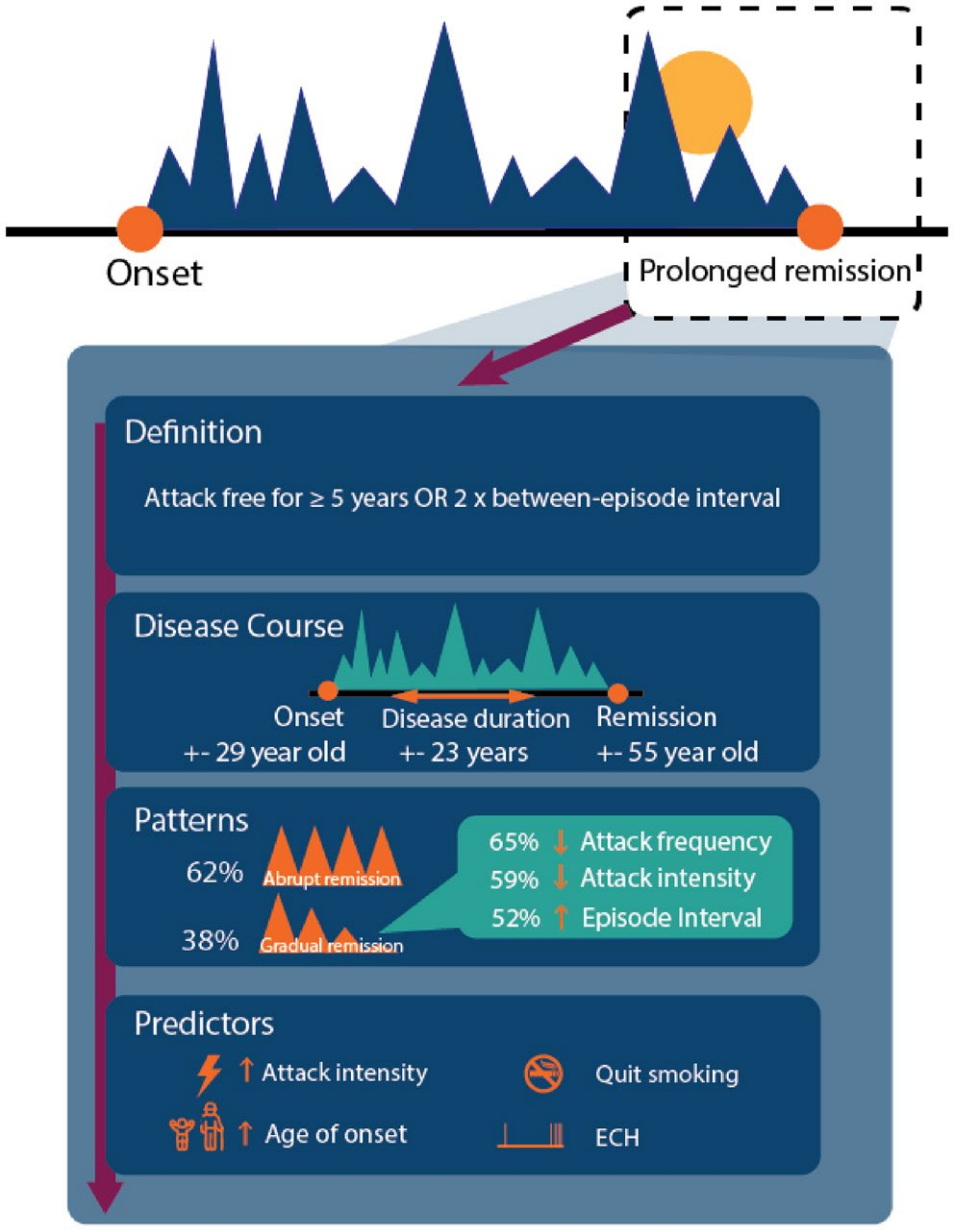
**FIGURE 2** Infographic on Prolonged remission in Cluster headache.

**Figure:**
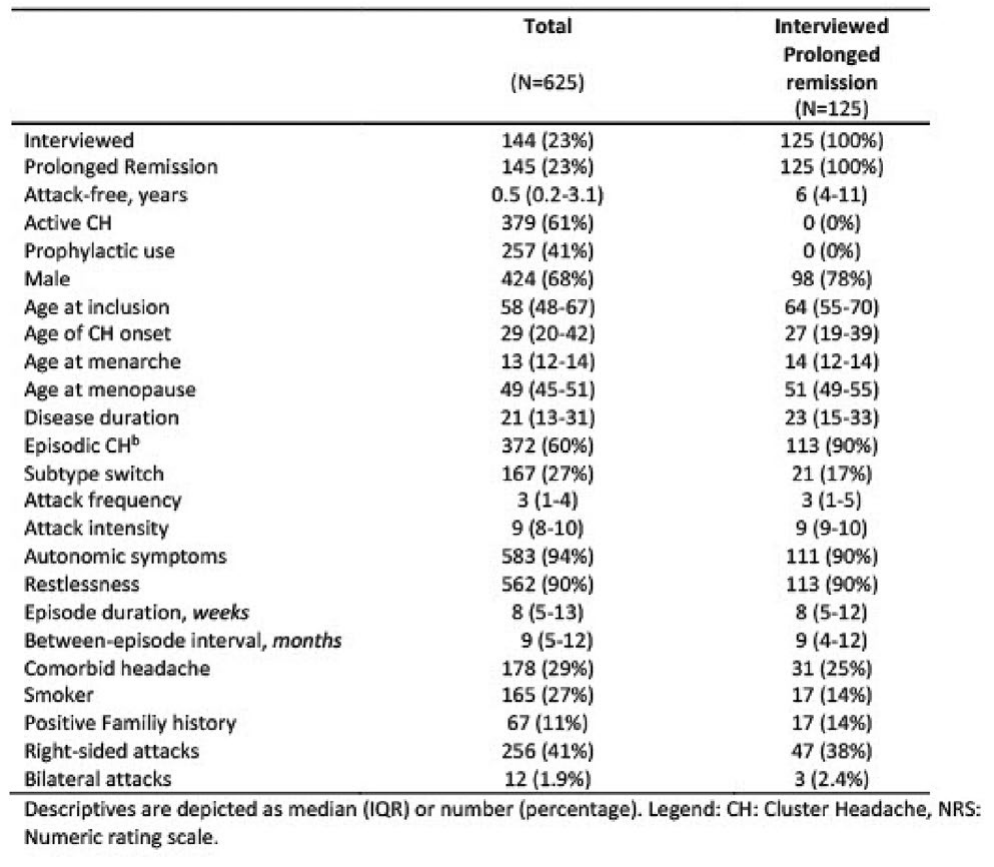
**TABLE 1** Baseline characteristics for participants.

**Conclusion:** This cohort provides a rare insight in prolonged CH remission and showed (i) an average age of CH onset around 30 years, (ii) with 25 years of active CH before (iii) start of remission when patients reach their mid‐50s. Disease duration until prolonged remission was shorter in episodic patients who had a high attack intensity, were older at the onset of CH, and had quit smoking.

**Disclosure:** The authors report no competing interests. RF reports consultancy and lecture fees from Novartis, Lundbeck, AbbVie, Lilly and TEVA, and independent support from the Dutch Brain Foundation, Leiden University Fund and Innovation Fund Dutch Healthcare Providers; these disclosures are not relevant for the topic of this abstract.; WM, PW, WN, PvT, AZ and RB report no relevant conflict of interest.

## MS and Related Disorders 2

## OPR‐078

### Soluble factors may contribute to broad rim lesion‐formation in multiple sclerosis

#### L. Lütje^1^; M. Martire^2^; H. Engelenburg^1^; C. Hsiao^1^; J. Hamann^1^; L. Klotz^3^; T. Kuhlmann^4^; I. Huitinga^1^; J. Smolders ^5^

##### ^1^Neuroimmunology research group, Netherlands Institute for Neuroscience, Amsterdam, The Netherlands; ^2^Neurology Unit, IRCCS San Raffaele Scientific Institute, Milan, Italy; ^3^Department of Neurology with Institute of Translational Neurology, University Hospital Münster, Münster Germany; ^4^Institute of Neuropathology, University Hospital Münster, Münster Germany; ^5^Departments of Neurology and Immunology, MS center ErasMS, Erasmus MC, University Medical Center Rotterdam, Rotterdam, The Netherlands

**Background and Aims:** Disability progression in multiple sclerosis (MS) is resistant to current therapies. We recently identified mixed active/inactive white matter lesions with a broad rim of HLA‐positive myeloid cells (broad rim lesions; BRLs) as an imageable biomarker of a severe MS course, which could disclose modifiable mechanisms.

**Methods:** We performed a comprehensive histological analysis of lesion rim thickness of BRLs (*n* = 94) and classical mixed active/inactive lesions (CL, *n* = 285) across MS cases (*n* = 52) within the Netherlands Brain Bank and explored correlations with pathological and clinical traits and lymphocyte‐presence.

**Results:** BRLs displayed broader HLA‐positive rims compared to classical mixed lesions (average 1,646 μm vs. 342 μm), with rim thickness across multiple lesions being identified as donor trait (hence referred to as BRL donors). Donors with largest rim sizes reached at earlier age disability milestones as reflected by the age‐related MS severity score (R = 0.46, *p* < 0.001). Presence of BRLs associated with a higher rate of leuko‐cortical but not subpial cortical lesions. Mixed lesions adjacent to ventricles and/or containing perivascular lymphocyte aggregations displayed broader rims, suggesting contributions of soluble factors. Distributions of perivascular CD79α‐positive B cells and CD3‐positive T cells are being investigated.

**Conclusion:** We consolidate BRLs as a pathological trait of donors with a more severe course of MS. B‐ and T‐cell populations and soluble factors from perivascular and subarachnoid compartments may contribute to their formation in MS distinctly from subpial lesion accumulation. Additional stainings for lymphocyte subsets and assessment of soluble factors in cerebrospinal fluid may reveal underlying mechanisms.

**Disclosure:** JS received research support and/or speaker fee and/or consulting fee of Biogen, Merck, Novartis, Roche, and Sanofi‐Genzyme. IH and HH received research support from Biogen.

## OPR‐079

### Temporal dynamics of serum neurofilament light chain in MS: A retrospective study in a clinical routine setting

#### M. Martínez‐Serrat ^1^; C. Tafrali^1^; R. Demjaha^1^; T. Kaiser^1^; E. Hofer^2^; S. Ropele^3^; B. Heschl^3^; S. Wurth^3^; A. Damulina^1^; D. Pinter^4^; D. Leppert^5^; P. Benkert^5^; J. Kuhle^5^

##### ^1^Neurology Biomarker Research Unit/Department of Neurology, Medical University of Graz, Graz, Austria; ^2^Institute for Medical Informatics, Statistics and Documentation, Medical University of Graz, Austria; ^3^Department of Neurology, Medical University of Graz, Graz, Austria; ^4^Research Unit for Neuronal Plasticity and Repair/Department of Neurology, Medical University of Graz, Graz, Austria; ^5^Department of Neurology & 5Multiple Sclerosis Centre and Research Center for Clinical Neuroimmunology and Neuroscience (RC2NB), Departments of Biomedicine and Clinical Research, University Hospital and University of Basel, Basel, Switzerland

**Background and Aims:** Serum neurofilament light chain(sNfL), a biomarker for neuroaxonal injury, is associated with MS disease activity. Elevated sNfL levels were linked to evidence of disease activity (EDA), compared to clinically/radiologically stable patients. However, the temporal dynamics of sNfL related to relapses and its routine clinical implications remain poorly understood. This study aims to evaluate the temporal changes in sNfL related to MS relapsing activity and their utility in clinical assessments.

**Methods:** Retrospective longitudinal data from 162MS patients (mean age = 32.5±7.8 years, median [IQR] disease duration 2.1[1‐7.1], 64.2% female) were analyzed, with a median of 7[IQR 6–9] serum samples per patient collected over a median follow‐up time of 10.4[IQR 7.8‐13.8] years. sNfL levels were quantified using Simoa HD‐X analyzer, results were adjusted for age and BMI using Z‐scores. Radiological activity was assessed through Gadolinium‐enhanced lesions using 3T‐MRI scans. EDA was defined as occurrence of clinical relapses, confirmed disability worsening (using EDSS scores) or radiological activity, within six months of sampling.

**Results:** sNfL Z‐scores were significantly elevated in patients with future EDA within one year of sampling, but only in samples taken during remission (*p* < 0.001). Additionally, sNfL levels didn't predict EDA beyond this one‐year window. The temporal analysis around clinical relapses showed increased sNfL Z‐scores at relapse onset (*p* < 0.001), with persisting levels up to 9 months post‐relapse.

**Conclusion:** These findings highlight the importance of monitoring sNfL as a dynamic biomarker for disease activity. Accurate knowledge of sNfL temporal dynamics is essential for correct interpretation in clinical practice and identifying patients at risk of disease activity. This approach could enhance clinical decision‐making and improve routine MS‐care.

**Disclosure:** C.T: travel funds, speaker honoraria from Merk R.D: travel funds from Janssen, Novartis, Sanofi A.D: was in sponsored meetings, received speaker honoraria or travel funds from Sanofi‐Aventis, Novartis, Janssen D.P: part of the advisory board “Cognition and MS” for Novartis; received speaker honoraria from Biogen, Novartis, MedAhead, Bristol‐Myers Squibb D.L: Chief Medical Officer of GeNeuro until end of 2023 J.K: received speaker fees, research support, travel support, and/or was on advisory boards by Swiss MS Society, Swiss National Research Foundation (320030_212534/1),University of Basel, Progressive MS Alliance, Alnylam, Bayer, Biogen, Bristol Myers Squibb, Celgene, Immunic, Merck, Neurogenesis, Novartis, Octave Bioscience, Quanterix, Roche, Sanofi, Stata DX C.E: travel funds and speaker honoraria from Biogen Idec, Bayer Schering Pharma, Merck Serono, Novartis, Genzyme, Teva Pharmaceutical Industries Ltd./Sanofi‐Aventis, Shire; received research support from Merck Serono, Biogen Idec, Teva Pharmaceutical Industries Ltd./Sanofi‐Aventis; is on scientific advisory boards for Bayer Schering Pharma, Biogen Idec, Merck Serono, Novartis, Genzyme, Roche, Teva Pharmaceutical Industries Ltd./Sanofi‐ Aventis M.K: travel funding and speaker honoraria from Bayer, Biogen, Novartis, Merck, Sanofi, Teva; is on scientific advisory boards for Biogen, Bristol‐Myers Squibb, Gilead, Merck, Novartis, Alexion, Amgen, Roche; received research grants from Biogen, Novartis and Teva Other: no disclosures.

## OPR‐080

### Effect of rituximab on non‐active secondary progressive multiple sclerosis – A retrospective multicenter analysis

#### N. Ehrhardt ^2^; K. Giglhuber^3^; A. Muzalyova^4^; J. Havla^5^; E. Oswald^5^; T. Kümpfel^5^; V. Rothhammer^2^; F. Nickel^2^; M. Naumann^1^; A. Berthele^3^; A. Bayas^1^

##### ^1^Department of Neurology and Clinical Neurophysiology, Medical Faculty, University of Augsburg, Augsburg, Germany; ^2^Department of Neurology, University of Erlangen‐Nuremberg, Erlangen, Germany; ^3^Department of Neurology, School of Medicine, Technical University of Munich, Klinikum rechts der Isar, Munich, Germany; ^4^Institute for Digital Medicine, University Augsburg, Neusäß, Germany; ^5^Institute of Clinical Neuroimmunology, LMU Hospital, Ludwig‐Maximilians University, Munich, Germany

**Background and Aims:** Non‐active secondary progressive multiple sclerosis (naSPMS) is characterized by a steady increase in disability without relapses or MRI activity. Currently, there is no approved immunotherapy available for, and a paucity of studies specifically addressing naSPMS. Rituximab (RTX) has occasionally been used off‐label for the treatment of naSPMS. The aim of this study was to investigate the efficacy and safety of RTX on disease progression in patients with naSPMS.

**Methods:** We conducted a retrospective multicenter study to identify patients with naSPMS lasting for at least six months, treated between February 2008 and October 2024. Baseline characteristics, disability progression before and during RTX treatment, magnetic resonance imaging (MRI) activity and safety aspects were analyzed.

**Results:** 46 patients with naSPMS and a mean age of 49.46 years (9.80, standard deviation, SD) at treatment start were included. Patients were predominantly male (59%), time since naSPMS onset to treatment was 4.72 (mean, 5.11, SD) years. EDSS at RTX treatment start was 5.45 (mean, 1.26, SD; median 6 (IQR 4‐6.5)). 12 months after treatment start, EDSS remained stable (*n* = 39), after 18 months EDSS had increased to 6 (IQR 4.38‐6.5, *p* = 0.046; *n* = 34), and to 6 (median, IQR 4,5‐6,5, *p* = 0,029; *n* = 24) after 36 months, compared to baseline. After treatment start, three patients had relapses and six MRI activity. Data on walking ability, MRI, B‐cells, and safety will be presented at the meeting.

**Conclusion:** We observed a mild, however, significant increase in disability despite RTX treatment in naSPMS. Treatment was generally well tolerated and safe.

**Disclosure:** NE, AM, EO, MN, FTN: none KG: travel grants from Nexstim, UCB, Viatris. JH: grant for OCT research from the Friedrich‐Baur‐Stiftung, Horizon, Sanofi and Merck, personal fees and nonfinancial support from Alexion, Amgen, Bayer, Biogen, BMS, Merck, Novartis and Roche, all outside the submitted work. TK: speaker honoraria and/or personal fees from Novartis Pharma, Roche Pharma, Alexion/Astra Zeneca, Horizon Therapeutics/Amgen, Merck, Chugai Pharma and Biogen. Compensation for serving as a member of a steering committee from Roche (institutional). Her institution has received compensation for clinical trials from Novartis Pharma, Roche Pharma and Sanofi Genzyme, all outside the present work. VR: speaker honoraria and compensation for serving in advisory boards for Alexion, Biogen, Novartis, Pfizer, Roche, Sanofi‐Aventis, and Teva. ABe: consulting and/or speaker fees from Alexion, Argenx, Biogen, Horizon/Amgen, Merck, Neuraxpharm, Novartis, Roche and Sandoz/Hexal, and his institution has received compensation for clinical trials from Alexion, Biogen, Merck, Novartis, Roche, and Sanofi Genzyme; all outside the present work. ABa: personal compensation from Merck, Biogen, Novartis, TEVA, Roche, Sanofi/Genzyme, Celgene/Bristol Myers Squibb, Janssen, Sandoz/HEXAL, Alexion, Horizon, Argenx, research support by Novartis, and grants for congress travel and participation from Biogen, TEVA, Novartis, Sanofi/Genzyme, Merck Serono, Celgene, Janssen and Roche. None related to the present work.

## OPR‐081

### sNfL and GFAP levels are associated with retinal layer thinning in multiple sclerosis

#### N. Krajnc^1^; M. Ponleitner^1^; F. Föttinger^1^; F. Leutmezer^1^; S. Macher^1^; T. Monschein^1^; P. Rommer^1^; B. Kornek^1^; C. Schmied^1^; K. Zebenholzer^1^; G. Zulehner^1^; T. Zrzavy^1^; T. Berger^1^; B. Pemp^2^; G. Bsteh ^1^

##### ^1^Department of Neurology, Medical University of Vienna, Vienna, Austria; ^2^Department of Ophthalmology, Medical University of Vienna, Vienna, Austria

**Background and Aims:** Serum neurofilament light chain (sNfL) and glial fibrillary acidic protein (GFAP) are emerging biomarkers of axonal damage and astrocytic activation, both likely paramount in MS associated neurodegeneration. However, their value in predicting retinal layer thinning remains underexplored.

**Methods:** This prospective observational study included patients with relapsing MS newly initiated on a disease‐modifying therapy (DMT). OCT scans were conducted 3–6 months after DMT initiation (rebaseline) and at 12‐month intervals measuring peripapillary retinal nerve fiber layer (pRNFL) and ganglion cell‐inner plexiform layer (GCIPL) thickness. Serum sNfL and GFAP levels were measured using single‐molecule array (Simoa) technology, with z‐scores adjusted for age, BMI and – for GFAP – sex.

**Results:** A total of 116 patients (mean age 34.5 years (SD 8.6)), 73.3% female, median disease duration 2.8 years (IQR 0.3–7.1), median EDSS 1.5 (range 0–5.5)) were included. Both pRNFL (b = –0.34; 95% CI –0.67, –0.02; *p* = 0.04) and GCIPL thicknesses (b = –0.39; 95% CI –0.65, –0.12; *p* = 0.004) were associated with GFAP – but not sNfL – z‐scores at baseline. GFAP z‐scores at M6 showed the strongest association with aLpRNFL (b = –0.24; 95% CI –0.27, –0.21, *p* < 0.001) and aLGCIPL (b = –0.15; 95% CI –0.18, –0.12, *p* < 0.001). Moreover, patients with low sNfL but high GFAP levels at M6 showed the most pronounced inner retinal layer thinning (aLpRNFL: –0.8%/year (1.1), aLGCIPL: –0.8%/year (0.9); both *p* < 0.001).

**Conclusion:** High GFAP levels – more than sNfL levels – are associated with inner retinal layer thinning in RMS, underscoring their value as biomarkers of disease progression.

**Disclosure:** All authors declare no conflict of interest relevant to this study.

## OPR‐082

### Multimodal analysis of brain networks contributes to characterize clinical profiles in multiple sclerosis

#### P. Preziosa ^1^; P. Valsasina^2^; N. Tedone^3^; M. Schoonheim^4^; A. Gallo^5^; P. Pantano^6^; C. Enzinger^7^; S. Llufriu^8^; M. Calabrese^9^; G. Pontillo^10^; E. Høgestøl^11^; S. Groppa^12^; N. De Stefano^13^; S. Collorone^14^; M. Rocca^1^; M. Filippi^15^

##### ^1^Neuroimaging Research Unit, Division of Neuroscience, and Neurology Unit, IRCCS San Raffaele Scientific Institute, and Vita‐Salute San Raffaele University, Milan, Italy; ^2^Neuroimaging Research Unit, Division of Neuroscience, IRCCS San Raffaele Scientific Institute, Milan, Italy; ^3^Neuroimaging Research Unit, Division of Neuroscience, IRCCS San Raffaele Scientific Institute, and Vita‐Salute San Raffaele University, Milan, Italy; ^4^Multiple Sclerosis Center Amsterdam, Anatomy and Neurosciences, Vrije Universiteit Amsterdam, Amsterdam Neuroscience, Amsterdam University Medical College VUMC, Amsterdam, the Netherlands; ^5^Department of Advanced Medical and Surgical Sciences and 3T MRI center, University of Campania “Luigi Vanvitelli”, Naples, Italy; ^6^Department of Human Neurosciences, Sapienza University of Rome, Rome, Italy, and IRCCS NEUROMED, Pozzilli, Italy; ^7^Department of Neurology, Medical University of Graz, Graz, Austria; ^8^Neuroimmunology and Multiple Sclerosis Unit and Laboratory of Advanced Imaging in Neuroimmunological Diseases (ImaginEM), Hospital Clinic and Institut d'Investigacions Biomèdiques August Pi i Sunyer (IDIBAPS), University of Barcelona, Barcelona, Spain; ^9^Department of Neurosciences and Biomedicine and Movement, The Multiple Sclerosis Center of University Hospital of Verona, Verona, Italy; ^10^Departments of Advanced Biomedical Sciences and Electrical Engineering and Information Technology, University of Naples “Federico II,” Italy; ^11^Department of Neurology, Division of Clinical Neuroscience, Oslo University Hospital, Oslo, Norway; ^12^Department of Neurology, Neurostimulation and Neuroimaging, University Medical Center of the Johannes Gutenberg University, Mainz, Germany; ^13^Department of Medicine, Surgery and Neuroscience, University of Siena, Siena, Italy; ^14^Queen Square MS Centre, Department of Neuroinflammation, UCL Queen Square Institute of Neurology, Faculty of Brain Science, University College of London, London, UK; ^15^Neurology Unit, Neurorehabilitation Unit, Neurophysiology Service, and Neuroimaging Research Unit, Division of Neuroscience, IRCCS San Raffaele Scientific Institute, and Vita‐Salute San Raffaele University, Milan, Italy

**Background and Aims:** Multiple sclerosis (MS) presents variable clinical manifestations among individuals. Here, we performed a multimodal analysis of grey matter (GM) structural and functional networks in a multi‐center cohort (12 European sites), to evaluate the contribution of structural/functional MRI GM damage in depicting MS clinical features.

**Methods:** 3D T1‐weighted, resting state (RS) functional MRI and clinical evaluations were obtained from 1754 MS patients (66 clinically isolated syndromes [CIS], 1342 relapsing‐remitting [RR] and 346 progressive [P] MS) and 597 healthy controls (HC). Parallel independent component analysis (P‐ICA) on GM volume and degree centrality maps produced structural/functional network components and corresponding Z‐scores.

**Results:** P‐ICA identified six structural GM networks with significant atrophy in MS patients vs HC (*p* range < 0.001‐0.02). CIS patients showed atrophy in the default‐mode network (DMN) (*p* < 0.001), RRMS had additional atrophy in occipital, deep GM, and fronto‐parietal networks (all *p* < 0.001), while PMS exhibited further atrophy in DMN and occipital networks (both *p* = 0.002). P‐ICA also identified three sensorimotor networks showing increased functional connectivity (FC) in MS patients vs HC (*p* = range 0.007/ < 0.001), while the DMN, fronto‐parietal and salience networks showed decreased FC (all *p* < 0.001). CIS patients presented limited FC abnormalities, while more pronounced decrease FC in PMS vs RRMS was found in the DMN (*p* = 0.002) and fronto‐parietal (*p* = 0.01) networks. In MS, most of networks showed decreased structural‐functional association (interaction *p* range = 0.04 to <0.001), correlating with higher disability and T2 lesion volume.

**Conclusion:** Multimodal analysis of structural/functional brain networks helped to unravel complex changes of human brain organization associated with MS disease.

**Disclosure:** Nothing to disclose.

## Epilepsy

## OPR‐083

### The Epilepsy Deaths Register: Friend, family and care‐giver reports of SUDEP in adults and older adolescents

#### A. Grundmann ^1^; J. Brolly^1^; D. Craig^1^; K. Osland^2^; J. Hanna^2^; E. Hughes^3^; M. Kerr^4^; B. Donovan^2^; R. Thomas^1^

##### ^1^Royal Victoria Infirmary, Newcastle‐upon‐Tyne, UK; ^2^SUDEP Action, Wantage, Oxfordshire, UK; ^3^Evelina London Children's Hospital, London, UK; ^4^Psychological Medicine and Clinical Neurosciences, Cardiff University, UK

**Background and Aims:** Sudden unexpected death in epilepsy (SUDEP) is the most common cause of epilepsy related death. Understanding SUDEP characteristics through third‐party accounts is vital, yet these valuable narratives remain underutilised. This study is the first comprehensive analysis of adult SUDEP characteristics from accounts in the epilepsy deaths register

**Methods:** We collected the characteristics of the deceased and narratives surrounding death via the SUDEP action UK epilepsy deaths register (EDR) third‐party reports. We included those aged ≥15 years with a post‐mortem, death‐certificate or narrative in keeping with SUDEP. Duplicate submissions, non‐SUDEP causes, and cases without certified causes of death were excluded. We collected the demographics, details of follow‐up, events leading to death, and attitudes towards condition and treatment in life.

**Results:** From 1,056 EDR registrations (2013‐2024), 409 met SUDEP criteria. Cases were predominantly male (59.4%), aged 19‐49 years (76.3%), and living with family/friends (71.6%). Of determinable cases, 85.9% occurred at night and 80.8% during sleep, with 63.2% found prone. While 90.6% were prescribed anti‐epileptics and 82.3% had specialist follow‐up, SUDEP was not recorded as cause of death in 24.9% despite consistent narratives. Notably, 51.5% of reporters were unaware epilepsy could be fatal.

**Conclusion:** SUDEP is an underreported cause of death in patients with epilepsy. Third‐party reports are an effective tool to sample SUDEP deaths. The heterogeneity of SUDEP cases and the high proportion of respondents unaware of fatal epilepsy outcomes support universal SUDEP risk counselling and emphasise the value of third‐party reporting in deepening SUDEP understanding.

**Disclosure:** RHT has received honoraria from Angelini, Bial, Eisai, GW Pharma, Paladin, NeuraxPharm, Sanofi, Takeda, UCB Pharma, UNEEG, Zogenix, and unrestricted research funding from Angelini/Arvelle and UCB Pharma, independent of this project ‐ Jacob Brolly has received honoraria from UCB phrarmapharma ‐ Donald P Craig has received a consultancy fee from Eisai ‐ Karen Osland was project lead for the epilepsy deaths register for the UK charity SUDEP action until April 2020. ‐ Ben Donovan is the project lead for the epilepsy deaths register for the UK charity SUDEP Action ‐ Jane Hanna OBE was previously chief executive of the UK charity SUDEP action ‐ Elaine Hughes, participated in multi‐centre commercial trials of fenfluramine in treatment of epilepsy in Dravet syndrome and is a member of the GW Pharmaceuticals supported LGS Advisory Board. ‐ Mike P Kerr is Vice Chair of SUDEP Action, the charity that supports the Epilepsy Deaths Register.

## OPR‐084

### P2Y12 receptor dysregulation in MTLE‐HS: Insights from human brain tissue and an in vitro model

#### B. Guerra Leal ^1^; P. Beça^2^; C. Carvalho^2^; C. Santos^3^; R. Samões^4^; C. Teixeira^5^; P. Pinho e Costa^1^; M. Lobo^6^; P. Correia de Sá^6^; J. Chaves^4^

##### ^1^Unit for Multidisciplinary Research in Biomedicine (UMIB, ICBAS‐UPorto); Immunogenetics Laboratory, ICBAS‐UPorto; Laboratory for Integrative and Translational Research in Population Health (ITR), Porto, Portugal; ^2^Immunogenetics Laboratory, Department of Molecular Pathology and Immunology, Instituto de Ciências Biomédicas Abel Salazar Universidade do Porto (ICBAS‐UPorto), Porto, Portugal; ^3^Unit for Multidisciplinary Research in Biomedicine (UMIB,ICBAS‐UPorto); Immunogenetics Laboratory, Department of Molecular Pathology and Immunology, Instituto de Ciências Biomédicas Abel Salazar Universidade do Porto (ICBAS‐UPorto), Porto, Portugal; ^4^Unit for Multidisciplinary Research in Biomedicine (UMIB,ICBAS‐UPorto); Hospital de Santo António, Unidade Local de Saúde de Santo António, Porto, Portugal; ^5^Hospital de Santo António, Unidade Local de Saúde de Santo António, Porto, Portugal; ^6^Laboratório de Farmacologia e Neurobiologia—Center for Drug Discovery and Innovative Medicines (MedInUP), ICBAS‐UP, Porto, Portugal

**Background and Aims:** Chronic microglial activation is a hallmark of mesial temporal lobe epilepsy with hippocampal sclerosis (MTLE‐HS). The ADP‐sensitive P2Y12 receptor plays a key role in initiating microglial activation. Given the link between prolonged microglial activation and increased synaptic activity, we aimed to study the role of P2Y12R in MTLE‐HS by (i) analyzing P2Y12R mRNA expression in human brain tissue and (ii) developing an in vitro model of microglia‐like cells derived from peripheral blood monocytes (MMG).

**Methods:** Hippocampal and temporal neocortex samples were obtained from 19 MTLE‐HS patients (8M, 43.5 ± 10.0 years) and 10 cadaveric controls (8M, 67.0 ± 10.0 years). MMG cells were differentiated from the blood monocytes of 15 MTLE‐HS patients and 5 healthy donors. P2Y12R mRNA expression was quantified using Real‐Time PCR.

**Results:** P2Y12R mRNA levels were significantly higher in the hippocampus (1.78‐fold, *p* = 0.004) and temporal neocortex (2.89‐fold, *p* < 0.001) of MTLE‐HS patients compared to controls, with no correlation to age. Homeostatic MMG cells presented a ramified morphology and expressed P2Y12R, Upon LPS stimulation, MMG cells became amoeboid and showed marked P2Y12R downregulation.

**Conclusion:** P2Y12R expression changes dynamically during epilepsy progression. It initially facilitates microglial activation but is later downregulated to promote migration. Its upregulation in the temporal neocortex suggests a role in MTLE‐HS progression. Further research is needed to clarify the interplay between P2Y12R and other purinergic receptors in MTLE‐HS. Moreover, our work confirms MMG cells as a valuable in vitro model for studying microglial function in epilepsy and neuroinflammatory conditions.

**Disclosure:** Work funded by an FCT grant 2022.10372.PTDC.

## OPR‐085

### Exploring apolipoprotein ε4 in progressive myoclonic epilepsy type 1

#### J. Gunnar ^1^; H. Eronen^1^; T. Joensuu^2^; Y. Liu^1^; M. Äikiä^3^; J. Hyppönen^3^; K. Silvennoinen^3^; E. Mervaala^4^; J. Hakumäki^4^; A. Lehesjoki^2^; R. Kälviäinen^5^

##### ^1^Institute of Clinical Medicine, University of Eastern Finland, Kuopio, Finland; ^2^Folkhälsan Research Center and Medicum, University of Helsinki, Helsinki, Finland; ^3^Kuopio Epilepsy Center, Kuopio University Hospital, Member of ERN EpiCARE, Kuopio, Finland; ^4^Institute of Clinical Medicine, University of Eastern Finland and Imaging Center, Kuopio University Hospital, Kuopio, Finland; ^5^Institute of Clinical Medicine, University of Eastern Finland and Kuopio Epilepsy Center, Kuopio University Hospital, Member of ERN EpiCARE, Kuopio, Finland

**Background and Aims:** Progressive myoclonic epilepsy type 1 (EPM1) is a neurodegenerative disease caused by biallelic alterations in the cystatin B (CSTB) gene. Despite a progressive course, phenotype severity varies among patients, even within families. We studied the possible role of APOE ε4 in modifying phenotypic diversity in EPM1.

**Methods:** As part of our large EPM1 study, APOE genotypes were determined for 65 genetically verified EPM1 patients homozygous for the CSTB expansion mutation. The Unified Myoclonus Rating Scale (UMRS), Quality of Life in Epilepsy Inventory‐31 questionnaire (QOLIE‐31), clinical data, and quantitative neuroimaging data were compared between APOE ε4 carriers and non‐carriers to assess potential correlations with EPM1 severity.

**Results:** The cohort included 20 ε4 carriers (16 ε3/ε4 and 4 ε4/ε4) and 45 non‐carriers (36 ε3/ε3, 8 ε2/ε3, and 1 ε2/ε2). No significant differences were found in UMRS or disease duration. Carriers had better QOLIE‐31 scores in emotional well‐being (*p* = 0.047), energy/fatigue (*p* = 0.048), and medical effects (*p* = 0.024). In volumetric analysis, carriers exhibited larger bilateral hippocampus and amygdala volumes but reduced cortical thickness in the left lingual gyrus, right lateral occipital gyrus, and right posterior cingulate (*p* < 0.05). Carriers exhibited widespread white matter degeneration in diffusion tensor imaging, characterized by reduced fractional anisotropy and increased mean diffusivity.

**Conclusion:** Despite greater white matter degeneration, APOE ε4 carriers exhibited preserved deep brain volumes and better self‐reported well‐being. This study highlights the complex interplay between genetic factors and neurodegenerative processes. Our future research aims to provide more natural history data of EPM1 and correlate long‐term phenotypic data with additional geno‐phenotypic analyses.

**Disclosure:** Nothing to disclose.

## OPR‐086

### Morphological and functional evaluation of epileptogenesis in tuberous sclerosis using brain organoids

#### L. Barea‐Moya ^1^; S. Nagumo Wong^2^; O. Eichmüller^2^; M. Zabolocki^2^; J. García Verdugo^3^; S. González‐Granero^3^; S. Gil‐Perotin^1^; J. Knoblich^2^

##### ^1^Group in Immunotherapy and Biomodels for Autoimmunity, La Fe Health Research Institute, Valencia, Spain; ^2^IMBA–Institute of Molecular Biotechnology of the Austrian Academy of Sciences, Vienna Biocenter (VBC); Vienna, Austria; ^3^Laboratory of Comparative Neurobiology, Cavanilles Institute of Biodiversity and Evolutionary Biology, University of Valencia and CIBERNED‐ISCIII; Valencia, Spain

**Background and Aims:** Tuberous sclerosis complex (TSC) is a neurodevelopmental disorder caused by mutations in TSC1 or TSC2, associated with widespread network dysfunction and drug‐resistant epilepsy. The mechanisms underlying epileptogenesis remain elusive due to the lack of human‐specific biomodels. This study integrates morphological and functional evaluations using patient‐derived brain organoids to uncover key processes in TSC‐related epileptogenesis.

**Methods:** Human induced pluripotent stem cells (hiPSCs) from TSC patients and isogenic controls were differentiated into brain organoids. Morphological features were examined using immunohistochemistry and electron microscopy to assess cellular organization, dendritic morphology, and ultrastructural features. Functional network dynamics were evaluated using extracellular silicon probe recordings. Organoid findings were correlated with intraoperative electrocorticography (ioECoG) data and post resection samples from TSC patients.

**Results:** Histological analyses revealed altered interneuron distribution, including expansion of caudal ganglionic eminence (CGE)‐derived populations in TSC organoids. Structural abnormalities, such as MAP2‐positive dendritic swellings and mitochondrial disruptions, highlighted excitotoxic damage. Ultrastructural analyses showed pathological synaptic densities and microtubule disarray (Figure 1). Functionally, TSC organoids exhibited hyperexcitability, with increased frequency of pathological high‐frequency oscillations (HFOs) and dysregulated network synchronization. These abnormalities were consistent with epileptogenic signatures observed in ioECoG recordings from TSC patients (Figures 2 and 3).

**Figure:**
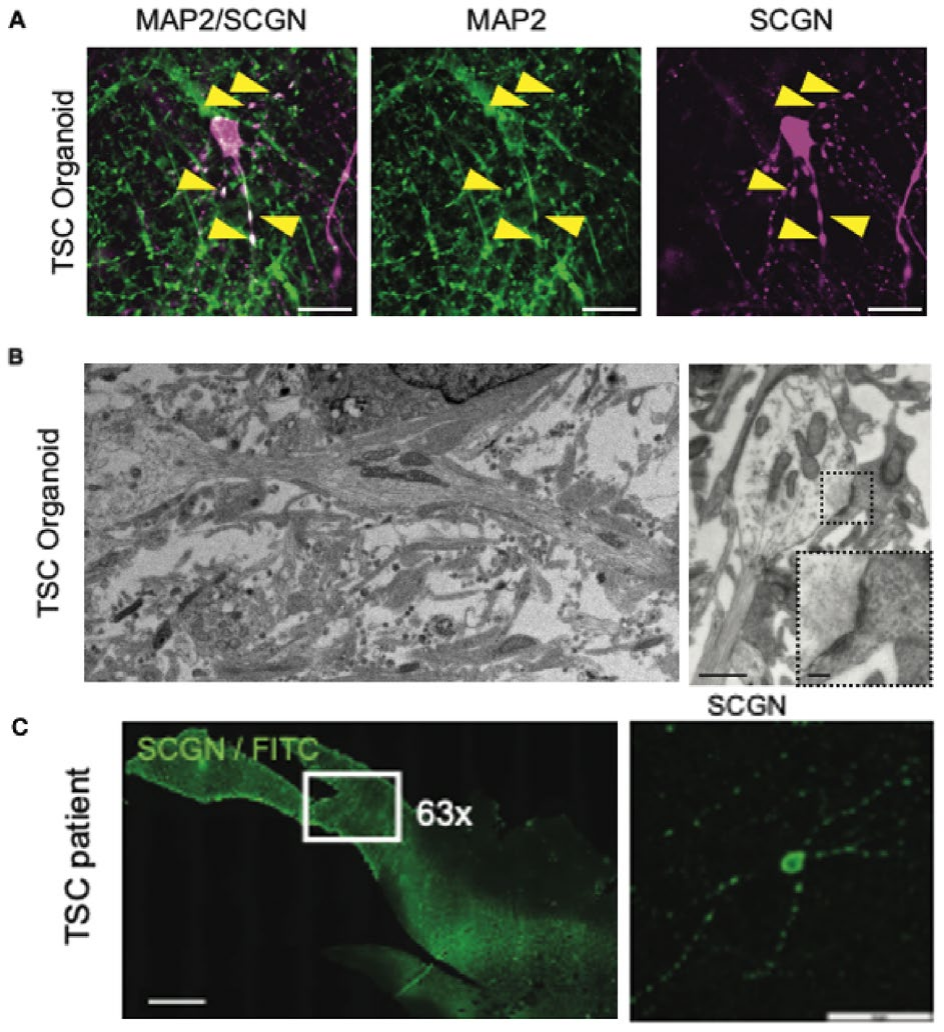
**FIGURE 1** A: SCGN+ interneurons in TSC organoids show dendritic beading (SCGN+/MAP2+). B: EM reveals enlarged postsynaptic beads with damaged microtubules and mitochondria. C: TSC resected samples confirm pathological SCGN+ dendritic beading.

**Figure:**
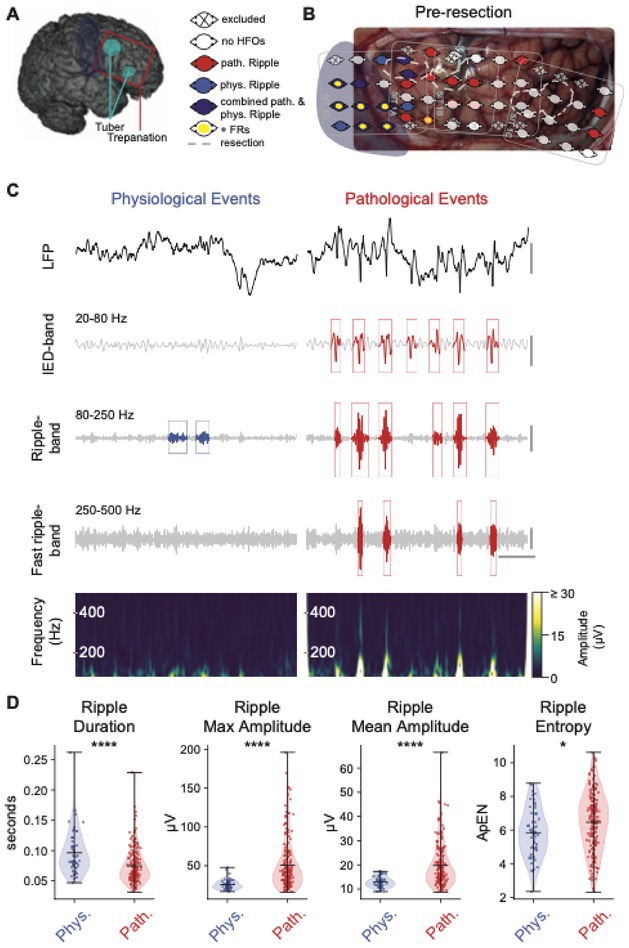
**FIGURE 2** A and B: Example of ioECoG recording. C: Characterization of physiological (blue) vs pathological (red) HFOs, which show IED‐ripple alignment. D: Ripple metrics reveal significant differences.

**Figure:**
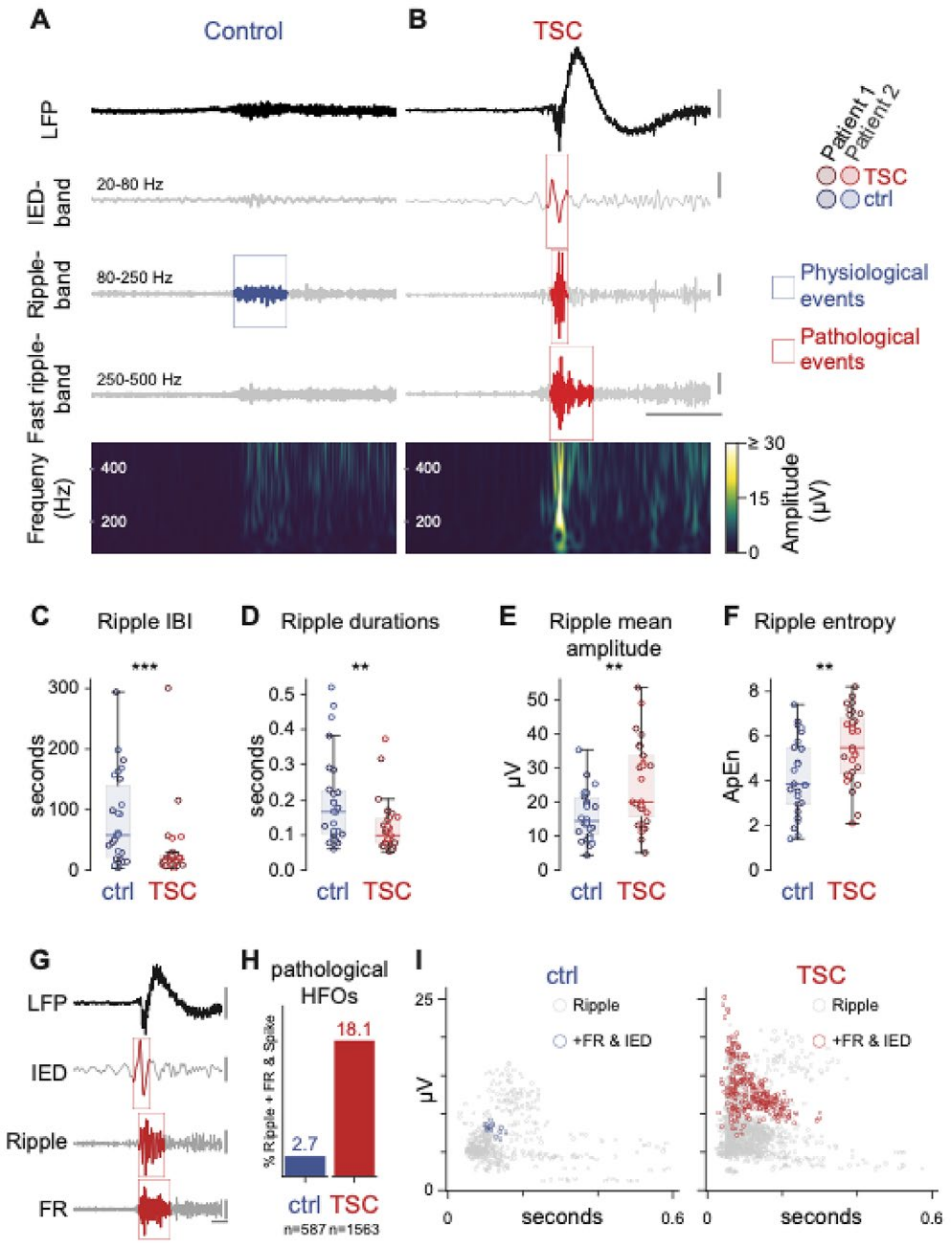
**FIGURE 3** A‐B: LFP and band‐filtered signals show spikes, ripples, and fast ripples (control: blue; TSC: red). C‐F: TSC organoids show altered ripple inter‐burst interval, duration, amplitude, and entropy. G‐I: Epileptogenic properties of TSC organoid.

**Conclusion:** This study demonstrates that brain organoids can faithfully model morphological and functional changes underlying epileptogenesis in TSC. By integrating structural and electrophysiological evaluations, this approach identifies critical roles for CGE‐derived interneurons, dendritic alterations, and pathological network synchronization. These findings provide a platform for advancing mechanistic understanding and exploring therapeutic strategies for TSC and epilepsy.

**Disclosure:** Nothing to disclose.

## OPR‐087

### Exploring high‐frequency oscillations (HFOs) in human organotypic brain slice cultures: An ex‐vivo approach

#### S. Wolking ^1^; J. Heckelmann^1^; A. Höllig^2^; H. Hamou^2^; J. Ort^2^; R. Pjontek^2^; K. van Loo^1^; Y. Weber^1^; H. Koch^1^

##### ^1^Department of Neurology, Section of Epileptology, University Hospital Aachen, Aachen, Germany; ^2^Department of Neurosurgery, RWTH Aachen University, Aachen, Germany

**Background and Aims:** High‐frequency oscillations (HFOs) have been studied for over 25 years and emerged as a valuable biomarker in the presurgical assessment of epilepsy patients. The underlying pathophysiologic mechanisms remain incompletely understood. Currently, HFOs in humans are predominantly investigated in‐vivo during stereo‐EEG (sEEG), limiting coverage and experimental interventions. Human organotypic brain slice cultures (HOBSCs) enable long‐term investigations of brain physiology, including the recording of neuronal activity and network properties using multi‐electrode arrays (MEA). Here, we present preliminary data demonstrating HFO detection in HBOSCs, offering an ex‐vivo model to investigate HFO pathophysiology.

**Methods:** We obtain brain tissue from epilepsy or tumor surgery to prepare brain slices. Human cerebrospinal fluid (hCSF) is used for slice culturing, granting viability for up to three weeks. We use 256‐channel MEAs, covering 3.2x3.2mm2, to record whole‐slice electrophysiology, enabling the measurement of local field potentials, action potentials, and propagation dynamics. Computational post‐processing includes frequency filtering and semi‐automated HFO detection. Slices are exposed to varying temperatures and excitatory or inhibitory agents to assess their impact on HFOs.

**Results:** We demonstrate a consistent detection of HFOs in HOBSCs from hippocampal resections of temporal lobe epilepsy patients. HFOs spatially correspond to anatomical regions of increased spiking. HFO frequency positively correlates with temperature. Treatment with norepinephrine and GABAA‐antagonists increases HFO frequency, while AMPA‐antagonists reduce HFO occurrence.

**Figure:**
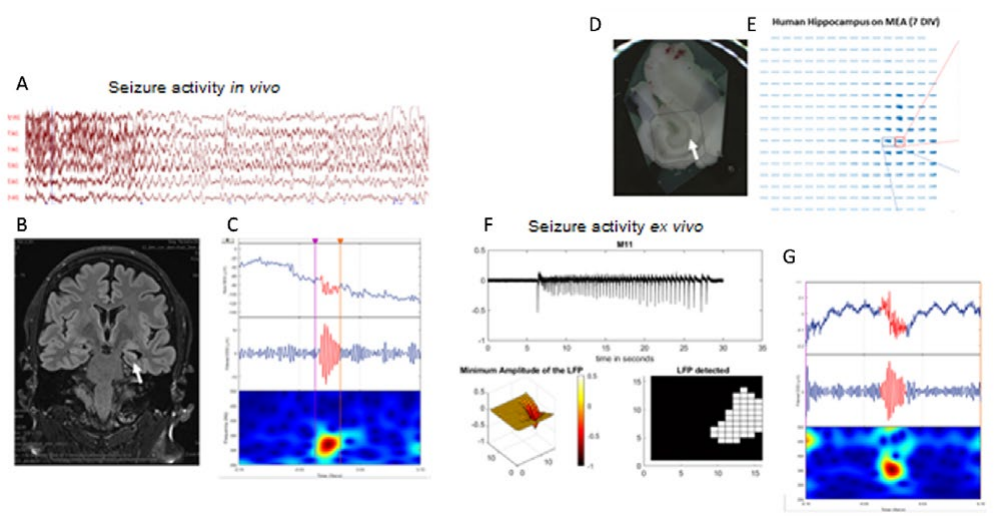
**FIGURE 1** A: Surface EEG of patient. B: MRI with hippocampal sclerosis. C: Filtered sEEG show hippocampal HFO. D: Resected hippocampus with superimposed position of MEA grid. E: MEA recording with spontaneous seizure activity. F: Extracted seizure activity.

**Conclusion:** We introduce a novel approach to study HFOs in human brain tissue, providing a platform for experimental interventions. This opens new perspectives to investigate the pathophysiologic basis of HFOs and their role across different brain lesions.

**Disclosure:** None.

## MS and Related disorders 3

## OPR‐088

### The Cerebellar Cognitive Affective Syndrome Scale in early Multiple Sclerosis: A diffusion and functional‐MRI study

#### A. Miscioscia ^1^; E. Silvestri^2^; G. Scialpi^3^; A. Berardi^3^; M. Colpo^2^; G. Vallini^2^; M. Anglani^4^; F. Rinaldi^3^; P. Perini^3^; M. Puthenparampil^1^; A. Bertoldo^2^; P. Gallo^1^

##### ^1^Department of Neuroscience, University of Padua, Italy; ^2^Department of Information Engineering, University of Padua, Italy; ^3^Multiple Sclerosis Centre of the Veneto Region (CeSMuV), Padua University Hospital, Italy; ^4^Neuroradiology Unit, Padua University Hospital, Padua, Italy

**Background and Aims:** Damage to cerebellar posterior lobes can result in Cerebellar Cognitive Affective Syndrome (CCAS). The validity of the CCAS Scale (CCAS‐S), a reliable tool to diagnose CCAS, remains unexplored in MS. In this study, we aimed to determine the ability of CCAS‐S to detect CCAS in MS at clinical onset. Using conventional, diffusion (dMRI), and resting state‐functional MRI (rs‐fMRI), we also assessed the clinical and MRI characteristics of MS patients with CCAS (CCAS+). Lastly, we identified the MRI predictor most strongly associated with CCAS in MS.

**Methods:** Seventy early MS patients underwent CCAS‐S and standard cognitive assessments. Twenty healthy controls and 56 patients also underwent MRI to obtain lesion and volumetric parameters, dMRI metrics, and cerebellum‐brain functional connectivity (FC). Regressions were used to study associations between MRI metrics and CCAS‐S scores.

**Results:** CCAS‐S identified 9 (13%) MS patients with CCAS and normal scores on standard cognitive assessments. CCAS+ patients showed lower fractional anisotropy in cerebellar normal‐appearing white matter (*p* = 0.020), and increased cerebro‐cerebellar connectivity on rs‐fMRI (*p* < 0.05). Finally, we found that the cortical lesion volume was the strongest predictor for low CCAS‐S performance in MS (R^2^ = 0.446, β = ‐0.009, *p* = 0.004).

**Figure:**
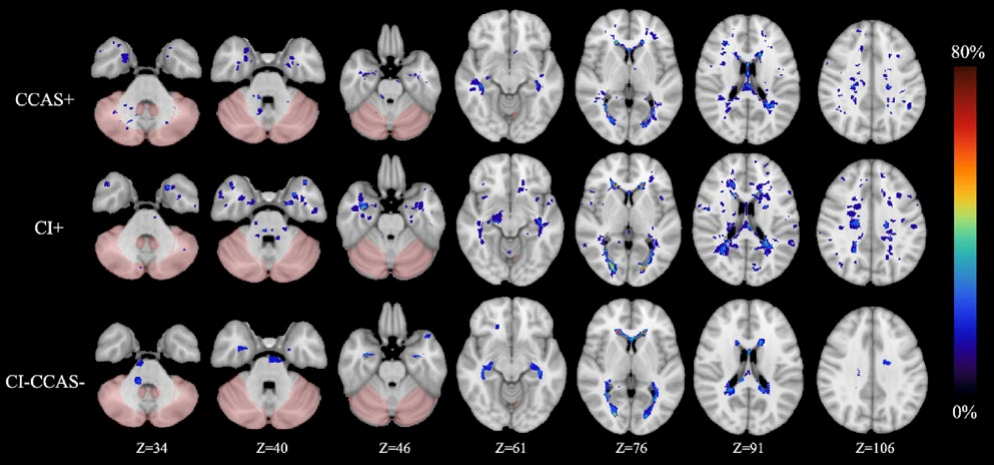
**FIGURE 1** Probabilistic lesion map over a selection of axial slices, showing the lesion load percentage in MS patients with CCAS+ (*n* = 9), MS patients with impaired standard cognitive test (CI+, *n* = 10), and cognitively normal MS patients (CI‐ CCAS‐, *n* = 37).

**Figure:**
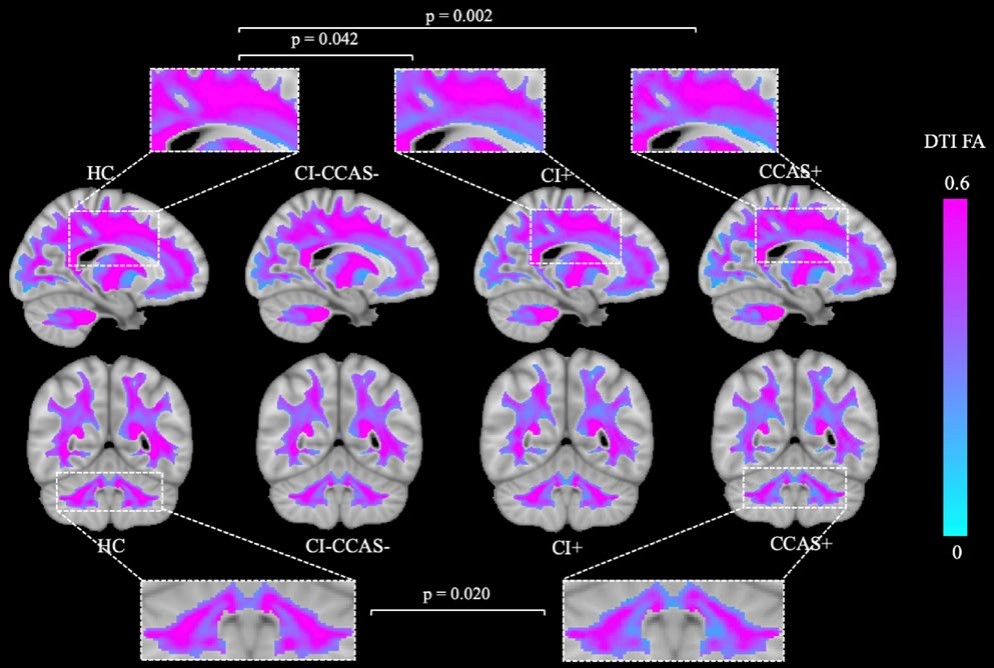
**FIGURE 2** DTI maps showing the mean NAWM FA for each group. Both CI+ and CCAS+ patients exhibited significantly lower FA in the brain NAWM compared to HC. Only CCAS+ patients demonstrated lower FA in the cerebellar NAWM compared to HC.

**Figure:**
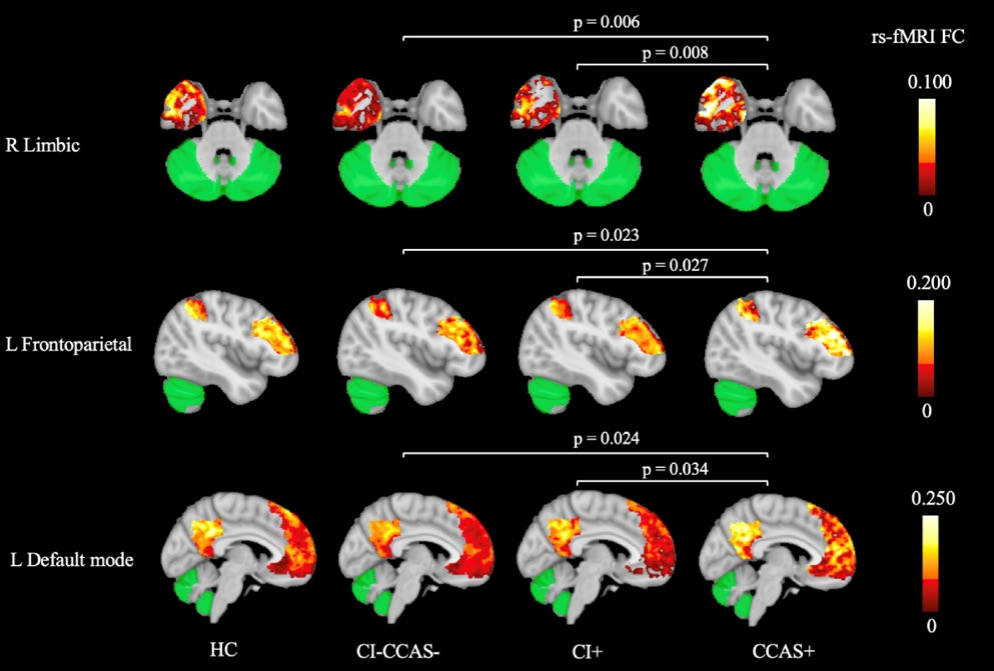
**FIGURE 3** Seed‐based rs‐fMRI connectivity analysis showing differences in functional connectivity across groups. CCAS+ patients showed increased cerebellum connectivity in the right limbic, left frontoparietal, and left DMN compared to CI‐ CCAS‐ and CI+ patients.

**Conclusion:** CCAS‐S is a valid tool to complement standard cognitive assessments, enhancing sensitivity in detecting cognitive impairment in MS at clinical onset. CCAS+ patients are characterized by severe microstructural cerebellar damage and increased brain‐cerebellar connectivity. Possibly due to a diffuse cortical pathology shared between the brain and cerebellum, the presence of brain cortical lesions is a strong predictor of CCAS in MS.

**Disclosure:** Nothing to disclose.

## OPR‐089

### Deep sulcal inflammation drives local cortical atrophy: A self‐sustained loop of neurodegeneration in multiple sclerosis

#### A. Lazzarotto

##### Sorbonne Université, Paris Brain Institute, Paris, France

**Background and Aims:** Within the cortex of individuals with MS, meningeal inflammation and activated innate immune cells are frequently observed, but their regional distribution along sulci and relationship with CSF dynamics and cortical atrophy remain unclear. This study examined sulcal inflammation, CSF stagnation, and cortical atrophy over two years in two independent MS cohorts.

**Methods:** Participants (36 and 40 MS patients, plus healthy controls) underwent baseline [18F]‐DPA‐714 PET to assess innate immune cell inflammation and annual MRI for 24 months. The validation cohort also underwent low b‐value diffusion tensor imaging to estimate CSF stagnation. Comparing [18F]‐DPA‐714 DVR maps between MS and controls, we identified inflamed cortical sulci and analyzed immune cell distribution, CSF dynamics, and sulcal enlargement over time.

**Results:** In MS patients, 11.7% of sulci were inflamed, with deeper sulcal regions showing greater immune activation (*p* = 0.007). Increased sulcal inflammation predicted local atrophy over two years (*p* < 0.0001), with each one‐point DVR increase raising atrophy likelihood by 30% (OR = 3.49, *p* < 0.0001). The validation cohort confirmed these findings and further linked sulcal inflammation to increased CSF stagnation (β = 0.10; *p* < 0.0001).

**Conclusion:** Cortical inflammation predominantly affects deep sulci, driving atrophy in MS. CSF stagnation may exacerbate this by prolonging exposure to pro‐inflammatory components, perpetuating the cycle of chronic inflammation and neurodegeneration.

**Disclosure:** Nothing to disclose.

## OPR‐090

### Dysarthria assessment in Multiple Sclerosis patients

#### D. Ranucci ^1^; A. Carotenuto^1^; L. Marra^1^; L. Migliaccio^1^; F. Caracciolo^1^; G. Corsini^1^; A. Esposito^1^; V. Nicolella^1^; A. Castiello^1^; M. Petracca^2^; M. Moccia^3^; V. Brescia Morra^1^; R. Lanzillo^1^

##### ^1^Department of Neurosciences, Reproductive Sciences and Odontostomatology, University of Naples Federico II, Naples, Italy; ^2^Department of Human Neurosciences, Sapienza University of Rome, Rome, Italy; ^3^Department of Molecular Medicine and Medical Biotechnology, Federico II University of Naples, Naples, Italy

**Background and Aims:** Multiple Sclerosis (MS) is a disabling disorder affecting young adults. Speech impairment patterns in MS are poorly characterized. Dysarthria Analyzer (DA) is a software allowing a detailed speech analysis.

**Methods:** All patients underwent clinical assessments (EDSS and BICAMS) and speech evaluation using the DA. Each patient performed 4 tasks: phonation A, phonation I, reading, monologue and syllable repetition. DA automatically extracted 28 speech features, adjusted for age, gender and education toward internal controls (z‐scores). A principal component analysis (PCA) was conducted, retaining components with eigenvalues>1. Correlations were assessed through forward stepwise analysis, including age, sex, EDSS, Symbol Digit Modalities Test (SDMT), California Verbal Learning Test (CVLT), Brief Visuospatial Memory Test Revised (BVMTR) and smoking status as covariates.

**Results:** We enrolled 72 patients [50 females; mean age 48.1±10.9 years; median disease duration of 14(0–34) years and a median EDSS of 3(1.5–6.5)]. PCA identified an 8‐component model: Monopitch, Nasal Voice, Slow Sequential Motion Rates, Monoloudness, Prolonged Pauses, Tremor Voice, Speech Timing, Respiration Quality. Higher Monoloudness scores were associated with lower SDMT scores (corr.coeff = ‐0.04, *p* = 0.001). Prolonged Pauses and Shorter Speech Timing correlated with higher EDSS (corr.coeff = 0.25, *p* = 0.03 and corr.coeff = ‐0.3, *p* = 0.001).

**Conclusion:** Detailed speech analysis can reflect various aspects of MS disability. Possibly, cognition is crucial in speech modulation for conveying meaning in social interactions, while motor disability is more closely linked to muscle control involved in phonation and speech articulation in MS patients.

**Disclosure:** A.E. has received honoraria from Novartis. M.M. has received research grants from ECTRIMS‐MAGNIMS, the UK MS Society, and Merck, and honoraria from Biogen, BMS Celgene, Ipsen, Janssen, Merck, Novartis, Roche, and Sanofi‐Genzyme. M.P. has received research grants from the Italian MS Foundation and Baroni Foundation, honoraria from Health & Life and Biogen, and sponsorship for travel/meeting expenses from Novartis, Roche, and Merck. R.L. has received honoraria from Biogen, Merck, Novartis, Roche, and Teva. V.B.M. has received research grants from the Italian MS Society and Roche, and honoraria from Bayer, Biogen, Merck, Mylan, Novartis, Roche, Sanofi‐Genzyme, and Teva. A.C. has received research grants from Almirall, research grants from ECTRIMS‐MAGNIMS, and honoraria from Almirall, Biogen, Roche, Sanofi‐Genzyme, Merck, Ipsen, and Novartis. None of the other authors has any conflict of interest to disclose.

## OPR‐091

### Failure to suppress effector B cells is associated with resistance to ocrelizumab treatment in multiple sclerosis

#### R. Türkoğlu^1^; E. Tuzun ^2^; E. Akbayır^3^; T. Kızılay^1^; R. Erol^1^; V. Yılmaz^2^

##### ^1^Department of Neurology, Istanbul Haydarpasa Numune Training and Research Hospital, Istanbul, Turkey; ^2^Department of Neuroscience, Aziz Sancar Institute for Experimental Medical Research, Istanbul University, Istanbul, Turkey; ^3^Department of Language and Speech Therapy, Faculty of Health Sciences, Istanbul Atlas University, Istanbul, Turkey

**Background and Aims:** The aim of this study was to explore the utility of peripheral blood cell subsets in prediction of treatment response to ocrelizumab, a CD20‐targeting monoclonal antibody, in relapsing remitting multiple sclerosis (RRMS).

**Methods:** Thirty‐one patients with RRMS resistant to first‐line immunomodulating agents were enrolled and followed‐up for 12 months under ocrelizumab treatment. Disease activity was monitored by 6‐monthly assessments of EDSS and cranial‐spinal magnetic resonance imaging. No evidence of disease activity (NEDA‐3) status was determined, and peripheral blood mononuclear cells were immunophenotyped by flow cytometry.

**Results:** NEDA‐3 status was achieved by 19 patients, who exhibited elevated baseline populations of regulatory CD49d+ T‐ and B‐1a‐cells and reduced post‐treatment (month 6 or 12) populations of switched memory B‐cells. Flow cytometry analysis of the intracytoplasmic cytokine production revealed increased ratios of CD19+IL‐10+, CD19+IL‐35+ and CD19+TGFβ+ cell subsets, which negatively correlated with EDSS and/or attack numbers. Despite a moderate elevation of serum BAFF levels at month‐6, ocrelizumab treatment significantly reduced BAFF‐mediated CD19+ and CD19+CXCR5+ B cell chemotaxis.

**Conclusion:** Response to ocrelizumab is linked to regulatory and effector B‐cell subset ratios. Specific B‐cell subsets may serve as markers of treatment efficacy for ocrelizumab.

**Disclosure:** Nothing to disclose.

## OPR‐092

### Real‐world efficacy and safety of off‐label rituximab in a cohort of middle eastern multiple sclerosis patients

#### G. Ismail ^1^; M. Zeineddine^2^; R. Al‐Roughani^3^; S. Farouk Ahmed^4^; A. Al‐Mahdawi^5^; S. Khoury^6^; N. El‐Ayoubi^6^; J. Inshasi^7^; J. Al‐Khabouri^8^; A. Al‐Asmi^9^; R. Gouider^10^; S. Aljarallah^11^; N. Alkhawajah^11^; Y. Al Malik^12^; A. Abulaban^12^; S. Makkawi^13^; O. Khojah^13^; T. El‐Hajj^14^; J. Massouh^15^; H. AlSalamat^16^; A. Al‐Hajje^17^; P. Salameh^17^; F. Boumediene^1^; B. Yamout^15^

##### ^1^Inserm U1094, IRD U270, Univ. Limoges, CHU Limoges, EpiMaCT ‐ Epidemiology of chronic diseases in tropical zone, Institute of Epidemiology and Tropical Neurology, Omega Health, Limoges, France; ^2^2School of Pharmacy, Lebanese American University, Byblos, Lebanon; ^3^Amiri Hospital, Kuwait, Kuwait; ^4^Ibn Sina Hospital, Kuwait, Kuwait; ^5^Baghdad Medical City Teaching Hospital, Baghdad, Iraq; ^6^American University of Beirut Medical Center, Nehme and Therese Tohme Multiple Sclerosis Center, Beirut, Lebanon; ^7^MS Department, Rashid Hospital and Dubai Medical College, Dubai Health Authority, Dubai, United Arab Emirates; ^8^Department of Neurology, The Royal Hospital, Muscat, Oman; ^9^Neurology Unit, Department of Medicine, College of Medicine and Health Sciences and Sultan Qaboos University Hospital, Sultan Qaboos University, Muscat, Oman; ^10^Department of Neurology, LR18SP03, Clinical Investigation Center “Neurosciences and Mental Health”‐ Razi University Hospital‐ Mannouba, Tunis, Tunisia; ^11^King Saud University, King Saud University Medical City, Riyadh, Saudi Arabia; ^12^College of Medicine, King Saud Bin Abdulaziz University for Health Sciences, Riyadh, Saudi Arabia; ^13^College of Medicine, King Saud Bin Abdulaziz University for Health Sciences, Jeddah, Saudi Arabia; ^14^Faculty of Medical Sciences, Lebanese University, Beirut, Lebanon; ^15^Neurology Institute, Harley Street Medical Centre, Abu Dhabi, United Arab Emirates; ^16^Faculty of Medicine, Al‐Balqa Applied University, Al‐Salt 19117, Jordan; ^17^Faculty of Pharmacy, Lebanese University, Beirut, Lebanon

**Background and Aims:** This study investigates the efficacy and safety of rituximab in Middle Eastern multiple sclerosis (MS) patients in a clinical practice setting.

**Methods:** This was a multicenter, observational, retrospective study including MS patients treated with off‐label rituximab from 7 Middle Eastern countries by analyzing data from the MENACTRIMS registry. The primary efficacy outcome was the annualized relapse rate (ARR). Data on disability progression and magnetic resonance imaging (MRI) activity were collected from medical charts.

**Results:** A total of 774 MS patients were included in the study: 482 relapsing‐remitting MS (RRMS) and 292 progressive‐relapsing MS (PRMS). Treatment consisted of 500 mg or 1000 mg rituximab IV every 6–12 months. The cohort was predominantly female (72.1%), with a mean (SD) age of 39.6 (9.1) years and mean (SD) disease duration of 11.9 (6.3) years from symptom onset. The ARR decreased significantly from 1.65 at baseline to 0.18 during treatment in RRMS and from 2.03 to 0.02 in PRMS (*p* < 0.001). The median Expanded Disability Status Scale (EDSS) remained unchanged in RRMS, while it significantly increased by 1.0 in PRMS (*p* < 0.001). No MRI lesions were reported in 89.1% of RRMS compared to 8.1% of PRMS. NEDA‐3 was achieved in 47.3 % of RRMS. A total of 491 adverse events (AEs) were observed, primarily mild infusion‐related reactions (92.4%). Only four patients experienced serious AEs requiring hospitalization.

**Figure:**
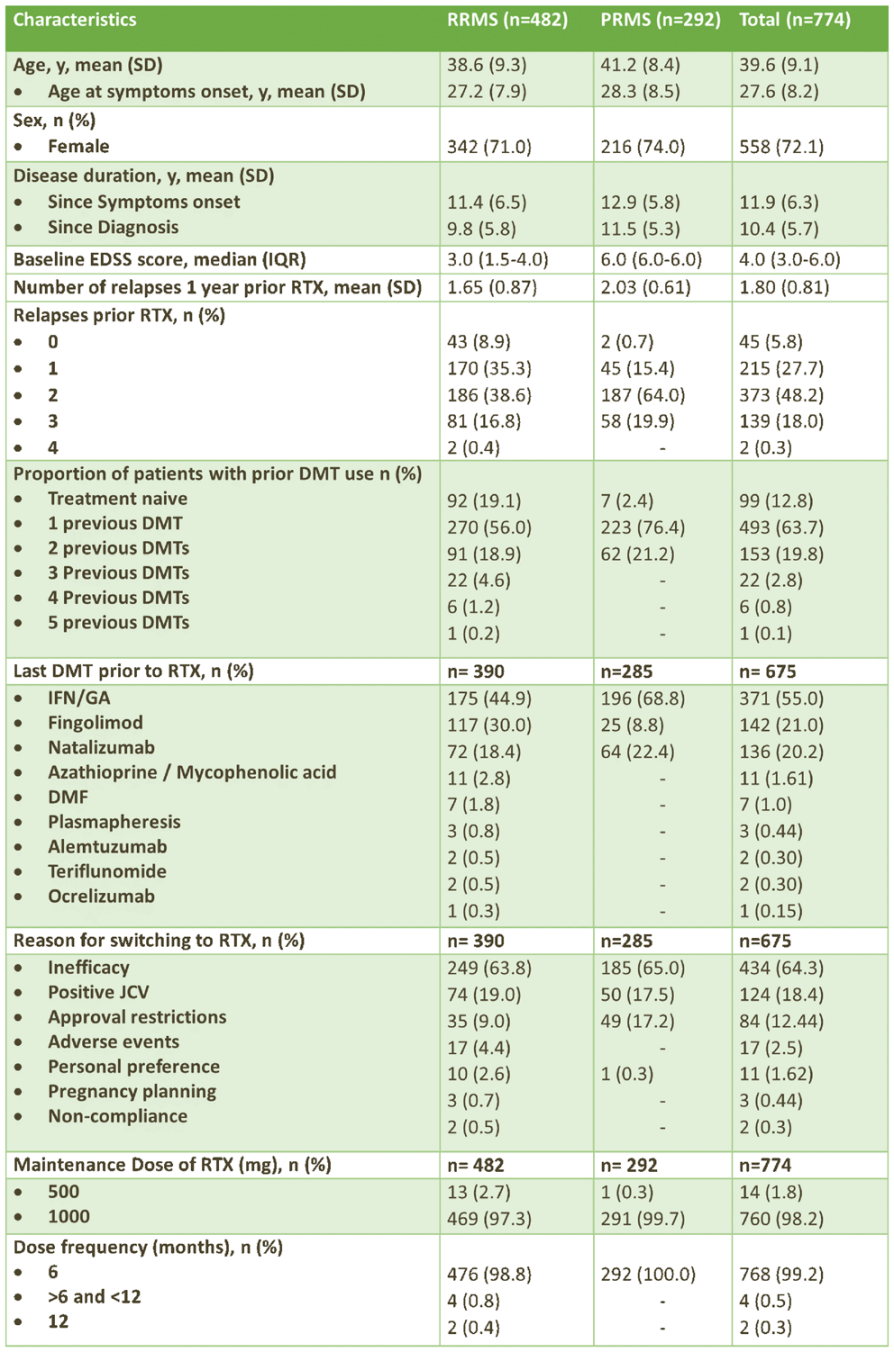
**TABLE 1** Baseline clinical and demographic data of MS patients.

**Figure:**
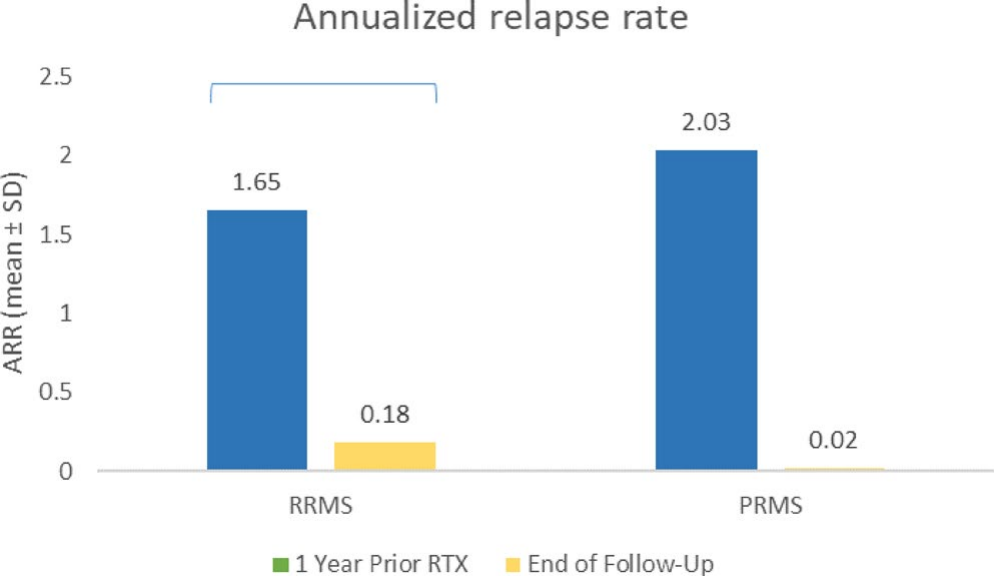
**FIGURE 1** Annualized relapse rate pre‐ and post‐rituximab.

**Figure:**
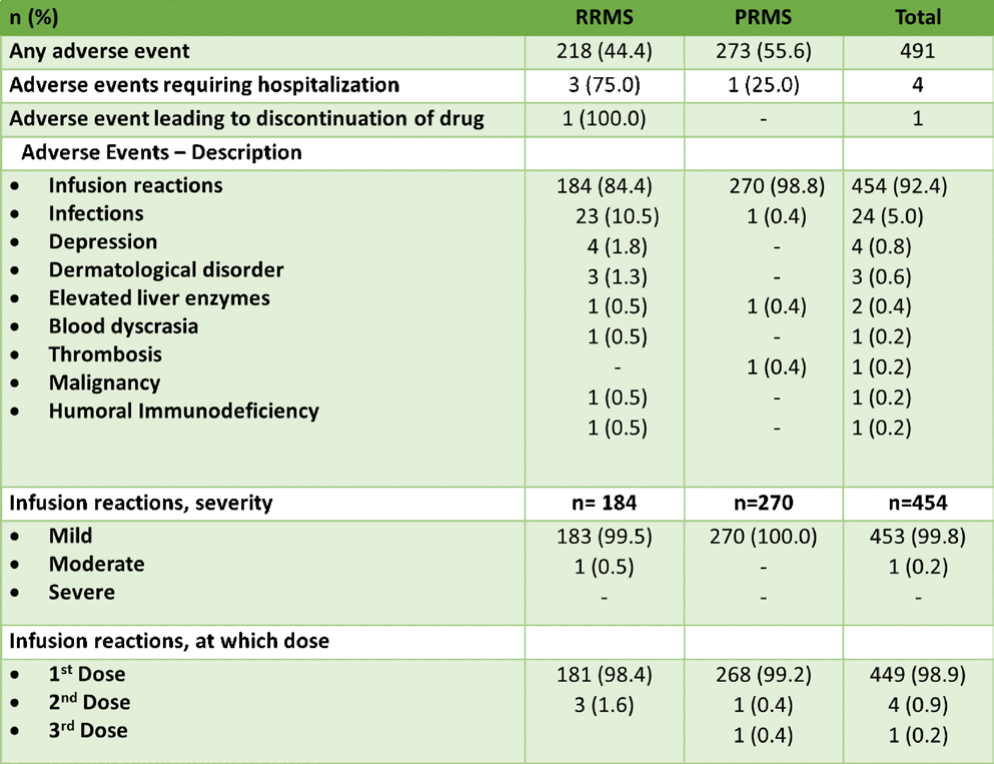
**TABLE 2** Safety profile of rituximab.

**Conclusion:** Off‐label rituximab demonstrated significant real‐world efficacy by reducing relapses in RRMS and PRMS patients, stabilizing disability and MRI findings in RRMS, and maintaining a well‐tolerated safety profile.

**Disclosure:** Nothing to disclose.

## OPR‐093

### Retinal microglia: A marker of inflammation or neurodegeneration in patients with multiple sclerosis?

#### V. A. Mauceri ^1^; C. Lapucci^3,4^; G. Boffa^3,4^; E. Cipriano^4^; M. Ponzano^5^; M. M. Fedriga^5^; F. Bovis^5^; E. Basili^1^; F. Rinaldi^2^; P. Perini^2^; P. Gallo^1,2^; M. Inglese^3,4^; M. Puthenparampil^1,2^

##### ^1^Università degli Studi di Padova, Department of Neuroscience, Padua, Italy; ^2^Azienda Ospedale Università Padova, Padua, Italy; ^3^IRCCS Ospedale Policlinico San Martino, Genoa, Italy; ^4^Department of Neurology, Rehabilitation, Ophthalmology, Genetics, Maternal and Child Health, University of Genoa, Genoa, Italy; ^5^Department of Health Sciences, University of Genoa, Genoa, Italy

**Background and Aims:** Retinal hyperreflective foci (HRF) are biomarkers of microglial activation in multiple sclerosis (MS). Studies show increased HRF in MS patients compared to controls, correlating with pro‐inflammatory cytokines in cerebrospinal fluid (CSF), radiological activity, and cortical lesions. HRF levels appear unaffected by treatments like Natalizumab, suggesting microglial activation is independent of adaptive immunity. HRF have also been identified in the outer retina of relapsing‐remitting MS (RRMS) patients. This study aimed to: (1) investigate the correlation between HRF and brain volumes as neurodegeneration markers, (2) explore relationships between HRF and CSF biomarkers of neuroinflammation (YKL‐40), neurodegeneration (NfLs), and intrathecal inflammation (IgG index), and (3) assess HRF presence in the outer retina of RRMS patients and controls.

**Methods:** Sixty‐six RRMS patients and 33 controls underwent neurological exams, lumbar puncture, optical coherence tomography (OCT), and MRI. OCT measured retinal thickness and volumes, with HRF independently counted by two observers. Brain MRI quantified gray (GM) and white matter (WM) volumes and inflammatory lesions. CSF levels of NfLs and YKL‐40 were analyzed via ELISA. Generalized estimating equation models adjusted for age, sex, and optic neuritis history were used.

**Figure:**
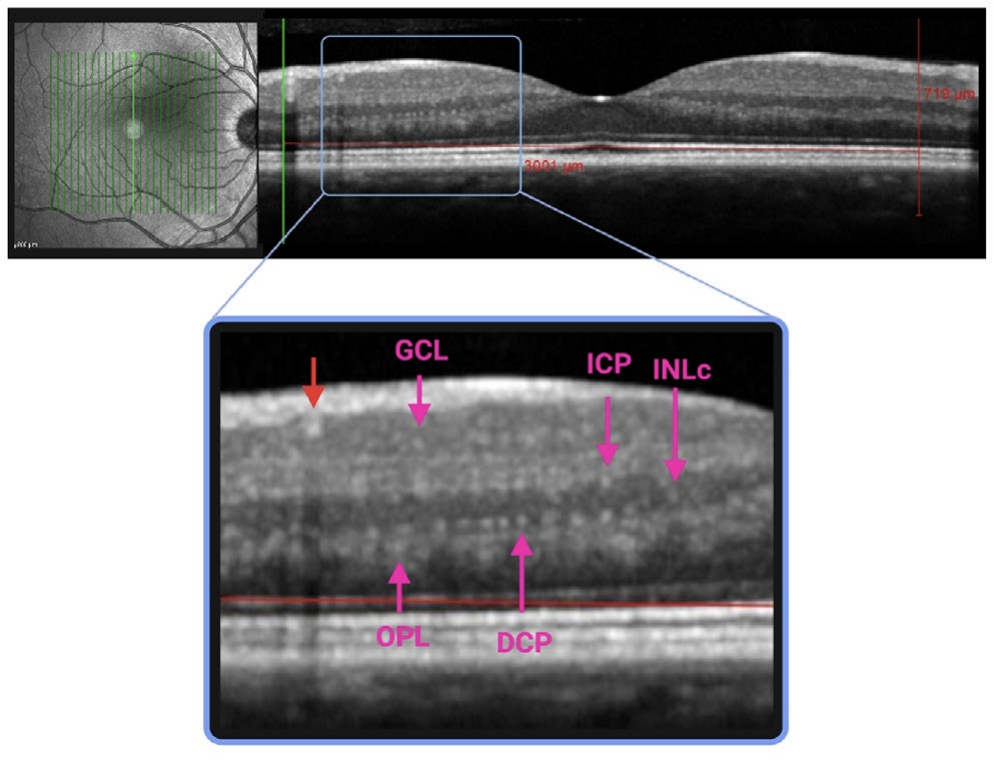
**FIGURE 1** Linear scan centered on the macula and passing through the fovea. The magenta arrows indicate retinal HRF in the ganglion cell layer (GCL), near the intermediate capillary plexus (ICP), near the deep capillary plexus (DCP), and in the central portion of t.

**Results:** RRMS patients had significantly higher HRF across all retinal layers (*p* < 0.001). GCPL HRF correlated positively with WM (β = 0.0022; *p* < 0.001), GM (β = 0.0017; *p* < 0.001), and YKL‐40 (β = 0.11; *p* = 0.018), but not NfLs. OPL‐ONL HRF correlated negatively with the IgG index (β = −0.23; *p* = 0.017).

**Figure:**
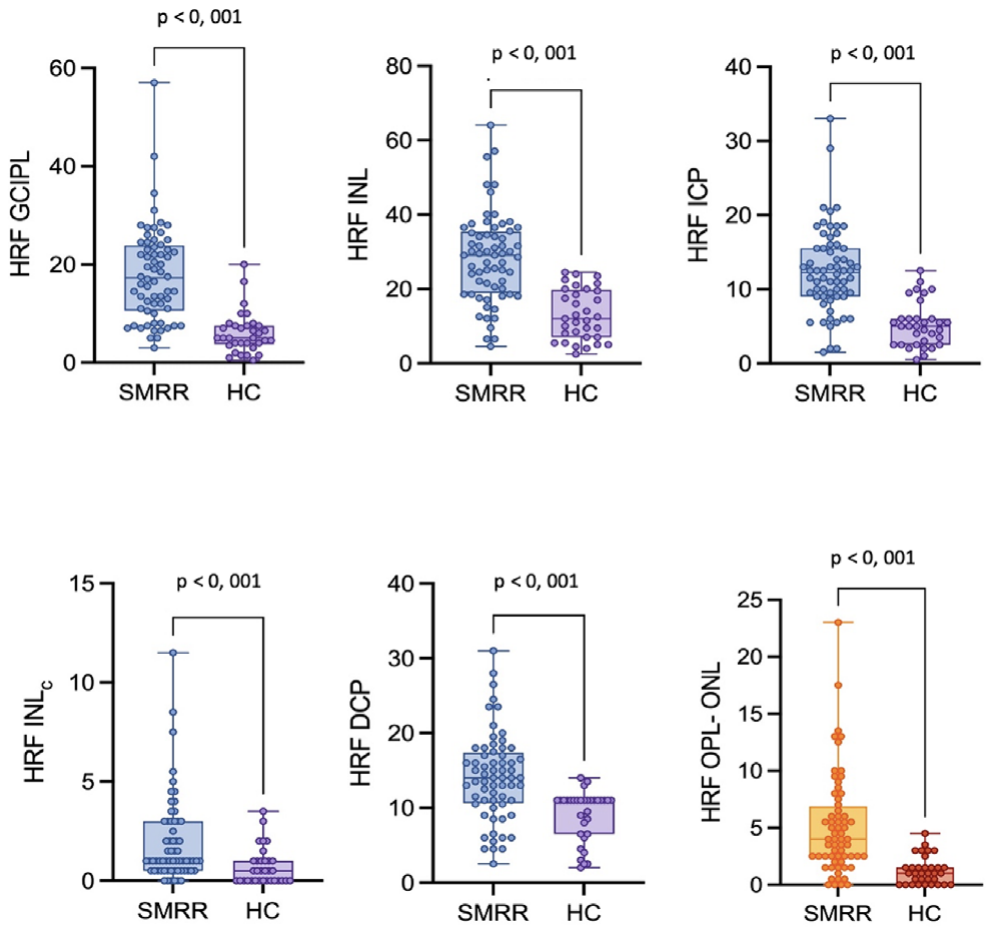
**FIGURE 2** Hyper‐reflective foci (HRF) in the various retinal layers in patients with RRMS and healthy controls (HC). The HRF count is significantly higher in RRMS patients compared to healthy controls across all retinal layers considered. Blue and purple indicate H.

**Figure:**
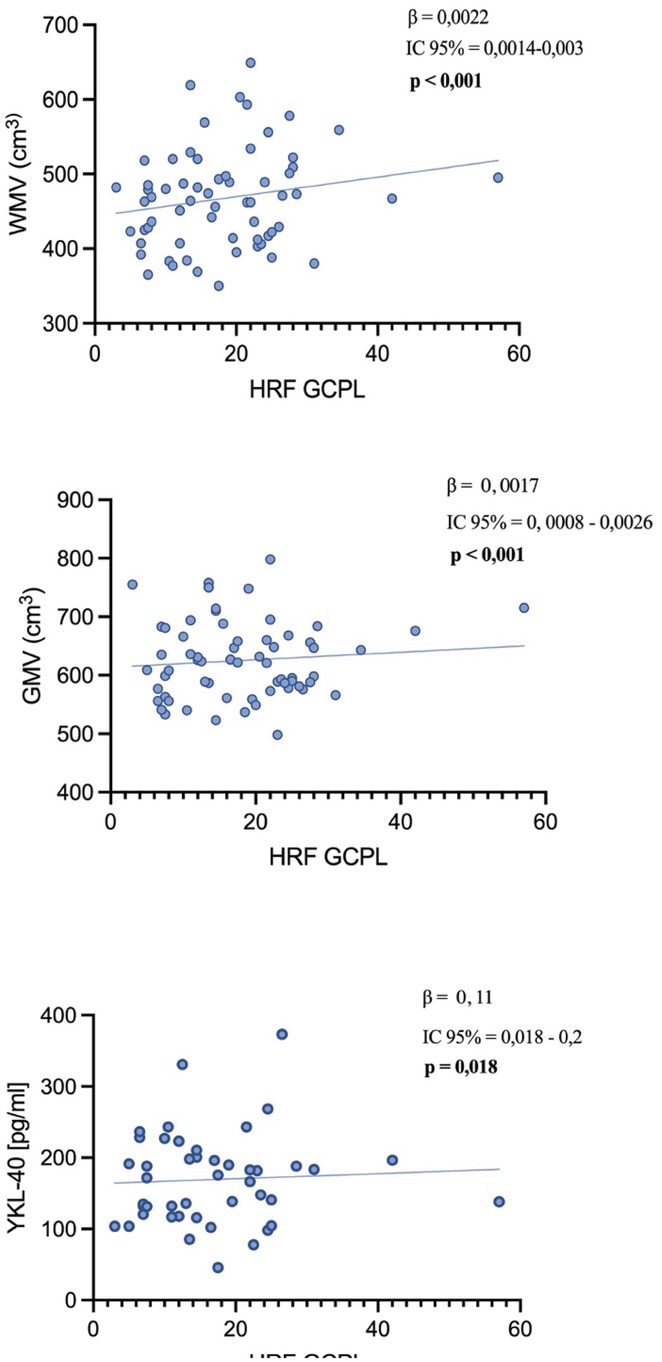
**FIGURE 3** Association of GCPL HRF with white matter (WM) and gray matter (GM) volumes, and the amount of chitinase (YKL‐40) in cerebrospinal fluid in pg/ml in patients with RRMS.

**Conclusion:** HRF, particularly in GCPL, may indicate neuroinflammation rather than neurodegeneration in RRMS.

**Disclosure:** M.Pu., report grants from Almirall, Teva, Sanofi Genzyme, Merck Serono, Biogen Italy, Novartis; consultancy for Novartis, Biogen Italy, Sanofi Genzyme; board membership Sanofi Genzyme, Novartis, Biogen Italy. VAM report grants from Sanofi Genzyme, Viatris. EB has nothing to disclose. P.P. reports grants from Almirall, Teva, Sanofi Genzyme, Merck Serono, Biogen Italy, Novartis, Roche; consultancy for Novartis, Biogen Italy, Sanofi Genzyme, Roche. RF report grants from Almirall, Teva, Sanofi Genzyme, Merck Serono, Biogen Italy, Novartis; consultancy for Novartis, Biogen Italy, Sanofi Genzyme. P.G. reports grant from Almirall, Teva, Sanofi Genzyme, Merck Serono, Biogen Italy, Novartis, Roche, Bristol Myers Squibb; consultancy for Novartis, Biogen Italy, Sanofi Genzyme, Roche, Bristol Myers Squibb; board membership Sanofi Genzyme, Novartis, Biogen Italy, Roche, Merck Serono, Bristol Myers Squibb.

Movement Disorders 2

## OPR‐094

### Significant weight loss in Parkinson´s disease. A 5‐year follow‐up study

#### D. Santos García ^1^; T. de Deus Fonticoba^2^; S. Jesús^3^; M. Cosgaya^4^; J. García Caldentey^5^; N. Caballol^6^; I. Legarda^7^; J. Hernández Vara^8^; I. Cabo^9^; L. López‐Manzanares^10^; I. González‐Aramburu^11^; A. Ávila Rivera^12^; V. Gómez Mayordomo^13^; V. Nogueira^14^; J. Dotor García‐Soto^15^; C. Borrue^16^; B. Solano^17^; M. Álvarez‐Sauco^18^; P. Mir^3^; S. COPPADIS^19^

##### ^1^Neurology Department, CHUAC (Complejo Hospitalario Universitario de A Coruña), A Coruña, Spain; ^2^Complejo Hospitalario Universitario de Ferrol, A Coruña, Spain; ^3^Instituto de Biomedicina de Sevilla, Hospital Universitario Virgen del Rocío/CSIC/Universidad de Sevilla, Seville, Spain; ^4^Hospital Clínic de Barcelona, Barcelona, Spain; ^5^Centro Neurológico Oms 42, Palma de Mallorca, Spain; ^6^Consorci Sanitari Integral, Hospital Moisés Broggi, Sant Joan Despí, Barcelona, Spain; ^7^Hospital Universitario Son Espases, Palma de Mallorca, Spain; ^8^Hospital Universitario Vall d´Hebron, Barcelona, Spain; ^9^Complejo Hospitalario Universitario de Pontevedra (CHOP), Pontevedra, Spain; ^10^Hospital Universitario La Princesa, Madrid, Spain; ^11^Hospital Universitario Marqués de Valdecilla ‐ IDIVAL, Santander, Spain; ^12^Consorci Sanitari Integral, Hospital General de L´Hospitalet, L´Hospitalet de Llobregat, Barcelona, Spain; ^13^Institute of Neuroscience, Vithas Madrid La Milagrosa University Hospital, Vithas Hospital Group, Madrid, Spain; ^14^Hospital Da Costa de Burela, Lugo, Spain; ^15^Hospital Universitario Virgen Macarena, Sevilla, Spain; ^16^Hospital Infanta Sofía, Madrid, Spain; ^17^Institut d'Assistència Sanitària (IAS) ‐ Institut Català de la Salut. Girona, Spain; ^18^Hospital General Universitario de Elche, Elche, Spain; ^19^Fundación Degen ‐ COPPADIS Study Group

**Background and Aims:** Significant weight loss (SWL) is considered a common complication of Parkinson's disease (PD). Our aim was (1) to compare the frequency of SWL in PD patients vs. controls, (2) to identify predictors of SWL in PD, and (3) to analyze the relationship between SWL and quality of life (QoL) and autonomy for activities of daily‐living (ADL).

**Methods:** In this prospective 5‐year follow‐up population‐based observational study, PD patients and controls from the COPPADIS cohort (Santos‐García et al. 2016) with repetitive weight examinations over 5 years were included. A decrease > 10% in weight at 5 years (V5) compared to baseline (V0) was defined as SWL (Kristiansen et al. 2024). Regression models were applied to identify predictors of SWL.

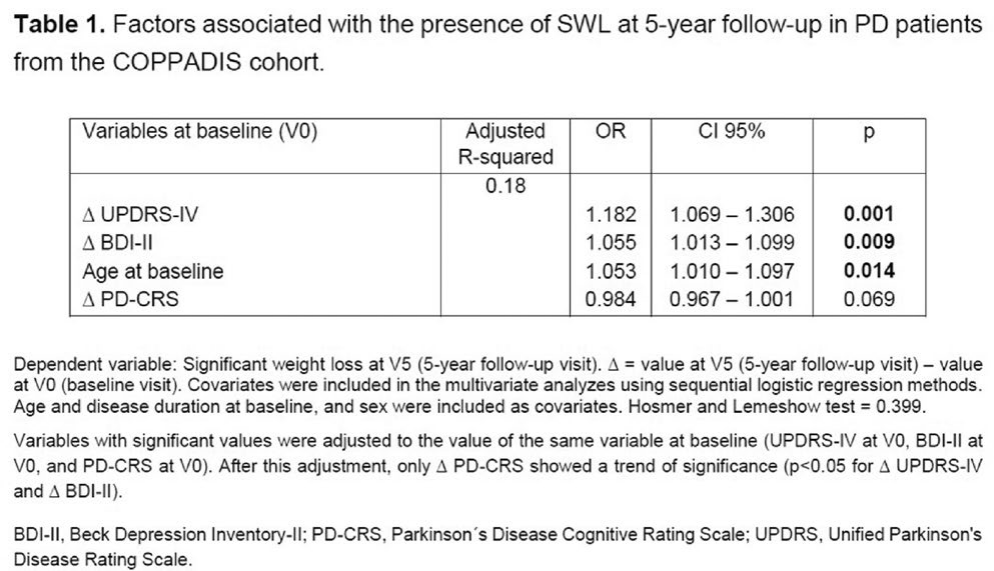

**Results:** Mean weight decreased in PD patients (N = 407; 61.9 ± 8.9 years old, 57% males) from 75.5 ± 13.2 at V0 to 73.8 ± 13.5 at V5 (*p* < 0.0001) but not in controls (N = 110; 61.9 ± 7.4 years old, 52.7% males; from 75.9 ± 13.8 at V0 to 76.3 ± 15.1 at V5 [*p* = 0.878]). The frequency of SWL was twice as high in PD patients than in controls (18.2% [74/407] vs 9.1% [10/110]; *p* = 0.013). SWL at V5 was associated with a worse QoL and autonomy for ADL (*p* < 0.0001; Figure 1). To be older (*p* = 0.014) and an impairment from V0 to V5 in motor complications (UPDRS‐IV) (*p* = 0.001) and mood (BDI‐II) (*p* = 0.009) were associated with SWL (Table 1).

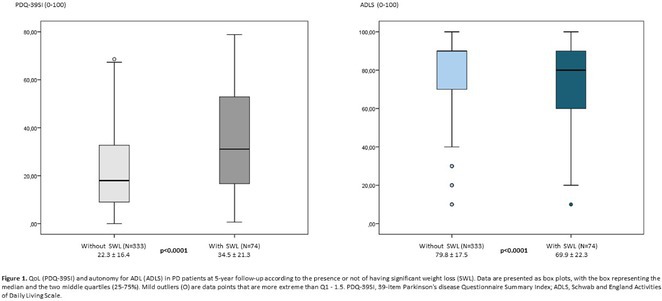

**Conclusion:** SWL is frequent in PD and is associated to a worse QoL and decreased autonomy for ADL.

**Disclosure:** The authors report no conflict of interest.

## OPR‐095

### Multidomain cognitive tele‐rehabilitation for Parkinson's disease with mild‐to‐moderate cognitive decline

#### G. Arabia ^1^; J. Buonocore^1^; G. Torchia^1^; F. Curcio^1^; F. Pirrotta^1^; M. Contrada^2^; C. Pucci^2^; A. Gambardella^1^; A. Quattrone^3^; A. Pilotto^4^; L. Pignolo^2^

##### ^1^Institute of Neurology, Department of Medical and Surgical Sciences, “Magna Graecia” University of Catanzaro, Italy; ^2^Regional Center for Serious Brain Injuries, S. Anna Institute, Crotone, Italy; ^3^Neuroscience Research Center, Magna Graecia University, Catanzaro, Italy; ^4^Geriatrics Unit, Department Geriatric Care, Orthogeriatrics and Rehabilitation, E.O. Galliera Hospital, Genoa, Italy

**Background and Aims:** Cognitive decline is a non‐motor symptom of Parkinson's disease (PD) that significantly impairs quality of life. Cognitive stimulation (CS) has demonstrated efficacy in enhancing cognitive function in PD‐related cognitive impairment. However, logistical challenges, like mobility limitations, frequently restrict access to regular in‐person CS programs. Telerehabilitation offers a promising, home‐based alternative that uses technology to deliver personalized interventions. This study aims to evaluate the efficacy of remote CS in individuals with mild‐to‐moderate PD‐related cognitive impairment.

**Methods:** Forty‐five PD patients were randomized into a tele‐rehabilitation group (TRG, *n* = 25) or a control group (CG, *n* = 20). The TRG underwent daily remote CS sessions, while the CG performed traditional paper‐and‐pencil‐based cognitive exercises. Clinical and neuropsychological assessments were conducted at baseline, immediately post‐intervention, and six months post‐intervention.

**Results:** In the TRG, participants significantly improved in executive, attentional, and visuospatial abilities, demonstrating the effectiveness of tele‐rehabilitation in addressing key cognitive deficits in PD. The CG showed similar benefits associated with a significant reduction in depressive symptoms, highlighting the added benefits of social interactions in traditional approaches. The cognitive decline observed at the six‐month follow‐up suggests the need for sustained engagement and long‐term interventions to preserve these benefits over time.

**Conclusion:** Telerehabilitation effectively enhances cognitive domains such as attention, executive function, and visuospatial skills, presenting a feasible and accessible solution for individuals with PD and mild‐to‐moderate cognitive decline. Future approaches may benefit from hybrid models that combine telerehabilitation's accessibility with the psychosocial benefits of in‐person interventions to maximize cognitive and emotional outcomes for PD patients.

**Disclosure:** This work was supported by the Ministry of Health, project title: “Development and implementation of common strategy for the management of community‐ dwelling older subjects with multimorbidity and polypharmacy: integration with a multicomponent intervention platform by using domotic, robotic and telecare systems. MULTIPLAT_AGE”; project code: NET‐2016‐02361805.

## OPR‐096

### LRRK2‐related PD: Hints at increased cancer risk

#### G. Di Rauso ^1^; F. Pirone^2^; G. Franco^2^; F. Arienti^2^; I. Trezzi^2^; E. Frattini^2^; G. Toschi^3^; V. Fioravanti^3^; F. Cavallieri^3^; F. Valzania^3^; E. Monfrini^2^; A. Di Fonzo^2^

##### ^1^Clinical and Experimental Medicine PhD Program, University of Modena and Reggio Emilia, Modena, Italy; ^2^Foundation IRCCS Ca' Granda Ospedale Maggiore Policlinico, Neurology Unit, Milan, Italy; ^3^Neurology Unit, Neuromotor & Rehabilitation Department, Azienda USL‐IRCCS di Reggio Emilia, Reggio Emilia, Italy

**Background and Aims:** Pathogenic variants in the LRRK2 gene are the most common monogenic cause of Parkinson's Disease (PD). Previous in‐vitro and mouse model studies suggested that pathological variants in LRRK2 gene may contribute to tumorigenesis. The aim of this study is to examine whether PD patients carrying pathogenic variants in LRRK2 gene (mutLRRK2‐PD) have a higher prevalence of malignancies compared to PD non mutated in LRRK2 gene (wtLRRK2‐PD).

**Methods:** We included only PD patients genetically tested for PD‐associated genes from the Foundation IRCCS Ca’ Granda Ospedale Maggiore Policlinico of Milan and from the AUSL‐IRCCS of Reggio Emilia. We retrospectively collected data on personal history of malignancies in mutLRRK2‐PD and wtLRRK2‐PD patients to compare tumor prevalence between these groups.

**Results:** 519 PD patients were included: 477 wtLRRK2‐PD (male: 300) and 42 mutLRRK2‐PD (male: 21). mutLRRK2‐PD patients had a significantly greater prevalence of oncological disease compared to wtLRRK2‐PD (33% vs. 19%, *p* = 0.03). All mutLRRK2‐PD patients with cancers carried the G2019S variant, except for five. The most prevalent malingnancy in the mutLRRK2‐PD cohort was cutaneous melanomas.

**Conclusion:** This study suggests that mutLRRK2‐PD patients may be more susceptible to specific malignancies compared to PD patients without LRRK2 mutations. If confirmed, the management of these patients could be enhanced by providing not only follow‐up care for PD, but also early malignancy screening.

**Disclosure:** The authors have no conflicts of interest to declare that are relevant to the content of this abstract.

## OPR‐097

### Spatiotemporal Deep Neural Networks for differentiation of iRBD, Parkinson's Disease, and controls using rs‐fMRI

#### S. Basaia ^1^; S. Pisano^1^; T. Filaferro^2^; E. Sarasso^3^; A. Gardoni^4^; S. Marelli^5^; R. Balestrino^6^; L. Zenere^1^; A. Castelnuovo^5^; M. Malcangi^6^; L. Ferini‐Strambi^5^; R. De Micco^7^; A. Tessitore^7^; M. Salvi^8^; F. Molinari^8^; F. Agosta^9^; M. Filippi^10^

##### ^1^Neuroimaging Research Unit, Division of Neuroscience, IRCCS San Raffaele Scientific Institute, Milan, Italy; ^2^Neuroimaging Research Unit, Division of Neuroscience, IRCCS San Raffaele Scientific Institute, Milan, Italy; and Biolab, PoliTo(BIO)Med Lab, Department of Electronics and Telecommunications, Politecnico di Torino, Torino, Italy; ^3^Neuroimaging Research Unit, Division of Neuroscience, and Department of Rehabilitation and Functional Recovery, IRCCS San Raffaele Scientific Institute, Milan, Italy; and DINOGMI, University of Genoa, Genoa, Italy; ^4^Neuroimaging Research Unit, Division of Neuroscience, IRCCS San Raffaele Scientific Institute, and Vita‐Salute San Raffaele University, Milan, Italy; ^5^Vita‐Salute San Raffaele University, Milan, Italy; and Sleep Disorders Center, Division of Neuroscience, IRCCS San Raffaele Scientific Institute, Milan, Italy; ^6^Neurology Unit, IRCCS San Raffaele Scientific Institute, Milan, Italy; ^7^Department of Advanced Medical and Surgical Sciences, University of Campania “Luigi Vanvitelli”, Napoli, Italy; ^8^Biolab, PoliTo(BIO)Med Lab, Department of Electronics and Telecommunications, Politecnico di Torino, Torino, Italy; ^9^Neuroimaging Research Unit, Division of Neuroscience, and Neurology Unit, IRCCS San Raffaele Scientific Institute, and Vita‐Salute San Raffaele University, Milan, Italy; ^10^Neurology Unit, Neurorehabilitation Unit, Neurophysiology Service, and Neuroimaging Research Unit, Division of Neuroscience, IRCCS San Raffaele Scientific Institute, and Vita‐Salute San Raffaele University, Milan, Italy

**Background and Aims:** This study aimed to develop and evaluate a spatiotemporal deep‐neural‐network (stDNN) leveraging resting‐state fMRI (rs‐fMRI) data to identify brain biomarkers associated with idiopathic REM sleep behavior disorder (iRBD) and Parkinson's disease (PD) and to differentiate these conditions from controls.

**Methods:** Data were collected from three cohorts: the IRCCS San Raffaele Scientific Institute (HSR), the Parkinson's Progression Markers Initiative (PPMI), and the Movement Disorders Unit at the University of Campania (MDU). The final sample included 771 subjects, comprising 423 patients with PD, 144 with iRBD, and 204 controls. stDNN model was applied to mean time series extracted for each subject from rs‐fMRI data. By integrating spatio‐temporal features, the network classified subjects based on distinct neural patterns. Model generalizability was assessed using a train‐fold‐validation split combined with k‐fold cross‐validation. Explainable artificial intelligence (XAI) methods were applied.

**Results:** stDNN achieved balanced accuracy rates of 74.9% in distinguishing controls from PD and up to 82.4% in moderate‐to‐severe PD cases. It also demonstrated over 80% accuracy in differentiating controls from iRBD. However, performance declined when differentiating iRBD from PD, likely due to overlapping functional characteristics. XAI analysis highlighted the involvement of the temporal pole, calcarine sulcus and precuneus in distinguishing controls from PD. Considering iRBD, the supplementary motor area (SMA), left superior temporal pole, and left middle temporal areas were identified as key regions.

**Conclusion:** This study demonstrates the potential of stDNN to differentiate between iRBD, PD, and controls using fMRI data.

**Disclosure:** Funding. Italian Ministry of Health [grant RF‐2018‐12366746]. Disclosures. SB, ES, SM, RB grant support from Italian Ministry of Health. AGa, LZ, MM, AGr, AC, DMR, TA, MS, FM nothing to disclose. LFS received speaker honoraria from Biprojet, Idorsia, Italfarmaco, and Takeda, and receives or has received research support from the Italian Ministry of Health and the Italian Ministry of University and Research. MF received compensation for consulting services or speaking activities from Alexion, Almirall, Bayer, Biogen, Celgene, Chiesi Italia SpA, Eli Lilly, Genzyme, Janssen, Merck‐Serono, Neopharmed Gentili, Novartis, Novo Nordisk, Roche, Sanofi Takeda, and TEVA; Advisory Boards for Alexion, Biogen, Bristol‐Myers Squibb, Merck, Novartis, Roche, Sanofi, Sanofi‐Aventis, Sanofi‐Genzyme, Takeda; scientific direction of educational events for Biogen, Merck, Roche, Celgene, Bristol‐Myers Squibb, Lilly, Novartis, Sanofi‐Genzyme; he receives research support from Biogen Idec, Merck‐Serono, Novartis, Roche, the Italian Ministry of Health, the Italian Ministry of University and Research, and FISM. FA received speaker honoraria from Biogen Idec, Roche, Eli Lilly and GE Healthcare; and grants from Italian Ministry of Health, Italian Ministry of University and Research, AriSLA, European Research Council, EU Joint Programme—Neurodegenerative Disease Research, and Foundation Research on Alzheimer Disease (France).

## OPR‐098

### Inventory of non‐ataxia signs in the European friedreich's ataxia consortium for translational studies study

#### S. Lischewski ^1^; I. Dogan^1^; P. Giunti^2^; M. Parkinson^2^; C. Mariotti^3^; A. Durr^4^; C. Ewenczyk^4^; S. Boesch^5^; W. Nachbauer^5^; T. Klopstock^6^; C. Stendel^6^; F. de Rivera Garrido^7^; L. Schöls^8^; Z. Fleszar^8^; T. Klockgether^9^; M. Grobe‐Einsler^9^; I. Giordano^9^; M. Rai^10^; M. Pandolfo^11^; J. Schulz^1^; K. Reetz^1^

##### ^1^Department of Neurology, RWTH Aachen University, Aachen, Germany; ^2^Ataxia Centre, Department of Clinical and Movement Neurosciences, UCL‐Queen Square Institute of Neurology, London, UK; ^3^Unit of Medical Genetics and Neurogenetics, Fondazione IRCCS Istituto Neurologico Carlo Besta, Milan, Italy; ^4^Sorbonne Universite, Paris Brain Institute, ICM Institut du Cerveau, AP‐HP, INSERM, CNRS, University Hospital Pitié‐Salpêtrière, Paris, France; ^5^Department of Neurology, Medical University Innsbruck, Innsbruck, Austria; ^6^Department of Neurology, Friedrich Baur Institute, University Hospital, LMU, Munich, Germany; German Center for Neurodegenerative Diseases, Munich, Germany; Munich Cluster for Systems Neurology, Munich, Germany; ^7^Reference Unit of Hereditary Ataxias and Paraplegias, Department of Neurology, IdiPAZ, Hospital Universitario La Paz, Madrid, Spain; ^8^Department of Neurology and Hertie‐Institute for Clinical Brain Research, University of Tübingen, Tübingen, Germany; ^9^German Center for Neurodegenerative Diseases, Bonn, Germany; ^10^Friedreich Ataxia Research Alliance, USA; ^11^Laboratory of Experimental Neurology, Université Libre de Bruxelles, Brussels, Belgium

**Background and Aims:** Friedreich ataxia is a rare neurodegenerative multisystem disorder. While ataxia is a hallmark, non‐ataxia symptoms and signs, including muscle weakness, spasticity and dysphagia are equally disabling. The Inventory of Non‐Ataxia Signs (INAS) is a 16‐item symptom list that can be transformed to a count. We sought to validate the INAS in this patient population.

**Methods:** Participants were drawn from the European Friedreich's Ataxia Consortium for Translational Studies (EFACTS). The INAS‐count (presence/absence, 0–16 scale) and newly‐derived INAS‐sum (severity‐weighted, 0–84 scale) were evaluated using linear mixed models and standardised response means (SRMs).

**Results:** 1129 participants (mean age 32.3) were assessed for up to 12 years. At baseline, the mean INAS‐count was 4.3 (±2.1), and INAS‐sum was 15.1 (± 9.9). Both showed strong correlations with existing outcome measures (SARA and ADL). Longitudinally, the INAS‐count increased by 0.15 points/year (95% 0.13, 0.16; *p* < 0.001) and INAS‐sum by 0.70 points/year (95% CIs 0.67, 0.76; *p* < 0.001). The INAS‐sum demonstrated greater responsiveness, with SRMs of 0.26, 0.38, 0.53 and 0.80 at 1, 2, 3, and 5 years, respectively, compared to 0.21, 0.34, 0.46 and 0.63 for the INAS‐count. In non‐ambulatory patients, responsiveness of the INAS‐sum was comparable at 3 years (SRM 0.47) and higher at 5 years (SRM 0.82).

**Figure:**
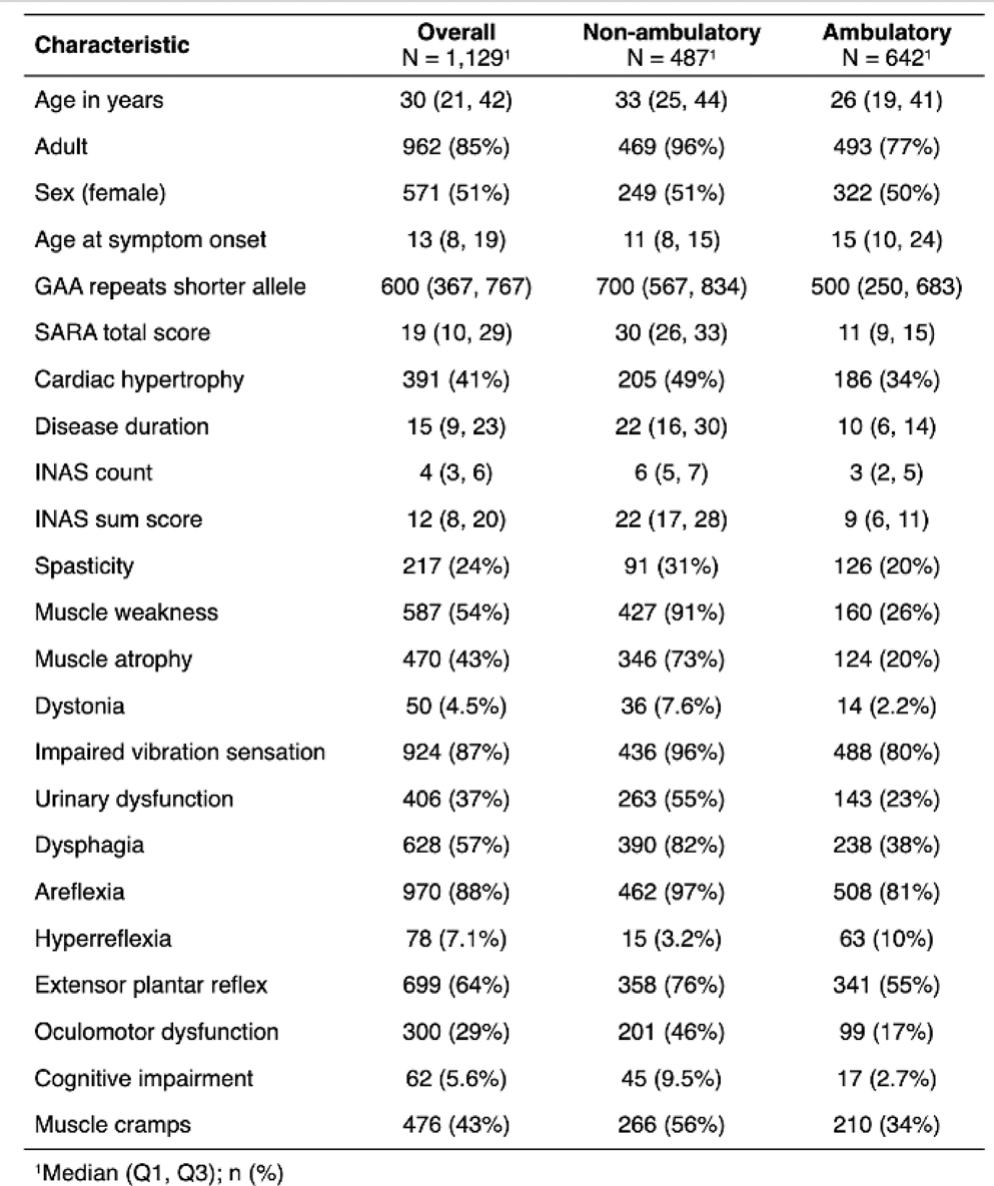
**TABLE 1** Main demographic and clinical characteristics of participants at baseline.

**Figure:**
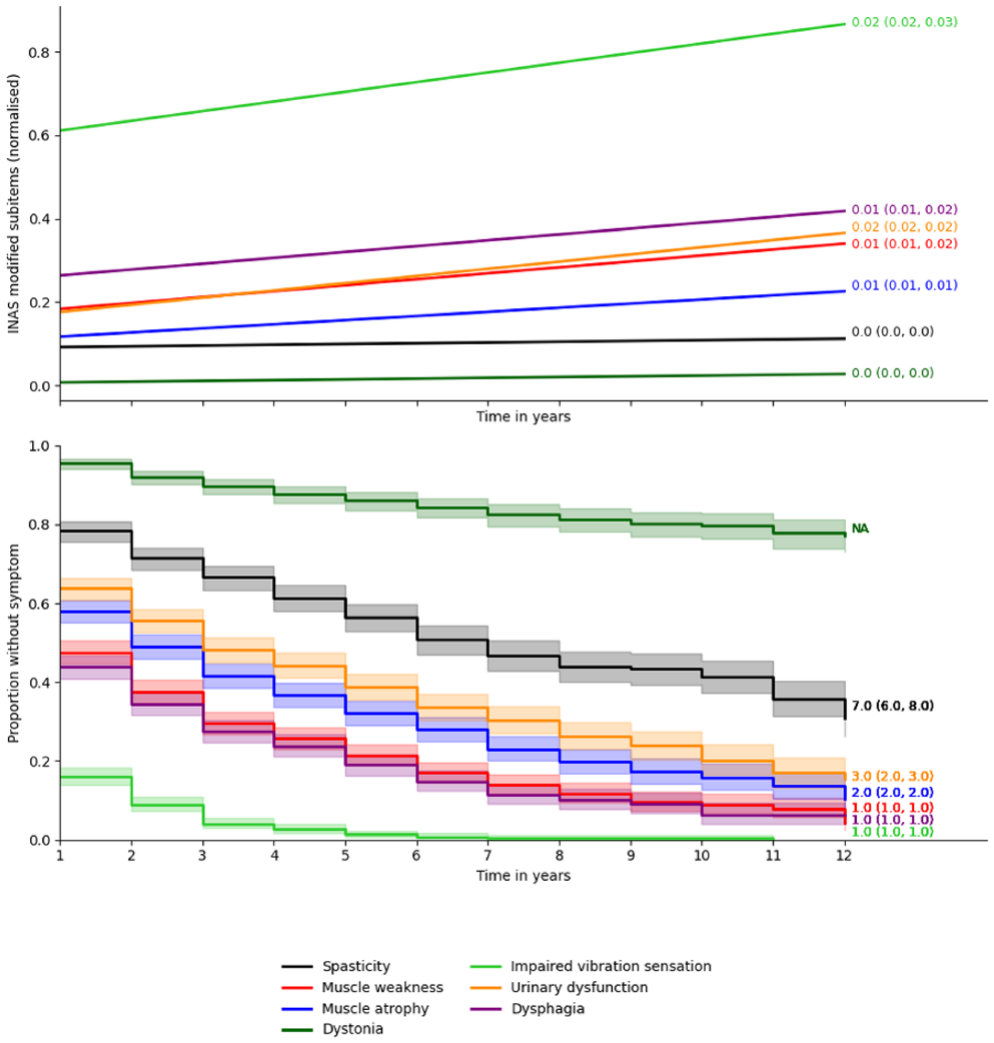
**FIGURE 1** Longitudinal evolution of INAS subitems over time.

**Figure:**
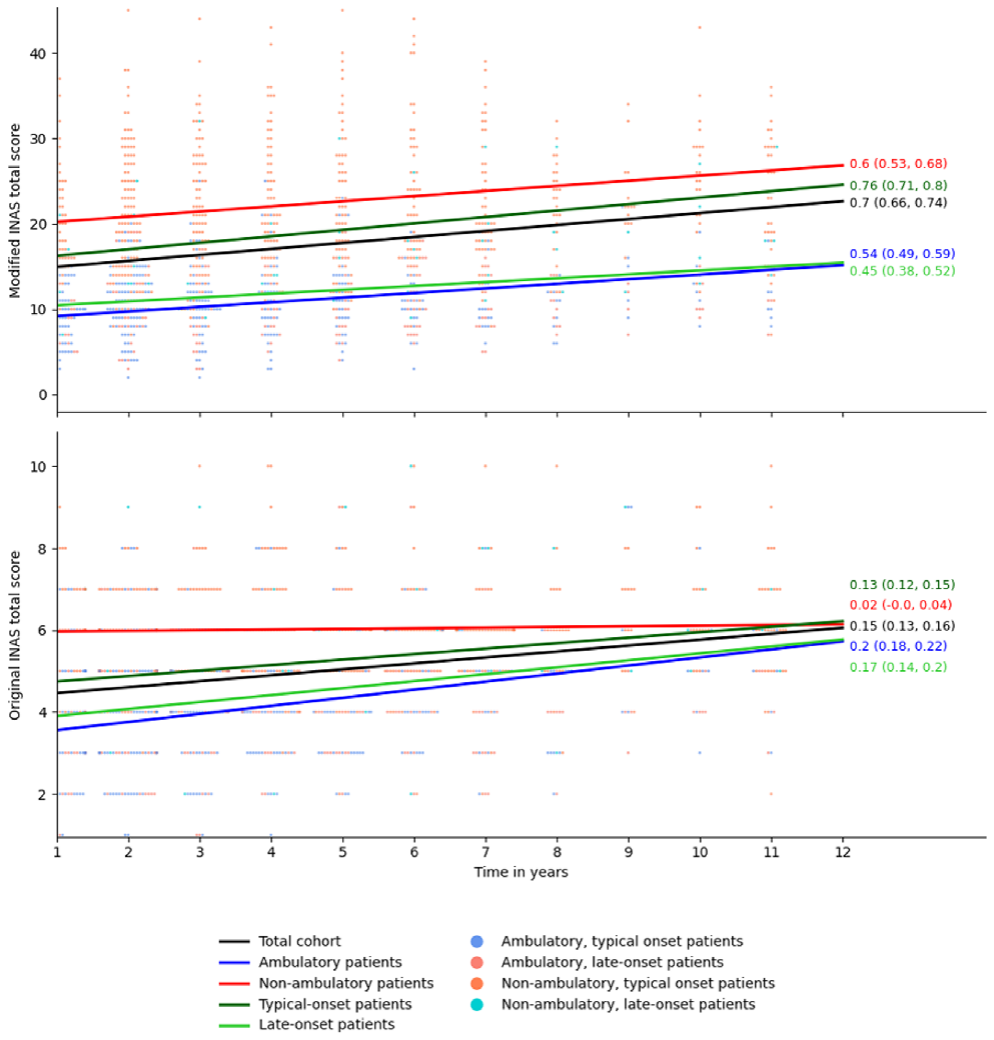
**FIGURE 2** Longitudinal evolution of the INAS summed and the INAS count total score.

**Conclusion:** The INAS‐sum showed good responsiveness in the medium‐term but not in the short‐term follow‐up. It may supplement existing outcome measures, contributing to a more holistic assessment of this multisystem disease, especially in non‐ambulatory patients, where ataxia‐focussed measures may be constrained by ceiling effects.

**Disclosure:** SL received speaker honoraria from Biogen unrelated to this study. MGE received research support from the German Ministry of Education and Research (BMBF) within the European Joint Program for Rare Diseases (EJP‐RD) 2021 Transnational Call for Rare Disease Research Projects (funding number 01GM2110), from the National Ataxia Foundation (NAF), Ataxia UK, and Biogen Germany, and received consulting fees from Healthcare Manufaktur, Germany, and Biogen Germany ‐ all unrelated to this study. W. Nachbauer has received speaker and advisory honoraria from Biogen and Reata Pharmaceuticals. S. Boesch reports consultancies from VICO Therapeutics, Reata pharmaceuticals and Biogen, Honoria from Ipsen, Merz, Abbvie and Reata and advisory boards for Biogen and Reata. L. Schöls received consultancies from VICO Therapeutics, Vigil Neuroscience and Novartis unrelated to this work. The remaining authors report no disclosures relevant to the abstract.

## Neuroimmunology 2

## OPR‐099

### Decoding the immune‐brain axis: Advancing neurovascular models for neuroinflammation and T cell trafficking research

#### E. Lauranzano ^1^; M. Ravanelli^1^; M. Rasile^2^; G. Liberatore^1^; C. Cutellè^1^; E. Faggiani^1^; E. Nobile‐Orazio^1^; M. Matteoli^1^

##### ^1^IRCCS Humanitas Clinical and Research Center, Rozzano (Milan), Italy; ^2^Humanitas University, Lab of Pharmacology and Brain Pathology, Rozzano (Milan), Italy

**Background and Aims:** The immune system and the brain are deeply interconnected, shaping both normal function and disease. This complex crosstalk is key to neuroinflammatory disorders like Multiple Sclerosis (MS), yet studying immune interactions at the neurovascular interface remains a challenge due to the scarcity of infiltrating cells.

**Methods:** To bridge this gap, we developed a microfluidic Neurovascular‐Unit (NVU) model integrating primary human or animal cells, enabling real‐time investigation of immune cell transmigration.

**Results:** When exposed to TNFα, NVU prototypes exhibited reduced TEER, increased permeability, and higher immune infiltration—hallmarks of a neuroinflammatory response. To push the boundaries further, we engineered a personalized human blood‐brain barrier (BBB) model using primary endothelial cells and autologous immune cells from MS patients, allowing patient‐specific insights into immune interactions. In vivo, we leveraged EGFP+ T cell adoptive transfer to track immune cells infiltrating the brain. Transmigrating leukocytes were precisely quantified via real‐time PCR, detecting EGFP+ cells in tissue. For precise immune profiling, we developed an isolation protocol followed by multi‐color flow cytometry, enabling absolute immune cell counting and characterization of subsets crossing brain borders compared to periphery.

**Conclusion:** By integrating these approaches, we provide insight into the immune‐brain axis, paving the way for breakthroughs in neuroimmunology and novel therapeutic strategies for brain disorders.

**Disclosure:** Nothing to disclose.

## OPR‐100

### Safety‐driven ocrelizumab discontinuation vs. continuation: Comparative insights from MSBase

#### E. D'Amico ^1^; T. Spelman^2^; M. Fabis‐Pedrini^3^; A. Kermode^3^; W. Carroll^3^; A. van der Walt^4^; H. Butzkueven^4^; A. Zanghì^1^

##### ^1^University of Foggia, Italy; ^2^MSBase Foundation, VIC, Melbourne, Australia; ^3^Perron Institute for Neurological and Translational Science, The University of Western Australia, Perth, Australia; ^4^Department of Neurology, The Alfred Hospital, Melbourne, Australia

**Background and Aims:** Safety concerns leading to treatment discontinuation pose significant challenges in Multiple Sclerosis (MS) management.

**Methods:** A multi‐center, longitudinal cohort study was conducted using data from the MSBase registry. Patients aged >18 years, diagnosed with relapsing‐remitting MS (RRMS) who had been treated with OCR, for a minimum of one year who recorded one safety event during treatment. Patients were categorized into two groups: those who discontinued Ocrelizumab (OCR) due to safety concerns (Switchers) and those who continued treatment despite adverse events (Continuers). The outcomes of the study were to compare the two groups in terms of total count of relapses, time to first relapse, confirmed‐disability‐worsening (CDW) at 24&48 weeks, and Progression Independent of Relapse Activity (PIRA). Propensity‐score‐matching with inverse‐probability‐weighting (PS‐IPTW) was used to balance baseline characteristics between groups.

**Results:** From an initial cohort of 10,774 patients, 310 Switchers and 1,315 Continuers were identified. After propensity score matching, 66 pairs were analyzed. The mean time on OCR for Switchers was 2.7 ±1.2 years, whilst for the Continuers was 4.4±1.2 years. Infections (43.9%) and cancers (9.1%) were the main safety events leading to discontinuation. Among matched pairs, Switchers had a higher trend in relapse count than Continuers (PS‐IPTW RR 2.62, 95%CI 0.96–7.15, *p* = 0.060). Time to first‐relapse showed no differences. CDW at 24 weeks trended higher for Switchers (HR 2.08, 95% CI 0.96–4.51, *p* = 0.064), with no differences at 48 weeks. PIRA‐risk also trended in Switchers (HR 2.45, 95% CI 0.95–6.29, *p* = 0.063).

**Conclusion:** Switching from OCR due to safety concerns may increase relapse risk, mitigated by transitioning to high‐efficacy DMTs.

**Disclosure:** Tim Spelman received compensation from servicing on steering committees and advisory boards from Biogen Marzena Fabis‐Pedrini received travel compensation from Merck Allan G Kermode Served on Scientific Advisory Boards for Bayer, BioCSL, Biogen‐Idec, Clene Nanomedicine, Esai, Innate Immunotherapeutics, Lgpharma, Merck, Mitsubishi Tanabe Pharma, NeuroScientific Biopharmaceuticals, Novartis, Progenis, Roche, Sanofi‐Aventis, Sanofi‐Genzyme, Teva, and View Health William M Carroll “Recipient of travel assistance and honoraria for participation in industry sponsored meetings from and provided advice to, Bayer Schering Pharma, Biogen‐Idec, Novartis, Roche, Genzyme, Sanofi‐Aventis, CSL, Teva, Merck and Cellgene” Anneke van der Walt served on advisory boards and receives unrestricted research grants from Novartis, Biogen, Merck and Roche She has received speaker's honoraria and travel support from Novartis, Roche, and Merck. She receives grant support from the National Health and Medical Research Council of Australia and MS Research Australia. Helmut Butzkueven received institutional (Monash University) funding from Biogen, Roche, Merck, Alexion and Novartis; has carried out contracted research for Novartis, Merck, Roche and Biogen; has taken part in speakers’ bureaus for Biogen, Novartis, Roche and Merck; has received personal compensation from Oxford Health Policy Forum for the Brain Health Steering Committee.

## OPR‐101

### Contribution of germline mutations to risk of neuromyelitis optica spectrum disorder

#### K. Ogawa ^1^; Y. Tomohiro^1^; G. Sato^2^; T. Naito^3^; K. Sonehara^3^; R. Saiki^4^; R. Edahiro^2^; S. Namba^3^; M. Watanabe^5^; Y. Shirai^2^; K. Yamamoto^2^; M. Kinoshita^1^; M. Niino^6^; Y. Nakatsuji^7^; S. Ogawa^4^; T. Matsushita^8^; J. Kira^9^; H. Mochizuki^1^; N. Isobe^5^; T. Okuno^1^; Y. Okada^3^

##### ^1^Department of Neurology, Osaka University Graduate School of Medicine; ^2^Department of Statistical Genetics, Osaka University Graduate School of Medicine; ^3^Department of Genome Informatics, Graduate School of Medicine, The University of Tokyo; ^4^Department of Pathology and Tumor Biology, Graduate School of Medicine, Kyoto University; ^5^Department of Neurology, Neurological Institute, Graduate School of Medical Sciences, Kyushu University; ^6^Department of Clinical Research, National Hospital Organization Hokkaido Medical Center; ^7^Department of Neurology, Faculty of Medicine, University of Toyama; ^8^Department of Neurology, Kochi Medical School, Kochi University; ^9^Department of Neurology, Brain and Nerve Center, Fukuoka Central Hospital

**Background and Aims:** Neuromyelitis optica spectrum disorder (NMOSD) is a rare autoimmune disease characterized by optic neuritis and transverse myelitis. Due to its low prevalence, the genetic backgrounds of NMOSD have not been elucidated in detail. We performed a large‐scale genome‐wide association study (GWAS) in Japanese to find risk variants of NMOSD.

**Methods:** We conducted a genome‐wide association study (GWAS) meta‐analysis of NMOSD in Japanese (240 patients and 50,578 controls). We applied human leukocyte antigen (HLA) imputation to fine‐map the risk HLA variants. To elucidate the cell‐type‐specific expression profile of the putative target gene, we performed single‐cell RNA sequencing (scRNA‐seq) in peripheral blood cells from 25 NMOSD patients and 101 controls.

**Results:** Our GWAS meta‐analysis identified NMOSD risks in the HLA region and CCR6 (rs12193698; *p* = 1.8 × 10^−8^, odds ratio [OR] = 1.73), a novel associated gene. HLA fine‐mapping showed the strongest association at HLA‐DRβ1 amino acid position 11 with NMOSD. In the scRNA‐seq analysis, the risk variant at CCR6 showed disease‐specific expression quantitative trait loci effects in CD4 memory T cells, especially in T helper 17 (Th17) cells.

**Conclusion:** This is an initial report of the GWAS‐driven NMOSD risk outside the MHC region. We could interpret the GWAS result more efficiently by using the cell‐type‐specific eQTL analysis and demonstrate the genetic regulation underlying the pathogenic role of Th17 cells in NMOSD.

**Disclosure:** Nothing to disclose.

## OPR‐102

### Tumor‐specific CSF B cell response in melanoma patients with leptomeningeal spread

#### M. Kowarik; N. Vasilenko; S. Schembecker

##### Department of Neurology & Stroke and Hertie‐Institute for Clinical Brain Research, Eberhard‐Karls University of Tübingen, Tübingen, Germany

**Background and Aims:** Meningeosis carcinomatosa is a diffuse dissemination of tumor cells into the cerebrospinal fluid (CSF) and occurs in approximately 5% of patients with malignant melanoma. Previous studies on CSF immune cell subsets have found an elevated CSF B‐cell fraction in a subset of these patients. The aim of this study is to evaluate whether these CSF B cells resemble a tumor‐specific immune response.

**Methods:** Single cell analysis of CSF cells was performed in melanoma patients with leptomeningeal spread including whole transcriptome, B cell receptor (BCR) and T cell receptor (TCR) sequencing. After BCR repertoire analysis, recombinant antibodies were generated and tested for antigen specificity using antigen microarrays, flow‐cytometry, ELISA, as well as bioinformatic analyses.

**Results:** We were able to generate representative BCR repertoires in 8 of 9 patients and produced 36 recombinant antibodies. We first tested these antibodies for binding to a melanoma tumor cell line. 8 antibodies were further selected and potential antigen targets were narrowed down by systematic microarrays. Among other potential antigens, specific binding of three antibodies to the individual proteins AKR1A1, KIFC3 and DDX53, a known cancer testis antigen, was detected. Further antibody testing and detailed transcriptome analysis are ongoing.

**Conclusion:** This study provides strong evidence for a targeted intrathecal B cell response against melanoma cells by clonally expanded CSF B cells. Antibody binding against the targets AKR1A1, KIFC3, and DDX53 indicate that the CSF B cell‐derived antibodies may indeed be tumor‐specific. These antibodies and their corresponding tumor antigens may be exploited for future tumor treatments.

**Figure:**
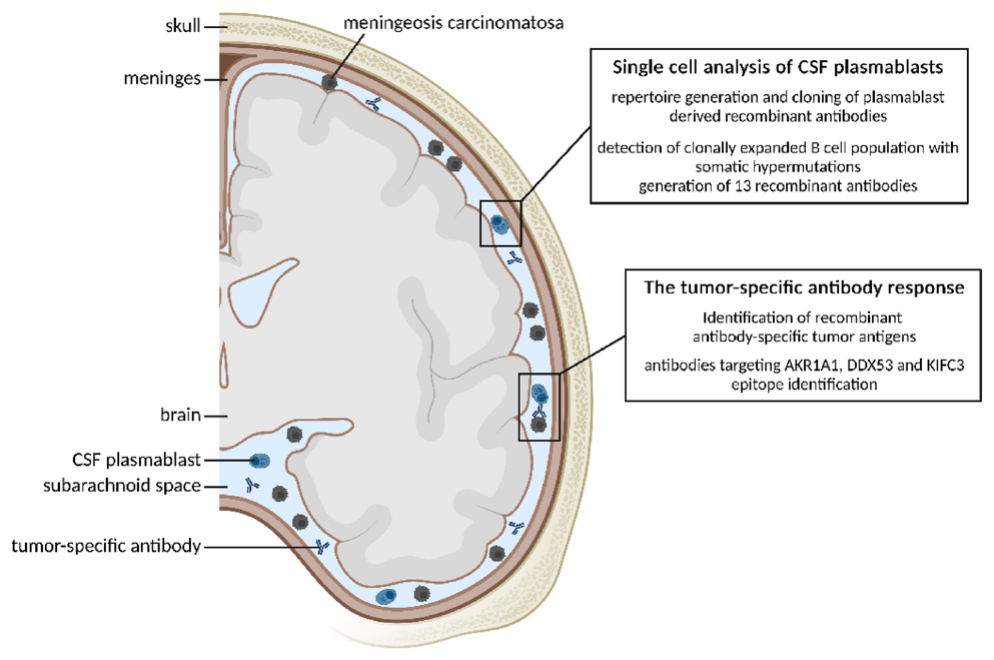
**FIGURE 1** Graphical abstract

**Disclosure:** S.S. has nothing to disclose. N.V. has nothing to disclose. M.K. has served on advisory boards and received speaker fees / travel grants from Merck, Sanofi‐Genzyme, Novartis, Biogen, Janssen, Alexion, Celgene / Bristol‐Myers Squibb and.

## Muscle and Neuromuscular Junction Disorder 2

## OPR‐103

### Differential effect of eculizumab and efgartigimod on subscores of the MG‐ADL and QMG in generalized myasthenia gravis

#### A. Sarnataro; C. Pane; N. Cuomo; A. Bonfini Rendina; G. Puorro; A. Marsili; F. Saccà

##### Federico II University, Naples, Italy

**Background and Aims:** Eculizumab (ECU) and Efgartigimod (EFGA) are both approved for the treatment of generalized Myasthenia Gravis (gMG).

**Methods:** We included 38 patients (22 ECU and 16 EFGA), and retrospectively collected data on the MG activities of daily living (MG‐ADL) and quantitative MG scale (QMG). We limited the observation of the MG‐ADL to weekly scores for the first 8 weeks of treatment, and of the QMG at baseline and after 5, 12, 24, 36, and 48 weeks. We analyzed the difference between treatments at the subscore level of both scales.

**Results:** We found a higher response to ECU at the MG‐ADL at week 7 (−6.3 vs 3.8; *p* = 0.038), and at the QMG at week 24 (−6.0 vs −0.9; *p* = 0.032), 36 (−7.7 vs −1.7; *p* = 0.020), and 48 (−8.5 vs −2.6; *p* = 0.018). We found no differences for the ocular and limb subscores of both the MG‐ADL and QMG. Response for the bulbar subscores at the MG‐ADL (*p* = 0.037) and QMG (*p* = 0.037), was higher with ECU treated patients. For the MG‐ADL, this occurred at week 6 (−3 to 4 vs −1.2, *p* = 0.009), and 7 (−3.1 vs −1.2, *p* = 0.020), and for the QMG at week 12 (−2.1 vs −.8, *p* = 0.025) and 36 (−3.0 vs −1.1, *p* = 0.018). Mean QMG score for the forced vital capacity (FVC) decreased more with ECU throughout the entire observation period (*p* = 0.036).

**Conclusion:** Our study shows a deeper effect of Eculizumab on bulbar scores compared to Efgartigimod. This could be considered when treating patients with high bulbar scores and ventilatory insufficiency.

**Disclosure:** Francesco Saccà received public speaking honoraria from Alexion, argenx, Biogen, Genpharm, Medpharma Madison Neopharm Israel, Pharma, Sanofi, Zai Lab; he also received compensation for Advisory boards or consultation fees from Alexion, Amgen, argenx, AstraZeneca, Alexis, Biogen, Dianthus, Johnson&Johnson, Lexeo, Novartis, Reata, Roche, Sandoz, Sanofi, Takeda, UCB, Zai Lab; he is PI in clinical trials for Alexion, argenx, Dianthus, Immunovant, Lediant, Novartis, Prilenia, Remgen, Sanofi.

## OPR‐104

### Patterns and predictors of therapeutic response to efgartigimod in AChR(+) generalized myasthenia gravis subtypes

#### L. Jin ^1^; Z. Zou^2^; Q. Wang^3^; W. Zeng^4^; Q. Jiang^5^; J. Chen^6^; J. Shi^7^; Y. Yu^8^; D. Hong^8^; Q. Zeng^9^; S. Tan^9^; Y. Yue^10^; Z. Zhang^11^; Y. Zhang^11^; X. Guo^12^; L. Du^13^; Z. Zhao^14^; S. Huang^14^; Y. Chen^15^; Z. Wu^16^; C. Yan^1^; J. Xi^1^; J. Song^1^; S. Luo^1^; C. Zhao^1^

##### ^1^Huashan Rare Disease Center and Department of Neurology, Huashan Hospital, Shanghai Medical College, National Center for Neurological Disorders, Fudan University; ^2^Department of Neurology, Fujian Medical University Union Hospital, Fuzhou; ^3^Department of Neurology, Qilu Hospital of Shandong University, Jinan; ^4^Department of Neurology, Hongkong University Shenzhen Hospital, Shenzhen; ^5^Department of Myopathy, The First Affiliated Hospital of Guangzhou University of Chinese Medicine, Guangzhou; ^6^Department of Neurology, The Second Affiliated Hospital of Soochow University, Suzhou; ^7^Department of Neurology, Nanjing First Hospital, Nanjing Medical University, Nanjing; ^8^Department of Neurology, the First Affiliated Hospital of Nanchang University, Nanchang; ^9^Department of Neurology, Sichuan Provincial People's Hospital, University of Electronic Science and Technology of China, Chengdu; ^10^Department of Neurology, Qilu Hospital (Qingdao), Shandong University, Qingdao; ^11^Department of Neurology, Affiliated Hospital of Xuzhou Medical University, Xuzhou; ^12^Department of Neurology, The First Affiliated Hospital of Chongqing Medical University, Chongqing; ^13^Department of Neurology, the first affiliated hospital of Xinjiang Medical University, Urumqi, Xinjiang Uygur Autonomous Region; ^14^Department of Neurology, Hainan General Hospital, Hainan Affiliated Hospital of Hainan Medical University, Haikou; ^15^Department of Neurology, The First Affiliated Hospital of Wannan Medical College, Wuhu; ^16^Department of Public Health and Primary Care, University of Cambridge, Cambridge CB2 0BB, UK

**Background and Aims:** Efgartigimod is an approved biologic for generalised myasthenia gravis (gMG), which is an autoimmune disease and can potentially be life‐threatening. However, the therapeutic response to efgartigimod among the acetylcholine receptor gMG (AChR‐gMG) subtypes remains inconclusive.

**Methods:** This prospective, observational study included AChR‐gMG patients treated with efgartigimod at 15 centres in China with a follow‐up for at least 20 weeks. The primary outcome was the proportion of MSE responders, denoted by a MG‐ADL score of 0 or 1 within 4 weeks and maintained for ≥4 weeks. AChR‐MG subtypes were classified into EOMG, LOMG, and TAMG. The predictive factors for MSE responders were identified by univariate and multivariate logistic regression analysis.

**Figure:**
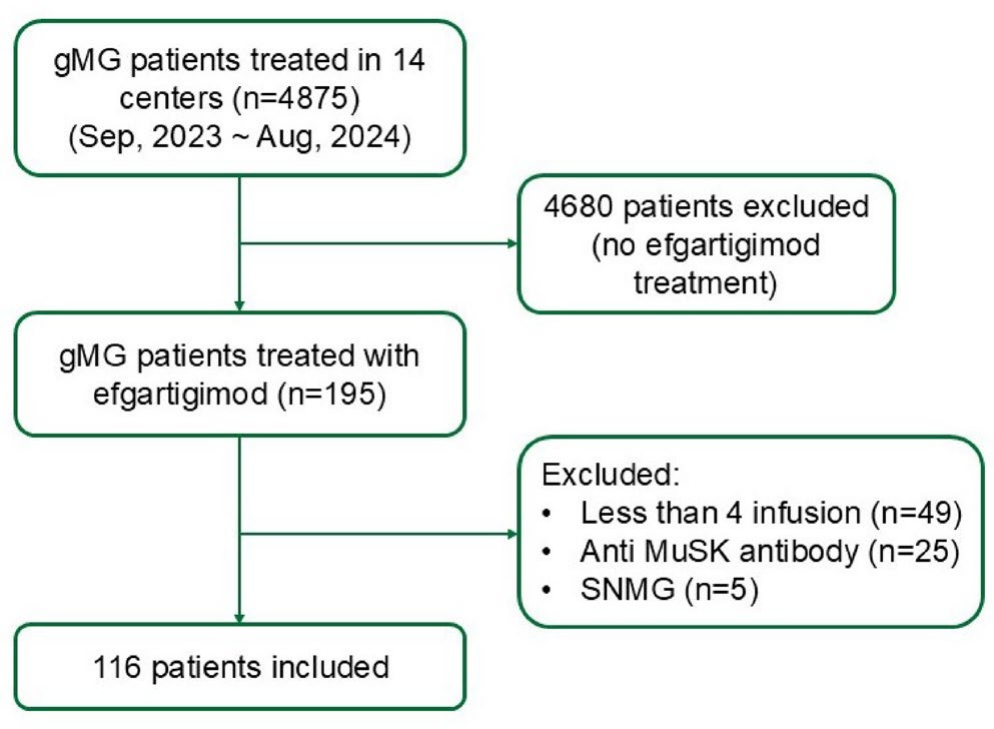
**FIGURE 1** Study flowchart.

**Results:** 116 patients were included with a median follow‐up duration of 238 days (172.5–306.3). There were 50 (43.1%) patients with EOMG, 28 (24.1%) with LOMG, and 38 (32.8%) with TAMG. After efgartigimod initiation, 35 (30.2%) patients were MSE responders, and the proportion of MSE responders was highest in the LOMG group (42.9%). Response patterns to efgartigimod among the AChR‐MG subtypes differed as measured by the proportion of improved patients and MSE. LOMG presented sustained symptom control, while EOMG and TAMG showed more fluctuations. Eight TAMG patients (21.1%) switched to another biologic (*p* = 0.005). Baseline MG‐ADL was an independent predictor for therapeutic response to efgartigimod (*p* < 0.001).

**Figure:**
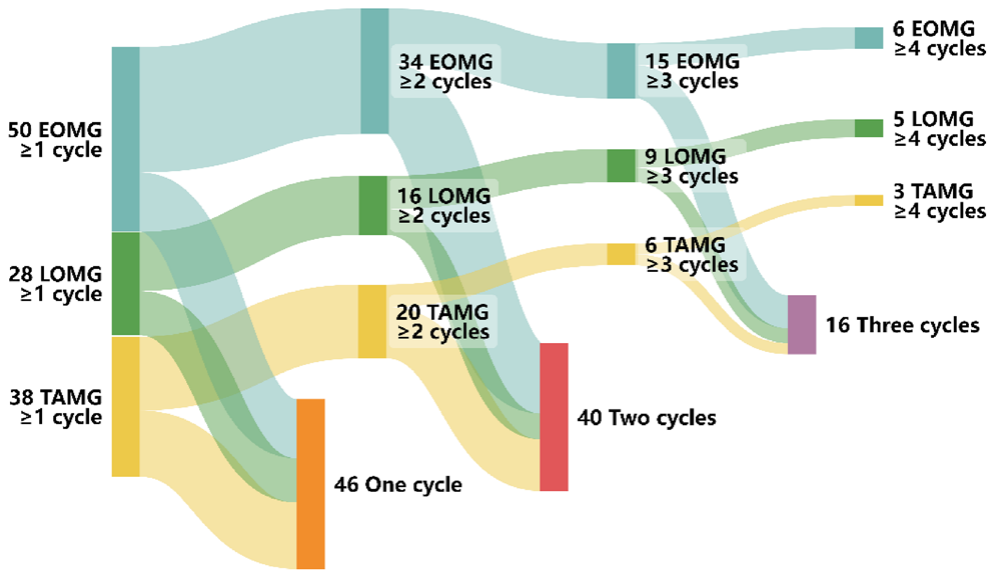
**FIGURE 2** Treatment cycles in different gMG subtypes. EOMG: early‐onset MG; LOMG: late‐onset MG; TAMG: thymoma‐associated MG.

**Figure:**
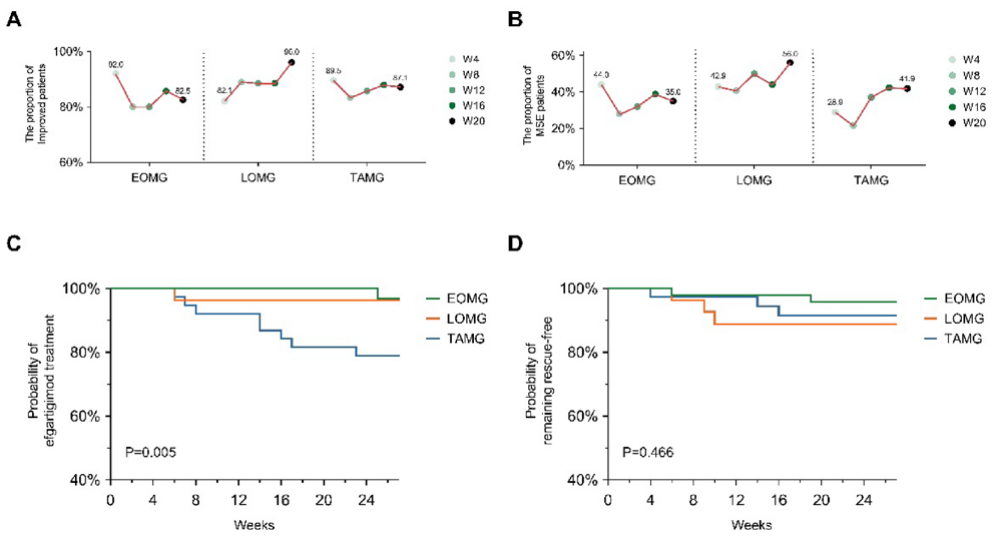
**FIGURE 3** The patterns of therapeutic response among AChR‐MG subtypes. The trajectories of improvement status and MSE varied among the three subtypes (A‐B). Time to treatment switch analysis presented significant differences (*p* = 0.005) (C).

**Conclusion:** Our findings revealed patterns of treatment responses among AChR‐gMG subtypes, with LOMG patients potentially presenting a more sustained response. These findings likely provide preliminary data for precision therapy in MG in the era of biologics.

**Disclosure:** This study is supported by financial grants from the National Key research and development plan (2022YFC3501305, 2022YFC3501303), the National Natural Science Foundation of China (82071410, 82471426), and Shanghai Hospital Development Center Program (SHDC2023CRD007).

## OPR‐105

### Real‐time MRI for accurate quantification of respiratory impairment in Pompe disease

#### L. Töpert ^1^; R. Zeng^1^; O. Al‐Bourini^2^; L. Lettermann^3^; U. Olgemöller^4^; S. Hofer^1^; M. Boentert^5^; T. Friede^6^; M. Nietert^7^; D. Voit^8^; J. Frahm^8^; M. Uecker^9^; A. Seif Amir Hosseini^2^; J. Schmidt^10^

##### ^1^Department of Neurology, University Medical Center Göttingen, 37075 Göttingen, Germany; ^2^Department of Clinical and Interventional Radiology, University Medical Center Göttingen, 37075 Göttingen, Germany; ^3^Institute for Theoretical Physics, Heidelberg University, 69120 Heidelberg, Germany, Bioquant‐Center, Heidelberg University, 69120 Heidelberg, Germany; ^4^Department of Cardiology and Pneumology, University Medical Center Göttingen, 37075 Göttingen, Germany; ^5^Department of Neurology, Münster University Hospital, 48149 Münster; Germany Department of Medicine, UKM‐Marienhospital Steinfurt, Steinfurt, Germany; ^6^Department of Medical Statistics, University Medical Center Göttingen, 37075 Göttingen, Germany; ^7^Department of Medical Bioinformatics, University Medical Center Göttingen, 37077 Göttingen, Germany; ^8^Biomedical NMR, Max Planck Institute for Multidisciplinary Sciences, 37077 Göttingen, Germany; ^9^Institute of Biomedical Imaging, Graz University of Technology, 8010 Graz, Austria; Department of Clinical and Interventional Radiology, University Medical Center Göttingen, 37075 Göttingen, Germany; ^10^Department of Neurology, University Medical Center Göttingen, 37075 Göttingen, Germany; Department of Neurology and Pain Treatment, Immanuel University Hospital Rüdersdorf, Brandenburg Medical School Theodor Fontane, 15562 Rüdersdorf bei Berlin, Germany

**Background and Aims:** Respiratory dysfunction significantly impacts morbidity and mortality in patients with neuromuscular diseases. In late‐onset Pompe disease (LOPD), early stages of respiratory muscle weakness can remain unnoticed due to compensatory breathing capacity. This study aims to evaluate real‐time MRI (RT‐MRI) for the characterization of breathing patterns in LOPD patients compared to standard diagnostic modalities.

**Figure:**
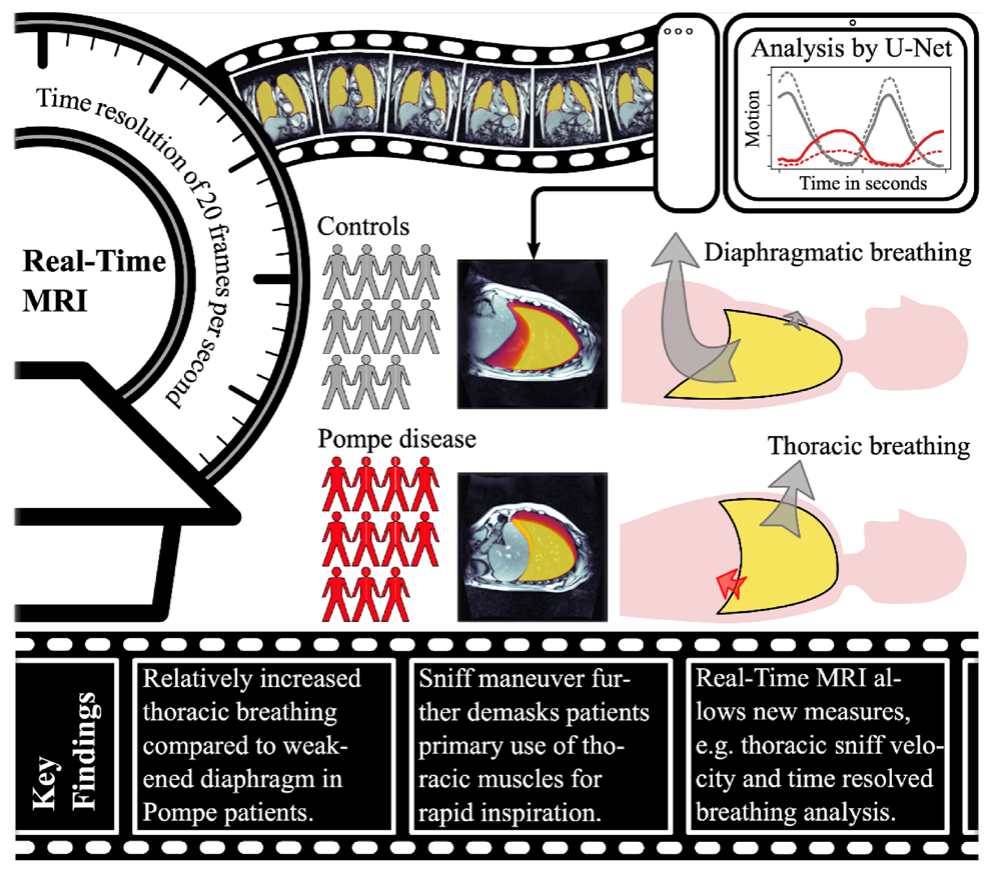
**FIGURE 1** Graphical Abstract of the study with exemplary heath maps of the lungs during sniff maneuver.

**Methods:** Eleven LOPD patients and matched healthy controls underwent RT‐MRI with a temporal resolution of 20 frames per second. Breathing patterns during natural breathing and dynamic respiratory maneuvers were analyzed manually and through U‐Net‐based segmentation (Figure 1). RT‐MRI findings were compared with pulmonary function tests and diaphragm ultrasound. Fast T1 mapping was conducted for non‐invasive assessment of tissue composition of the diaphragmatic crurae. This enabled the comparison of morphological and functional diaphragm characteristics.

**Results:** RT‐MRI precisely quantified reduced diaphragmatic motion in LOPD patients and uniquely revealed compensatory thoracic movement during breathing maneuvers (Figures 1 and 2). In 7 out of 11 patients, the sniff maneuver unmasked paradoxical diaphragmatic movement. U‐Net‐based segmentation facilitated an in‐depth analysis of respiratory mechanics, including diaphragmatic/thoracic synchronicity and velocity during sniff maneuvers. T1 mapping demonstrated fatty infiltration in the diaphragm, which correlated significantly with ultrasound findings of the diaphragm, and with functional respiratory parameters from RT‐MRI and pulmonary function tests.

**Figure:**
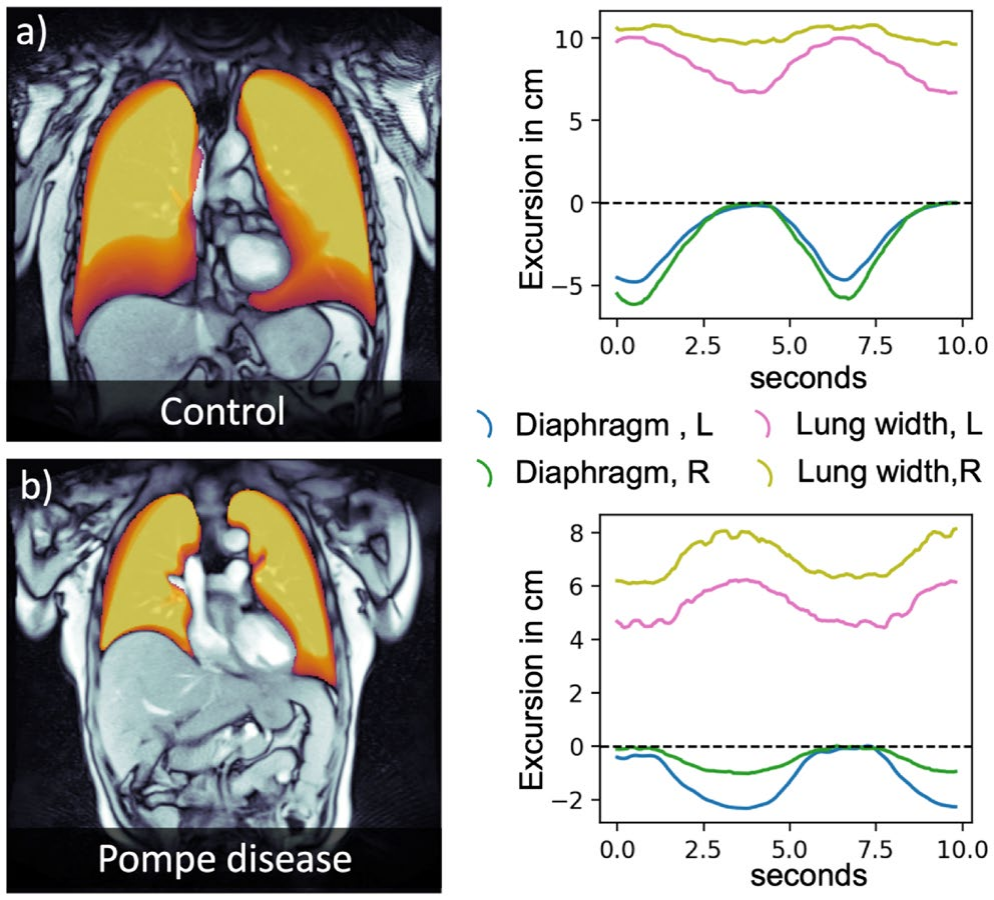
**FIGURE 2** RT‐MRI assessment of diaphragmatic and chest wall motion in patients with late‐onset Pompe disease and controls. Exemplary heath maps (left) and time series (right) show the dynamics of the diaphragmatic motion and chest wall motion (lung width).

**Conclusion:** RT‐MRI provides a detailed and quantitative assessment of respiratory muscle function in LOPD. T1 mapping offers a non‐invasive approach to evaluate diaphragmatic morphology and its functional implications. These imaging techniques hold promise for enhancing early detection and monitoring of respiratory involvement in neuromuscular diseases.

**Disclosure:** Jens Frahm, Martin Uecker and Dirk Voit are co‐inventors and patent holders of the software describing the real‐time MRI technique used here. The remaining authors declare no conflict of interest. RZ and JS are members of the European Reference network for rare Neuromuscular disorders (ERN EURO‐NMD). This work incorporates the results of the dissertation submitted by LT at the Faculty of Medicine of the University of Goettingen. Funding by German patient support group (DGM) (application number Sc21/2) and German Research Foundation (DFG) within the Clinician Scientist Program “Cell Dynamics in Disease and Therapy” at the University Medical Center Goettingen (project number 413501650).

## OPR‐106

### Motor Unit Number Index (MUNIX) as a biomarker of disease progression in myasthenia gravis

#### L. Bevilacqua ^1^; C. Noioso^2^; G. Acerra^2^; S. Avventura^2^; A. Iovino^1^; G. Piscosquito^1^; A. Toriello^1^; P. Barone^2^; C. Vinciguerra^1^

##### ^1^Neurology Unit, University Hospital “San Giovanni di Dio e Ruggi d’Aragona”, Salerno, Italy; ^2^University of Salerno, Department of Medicine Surgery and Dentistry, “Scuola Medica Salernitana”, Neuroscience section, Salerno, Italy

**Background and Aims:** Myasthenia gravis (MG) is an autoimmune disorder causing impaired neuromuscular transmission. Currently, no neurophysiological biomarker of disease progression is available. This pilot study evaluates the role of the motor unit number index (MUNIX) as biomarker of motor unit function and disease monitoring in MG, by comparing MUNIX values between MG patients and healthy controls (HCs), alongside their correlations with clinical and electrophysiological measures.

**Methods:** Compound motor action potential (CMAP), MUNIX, and motor unit size index (MUSIX) were assessed in the abductor pollicis brevis (APB) and orbicularis oculi (OO) muscles in the symptomatic or non‐dominant side. Clinical severity was evaluated using MGCS, MG‐ADL, and MGFA classification. Statistical analyses included ANOVA, Spearman correlations, and multivariate analysis (*p* < 0.05).

**Results:** 42 MG patients (63.1 ± 12.7 years) and 21 age‐matched HCs (59.7 ± 8.7 years) were enrolled. MUNIX values were lower in MG patients than HCs for APB (*p* = 0.022) and OO (*p* = 0.028), while MUSIX was higher (APB: *p* = 0.047; OO: *p* = 0.002). Age negatively correlated with CMAP APB (*p* = 0.010) and MUNIX APB (*p* = 0.033) but positively with MUSIX (*p* = 0.045 for APB, *p* = 0.020 for OO). Disease duration negatively correlated with CMAP, MUNIX, and MUSIX, while MuSK serotype showed lower CMAP ORB values (*p* = 0.001). CMAP ORB (*p* = 0.001) and MUSIX ORB (*p* = 0.002) predicted disease duration.

**Figure:**
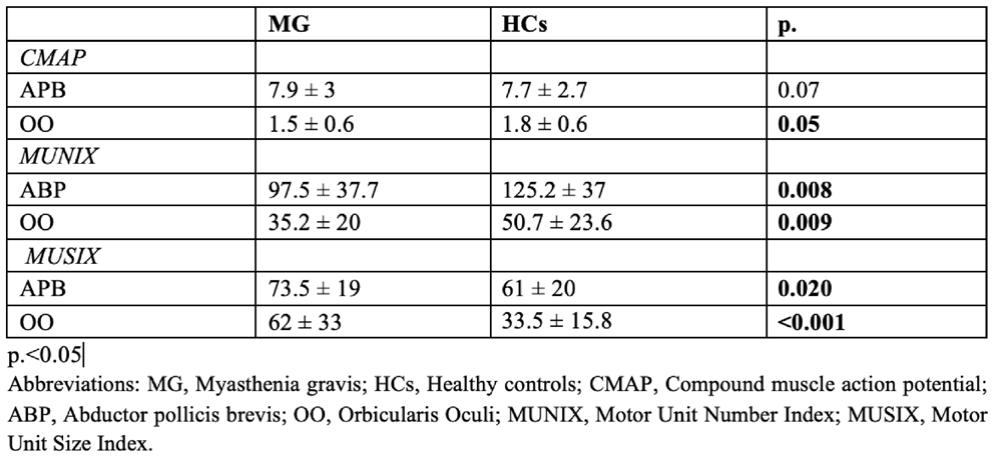
**TABLE 1** Electrophysiological comparison between MG and HCs.

**Figure:**
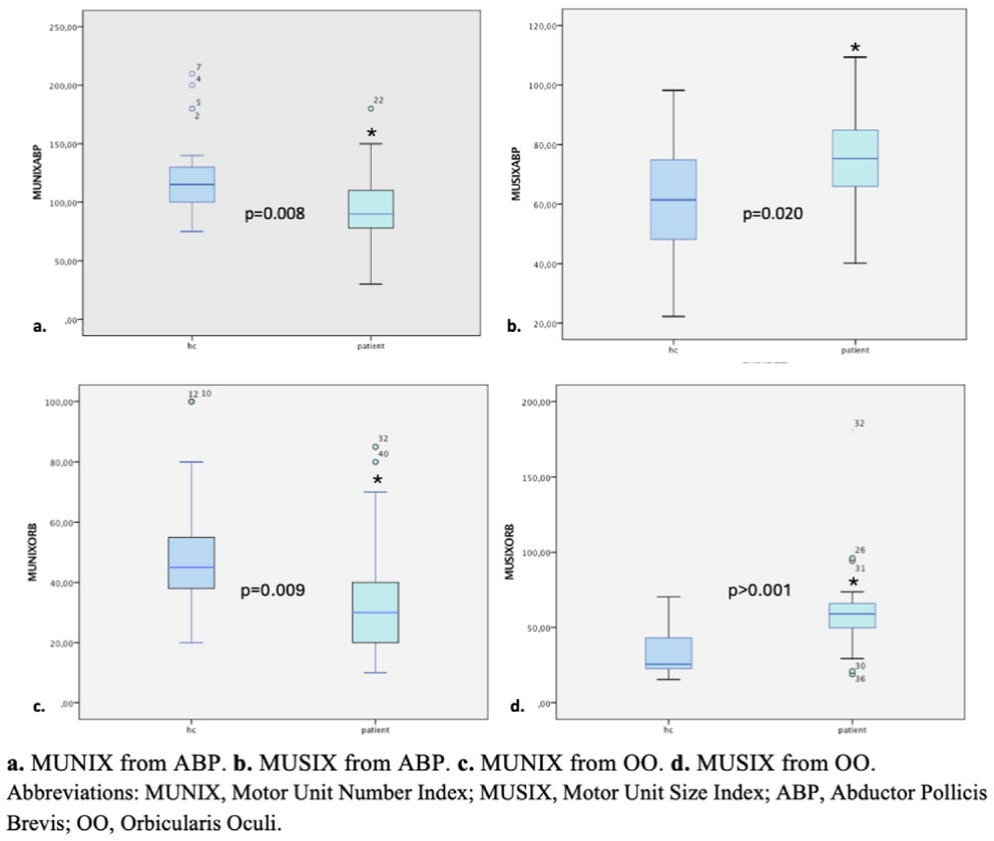
**FIGURE 1** Comparison of MUNIX and MUSIX values between MG patients and HCs.

**Figure:**
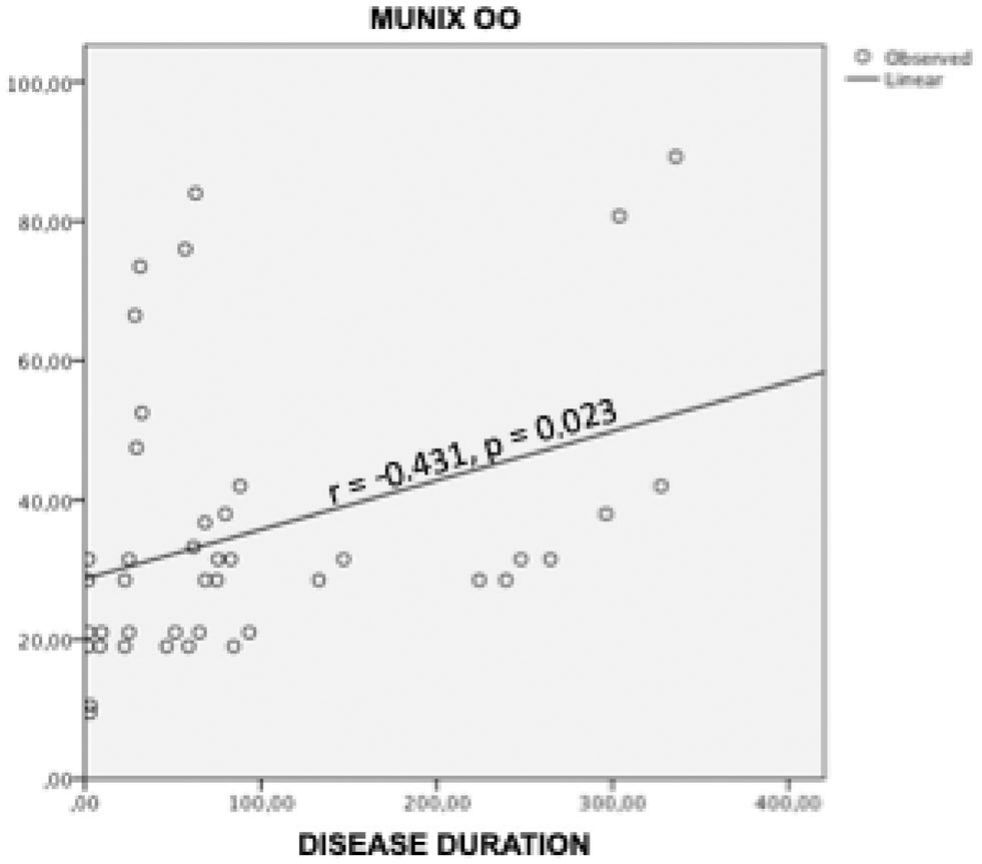
**FIGURE 2** Univariate analysis showing significant negative correlation between MUNIX (OO) and disease duration.

**Conclusion:** MG patients showed reduced CMAP and MUNIX, indicating motor unit loss and impaired neuromuscular transmission, while higher MUSIX suggests compensatory remodeling. Correlations with disease duration and clinical severity underscore MUNIX's potential in disease monitoring. Further research with larger cohorts and longitudinal designs are warranted.

**Disclosure:** Nothing to disclose.

## Neuro‐opthalmology/Neuro‐otology

## OPR‐107

### The supporting role of visual evoked potentials for the diagnosis of optic neuritis

#### G. Greco ^1^; E. Rigoni^2^; F. Masi^2^; M. Todisco^3^; G. Cosentino^1^; E. Caverzasi^4^; A. Pichiecchio^1^; M. Terzaghi^1^; E. Colombo^2^; M. Gastaldi^5^

##### ^1^Department of Brain and Behavioural Sciences, University of Pavia, Pavia, Italy; ^2^Multiple Sclerosis Centre, IRCCS Mondino Foundation, Pavia, Italy; ^3^Neurophysiology Unit, IRCCS Mondino Foundation, Pavia, Italy; ^4^Advanced Imaging and Artificial Intelligence Center, Department of Neuroradiology, IRCCS Mondino Foundation, Pavia, Italy; ^5^Neuroimmunology Laboratory, IRCCS Mondino Foundation, Pavia, Italy

**Background and Aims:** Visual evoked potentials (VEPs) are commonly employed in the assessment of optic neuritis (ON). The International Consortium on Optic Neuritis (ICON) diagnostic criteria only included MRI and optical coherence tomography (OCT) as supportive paraclinical tests, as VEPs were considered non‐specific. We aimed to assess the diagnostic performance of VEPs in a large consecutive cohort of patients with suspected ON.

**Methods:** We screened 207 consecutive patients with suspected ON and eventually included 71 with available clinical, MRI, OCT and VEPs data within 30 days of clinical onset. ON diagnosis was established according to the judgment of two expert neuro‐ophthalmologists after exclusion of other causes. We calculated diagnostic performance measures for each test and for the ICON criteria with and without VEPs as an additional paraclinical test.

**Results:** VEPs were the most sensitive paraclinical test (91.5%) with good specificity (83.3%). Positive and negative predictive value (PPV, 91.5%; NPV, 83.3%) were also high. MRI showed the highest specificity and PPV (87.5% and 91.9%). VEPs also had the highest overall diagnostic accuracy (88.7%) and area under curve in the ROC analysis (0.89) followed by OCT (78.9%, 0.78) and MRI (77.5%, 0.80). The addition of VEPs improved ICON criteria sensitivity (95.7% to 100.0%, identifying two additional patients) and NPV (89.5% to 100.0%), maintaining the same diagnostic accuracy (87.3%), although lowering their specificity (70.8% to 62.5%).

**Figure:**
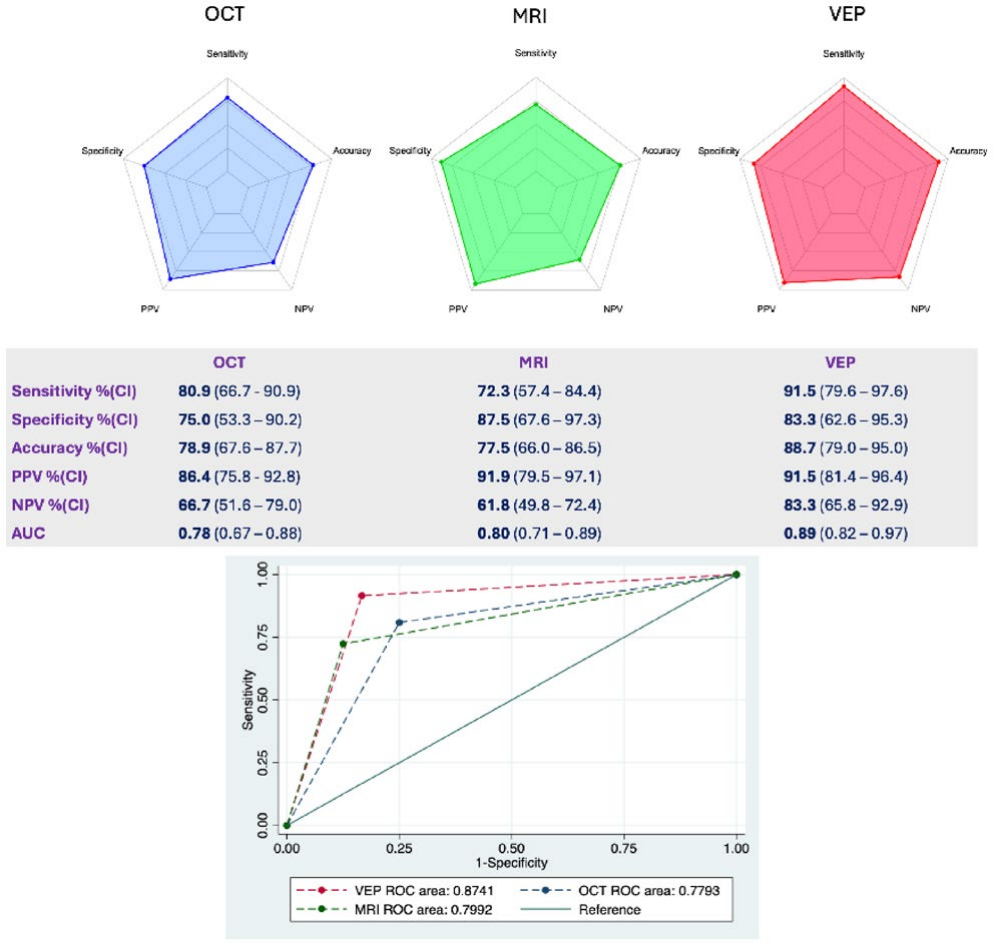
**FIGURE 1** Diagnostic measures and receiver operating curves for OCT, MRI and VEPs.

**Figure:**
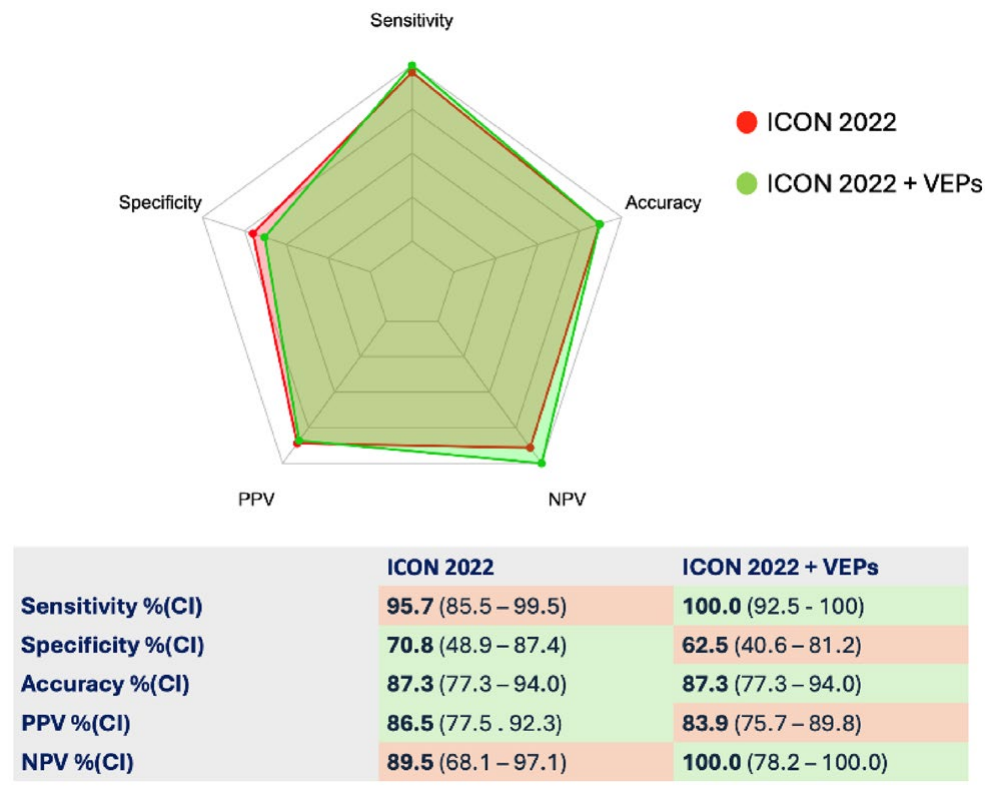
**FIGURE 2** Diagnostic performance of the ICON criteria with and without VEPs as a further paraclinic test.

**Conclusion:** VEPs can identify additional cases of ON that would remain undiagnosed with the current criteria and could be helpful in selected cases with high pre‐test probability.

**Disclosure:** Nothing to disclose.

## OPR‐108

### Eye movements mirror vertigo symptoms in vestibular migraine

#### M. Villar‐Martinez ^1^; A. Bronstein^2^; P. Castro‐Abarca^3^; F. Greenwood^1^; S. Maniataki^1^; F. Puledda^1^; D. Kaski^3^; D. Moreno‐Ajona^1^; P. Goadsby^1^

##### ^1^Wolfson Sensory Pain and Regeneration, Institute of Psychiatry, Psychology and Neuroscience, King's College London, UK; ^2^Centre for Vestibular Neurology. Imperial College London; ^3^Centre for Vestibular and Behavioural Neurosciences. University College Hospital London

**Background and Aims:** Interpreting vertiginous symptoms is challenging and crucial for understanding pathophysiological mechanisms. We aimed to correlate video‐oculographic findings with vertigo symptoms in vestibular migraine (VM).

**Methods:** Ten healthy participants (HP) and 10 participants with VM, diagnosed per Bárány Society and ICHD‐3 criteria, underwent neuro‐otological assessment and 3D video‐oculography in primary gaze with and without visual fixation, for 20 seconds each. Subjective reports during ocular motor assessment were recorded. Nitroglycerin (0.5 μg/kg) was administered to one patient and one healthy participant as part of the NITROVest study (IRAS 312478).

**Results:** VM participants described several spinning, oscillating and sliding sensations across axes and planes, pulsions and unsteadiness. All participants had stable gaze with, and ocular drifts without, fixation. In VM, horizontal (cases 1,4–9) or vertical drifts and nystagmus (1–3, 5–6, 8–10) aligned with the reported vestibular sensations. Nitroglycerin induced spinning symptoms in the yaw axis in a VM (case 1), corresponding to a moderate‐amplitude fast‐phase left‐beating nystagmus, and no symptoms in the HP (case 11). A VM participant had migraine on the assessment day with a 2‐second backwards spinning sensation coinciding with moderate‐amplitude fast‐phase upbeat nystagmus, which she identified as typical of her “roller‐coaster” symptom (case 9).

**Conclusion:** The minor findings observed, even if not frankly abnormal, show a remarkable oculo‐perceptual congruence. Motion illusions in VM may result from altered central modulatory mechanisms amplifying eye movements, leading to the perception of self‐motion. Thorough characterization of subjective motion symptoms and their correlation with objective ocular‐motor findings could be key for understanding VM.

**Disclosure:** None of the authors have relevant disclosures for this abstract.

## OPR‐109

### Head circumduction – A simple technique to examine peripheral and central function of the torsional VOR

#### M. Corrado ^1^; B. Nicklen^2^; Y. Lì^2^; T. Ellmers^2^; A. Bronstein^2^

##### ^1^IRCCS Mondino Foundation, Movement Analysis Research Section, Pavia, Italy; ^2^Department of Brain Sciences, Charing Cross Hospital, Imperial College, London

**Background and Aims:** We introduce a novel clinical paradigm to induce the torsional vestibulo‐ocular reflex (VOR) using head circumduction ‐ a circular motion of the head combining neck extension, rotation and flexion. We (i) evaluated the reliability of this manoeuvre in generating torsional nystagmus, (ii) explored the impact of post‐rotational head tilt on induced responses (iii) assessed this paradigm as a clinical test of vestibular function.

**Methods:** Fourteen healthy participants were tested using an eye‐tracker to record eye movements induced by the circumduction, performed at a frequency of 0.75 Hz. Participants were recorded on stopping in either a head‐up or head‐down condition. Exploratively, we also tested two patients with bilateral vestibular failure (BVF).

**Results:** All healthy participants showed robust post‐rotational torsional nystagmus. This was significantly shorter during head‐up (duration = 10.7±2.4 s) compared to head‐down (duration = 15.7±3.7 s; *p* = 0.0001). Vertical nystagmus was also observed in most healthy participants, which was either disconjugate or overtly skewed. The two patients with BVF did not show any post‐rotational nystagmus.

**Conclusion:** The shortening of torsional nystagmus duration and time constant in the head‐up position supports (i) a role for the velocity storage mechanism in the torsional VOR (which was previously disputed) and (ii) the existence of otolith dumping effects in the torsional VOR. The vertical ocular findings during the stopping response confirm that skewed eye movements can be generated by vertical semicircular canal activity. These findings show that head circumduction is a simple method for assessing the torsional VOR – further supported by the lack of post‐rotational response in BVF patients.

**Figure:**
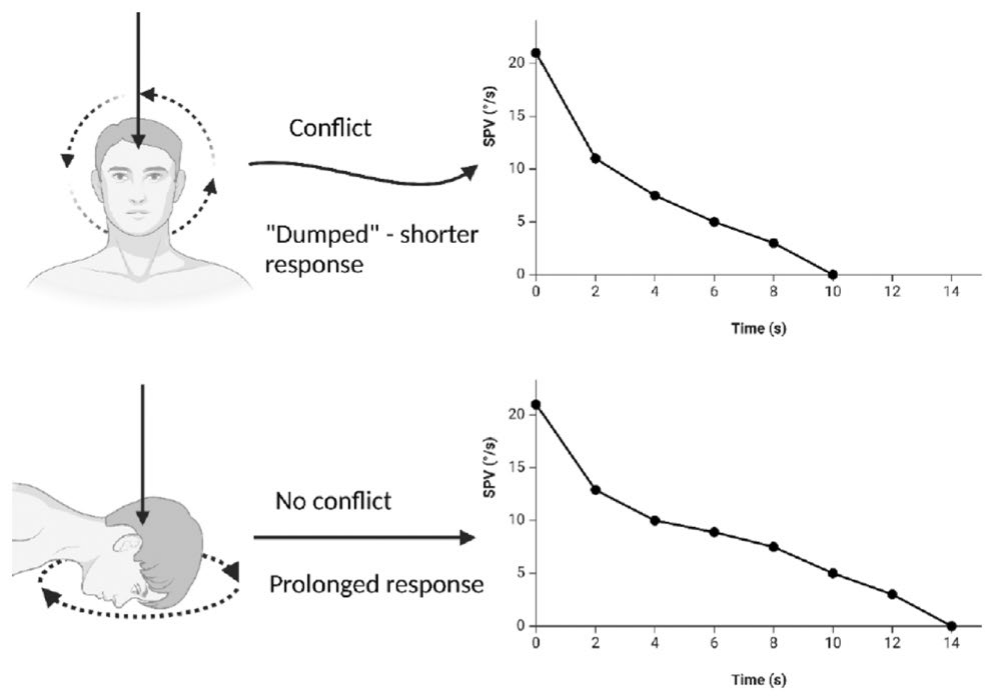
**FIGURE 1** Stopping response with vertical canal stimulation after circumduction: upright head position causes a conflict between dynamic canal signals (rolling) and static otolith signal, shortening nystagmus. Forward head tilt aligns signals, reducing the conflict.

**Disclosure:** Nothing to disclose.

## OPR‐110

### Vestibular Migraine: Course of symptoms during a four‐year follow‐up

#### N. Celebisoy ^1^; A. Kisabay Ak^2^; H. Ozdemir^1^; F. Gokcay^1^; A. Saritas^2^

##### ^1^Ege University Department of Neurology Izmir, Turkey; ^2^Celal Bayar University, Department of Neurology, MANISA/TURKEY

**Background and Aims:** Data about the prognosis of vestibular migraine (VM) is scarce.

**Methods:** VM patients on follow‐up for at least four years were included in this multicenter study to evaluate the course of symptoms. A structured questionnaire was used inquiring demographic features, age of onset of migraine headaches and vertigo attacks, headache and vertigo attack frequency, severity, associated features and the presence of interictal dizziness and positional vertigo. Menopause, history of motion sickness, family history of migraine was recorded. Answers of the first visit were compared with the answers of the last visit. In addition, variables considered were evaluated regarding their effect on symptom course.

**Results:** 203 patients were studied. Median vertigo and headache attack frequency and severity had significantly dropped during follow‐up (*p* < 0.01 for all comparisons). Complete resolution was reported by only 5.4%. Dizziness in between the attacks was present in 67% and positional vertigo was reported by 20.2%. Univariate analysis showed that aural symptoms (*p* = 0.013) and menopause (*p* = 0.016) were risk factors for ongoing frequent vertigo attacks. History of motion sickness (*p* = 0.019) and a family history of migraine (*p* = 0.004) were associated with the risk of frequent migraine headaches. Presence of allodynia (*p* = 0.002) was associated with severe headache attacks when an early age of onset of vertigo attacks (*p* = 0.005) was a risk factor for continuing high frequency vertigo attacks.

**Conclusion:** In conclusion, though the frequency and severity of the headache and vertigo attacks decrease, complete resolution is reported by a minority

**Disclosure:** Nothing to disclose.

## Sleep‐wake Disorders 2

## OPR‐111

### Nightmares accelerate biological aging and predict premature mortality in humans

#### A. Otaiku

##### UK Dementia Research Institute, Imperial College London, London, UK

**Background and Aims:** Nightmares are associated with an increased risk of developing neurodegenerative diseases. Whether nightmares increase the risk of other age‐related health outcomes is unknown. This study investigated whether nightmares increase the risk of premature mortality and accelerated biological aging in the general population.

**Methods:** Data from 4,196 participants (ages 26–74) from four population‐based cohort studies (Midlife in the United States [MIDUS]; MIDUS Refresher; Wisconsin Sleep Cohort; The Osteoporotic Fractures in Men Study) were used in this longitudinal analysis. Nightmare frequency was self‐reported at baseline. Premature all‐cause mortality (age < 75 years) was defined using study records. Cox regression was used to examine the prospective association between nightmare frequency and premature mortality. Participants' biological aging rates were measured at baseline using a composite of three epigenetic clocks (DunedinPACE, GrimAge, PhenoAge). Mediation analysis was performed to determine whether accelerated biological ageing mediates the nightmare‐mortality association.

**Results:** During 18‐years of follow‐up, 227 premature deaths occurred. A higher frequency of nightmares was linearly associated with a greater risk of premature death (*p* < 0·001). Compared with adults who had no nightmares at baseline, those who reported having weekly nightmares had a 3‐fold risk of premature mortality (adjusted hazard ratio = 2·73; *p* < 0.001). Furthermore, individuals with a higher frequency of nightmares exhibited faster rates of biological aging (*p* < 0.001). Accelerated biological ageing mediated 39% of the nightmare‐mortality association.

**Conclusion:** Adults with frequent nightmares experience faster biological aging and die at younger ages. Future studies are needed to determine whether treating nightmares could slow biological ageing and reduce mortality risk in the general population.

**Disclosure:** Nothing to disclose.

## OPR‐112

### Actigraphy for wake‐sleep rhythm characterization in patients with post‐traumatic confusional state

#### A. Comanducci ^1^; T. Atzori^1^; C. Derchi^1^; P. Arcuri^1^; C. Valota^1^; A. Bellinvia^1^; P. Trimarchi^1^; G. Ferrarazzo^1^; A. Caronni^2^; M. Rabuffetti^1^; J. Navarro Solano^1^

##### ^1^IRCCS Fondazione Don Carlo Gnocchi ONLUS, Milan, Italy; ^2^Department of Biomedical Sciences for Health, University of Milan, Milan, Italy

**Background and Aims:** Post‐traumatic confusional state (PTCS) is a syndrome that often occurs after a severe traumatic brain injury (TBI). PTCS is characterized by cognitive impairments, disorientation, attention fluctuations, sleep‐wake disturbances, agitation and psychotic‐like symptoms, and represents a critical phase of recovery, with its duration closely linked to long‐term functional outcomes. Sleep‐wake rhythm disturbances may exacerbate the cognitive and functional impairments seen in these patients. This study evaluates actigraphy‐based metrics for sleep‐wake rhythm profiling in PTCS and their potential to predict recovery trajectories.

**Methods:** We included 28 patients with severe TBI undergoing rehabilitation. Seven‐day actigraphy was used to measure sleep efficiency (SE), wake efficiency (WE), and the expected wake‐sleep rhythm (EWSR). Clinical assessments, including the Confusion Assessment Protocol (CAP), Barthel Index, and agitation scores, were performed at T0 (admission) and T1 (discharge).

**Results:** Actigraphy metrics were significantly altered compared to healthy reference values in the whole TBI‐cohort. SE, WE and EWSR were more impaired in confused patients than in controls. Moderate‐severe agitation was associated with greater SE and EWSR impairment. Using SE = 85% as cut‐off, the CAP sleep item failed to identify 15 out of 22 patients with impaired SE, resulting in a 71% false‐negative rate, underscoring its limitations in identifying objective sleep disturbances. Univariate analyses identified WE and EWSR as significant predictors of PTCS persistence and duration, respectively. Finally, a significant correlation was observed between WE and Barthel Index improvement at T1.

**Conclusion:** Actigraphy can provide objective, clinically relevant metrics to assess sleep‐wake rhythm disturbances in PTCS, offering predictive insights for rehabilitation outcomes.

**Disclosure:** This work is supported by the Italian Ministry of Health—(Ricerca Corrente 2025–2027).

## OPR‐113

### Impacts of physical exercise and keto diet on idiopathic hypersomnia, narcolepsy type 2: A randomized, controlled trial

#### H. Cintosun ^1^; F. Tepel^1^; D. Borth^1^; F. Hanakam^2^; H. Lemberger^3^; V. Vael^4^; D. Bijlenga^4^; G. Lammers^4^; U. Kallweit^1^

##### ^1^Center for Narcolepsy and Hypersomnias, Professorship for Narcolepsy and Hypersomnolence Research, Department of Medicine, University Witten/Herdecke, Witten, Germany; ^2^Fakultät für Sportwissenschaft, Ruhruniversität Bochum; ^3^Diplom‐Oecotrophologin, Ernährungsberaterin, Institut für Sport und Bewegungsmedizin der Universität Hamburg; ^4^Leiden University Medical Centre, Department of Neurology, Leiden, The Netherlands, and Sleep Wake Centre SEIN, Heemstede, The Netherlands

**Background and Aims:** Behavioural treatment recommendations for narcolepsy type 2 (NT2) and idiopathic hypersomnia (IH) are mainly based upon expert opinion. The aim of this study is to investigate the effects of regular physical activity and ketogenic diet in NT2 and IH.

**Methods:** In our 10‐week trial adult patients with NT2 and IH were randomized into two intervention groups: ketogenic diet, and regular physical exercise following a training plan, and a control group. Study parameters included demographic and clinical data, ketone levels, power‐walking tests, and questionnaires on daytime sleepiness (ESS), sleep quality (PSQI), fatigue (FSMC), well‐being, recovery and life quality (SF‐12, WHO‐5).

**Results:** In total 45 patients with IH (*n* = 19) and NT2 (*n* = 26) were randomized, 34 completed the study. In the IH‐Keto group (*n* = 7), daytime sleepiness improved significantly, with a reduction of 30% after 10 weeks of intervention (ESS1: 16 vs. ESS2: 9). Fatigue and quality of life also showed significantly improvements only in this group. In NT2, significant improvements were observed in the Keto group for sleep quality, physical health, and quality of life, while only a positive trend in daytime sleepiness, fatigue and sleep quality was noted in the exercise group. Additionally, an average weight loss of 5 kg was observed in both Keto groups.

**Conclusion:** The ketogenic diet showed a strong treatment effect in IH and can be considered an effective non‐drug therapy. Both ketogenic diet and regular physical exercise also indicate improvements for individual parameters.

**Disclosure:** The authors declare no conflict of interest.

## OPR‐114

### Measuring REM‐sleep without atonia (RWA) on v‐PSG: Does the scoring method make a difference? (preliminary data)

#### M. Muntean ^1^; S. Hink^1^; T. Weiberg^1^; R. Funk^1^; A. Stefani^2^; M. Cesari^2^; B. Högl^2^; C. Trenkwalder^1^

##### ^1^Paracelsus Elena Hospital, Kassel, Germany; ^2^Department of Neurology, Medical University of Innsbruck, Austria

**Background and Aims:** Within the multicentric BRAVA Project, REM‐Sleep without atonia (RWA) was measured using an adapted protocol following the SINBAR criteria. We compared RWA measurements using the BRAVA protocol with the AASM criteria for scoring RWA to analyse differences in RWA indices.

**Methods:** We analysed v‐PSG data from a subgroup of 14 subjects from the centre in Kassel: 3 Parkinson Disease (PD) without RBD, 4 PD with RBD, 4 iRBD and 3 controls. RWA was measured using AASM criteria for the entire REM Sleep and the BRAVA protocol‐REM‐sleep periods were scored and a simplified version of the SINBAR criteria quantifying RWA in 3‐s mini‐epochs as “any” chin activity and/or phasic FDS activity (without specifications of the side) was applied.

**Results:** The study group consisted of 5 (36%) men and 9 women (64%), with a mean age of 63,43± 15,96 years. RWA indices did not significantly differ between the two methods (37,95 ± 30,53% with BRAVA protocol vs 38,11 ± 30,36% with AASM, *p* = 0.99). When using the BRAVA protocol 6 (43%) subjects presented RWA above the cutoff of 31,9%, compared to 8 (57%) subjects above the AASM cutoff of 27,2% (*p* = 0.43). With the BRAVA protocol fewer 3s‐mini‐epochs (669,71± 547,14) were analysed compared to the AASM criteria (990,93 ± 541,67).

**Conclusion:** When measuring RWA using the BRAVA protocol a number of the REM 3s‐mini‐epochs was excluded. Nonetheless the two methods resulted in a similar amount of total RWA. Further data with more subjects in different diagnosis groups is currently analysed.

**Disclosure:** This project was supported in part by the Austrian Science Fund [grant DOI: 10.55776/I5894] and by Bundesministerium für Bildung und Forschung, Projektträger: Deutsches Zentrum für Luft‐ und Raumfahrt e.V. (01KU2206), under the frame of ERA PerMed.

## OPR‐115

### Exploring the neural correlates of insomnia: A meta‐analysis of functional and structural alterations

#### T. Li; Y. Fang; C. Pan; Z. Zhu

##### Department of Neurology, Tongji Hospital, Wuhan, China

**Background and Aims:** Insomnia is one of the most common sleep disorders, and a large number of neuroimaging studies have indicated abnormalities in the brain's function and structure in individuals with insomnia. However, the heterogeneity of these findings has hindered the understanding of the underlying mechanisms of insomnia. Increasingly, there is a growing recognition that localizing the disease to brain networks is more informative than focusing on individual anatomical regions.

**Methods:** We included 53 previously published studies with a total of 66 comparisons. Using a novel functional connectivity network mapping method, we combined the resting‐state functional connectivity database from 1,000 healthy volunteers to map the affected coordinates from these studies onto three brain networks.

**Figure:**
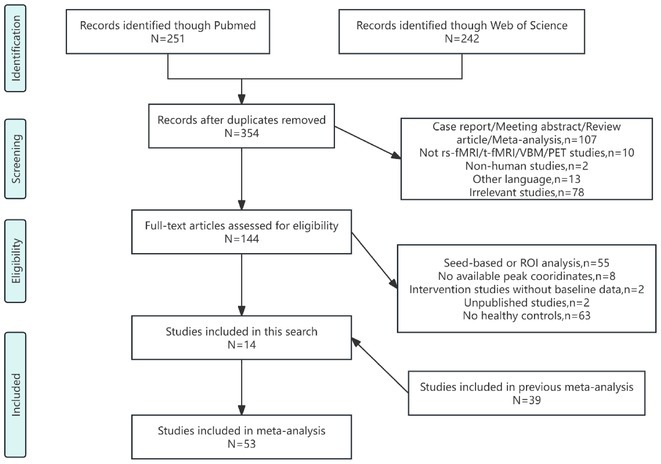
**FIGURE 1** Study selection strategy flowchart.

**Results:** The resting‐state, task‐state, and gray matter volume networks in insomnia are interrelated yet distinct. Specifically, all three networks are significantly associated with the cingulo‐opercular network. Additionally, the resting‐state network is closely linked to the default mode network and the salience network. The task‐state network is primarily associated with the ventral attention network, while the gray matter volume network is mainly connected to the ventral/dorsal attention networks and the somatomotor network.

**Figure:**
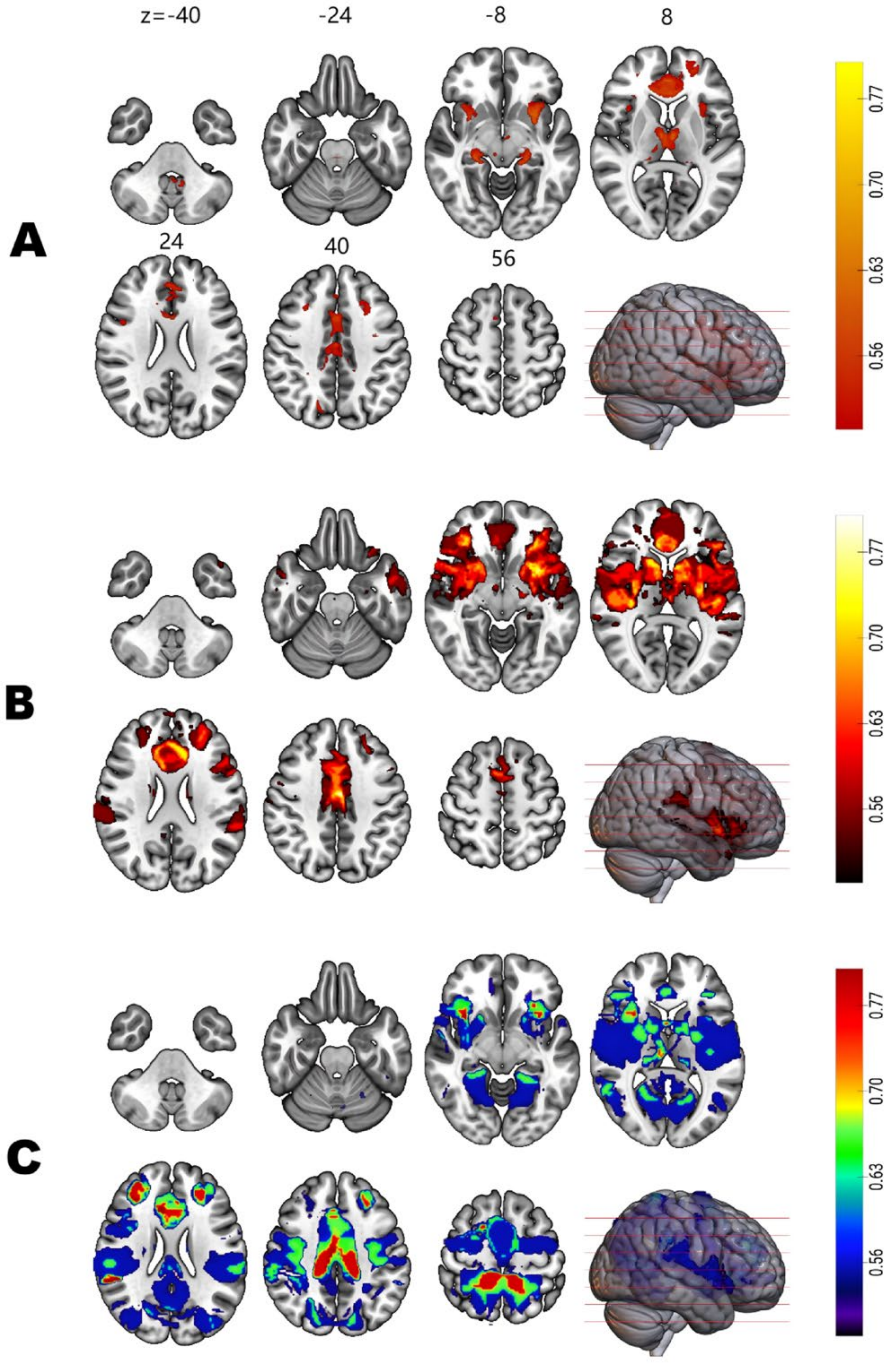
**FIGURE 2** Network localization of insomnia in resting‐state (A), task‐state (B), and gray matter volume (C), with the network threshold set between 50% and 80% as shown in the figure.

**Figure:**
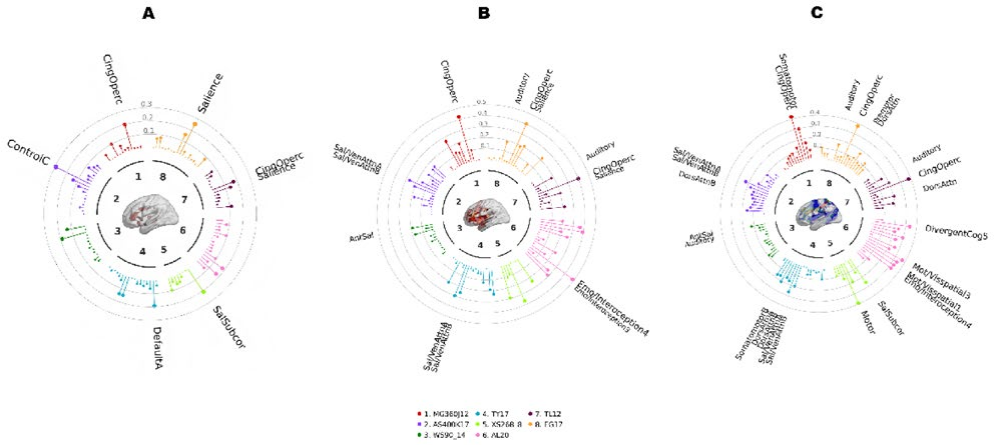
**FIGURE 3** Overlap analysis of insomnia networks in resting‐state (A), task‐state (B), and gray matter volume (C) with classical brain network atlases. Named networks are significantly associated with abnormal insomnia networks.

**Conclusion:** Our meta‐analysis integrates previous inconsistent findings from the perspective of network localization, which not only helps to elucidate the unique neurobiological mechanisms of insomnia but also provides insights for further research and clinical interventions in insomnia.

**Disclosure:** Nothing to disclose.

